# Assembly of 2‑Substituted Tetrahydroquinolines from *ortho*-Methylbenzenesulfamides and Dienes, Using a C(sp^3^)–H Activation/Annulation Sequence

**DOI:** 10.1021/acs.orglett.4c02292

**Published:** 2024-09-11

**Authors:** Iván Huertas-Morales, Borja Cendón, Domingo Costa, José Luis Mascareñas, Moisés Gulías

**Affiliations:** Centro Singular de Investigación en Química Biolóxica e Materiais Moleculares (CiQUS) and Departamento de Química Orgánica, 16780 Universidade de Santiago de Compostela, 15782, Santiago de Compostela, Spain

## Abstract

1,2,3,4-Tetrahydroquinolines (THQs) are essential structural cores in many natural products and pharmaceutical drugs. Especially relevant are those presenting substitutions at position 2, yet practical methods for their one-step assembly from acyclic precursors are very scarce. Herein, we present a straightforward approach to assembling these skeletons from *ortho*-methylanilines using a palladium-catalyzed C­(sp^3^)–H activation/formal cycloaddition sequence. Key for the success of the approach is the use of dienes as partners, since they lead to stable π–allyl palladium intermediates that prevent β-hydride elimination processes and allow installation of versatile alkenyl handles at position 2. Moreover, installing a perfluorobenzenesulfonyl substituent at the amine not only facilitates the C–H activation but also allows for an easy recovery of the free amine.

## Graphical abstract

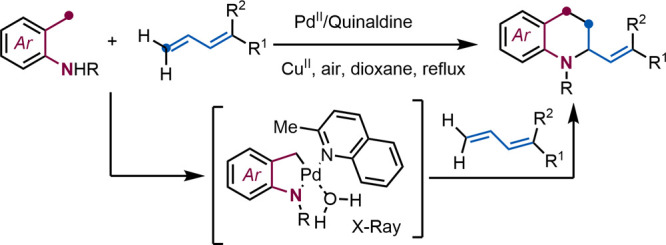

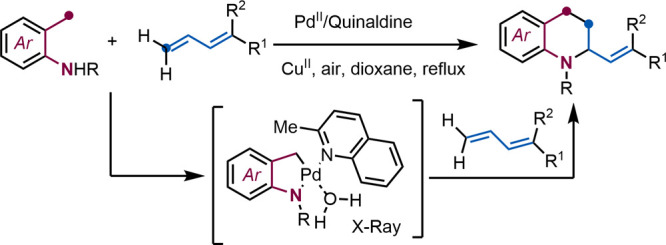

1,2,3,4-Tetrahydroquinolines are privileged heterocycles that form the structural core of many types of natural products and drugs.^1^ For instance, they are an integral part of drugs like oxamniquine (marketed under the name Vansil),^2^ used for schistosomiasis, nadifloxacin, that exhibits antibacterial properties and is employed in the treatment of acne,^3^ or torcetropib, a known therapy for hypercholesterolemia,^4^ among others (see Figure 1). Most of these compounds present a substitution in position 2, and therefore, the development of synthetic routes that allow an expedient assembly of this type of substituted tetrahydroquinole represents a significant objective in organic synthesis and medicinal chemistry.

**Figure 1:**
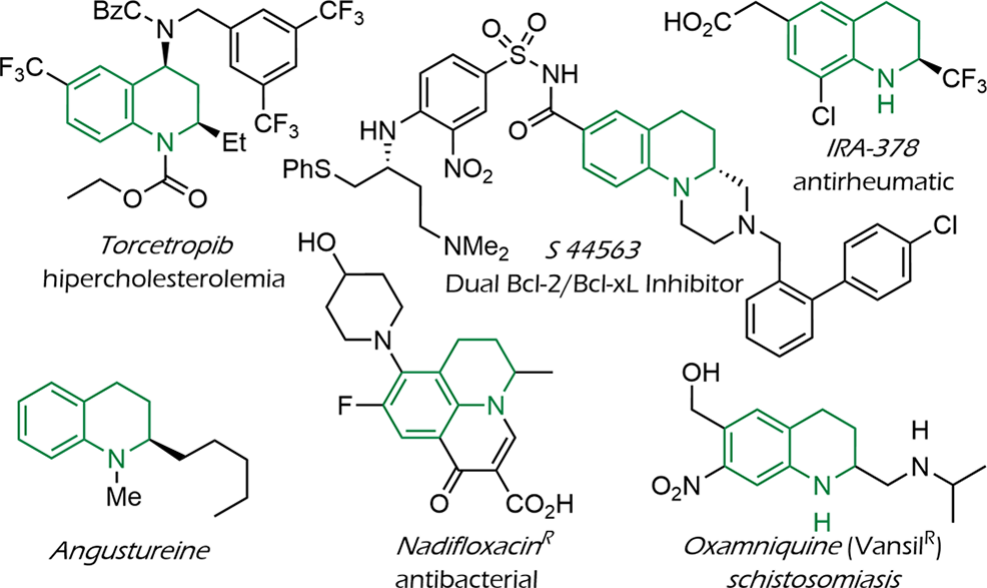
Pharmaceutical drugs and natural products with 2-substituted THQ cores.

Over the past few years, metal-promoted C–H functionalization reactions have become powerful and versatile tools for transforming readily available precursors into more complex and functionally relevant products.^5^ When the C–H activation is combined with an annulation process, the reaction can be used for the synthesis of carbo- or heterocyclic products in a very straightforward manner.^6,7^

We recently reported a Pd-promoted C–H activation/annulation methodology that allows building tetrahydroquinolines (THQs) from triflyl *ortho*-methylanilides and allenes (Scheme 1A).^8^ Whereas the reaction allows the assembly of a THQ skeleton from simple precursors, it is not valid for the preparation of 2-monosubstituted products, which are more relevant from a biomedicinal perspective. Moreover, the annulation protocol using allene partners presented some limitations, such as the need to use an excess of the amide because of partial degradation under the reaction conditions and the difficulties for removing the amine triflyl substituent.

**Scheme 1:**
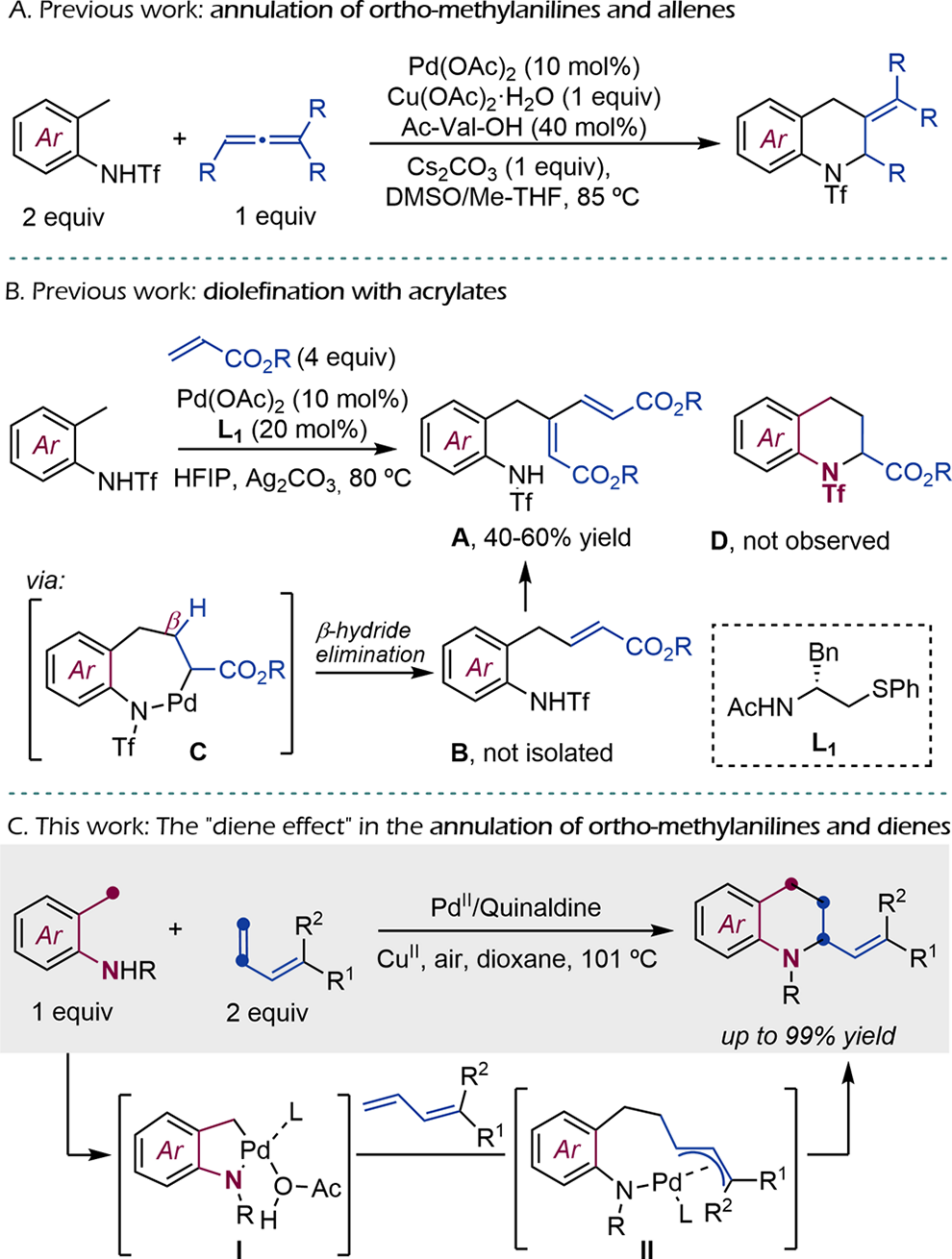
Previous and Present Work

Preliminary assays using alkenes (styrene or methyl acrylate) instead of allenes as reaction partners failed to give the desired THQs. Along these lines, Ji’s group has recently reported that triflyl *ortho*-methylanilides can react with acrylates but give addition rather that cycloaddition products.^9^ Specifically, the reaction yields dienic products like **A** (Scheme 1B), which are proposed to arise from the formal addition product **B**, itself formed through a β-hydride elimination in palladacyclic intermediates like **C**. Cyclic tetrahydroquinolines of type **D** were not observed.

Considering these results, we questioned whether the use of dienes as reaction partners could allow us to obtain 2-substituted THQs, because in this case the β-hydride elimination should be more difficult. Moreover, the “diene” effect might favor the migratory insertion and reductive elimination steps.^10,11^

In this study, we demonstrate the viability of this “diene” approach to build 2-substituted THQs (Scheme 1C). We also show that using a perfluorobenzenesulfonyl group as *N*-substituent instead of the canonical triflyl allows a much easier uncaging of the amine precursor. The reaction mechanism likely involves the formation of a relatively stable π-allyl palladium intermediate (**II**) where the β-hydride elimination path is less accessible (Scheme 1C).

Initial assays were carried out with triflyl precursor **1a** and diene **2a**. Heating a 1:2 mixture of both compounds at 110 °C in dichloroethane, in the presence of 20 mol % of quinaldine as palladium ligand,^9,12^ we observed the desired tetrahydroquinoline product, but only in 16% yield (entry 1, Table 1). Using other solvents like toluene, methyl-THF, or chlorotoluene, we observed similar results. However, in dioxane, we obtained the cyclic product in 37% isolated yield (entry 5).

**Table 1:**
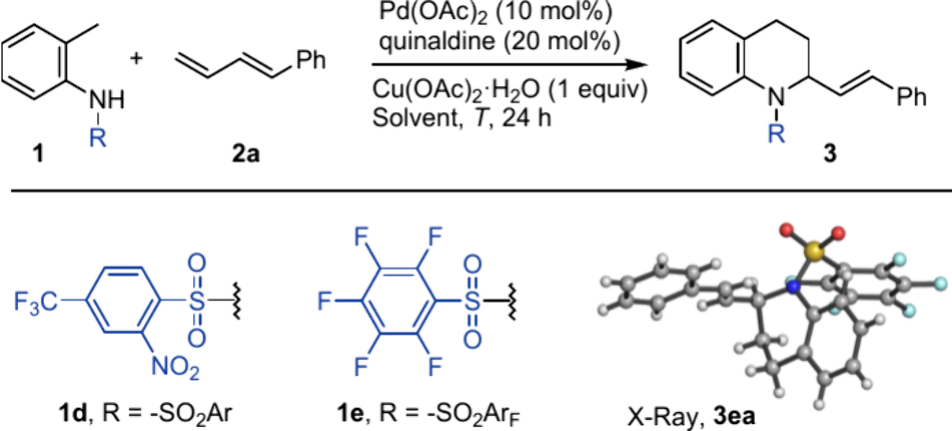
Optimization of Reaction Conditions^a^

Entry	R	Solvent	Temp	Yield (**3**)
1	Tf (**1a**)	Dichloroethane	110 °C	16%
2	Tf	Chlorotoluene	140 °C	20%
3	Tf	Toluene	110 °C	26%
4	Tf	Me-THF	110 °C	23%
5	Tf	Dioxane	110 °C	37%
6	Tf	Dioxane	110 °C	71%^b^
7	H (**1b**)	Dioxane	110 °C	0%^b^
8	Ns (**1c**)	dioxane	110 °C	0%^b^
9	SO_2_Ar (**1d**)	Dioxane	101 °C	<5%^b^
10	SO_2_Ar_F_ (**1e**)	Dioxane	101 °C	83%^b^
11	SO_2_Ar_F_ (**1e**)	Dioxane	101 °C	93%^b^ ^,^ ^c^
12	SO_2_Ar_F_ (**1e**)	Dioxane	101 °C	0%^b^ ^,^ ^c^ ^,^ ^d^
13	SO_2_Ar_F_ (**1e**)	Dioxane	101 °C	<5%^c^ ^,^ ^e^
14	SO_2_Ar_F_ (**1e**)	Dioxane	101 °C	78%^b^ ^,^ ^c^ ^,^ ^f^

a Conditions found in the table: 0.1 mmol of **1**, 0.2 mmol of diene **2**, 10 mol % Pd­(OAc)_2_, 20 mol % of quinaldine, 0.1 mmol of Cu­(OAc)_2_·H_2_O, 0.1 M.

b 0.2 mmol of Cu­(OAc)_2_·H_2_O, 0.05 M.

c 1 equiv of AcOH.

d Without quinaldine.

e Without Cu­(OAc)_2_·H_2_O.

f Under Ar.

Further optimization of the reaction conditions, such as the use of a higher dilution and 2 equiv of the copper salt, allowed us to increase the yield up to 71% (entry 6). Not surprisingly, the parent amine **1b** gave a poor conversion, with no traces of the cycloadduct (entry 7), whereas using a nosyl derivative (**1c**) we only observed products arising from the activation of the nosyl ring (entry 8). Assuming that the reaction requires a strong electron-withdrawing group at the amine and considering the requirement of its removal after the reaction, we tested an anilide derivative with a *p*-CF_3_-*o*-Ns group (**1d**); however, we only observed traces of the product (entry 9). To our delight, using the perfluorinated benzenesulfonamide **1e** we obtained an excellent 83% yield of the desired cycloadduct, a yield that was increased to 93% if 1 equiv of acetic acid is added to the reaction mixture (entries 9, 10). This reaction was repeated at 1 mmol scale to obtain an even better 95% yield. It is worth noting that the reaction is very efficient even using the anilide as a limiting reagent. This contrasts with our previous reaction with allenes, which required an excess of the triflyl-anilide partner and led to poorer yields due to its partial degradation under the harsher reaction conditions.^8^ The structure of product **3ea** was definitively confirmed by X-ray diffraction (Scheme 2). The quinaldine additive is key for the success of the reaction because when the reaction was performed in the absence of this ligand, only traces of the cycloadduct were isolated (entry 12). Other related pyridine ligands also enable the reaction, but they are not as effective as quinaldine (see the Supporting Information).

**Scheme 2:**
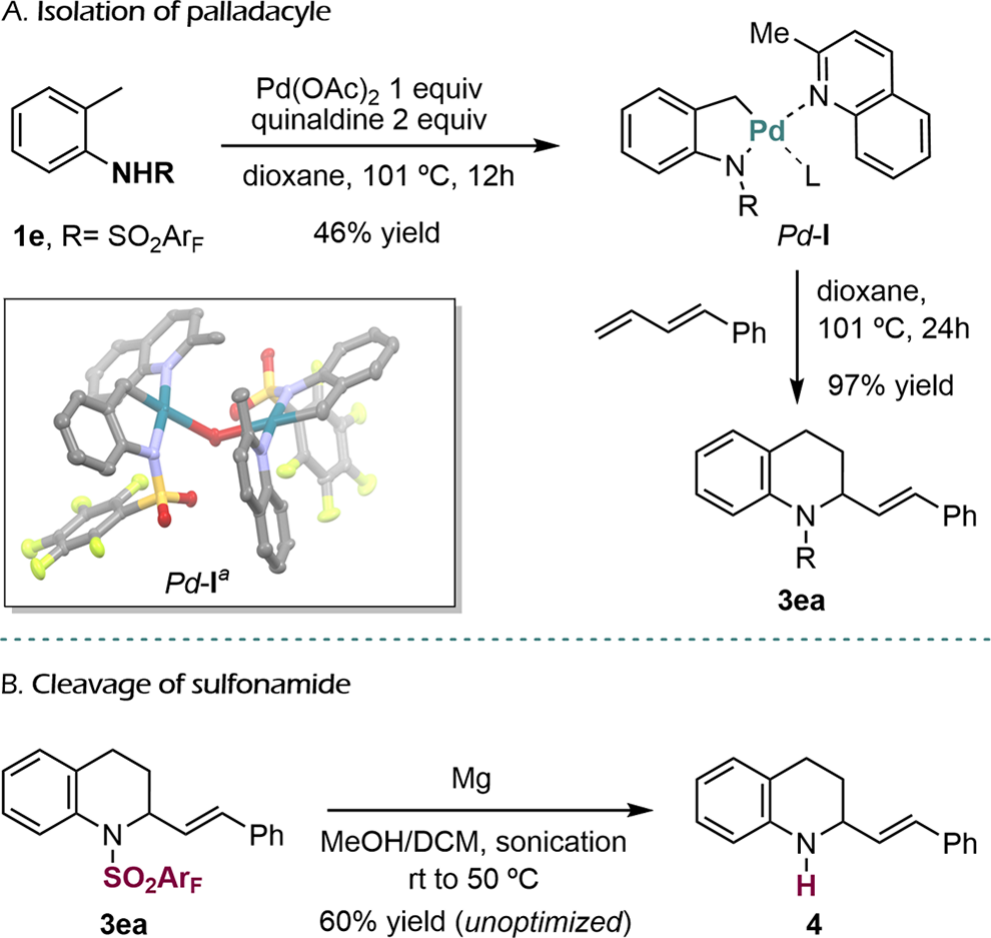
Isolation of the Palladacycle and Deptrotection of the Amine

a For clarity only one of the two conformations found in the crystal structure of *Pd*-**I** is depicted. Hydrogens are omitted.

Mechanistically relevant, we were able to isolate and characterize by X-ray crystallography the palladacycle intermediate *Pd* **-I**, formed after treatment of **1e** with 1 equiv of palladium acetate and 2 equiv of quinaldine (Scheme 2A). Although this complex is dimeric, with a molecule of water as a bridge between both palladium atoms, it very likely becomes monomeric in solution. When *Pd* **-I** was treated with the diene **1a**, we obtained the tetrahydroquinoline **3ea** in 97% yield, confirming that this complex in an intermediate in the reaction.

We next explored the removal of the perfluorobenzenesulfonyl substituent at the amine site in the cycloadduct. We tested various conditions previously described for the cleavage of sulfonamides, including treatments with Red-Al, with Ph_2_P­(H), or with KOH, TBAF, NaI, and TMSCl. While these assays proved unsuccessful, we were glad to observe that using Mg turnings and sonication in a methanol/dichloromethane mixture allows obtaining the amine **4** in a 60% unoptimized yield (Scheme 2B).^13^

Once the feasibility of the cycloaddition and the possibility of removing the nitrogen protecting group were confirmed, we explored the reaction scope. As depicted in Scheme 3, the annulation reaction was also effective with substrates featuring chloride, nitrile, or methoxy groups in *para* position to the aromatic sulfonamide, obtaining the corresponding products **(3fa**–**3ha**) in yields between 55% and 99%. Sulfonamides featuring substituents at the *meta* position, either electron-donating or electron-withdrawing, were also good substrates, and products **3ia**–**3ma** were obtained with yields varying from 50% for the *para* chloride (**3ka**) to 83% for the fluoride derivative (**3ja**).

**Scheme 3:**
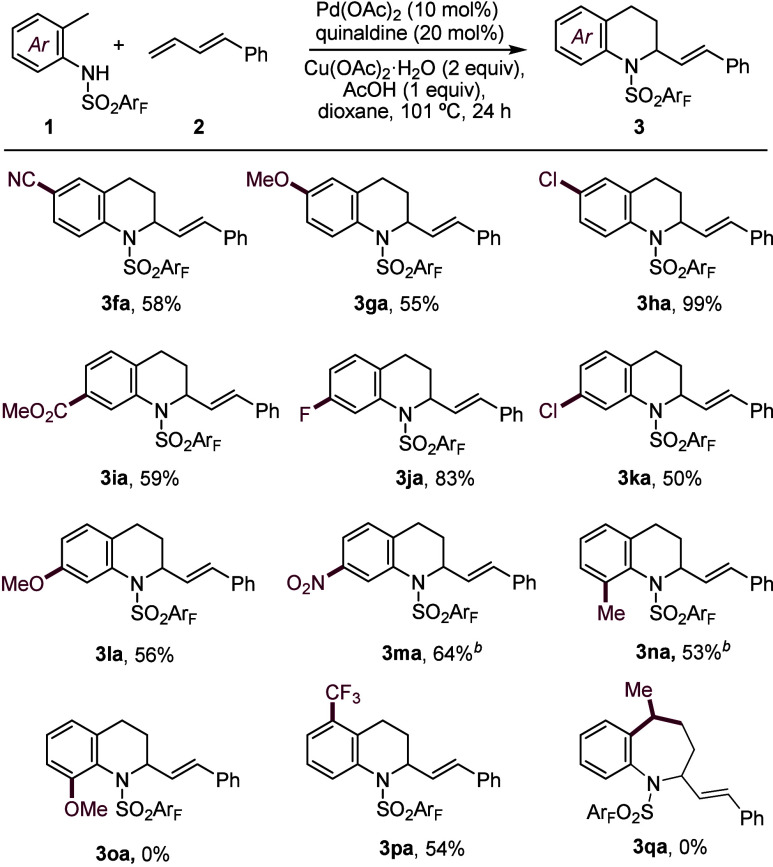
Scope of *ortho*-Methyl Sulfonamides^a^

a Standard contions: 0.1 mmol of **1**, 0.2 mmol of **2**, 10 mol % of Pd­(OAc)_2_, 20 mol % of quinaldine, 0.2 mmol of Cu­(OAc)_2_·H_2_O, 0.1 mmol of AcOH, 101 °C, 0.05 M.
b Another 5 mol % of Pd­(OAc)_2_, 10 mol % of quinaldine, and 1 equiv of copper were added after 24 h.

The presence of a methyl group *ortho* to the sulfonamide group leads to a slower reaction, but still the product **3na** was formed in 53% yield. Surprisingly, no reaction was detected with a substrate with a methoxy instead of a methyl group at this position, probably because of a deactivating coordination to an aminopalladium intermediate. The presence of groups *ortho* to the methyl substituent was also tolerated, as demonstrated for a trifluoromethyl derivative, which gave the product **3pa** in 54% yield. We also tested an *ortho*-isopropylanilide, but the expected adduct **3qa** was not observed.

We then analyzed the scope with respect to the diene component (Scheme 4). We found that the reaction is general for other dienes bearing different aromatic substituents at the terminal position. In general, better results were obtained with *para*-substituents such as nitro (**3eb**, 82% yield), fluoro (**3ec**, 40% yield), trifluoromethyl (**3ed**, 76% yield), or methoxy (**3ee**, 70%) than with the *ortho*-substituted methoxy tested (**3ef**, 53% yield). The disubstituted diene **2g** also worked, leading to the product **3eg** in 52% yield, while disubstituted diene **2h** did not give adduct **3eh**, which indicates that a terminal diene is required for good reactivity. Finally, other dienes with ester or alkyl chains also worked in moderate yields (**3ei** and **3ej**, 52–63% yield).

**Scheme 4:**
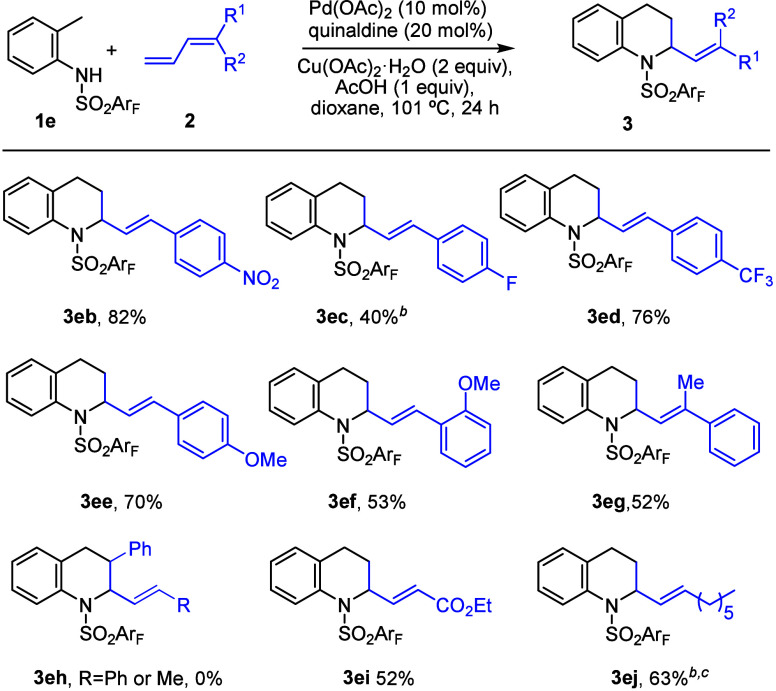
Scope of Dienes

a Standard conditions from Table 1.
b Another 5 mol % of Pd­(OAc)_2_, 10 mol % of quinaldine and 1 equiv of copper were added after 24 h.
c Five equivalents of diene was used.

Not surprisingly, when the reaction was tested with styrene, we did not detect the tetrahydroquinoline product **5**, but a mixture of products, from which we could isolate the indoline **6**, albeit in very low yield. This is an interesting adduct that incorporates two units of styrene (Scheme 5A). Very likely, this product is formed in a cascade reaction involving the C–H activation to give the palladacyle, styrene migratory insertion, and β-elimination. Then a reinsertion of the Pd-hydride occurs, followed by a second styrene insertion and a final β-elimination. The formation of this side product highlights the need of the “diene effect” for the desired reactivity toward tetrahydroquinolines.

**Scheme 5:**
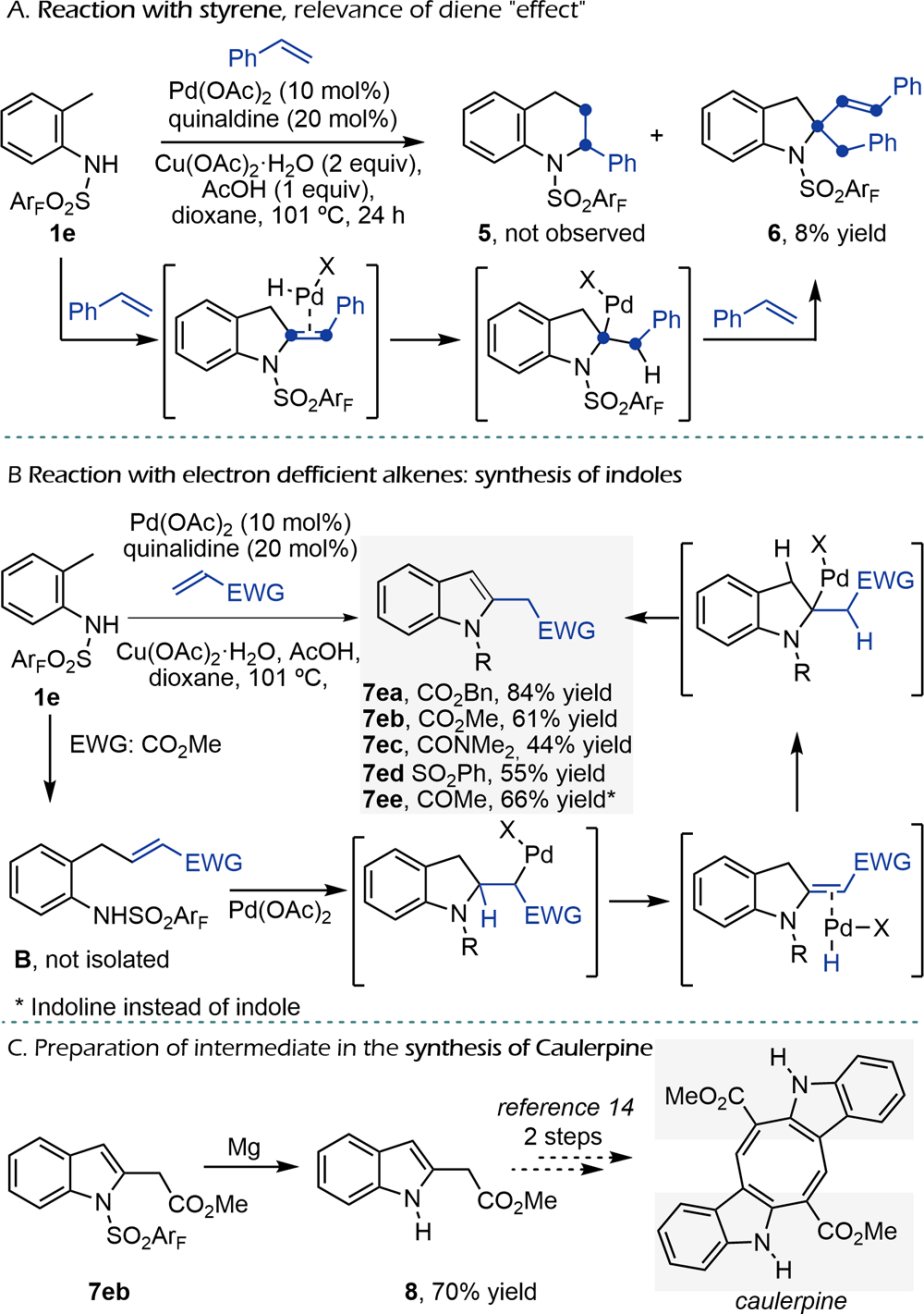
Reactivity of Alkenes

In view of this result, we revisited the reaction using acrylate as the reaction partner but under the newly optimized conditions. Interestingly, treatment of the substrate **1e** with benzyl acrylate (2 equiv), under the optimal conditions developed for dienes, provided the indole **7ea** in an excellent 84% yield. A similar product was obtained using methyl instead of benzyl acrylate, although in a slightly lower yield of 61%, likely due to the volatility of the reactant. Notably, acrylamide and vinylsulfone also proved effective, so that the indoles **7ec** and **7ed** were formed in a 44% and 55% yield, respectively, while but-3-en-2-one gave the indoline product **7ee** in 66% yield (Scheme 5B). The formation of these indoles likely involves a C–H activation/olefination sequence to give intermediate **B**, followed by a Wacker-type addition and elimination and a palladium hydride-mediated isomerization to the aromatic compound.

These results again highlight the relevance of the diene partners for the synthesis of the THQ skeletons but also propose a straightforward methodology to obtain interesting indole products. Indeed, removal of the sulfonamide group in compound **7eb** resulted in the formation of product **8**, which can be transformed in only two steps in caulerpine (Scheme 5C), a natural compound derived from algae known for its anti-inflammatory properties and demonstrating protective effects against colon cancer and liver tumors.^14^

In conclusion, we have implemented a straightforward methodology for the synthesis of 2-substituted tetrahydroquinoline products through the activation of the sp^3^ C–H bond of readily available *ortho*-anilines and a concomitant formal (4 + 2) cycloaddition with dienes. The use of a perfluorobenzosulfonyl substituent at the nitrogen not only enhances the reactivity but also facilitates the recovery of the unprotected amine.

## Supplementary Material



## Data Availability

The data underlying this study are available in the published article and its Supporting Information.

## Notes

Experimental details and characterization data for all new compounds (PDF)
