# Supplementary material for: Assembly of 2‑Substituted Tetrahydroquinolines from ortho-Methylbenzenesulfamides and Dienes, Using a C(sp3)–H Activation/Annulation Sequence
Source: Org Lett. 2024 Sep 11;26(37):7789–94. doi: 10.1021/acs.orglett.4c02292 (PMC12442084; doi:10.1021/acs.orglett.4c02292)

Supporting Information

Assembly of 2-substituted tetrahydroquinolines from *ortho*-methylbenzenesulfamides and dienes, using a C(sp^3)-H activation/ annulation sequence

Iván Huertas-Morales, Borja Cendon, Domingo Costa, José Luis Mascareñas*, Moisés Gulías*

e-mail: joseluis.mascarenas@usc.es, moises.gulias@usc.es

Centro Singular de Investigación en Química Biológica y Materiales Moleculares (CiQUS) and Departamento de Química Orgánica, Universidad de Santiago de Compostela, 15782 Santiago de Compostela, Spain.

Table of contents

1. **General experimental information** 1
2. **Synthesis of Starting Materials and dienes** 2
   2.1 Synthesis of 1,1,1-trifluoro-*N*-(o-tolyl)methanesulfonamide 1a 2
   2.2 Synthesis of sulfonamides 2
   2.3 Synthesis of dienes and acrylates 9
3. **Optimization of the reaction conditions** 10
   3.1 Temperature and solvent optimization 10
   3.2 Copper, Ligand and Solvent concentrations screening 11
   3.3 Protecting group screening 12
   3.4 Ligand screening 13
   3.5 Reaction scope 14
   3.6 Pd-*I* isolation and reaction with diene 28
   3.7 Deprotection of sulfonamides 3ea and 7eb 29
   3.8 1 mmol scale 31
4. **Crystallographic Data** 32
5. **References** 36
6. **NMR Spectra** 37

1. General experimental information

Dry solvents were obtained from Across Organics, Extra Dry over Molecular Sieves, and used without further purification. $Pd(OAc)_2$ (98%) [3375-31-1] was obtained from Strem. All other chemicals were purchased from Sigma Aldrich, Acros Organics, Alfa Aesar, Fluorochem, TCI Chemical, Fluka or BLD Pharm, and were used as received; unless Et_3N that was distilled over CaH_2. All palladium-catalyzed C-H cycloadditions were carried without precautions to elude moisture or oxygen. The abbreviation "rt" refers to a temperature between 20-25 ºC. Reaction mixtures were stirred using Teflon-coated magnetic stir bars. Thin layer chromatography (TLC) was carried out on pre-coated silica gel F_{254} plates with visualization under UV light or by dipping the plate into *p*-anisaldehyde or ceric ammonium molybdate solutions followed by heating. Column chromatography was performed on silica gel (40-60 μm) unless otherwise stated. NMR data was collected on Varian Mercury 300 MHz or Bruker AVIII 500 MHz spectrometers. Chemical shifts are given in ppm (δ) and are referenced to the residual solvent $CHCl_3$. NMR data was analyzed using MestReNova NMR data processing software (http://mestrelab.com/). High Resolution Mass Spectra (HRMS) were performed at the CACTUS facility of the University of Santiago de Compostela on a Bruker micrOTOF spectrometer.

X-ray crystallographic analysis of compound **3ea** and ***Pd*-I** was performed at the CACTUS facility of the University of Santiago de Compostela.

2. Synthesis of Starting Materials and dienes

2.1 Synthesis of 1,1,1-trifluoro-*N*-(o-tolyl)methanesulfonamide 1a

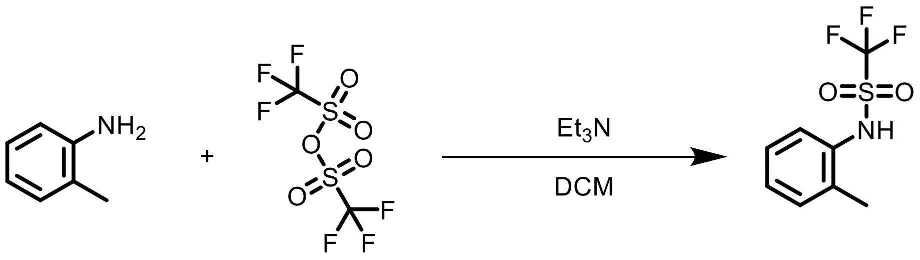

To a solution of *o*-toludine (536 mg, 5.0 mmol, 2 equiv) in dichloromethane (5 mL) at -78 °C under nitrogen was added triethylamine (0.68 mL, 5.0 mmol, 2.0 equiv). After the solution was stirred 5 minutes at that temperature trifluoromethanesulfonic anhydride (0.42 mL, 2.5 mmol, 1.0 equiv) was added dropwise to the mixture. The reaction was stirred 1 hour at -78 °C before being quenched by ice water (10 mL). The organic layer was separated and the aqueous layer extracted with dichloromethane (5 mL x 2). The combined organic phase was washed with brine (10 mL) and then the combined organic phase was dried over anhydrous Na_2SO_4. Evaporation of the solvents and purification of the product by column chromatography on silica gel (ethyl acetate/hexane = 1:100-1:5 as eluent) afforded product **1a** as a white solid (448 mg, 75% yield**).** Spectral data recorded was in agreement with the previously reported[1]

2.2 Synthesis of sulfonamides

General procedure: preparation of sulfonamides substrates 1c to 1q

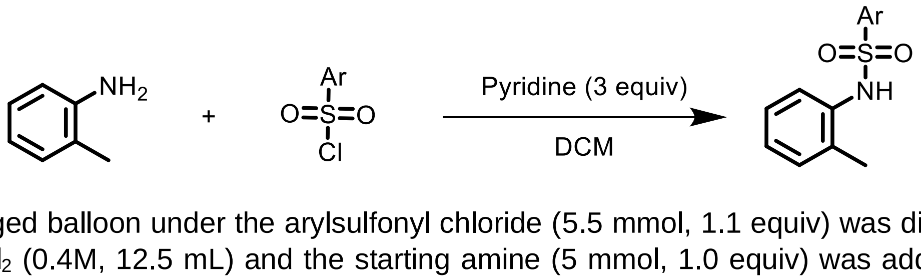

In a purged balloon under the arylsulfonyl chloride (5.5 mmol, 1.1 equiv) was dissolved in CH_2Cl_2 (0.4M, 12.5 mL) and the starting amine (5 mmol, 1.0 equiv) was added and pyridine (1.2 mL, 3.0 equiv) were added and the mixtue was stirred for about 2h at rt. When the reaction was completed HCl (10%) is added until pH close to 2 and the aqueous phase was extracted three times with DCM. The assembled organic phases are washed with brine and dried with anhydrous sodium sulphate, concentrated and purified by column chromatography if necessary.

4-nitro-*N*-(*o*-tolyl)benzenesulfonamide

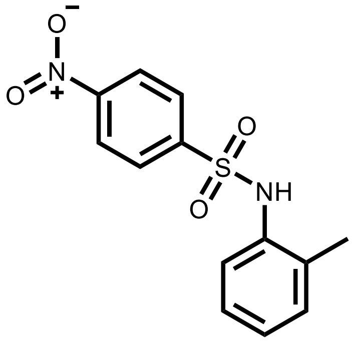

1c was obtained without further purification as a pink solid (1.45 g, 99% yield)

^{1}H NMR (500 MHz, $CDCl_3$) δ 8.28 (m, 2H), 7.90 (m, 2H), 7.27 (m, 1H), 7.15 (m, 3H), 6.50 (s, 1H), 2.02 (s, 3H).

^{13}C NMR (126 MHz, $CDCl_3$) δ 150.4 (C), 145.5 (C), 133.5 (C), 132.2 (C), 131.3 (CH), 128.6 (CH), 127.46 (CH), 127.42 (CH), 125.2 (CH), 124.4 (CH), 17.8 (CH_3).

HRMS (APCI+) *m/z* calcd. for $C_{13}H_{13}N_2O_4S$ $[M+H]^+$: 293.0591; found: 293.0591.

2-nitro-*N*-(*o*-tolyl)-4-(trifluoromethyl)benzenesulfonamide

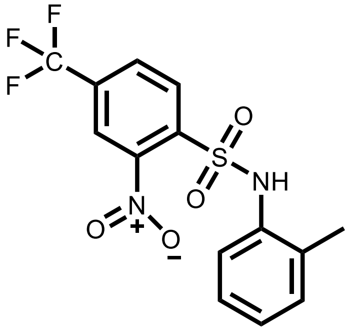

The residue was purified via silica gel flash chromatography (40% to 60% Et_2O/Hexane) to afford **1d** as a pale yellow solid (1.49 g, 83% yield)

^{1}H NMR (300 MHz, $CDCl_3$) δ 8.12 (d, *J* = 1.7 Hz, 1H), 7.99 (d, *J* = 8.2 Hz, 1H), 7.87 (dd, *J* = 8.3, 1.8 Hz, 1H), 7.17 (m, 4H), 2.24 (s, 3H).

^{19}F NMR (282 MHz, $CDCl_3$) δ -63.25.

^{13}C NMR (75 MHz, $CDCl_3$) δ, 148.3 (C), 137.0 (C), 136.0 (q, J = 34.9 Hz, C), 133.8 (C), 133.4 (C), 132.6 (CH), 131.5 (CH), 129.6 (q, J = 3.6 Hz, CH), 127.8 (CH), 127.2 (CH), 125.7 (CH), 122.8 (q, J = 3.7 Hz, CH), 122.1 (q, J = 273.7 Hz, CF), 18.0 (CH_3).

HRMS (APCI+) *m/z* calcd. for $C_{14}H_{12}F_3N_2O_4S$ $[M+H]^+$: 361.0466; found: 361.0464 .

2,3,4,5,6-pentafluoro-*N*-(*o*-tolyl)benzenesulfonamide

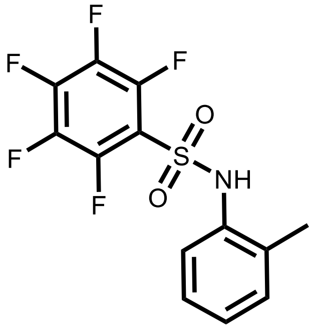

The residue was purified via silica gel flash chromatography (10% to 20% Et_2O/Hexane) to afford **1e** as a white solid (960.5 mg, 57% yield)

^{1}H NMR (500 MHz, $CDCl_3$) δ 7.14 (dd, *J* = 7.5, 1.9 Hz, 1H), 7.07 (dd, *J* = 7.0, 2.3 Hz, 1H), 7.01 (m, 2H), 6.91 (bs, 1H), 2.14 (s, 3H).

^{19}F NMR (471 MHz, $CDCl_3$) δ -135.76 (m), -144.59 (m), -158.21 (m).

^{13}C NMR (126 MHz, $CDCl_3$) δ 144.9 (dm, J = 259.8 Hz, CF), 144.4 (dm, *J* = 262.9 Hz, CF), 138.0 (dm, *J* = 258.4 Hz, CF), 133.1 (C), 131.6 (CH), 131.2 (C), 127.5 (CH), 127.1 (CH), 122.5 (CH), 115.8 (m, C), 17.7 (CH_3).

HRMS (APCI+) *m/z* calcd. for $C_{13}H_9F_5NO_2S$ $[M+H]^+$: 338.0269; found: 338.0269.

N-(4-cyano-2-methylphenyl)-2,3,4,5,6-pentafluorobenzenesulfonamide

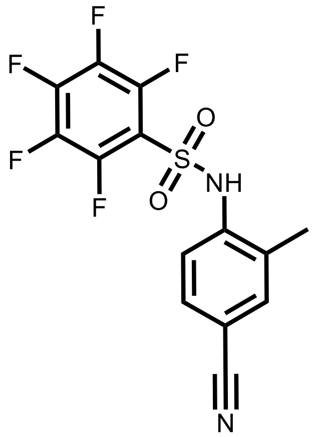

The residue was purified via silica gel flash chromatography (10% to 30% EtOAc/Hexane) to afford **1f** as a pale yellow solid (199.1 mg, 11% yield)

^{1}H NMR (500 MHz, $CDCl_3$) δ 7.54 (d, *J* = 9.0 Hz, 1H), 7.49 (m, 2H), 7.19 (s, 1H), 2.30 (s, 3H).

^{19}F NMR (471 MHz, $CDCl_3$) δ -135.89 (m), -142.67 (tt, *J* = 21.0, 7.5 Hz), -157.12 (m).

^{13}C NMR (126 MHz, $CDCl_3$) 145.0 (dm, *J* = 243.4 Hz, CF), 138.2 (dm, *J* = 260.0 Hz, CF), 137.8 (C), 135.1 (CH), 131.9 (CH), 128.9 (C), 119.4 (CH), 118.1 (C),115.1 (m,C), 109.5 (C), 17.5 (CH_3).

HRMS (APCI+) *m/z* calcd. for $C_{14}H_8F_5N_2O_2S$ $[M+H]^+$: 363.0221; found: 363.0216.

2,3,4,5,6-pentafluoro-N-(4-methoxy-2-methylphenyl)benzenesulfonamide

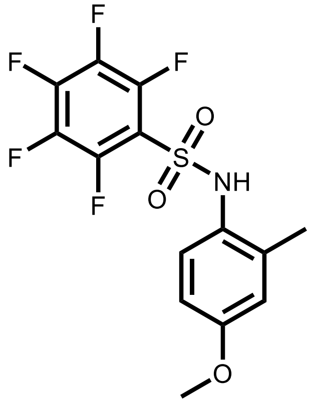

The residue was purified via silica gel flash chromatography (5% to 20% EtOAc/Hexane) to afford **1g** as a brown solid (990 mg, 54% yield)

^{1}H NMR (300 MHz, CDCl3) δ 7.00 (d, *J* = 8.8 Hz, 1H), 6.72 (d, *J* = 2.7 Hz, 1H), 6.63 (dd, *J* = 8.7, 2.9 Hz, 1H), 3.75 (s, 3H), 2.24 (s, 3H).

^{19}F NMR (282 MHz, CDCl3) δ -135.63 (m), -145.18 (tt, *J* = 21.1, 6.8 Hz), -158.51 (m).

^{13}C NMR (75 MHz, CDCl3) δ 159.1 (C), 144.7 (d, J = 259.5 Hz, CF), 144.2 (dm, *J* = 262.4 Hz, CF), 137.7 (dm, *J* = 256.3 Hz, CF), 136.4 (C), 127.0 (C), 125.2 (CH), 116.7 (CH), 116.0 (m, C), 112.2 (CH), 55.4 (OCH3), 18.1 (CH3).

HRMS (APCI+) *m/z* calcd. for $C_{14}H_{11}F_5NO_3S$ $[M+H]^+$: 368.0374; found: 368.0374.

N-(4-chloro-2-methylphenyl)-2,3,4,5,6-pentafluorobenzenesulfonamide

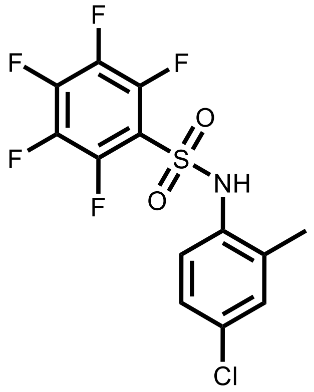

The residue was purified via silica gel flash chromatography (5% to 20% EtOAc/Hexane) to afford **1h** as a white solid (1.17g, 63% yield)

^{1}H NMR (500 MHz, $CDCl_3$) δ 7.22 (d, J = 8.7 Hz, 1H), 7.19 (d, J = 2.8 Hz, 1H), 7.13 (dd, J = 8.7, 2.6 Hz, 1H), 2.25 (s, 3H).

^{19}F NMR (471 MHz, $CDCl_3$) δ -135.67 (dq, *J* = 20.8, 6.9 Hz), -144.01 (tt, *J* = 20.8, 6.9 Hz), -157.85 (tt, *J* = 20.8, 6.5 Hz).

^{13}C NMR (126 MHz, $CDCl_3$) δ 144.9 (dm, *J* = 260.2 Hz, CF), 144.5 (dm, J = 263.8 Hz, CF), 138.0 (dm, *J* = 259.3 Hz, CF), 133.5 (C), 132.7 (C), 131.6 (C), 131.4 (CH), 127.5 (CH), 124.2 (CH), 115.5 (m, C), 17.7 (CH_3).

HRMS (APCI+) *m/z* calcd. for $C_{13}H_7ClF_5NO_2S$ $[M]^+$: 370.9801; found: 370.9794.

Methyl 4-methyl-3-((perfluorophenyl)sulfonamido)benzoate

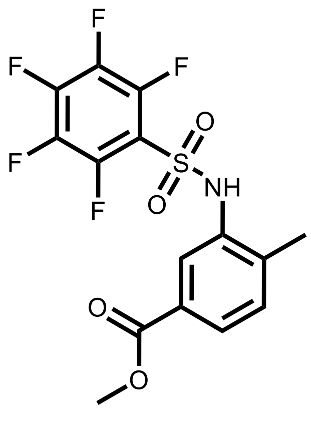

The residue was purified via silica gel flash chromatography (10% to 30% EtOAc/Hexane) to afford **1i** as a white solid (928.3 mg, 47% yield)

^{1}H NMR (500 MHz, $CDCl_3$) δ 7.84 (d, *J* = 1.9 Hz, 1H), 7.80 (dd, *J* = 8.0, 1.8 Hz, 1H), 7.29 (d, *J* = 8.0 Hz, 1H), 3.88 (s, 3H), 2.35 (s, 3H).

^{19}F NMR (471 MHz, $CDCl_3$) δ -135.64 (m), -144.24 (tt, *J* = 20.9, 7.0 Hz), -158.06 (tt, *J* = 21.1, 6.6 Hz).

^{13}C NMR (126 MHz, $CDCl_3$) δ 166.2 (C), 145.0 (dm, *J* = 260.2 Hz, CF), 144.1 (dm, *J* = 263.3 Hz, CF), 137.7 (C), 137.3 (dm, *J* = 258.9 Hz, CF), 133.4 (C), 131.7 (CH), 129.6 (C), 128.3 (CH), 124.2 (CH), 115.6 (m, C), 52.5 (CH_3), 18.2 (CH_3).

HRMS (APCI+) *m/z* calcd. for $C_{15}H_{11}F_5NO_4S$ $[M+H]^+$: 396.0323; found: 396.0325.

2,3,4,5,6-pentafluoro-*N*-(5-fluoro-2-methylphenyl)benzenesulfonamide

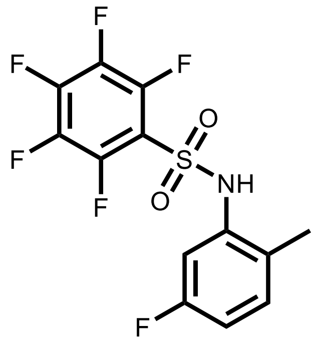

The residue was purified via silica gel flash chromatography (10% to 30% EtOAc/Hexane) to afford **1j** as a pale yellow solid (905.3 mg, 51% yield)

^{1}H NMR (500 MHz, $CDCl_3$) δ 7.15 (d, *J* = 2.2 Hz, 1H), 7.14 (m, 2H), 7.08 (bs, 1H), 6.82 (td, *J* = 8.2, 2.6 Hz, 1H), 2.22 (s, 3H).

^{19}F NMR (471 MHz, $CDCl_3$) δ -113.37, -135.83 (m), -143.79 (tt, *J* = 20.8, 6.9 Hz), -157.76 (m).

^{13}C NMR (126 MHz, $CDCl_3$) δ 161.6 (d, *J* = 245.7 Hz, CF), 145.0 (d, J = 260.2 Hz, CF), 144.6 (d, *J* = 263.8 Hz, CF), 138.1 (d, *J* = 259.3 Hz, CF), 134.2 (d, *J* = 10.4 Hz, C), 132.4 (d, *J* = 9.1 Hz, CH), 124.9 (d, *J* = 3.6 Hz, C), 115.4 (m, C), 113.4 (d, *J* = 20.9 Hz, CH), 108.6 (d, *J* = 26.3 Hz, CH), 16.7 (CH_3).

HRMS (APCI+) *m/z* calcd. for $C_{13}H_8F_6NO_2S$ $[M+H]^+$: 356.0174; found: 356.0169.

***N*-(5-chloro-2-methylphenyl)-2,3,4,5,6-pentafluorobenzenesulfonamide**

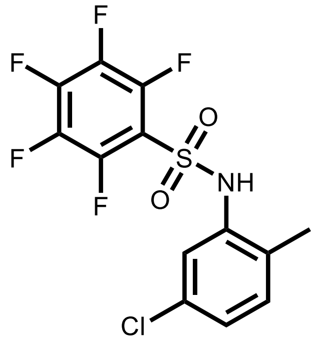

The residue was purified via silica gel flash chromatography (10% to 30% EtOAc/Hexane) to afford **1k** as a pale yellow solid (1.29 g, 70% yield)

^{1}H NMR (500 MHz, $CDCl_3$) δ 7.35 (d, *J* = 2.0 Hz, 1H), 7.12 (m, 2H), 2.23 (s, 3H).

^{19}F NMR (471 MHz, $CDCl_3$) δ -135.73 (dq, *J* = 20.8, 6.9 Hz), -143.82 (m), -157.76 (td, *J* = 20.4, 4.8 Hz).

^{13}C NMR (126 MHz, $CDCl_3$) δ 144.9 (dm, J = 259.8 Hz, CF), 144.6 (dm, *J* = 263.4 Hz, CF), 138.1 (dm, *J* = 259.8 Hz, CF), 134.2 (C), 132.9 (C), 132.4 (CH), 128.8 (C), 126.9 (CH), 122.0 (CH), 115.4 (m, C), 17.3 (CH_3).

HRMS (APCI+) *m/z* calcd. for $C_{13}H_8ClF_5NO_2S$ $[M+H]^+$: 371.9879; found: 371.9880.

2,3,4,5,6-pentafluoro-*N*-(5-methoxy-2-methylphenyl)benzenesulfonamide

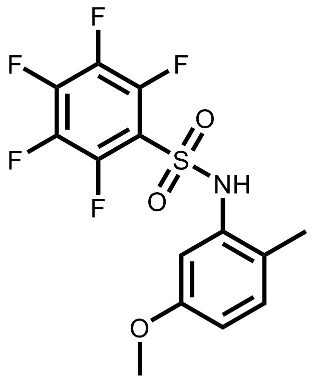

The residue was purified via silica gel flash chromatography (10% to 30% EtOAc/Hexane) to afford **1l** as a white solid (1.15 g, 63% yield)

^{1}H NMR (500 MHz, $CDCl_3$) δ 7.25 (bs, 1H), 7.05 (d, *J* = 8.4 Hz, 1H), 6.95 (d, *J* = 2.6 Hz, 1H), 6.64 (dd, *J* = 8.5, 2.6 Hz, 1H), 3.74 (s, 3H), 2.17 (s, 3H).

^{19}F NMR (471 MHz, $CDCl_3$) δ -135.83 (dq, *J* = 19.9, 6.5 Hz), -144.66 (tt, *J* = 20.8, 6.9 Hz), -158.42 (tt, *J* = 20.8, 6.1 Hz).

^{13}C NMR (126 MHz, $CDCl_3$) δ 158.7 (C), 144.9 (dm, *J* = 259.8 Hz, CF), 144.4 (dm, *J* = 262.9 Hz, CF), 138.0 (dm, *J* = 258.4 Hz, CF), 133.8 (C), 132.0 (CH), 121.8 (C), 115.5 (m, C), 112.3 (CH), 107.4 (CH), 55.4 (CH_3), 16.6 (CH_3).

HRMS (APCI+) *m/z* calcd. for $C_{14}H_{11}F_5NO_3S$ $[M+H]^+$: 368.0374; found: 368.0376.

2,3,4,5,6-pentafluoro-*N*-(2-methyl-5-nitrophenyl)benzenesulfonamide

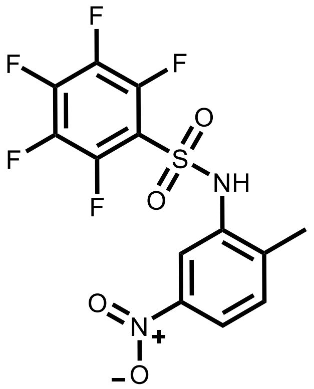

The residue was purified via silica gel flash chromatography (10% to 30% EtOAc/Hexane) to afford **1m** as a pale yellow solid (649.4 mg, 34% yield)

^{1}H NMR (500 MHz, $CDCl_3$) δ 8.19 (d, *J* = 2.3 Hz, 1H), 8.00 (dd, *J* = 8.4, 2.3 Hz, 1H), 7.40 (d, *J* = 8.4 Hz, 1H), 7.24 (s, 1H), 2.41 (s, 3H).

^{19}F NMR (471 MHz, $CDCl_3$) δ -135.65 (m), -142.95 (tt, *J* = 21.3, 7.3 Hz), -157.22 (m).

^{13}C NMR (126 MHz, $CDCl_3$) δ 147.2 (C), 145.0 (dm, J = 260.4 Hz, CF), 144.9 (dm, J = 260.2 Hz, CF), 138.18 (dm, J = 260.2 Hz, CF), 138.10 (C), 134.2 (C), 132.3 (CH), 121.5 (CH), 116.9 (CH), 115.3 (m, C), 18.2 (CH_3).

HRMS (APCI+) m/z calcd. for $C_{13}H_8F_5N_2O_4S$ $[M+H]^+$: 383.0119; found: 383.0121.

***N*-(2,6-dimethylphenyl)-2,3,4,5,6-pentafluorobenzenesulfonamide**

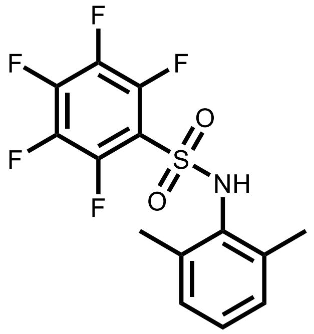

The residue was purified via silica gel flash chromatography (10% to 30% EtOAc/Hexane) to afford **1n** as a white solid (1.44 g, 82% yield)

^{1}H NMR (500 MHz, $CDCl_3$) δ 7.16 (dd, *J* = 8.3, 6.8 Hz, 1H), 7.09 (d, *J* = 7.5 Hz, 2H), 6.53 (s, 1H), 2.21 (s, 6H).

^{19}F NMR (471 MHz, $CDCl_3$) δ -135.83 (m), -145.62 (tt, *J* = 20.9, 6.2 Hz), -158.32 (m).

^{13}C NMR (126 MHz, $CDCl_3$) δ 144.8 (dm, J = 259.3, CF), 144.3 (dm, J = 262.5 Hz, CF), 138.1 (dm, J = 258.4 Hz, CF), 137.8 (C), 131.6 (C), 129.18 (CH), 129.11 (CH), 118.1 (m, C), 19.0 (CH_3).

HRMS (APCI+) *m/z* calcd. for $C_{14}H_{11}F_5NO_2S$ $[M+H]^+$: 352.0425; found: 352.0425.

2,3,4,5,6-pentafluoro-*N*-(2-methoxy-6-methylphenyl)benzenesulfonamide

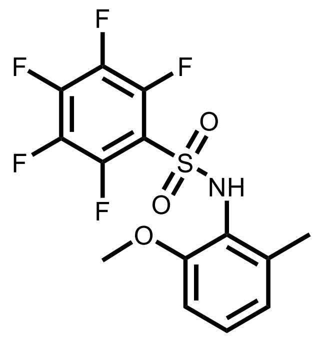

The residue was purified via silica gel flash chromatography (5% to 20% EtOAc/Hexane) to afford **1o** as a white solid (623.9 mg, 34% yield)

^{1}H NMR (500 MHz, $CDCl_3$) δ 7.16 (t, *J* = 8.0 Hz, 1H), 6.89 (d, *J* = 7.6 Hz, 1H), 6.75 (s, 1H), 6.60 (d, *J* = 8.3 Hz, 1H), 3.48 (s, 3H), 2.48 (s, 3H).

^{19}F NMR (471 MHz, $CDCl_3$) δ -135.23 (m), -146.62 (tt, *J* = 20.8, 6.5 Hz), -160.24 (m).

^{13}C NMR (126 MHz, $CDCl_3$) δ 154.6 (C), 145.1 (dm, J = 259.8 Hz, CF), 143.7 (dm, J = 261.6 Hz, CF), 140.3 (C), 137.5 (dm, J = 256.7 Hz, CF), 129.4 (CH), 124.0 (CH), 121.2 (C), 116.9 (m, C), 108.2 (CH), 55.2 (CH_3), 18.9 (CH_3).

HRMS (APCI+) *m/z* calcd. for $C_{14}H_{11}F_5NO_3S$ $[M+H]^+$: 368.0374; found: 368.0374.

2,3,4,5,6-pentafluoro-*N*-(2-methyl-3-(trifluoromethyl)phenyl)benzenesulfonamide

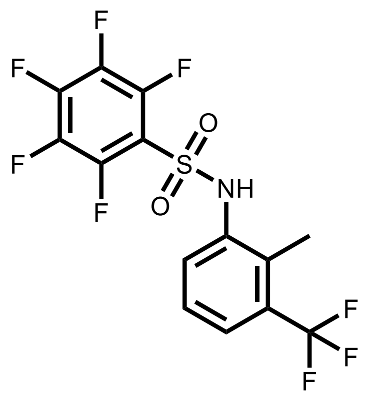

The residue was purified via silica gel flash chromatography (10% to 30% EtOAc/Hexane) to afford **1p** as a pale yellow solid (1.01 g, 50% yield)

^{1}H NMR (500 MHz, $CDCl_3$) δ 7.55 (dd, *J* = 8.0, 1.2 Hz, 1H), 7.49 (d, *J* = 8.1 Hz, 1H), 7.29 (t, *J* = 8.0 Hz, 1H), 7.01 (s, 1H), 2.38 (d, *J* = 1.6 Hz, 3H).

^{19}F NMR (471 MHz, $CDCl_3$) δ -60.87, -135.70 (m), -143.66 (tt, *J* = 21.4, 7.2 Hz), -157.60 (m).

^{13}C NMR (126 MHz, $CDCl_3$) δ 144.9 (dm, *J* = 259.8 Hz), 144.6 (dm, *J* = 263.4 Hz), 138.1 (dm, *J* = 259.3 Hz), 134.7, 131.1 (dm, *J* = 30.0 Hz), 130.7, 127.2, 126.5, 124.9 (q, *J* = 5.8 Hz), 123.9 (q, *J* = 273.8 Hz), 115.6 (m), 13.7 (q, *J* = 2.6 Hz).

HRMS (APCI+) m/z calcd. for $C_{14}H_8F_8NO_2S$ $[M+H]^+$: 406.0143; found: 406.0143.

2,3,4,5,6-pentafluoro-*N*-(2-isopropylphenyl)benzenesulfonamide

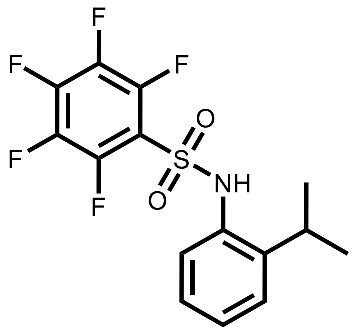

The residue was purified via silica gel flash chromatography (5% to 20% EtOAc/Hexane) to afford **1q** as a yellow solid (456.9 mg, 25% yield)

^{1}H NMR (500 MHz, $CDCl_3$) δ 7.35 (dd, *J* = 7.8, 1.6 Hz, 1H), 7.27 (td, *J* = 7.5, 1.5 Hz, 1H), 7.21 (dd, *J* = 8.1, 1.5 Hz, 1H), 7.15 (ddd, *J* = 8.3, 7.2, 1.6 Hz, 1H), 3.22 (hept, *J* = 6.8 Hz, 1H), 1.21 (d, *J* = 6.8 Hz, 6H).

^{19}F NMR (471 MHz, $CDCl_3$) δ -135.59 (ddt, *J* = 19.9, 13.9, 7.8 Hz), -145.09 (tt, *J* = 20.8, 6.9 Hz), -158.57 (m).

^{13}C NMR (126 MHz, $CDCl_3$) δ 144.8 (dm, *J* = 260.2 Hz, CF), 144.2 (dm, *J* = 262.7 Hz, CF), 143.8 (C), 138.0 (dm, *J* = 258.4 Hz, CF), 131.2 (C), 128.1 (CH), 126.93 (CH), 126.87 (CH), 124.5 (CH), 115.9 (m, C), 27.6 (CH), 23.5 (CH_3).

HRMS (APCI+) *m/z* calcd. for $C_{15}H_{13}F_5NO_2S$ $[M+H]^+$: 366.0582; found: 366.0584.

2.3 Synthesis of dienes and acrylates

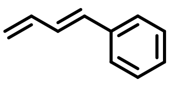

2a

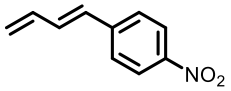

2b

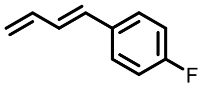

2c

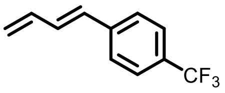

2d

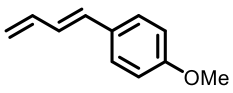

2e

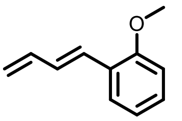

2f

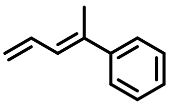

2g

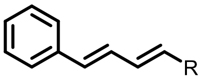

2h, R = Me or Ph

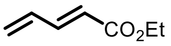

2i

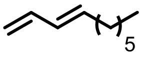

2j

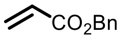

benzyl acrylate

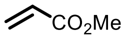

methyl acrylate

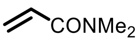

***N*,*N*-dimethylacrylamide**

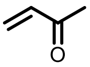

but-3-en-2-one

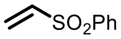

Phenyl vinyl sulfone

Dienes **2a ((*E*)-buta-1,3-dien-1-ylbenzene)** and **2h ((1*E*,3*E*)-1,4-diphenylbuta-1,3-diene)** and **benzyl acrylate**, **methyl acrylate**, ***N*,*N*-dimethylacrylamide, but-3-en-2-one** and **Phenyl vinyl sulfone** were commercially available. **2a** and **2h** (R = Ph) and ***N*,*N*-dimethylacrylamide, but-3-en-2-one** and **ethyl acrylate** were purchased from Aldrich, **Benzyl acrylate** and **Methyl acrylate** from Alfa Aesar, while **Phenyl vinyl sulfone** was purchased from Fluka.

Dienes **2b ((*E*)-1-(1,3-butadienyl)-4-nitrobenzene)**, **2c ((*E*)-1-(1,3-butadienyl)-4-fluorobenzene)**, **2d ((*E*)-1-(buta-1,3-dien-1-yl)-4-(trifluoromethyl)benzene)**, **2e ((*E*)-1-(buta-1,3-dien-1-yl)-4-methoxybenzene)**, **2f ((*E*)-1-(buta-1,3-dien-1-yl)-2-methoxybenzene)**, **2g ((*E*)-penta-2,4-dien-2-ylbenzene)**, **2j (*E*)-hexa-1,3-diene)** and **2h** (R = Me) **((1*E*,3*E*)-penta-1,3-dien-1-yl)benzene** were synthesized from the corresponding aldehyde via Wittig reaction according to the literature[2,3,4]. Diene **2i (ethyl (*E*)-penta-2,4-dienoate)** was also synthesized with a method previously reported in literature[5]. Spectral data recorded was in agreement with the previously reported.

3. Optimization of the reaction conditions

3.1 Temperature and solvent optimization

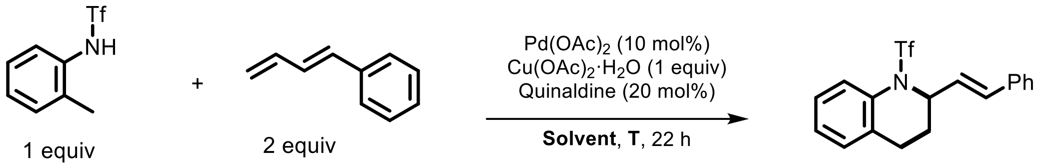

Table S1. Temperature and solvent optimization.

Entry	**T (ºC)**	**Solvent**	Yield (%)
1	110	DCE	16
2	140	PhCl	20
3	110	Toluene	26
**4**	**110**	**Dioxane**	**37**
5	110	Me-THF	23
6	140	Dioxane	57

Reaction conditions: **1a** (0.1 mmol), **2a** (0.2 mmol), $Pd(OAc)_2$ (10 mol%), Quinaldine (20 mol%), $Cu(OAc)_2{\cdot}H_2O$ (1.0 equiv), , **Solvent** (1.0 mL), air. Isolated yields.

3.2 Copper, Ligand and Solvent concentrations screening

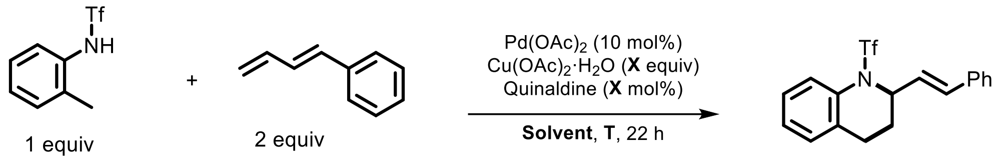

Table S2. Copper, Ligand and Solvent concentrations screening.

Entry	**Equiv Cu**	**Mol% L**	**T (ºC)**	**Dioxane [M]**	Yield (%)
1	1	20	110	0.1	37
2	2	20	110	0.1	53
3	2	10	110	0.1	36
4	2	30	110	0.1	54
5	2	20	110	0.2	29
**6**	**2**	**20**	**110**	**0.05**	**71**
7	2	20	140	0.05	77

Reaction conditions: **1a** (0.1 mmol), **2a** (0.2 mmol), $Pd(OAc)_2$ (10 mol%), Quinaldine (x mol%), $Cu(OAc)_2 \cdot H_2O$ (x equiv), **Solvent** (x mL), air. Isolated yields.

3.3 Protecting group screening

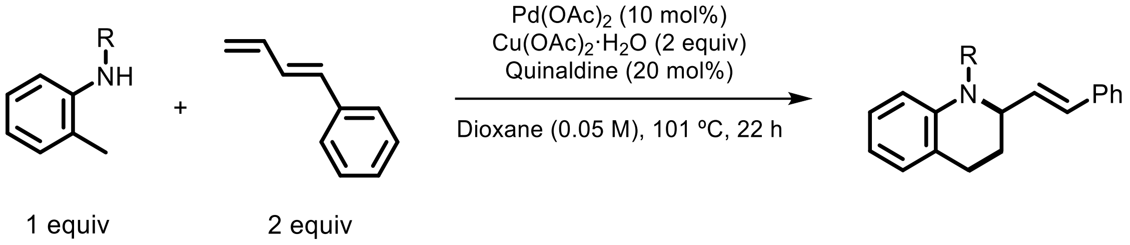

Table S3. Protecting groups screening.

Entry	R	Yield (%)	Comments
1[a]	H	0	-
2[a]	Tf	71	-
3[a]	Ns	-	Ns ring activation (18%) Mixture of products
4	SO_2Ar	Traces	-
5	SO_2Ar_F	83	w/o AcOH
**6**	**SO_2Ar_F**	**93**	**Adding 1 equiv AcOH**
7	SO_2Ar_F	5	w/o $Cu(OAc)_2 \cdot H_2O$
8	SO_2Ar_F	78	Under Argon

Reaction conditions: **1a** (0.1 mmol), **2a** (0.2 mmol), $Pd(OAc)_2$ (10 mol%), Quinaldine (20 mol%), $Cu(OAc)_2 \cdot H_2O$ (2.0 equiv), **Solvent** (2.0 mL), air. Isolated yields. [a] 110 ºC

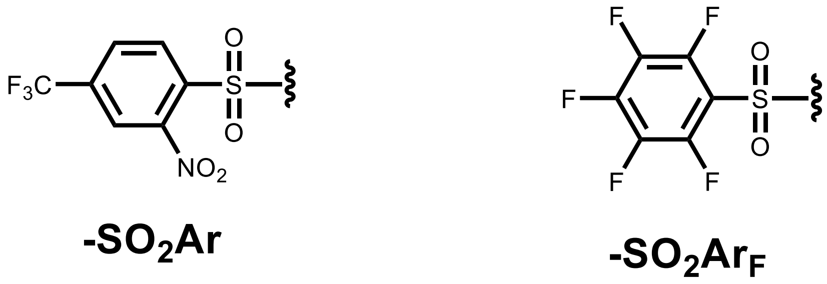

3.4 Ligand screening

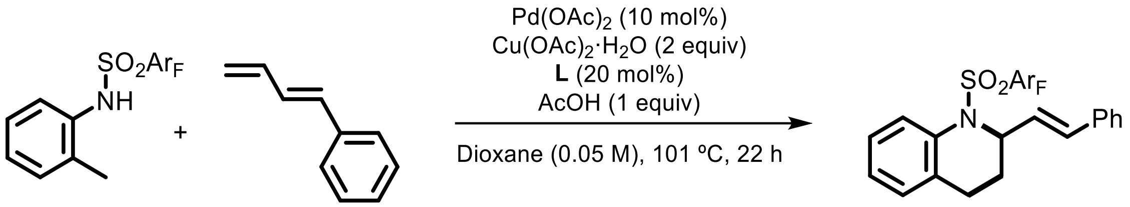

1 equiv 2 equiv

Table S4. Ligand screening.

Entry	L	Yield (%)
1	**Quinaldine**	**93**
2	1-methylisoquinoline	73
3	Pyridine	35
4	2-Picoline	78
5	2,6-Lutidine	80
6	Jin-Quan Yu´s Pyridine-Pyridone Ligand [6]	0

Reaction conditions: **1a** (0.1 mmol), **2a** (0.2 mmol), $Pd(OAc)_2$ (10 mol%), Quinaldine (20 mol%), $Cu(OAc)_2 \cdot H_2O$ (2.0 equiv), **Solvent** (2.0 mL), air. Isolated yields

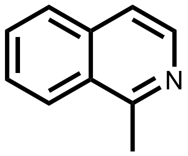

1-methylisoquinoline

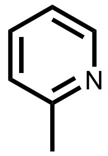

2-Picoline

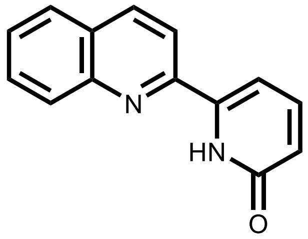

Pyridine-Pyridone ligand

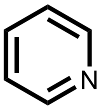

Pyridine

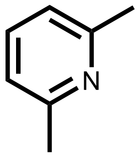

2,6-Lutidine

3.5 Reaction scope

General procedure for the formal C-H activation/annulation between ortho-methylbenzenesulfamides and dienes

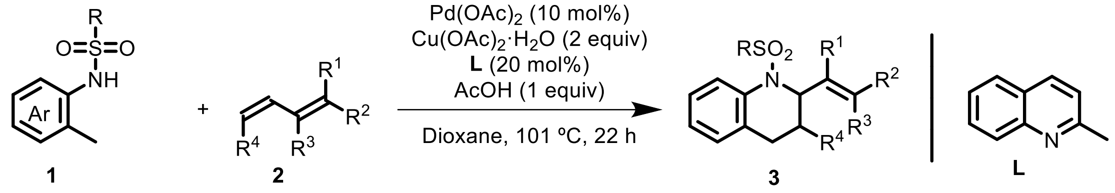

ortho-Methylbenzenesulfonamides **1** (0.10 mmol, 1.0 equiv), $Pd(OAc)_2$ (2.2 mg, 10 mol%), $Cu(OAc)_2 \cdot H_2O$ (39.9 mg, 2.0 equiv) were weighed and added into a Schlenk flask under air. Then, 1,4-dioxane (2.0 mL, 0.05 M), quinaldine (2.8 µL, 20 mol%), AcOH (5.7 µL, 1 equiv) and the corresponding diene **2** (0.20 mmol, 2.0 equiv) were added. The mixture was refluxed at 101 ºC in a Schlenk heating block for 20-24 h using a cold finger setup. After cooling to rt, the reaction mixture was diluted with DCM and filtered through a Fluorisil-silica gel pad, washing the flask and the pad with more DCM (x3). The filtrate was concentrated under reduced pressure and the resulting residue was purified by flash column chromatography to afford the corresponding tetrahydroquinoline products **3**.

(*E*)-2-styryl-1-((trifluoromethyl)sulfonyl)-1,2,3,4-tetrahydroquinoline

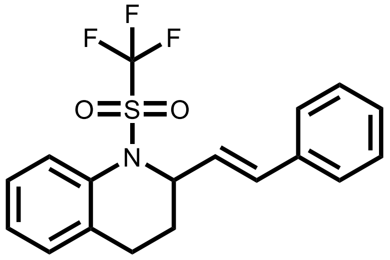

Reaction performed at 110 ºC instead of 101 ºC in a sealed tube.

The residue was purified via silica gel flash chromatography (5% to 15% EtOAc/Hexane) to afford **3aa** as a yellowish solid (26.8 mg, 71% yield).

^{1}H NMR (300 MHz, $CDCl_3$) δ, ppm: 7.62 (d, *J* = 7.8 Hz, 1H), 7.27 (m, 8H), 6.60 (dd, *J* = 15.9, 1.1 Hz, 1H), 6.08 (dd, *J* = 15.9, 6.4 Hz, 1H), 5.20 (q, *J* = 6.0 Hz, 1H), 2.86 (t, *J* = 6.7 Hz, 2H), 2.52 (dq, *J* = 13.6, 6.8 Hz, 1H), 1.98 (dq, *J* = 12.7, 6.3 Hz, 1H).

^{19}F NMR (282 MHz, $CDCl_3$) δ, ppm: -74.69.

^{13}C NMR (75 MHz, $CDCl_3$) δ, ppm: 136.1 (C), 133.9 (C), 133.7 (CH), 132.8 (C), 132.6 (CH), 128.9 (CH), 128.7 (CH), 128.2 (CH), 127.3 (CH), 126.75 (CH), 126.71 (CH), 125.1 (CH), 120.3 (q, *J* = 324.5 Hz, C) 59.8 (CH), 30.1 (CH_2), 24.6 (CH_2).

HRMS (APCI+) *m/z* calcd. for $C_{18}H_{17}F_3NO_2S$ $[M+H]^+$: 368.0927, found 368.0935.

.

(*E*)-1-((perfluorophenyl)sulfonyl)-2-styryl-1,2,3,4-tetrahydroquinoline

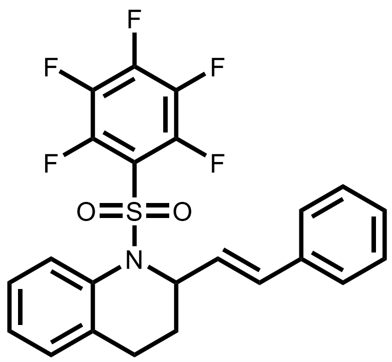

The residue was purified via silica gel flash chromatography (0% to 30% Et_2O/Hexane) to afford **3ea** as a white solid (43.2 mg, 93% yield)

^{1}H NMR (500 MHz, $CDCl_3$) δ 7.80 (d, *J* = 7.8 Hz, 1H), 7.28 (m, 6H), 7.11 (m, 2H), 6.64 (dd, *J* = 15.9, 1.5 Hz, 1H), 6.16 (dd, *J* = 15.9, 5.8 Hz, 1H), 5.37 (qd, *J* = 5.9, 1.6 Hz, 1H), 2.76 (ddd, *J* = 16.2, 8.9, 5.3 Hz, 1H), 2.50 (ddd, *J* = 16.2, 6.9, 5.0 Hz, 1H), 2.24 (dddd, *J* = 13.8, 9.0, 6.0, 5.0 Hz, 1H), 1.93 (dtd, *J* = 13.4, 6.7, 5.5 Hz, 1H).

^{19}F NMR (471 MHz, $CDCl_3$) δ -133.83 (dt, *J* = 21.4, 5.2 Hz), -144.80 (tt, *J* = 21.2, 6.8 Hz), -158.37 (m).

^{13}C NMR (126 MHz, $CDCl_3$) δ 145.0 (dm, J = 259.8 Hz, CF), 144.1 (dm, *J* = 262.5 Hz, CF), 137.9 (dm, *J* = 258.4 Hz, CF), 136.3 (C), 134.1 (C), 132.1 (CH), 131.1 (C), 129.2 (CH), 128.6 (CH), 128.0 (CH), 127.7 (CH), 127.4 (CH), 126.7 (CH), 125.8 (CH), 123.9 (CH), 115.9 (m, C), 58.5 (CH), 28.9 (CH_2), 24.9 (CH_2).

HRMS (APCI+) *m/z* calcd. for $C_{23}H_{17}F_5NO_2S$ $[M+H]^+$: 466.0895; found: 466.0897.

(*E*)-1-((perfluorophenyl)sulfonyl)-2-styryl-1,2,3,4-tetrahydroquinoline-6-carbonitrile

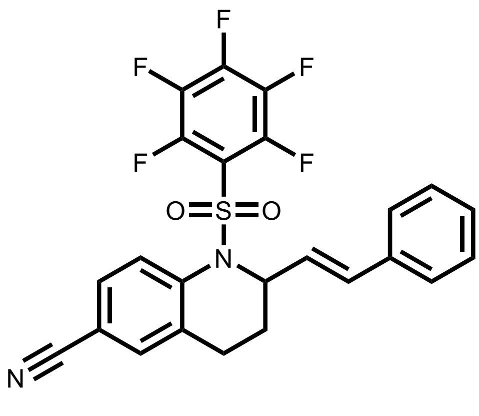

The residue was purified via silica gel flash chromatography (5% to 20% EtOAc/Hexane) to afford **3fa** as a yellow oil (28.4 mg, 58% yield)

^{1}H NMR (300 MHz, $CDCl_3$) δ 7.89 (d, *J* = 8.7 Hz, 1H), 7.48 (dd, *J* = 8.6, 2.0 Hz, 1H), 7.39 (s, 1H), 7.26 (m, 5H), 6.54 (dd, *J* = 15.9, 1.6 Hz, 1H), 6.07 (dd, *J* = 15.9, 5.5 Hz, 1H), 5.40 (q, *J* = 4.5 Hz, 1H), 2.83 (ddd, *J* = 16.7, 10.4, 6.1 Hz, 1H), 2.65 (dt, *J* = 16.7, 5.1 Hz, 1H), 2.08 (dddd, *J* = 19.3, 13.7, 9.1, 5.5 Hz, 2H).

^{19}F NMR (471 MHz, $CDCl_3$) δ -134.27 (m), -143.01 (tt, *J* = 21.4, 7.3 Hz), -157.38 (m).

^{13}C NMR (126 MHz, $CDCl_3$) δ 145.2 (dm, *J* = 265.2 Hz, CF), 144.7 (dm, *J* = 264.3 Hz, CF), 138.6 (C), 138.1 (dm, *J* = 259.8 Hz, CF), 135.8 (C), 133.5 (CH), 133.0 (CH), 131.1 (CH), 130.3 (C), 128.8 (CH), 128.4 (CH), 126.7 (CH), 126.2 (CH), 122.7 (CH), 118.4 (C), 115.3 (m, C), 108.7 (CH), 58.2 (CH), 27.1 (CH_2), 24.2 (CH_2).

HRMS (APCI+) *m/z* calcd. for $C_{24}H_{16}F_5N_2O_2S$ $[M+H]^+$: 491.0847; found: 491.0847.

(*E*)-6-methoxy-1-((perfluorophenyl)sulfonyl)-2-styryl-1,2,3,4-tetrahydroquinoline

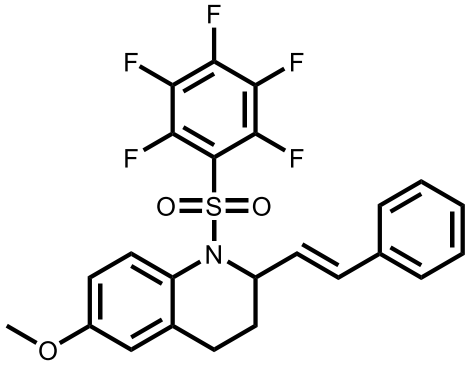

The residue was purified via silica gel flash chromatography (0% to 2% Et_2O/Pentane) to afford **3ga** as a yellow oil (27.2 mg, 55% yield)

^{1}H NMR (500 MHz, $CDCl_3$) δ 7.70 (d, *J* = 9.0 Hz, 1H), 7.32 (m, 2H), 7.28 (dd, *J* = 8.5, 6.5 Hz, 2H), 7.22 (m, 1H), 6.78 (dd, *J* = 9.0, 3.0 Hz, 1H), 6.62 (m, 2H), 6.12 (dd, *J* = 15.9, 5.9 Hz, 1H), 5.27 (q, *J* = 7.1, 6.4 Hz, 1H), 3.78 (s, 3H), 2.66 (ddd, *J* = 15.9, 8.0, 5.2 Hz, 1H), 2.36 (ddd, *J* = 15.9, 7.9, 5.0 Hz, 1H), 2.23 (m, 1H), 1.83 (m, 1H).

^{19}F NMR (471 MHz, $CDCl_3$) δ -133.41 (m), -145.04 (m), -158.37 (td, *J* = 20.9, 4.4 Hz).

^{13}C NMR (126 MHz, $CDCl_3$) δ 157.7 (C), 144.9 (dm, J = 260.2 Hz, CF), 144.1 (dm, *J* = 262.5 Hz, CF), 137.9 (dm, *J* = 258.4 Hz, CF), 136.4 (C), 133.7 (C), 128.7 (CH), 128.0 (CH), 127.9 (CH), 126.8 (C), 126.7 (CH), 126.6 (CH), 115.8 (m, C), 114.0 (CH), 112.6 (CH), 58.5 (CH), 55.5 (CH_3), 29.8 (CH_2), 25.4 (CH_2).

HRMS (APCI+) *m/z* calcd. for $C_{24}H_{19}F_5NO_3S$ $[M+H]^+$: 496.1000; found: 496.1003.

(*E*)-6-chloro-1-((perfluorophenyl)sulfonyl)-2-styryl-1,2,3,4-tetrahydroquinoline

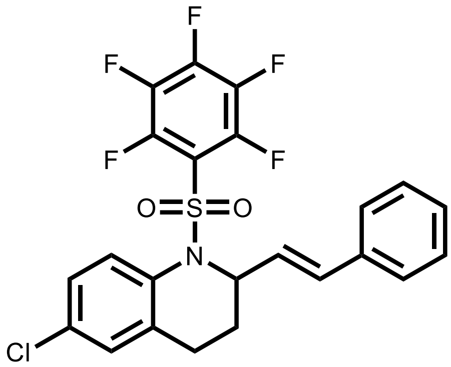

The residue was purified via silica gel flash chromatography (5% to 30% EtOAc/Hexane) to afford **3ha** as a yellow oil (49.4 mg, 99% yield)

^{1}H NMR (500 MHz, $CDCl_3$) δ 7.65 (d, *J* = 8.8 Hz, 1H), 7.18 (m, 6H), 7.00 (d, *J* = 2.5 Hz, 1H), 6.50 (d, *J* = 15.8 Hz, 1H), 6.01 (dd, *J* = 15.9, 5.7 Hz, 1H), 5.24 (q, *J* = 5.8, 5.2 Hz, 1H), 2.65 (ddd, *J* = 15.6, 9.4, 5.5 Hz, 1H), 2.41 (dt, *J* = 16.5, 5.7 Hz, 1H), 2.08 (ddt, *J* = 14.6, 9.6, 5.5 Hz, 1H), 1.84 (dq, *J* = 13.9, 5.8 Hz, 1H).

^{19}F NMR (471 MHz, $CDCl_3$) δ -133.79 (m), -144.12 (tt, *J* = 21.3, 7.0 Hz), -157.91 (tt, *J* = 21.0, 6.6 Hz).

^{13}C NMR (126 MHz, $CDCl_3$) δ 145.1 (dm, J = 260.2 Hz), 144.3 (dm, *J* = 263.4 Hz), 138.0 (dm, *J* = 258.4 Hz), 136.1 (C), 132.7 (C), 132.53 (C), 132.45 (CH), 131.3 (C), 129.1 (CH), 128.7 (CH), 128.2 (CH), 127.5 (CH), 127.0 (CH), 126.7 (CH), 125.2 (CH), 115.7 (m, C), 58.2 (CH), 28.2 (CH_2), 24.7 (CH_2).

HRMS (APCI+) *m/z* calcd. for $C_{23}H_{16}ClF_5NO_2S$ $[M+H]^+$: 500.0505; found: 500.0504.

Methyl (*E*)-1-((perfluorophenyl)sulfonyl)-2-styryl-1,2,3,4-tetrahydroquinoline-7-carboxylate

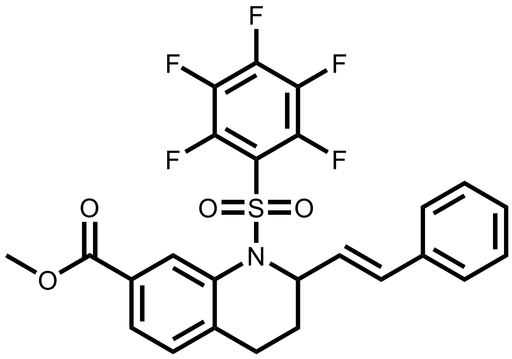

The residue was purified via silica gel flash chromatography (5% to 30% EtOAc/Hexane) to afford **3ia** as a yellow oil (30.9 mg, 59% yield)

^{1}H NMR (500 MHz, CDCl3) δ 8.38 (d, *J* = 1.6 Hz, 1H), 7.75 (dd, *J* = 7.9, 1.6 Hz, 1H), 7.25 (qt, *J* = 6.0, 1.4 Hz, 4H), 7.20 (m, 1H), 7.16 (m, 1H), 6.57 (dd, *J* = 15.9, 1.6 Hz, 1H), 6.08 (dd, *J* = 15.8, 5.5 Hz, 1H), 5.36 (qd, *J* = 5.5, 1.7 Hz, 1H), 3.90 (s, 3H), 2.80 (ddd, *J* = 16.1, 9.9, 5.6 Hz, 1H), 2.58 (dt, *J* = 16.8, 5.4 Hz, 1H), 2.17 (m, 1H), 1.95 (m, 1H).

^{19}F NMR (282 MHz, cdcl$_3$) δ -133.87 (m), -144.19 (tt, *J* = 21.1, 7.1 Hz), -158.15 (m).

^{13}C NMR (126 MHz, CDCl$_3$) δ 166.4 (CO), 145.2 (d, J = 254.3 Hz, CF), 144.4 (dm, *J* = 263.8 Hz, CF), 138.0 (dm, *J* = 259.8 Hz, CF), 136.1 (C), 135.4 (C), 134.4 (C), 132.5 (CH), 129.58 (CH), 129.55 (C), 128.7 (CH), 128.2 (CH), 126.9 (CH), 126.7 (CH), 126.5 (CH), 124.3 (CH), 115.7 (m, C), 58.0 (CH), 52.4 (CH$_3$), 27.9 (CH$_2$), 24.8 (CH$_2$).

HRMS (APCI+) *m/z* calcd. for $C_{25}H_{19}F_5NO_4S$ $[M+H]^+$: 524.0949; found: 524.0947

(*E*)-7-fluoro-1-((perfluorophenyl)sulfonyl)-2-styryl-1,2,3,4-tetrahydroquinoline

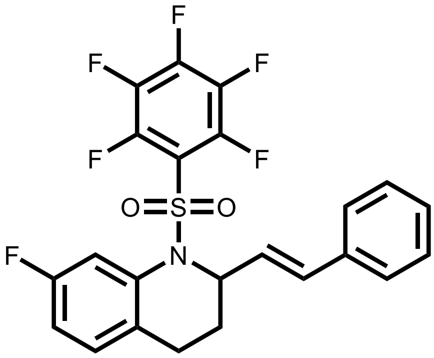

The residue was purified via silica gel flash chromatography (10% to 30% EtOAc/Hexane) to afford **3ja** as a yellow oil (40.1 mg, 83% yield)

^{1}H NMR (500 MHz, CDCl$_3$) δ 7.60 (dd, *J* = 10.9, 2.5 Hz, 1H), 7.30 (m, 4H), 7.24 (m, 1H), 7.05 (ddt, *J* = 8.2, 6.2, 0.9 Hz, 1H), 6.82 (td, *J* = 8.2, 2.6 Hz, 1H), 6.60 (dd, *J* = 15.8, 1.6 Hz, 1H), 6.12 (dd, *J* = 15.9, 5.6 Hz, 1H), 5.37 (qd, *J* = 5.6, 1.6 Hz, 1H), 2.75 (ddd, *J* = 15.9, 10.0, 5.6 Hz, 1H), 2.53 (dt, *J* = 16.3, 5.4 Hz, 1H), 2.15 (ddt, *J* = 13.7, 10.4, 5.3 Hz, 1H), 1.97 (dq, *J* = 13.7, 5.6 Hz, 1H).

^{19}F NMR (471 MHz, CDCl$_3$) δ -113.10, -133.95 (m), -144.12 (tt, *J* = 20.9, 7.0 Hz), -157.98 (m).

^{13}C NMR (126 MHz, CDCl$_3$) δ 161.3 (dm, *J* = 244.7 Hz, CF), 145.2 (dm, J = 260.7 Hz, CF), 144.4 (dm, J = 262.9 Hz, CF) 138.0 (dm, *J* = 257.5 Hz, CF), 136.1 (CH), 135.0 (d, *J* = 10.4 Hz, CH), 132.4 (CH), 130.4 (d, *J* = 8.8 Hz, CH), 128.7 (CH), 128.1 (CH), 127.0 (CH), 126.7 (CH), 125.7 (d, *J* = 3.2 Hz, C), 115.6 (m, C), 112.6 (d, *J* = 21.4 Hz, CH), 110.6 (d, *J* = 26.7 Hz, CH), 58.1 (CH), 28.0 (CH$_2$), 24.0 (CH$_2$).

HRMS (APCI+) *m/z* calcd. for $C_{23}H_{16}F_6NO_2S$ $[M+H]^+$: 484.0800; found: 484.0799.

(*E*)-7-chloro-1-((perfluorophenyl)sulfonyl)-2-styryl-1,2,3,4-tetrahydroquinoline

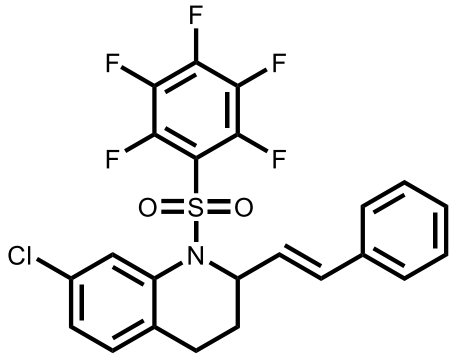

The residue was purified via silica gel flash chromatography (10% to 30% EtOAc/Hexane) to afford **3ka** as a yellow oil (24.9 mg, 50% yield)

^{1}H NMR (500 MHz, $CDCl_3$) δ 7.84 (d, *J* = 2.0 Hz, 1H), 7.28 (m, 5H), 7.08 (dd, *J* = 8.1, 2.0 Hz, 1H), 7.02 (d, *J* = 8.2 Hz, 1H), 6.59 (dd, *J* = 15.8, 1.6 Hz, 1H), 6.10 (dd, *J* = 15.9, 5.6 Hz, 1H), 5.34 (qd, *J* = 5.7, 1.6 Hz, 1H), 2.74 (ddd, *J* = 15.8, 9.8, 5.6 Hz, 1H), 2.51 (dt, *J* = 16.4, 5.5 Hz, 1H), 2.15 (m, 1H), 1.94 (dq, *J* = 13.8, 5.7 Hz, 1H).

^{19}F NMR (471 MHz, $CDCl_3$) δ -133.86 (m), -144.01 (tt, *J* = 21.0, 7.0 Hz), -157.95 (m).

^{13}C NMR (126 MHz, $CDCl_3$) δ 145.1 (dm, J = 260.7 Hz, CF), 144.4 (dm, J = 268.0 Hz, CF), 138.0 (dm, J = 258.8 Hz, CF), 136.1 (C), 135.1 (C), 132.7 (C), 132.5 (CH), 130.3 (CH), 128.7 (CH), 128.2 (CH), 126.9 (CH), 126.7 (CH), 125.8 (CH), 123.5 (CH), 115.6 (m, C), 58.2 (CH), 28.1 (CH_2), 24.2 (CH_2).

HRMS (APCI+) *m/z* calcd. for $C_{23}H_{16}ClF_5NO_2S$ $[M+H]^+$: 500.0505; found: 500.0504.

(*E*)-7-methoxy-1-((perfluorophenyl)sulfonyl)-2-styryl-1,2,3,4-tetrahydroquinoline

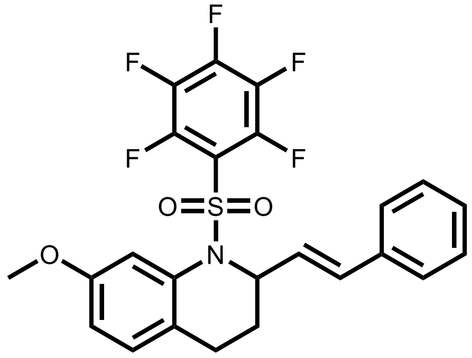

The residue was purified via silica gel flash chromatography (5% to 20% EtOAc/Hexane) to afford **3la** as a yellow oil (27.7 mg, 56% yield)

^{1}H NMR (500 MHz, $CDCl_3$) δ 7.42 (d, *J* = 2.5 Hz, 1H), 7.31 (m, 4H), 7.23 (m, 1H), 6.98 (d, J = 8.4 Hz, 1H), 6.67 (dd, *J* = 8.4, 2.5 Hz, 1H), 6.64 (dd, *J* = 15.9, 1.5 Hz, 1H), 6.16 (dd, *J* = 15.9, 5.8 Hz, 1H), 5.39 (qd, *J* = 5.7, 1.5 Hz, 1H), 3.82 (s, 3H), 2.71 (ddd, *J* = 15.6, 9.7, 5.5 Hz, 1H), 2.47 (dt, *J* = 16.0, 5.5 Hz, 1H), 2.17 (ddt, *J* = 13.7, 9.7, 5.3 Hz, 1H), 1.93 (dq, *J* = 13.6, 5.8 Hz, 1H).

^{19}F NMR (471 MHz, $CDCl_3$) δ -133.95 (m), -144.64 (tt, *J* = 21.4, 6.8 Hz), -158.33 (m).

^{13}C NMR (126 MHz, $CDCl_3$) δ 158.7 (C), 145.1 (dm, J = 260.2 Hz, CF), 144.2 (dm, J = 262.5 Hz, CF), 137.9 (dm, J = 258.4 Hz, CF), 136.4 (C), 134.7 (C), 132.1 (CH), 130.0 (CH), 128.7 (CH), 128.0 (CH), 127.7 (CH), 126.7 (CH), 122.2 (C), 115.8 (m, C), 112.3 (CH), 108.2 (CH), 58.4 (CH_3), 55.5 (CH), 28.6 (CH_2), 23.9 (CH_2).

HRMS (APCI+) *m/z* calcd. for $C_{24}H_{19}F_5NO_3S$ $[M+H]^+$: 496.1000; found: 496.1002.

(*E*)-7-nitro-1-((perfluorophenyl)sulfonyl)-2-styryl-1,2,3,4-tetrahydroquinoline

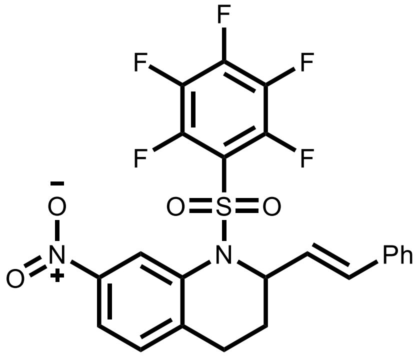

Another 5 mol% Pd(OAc)$_2$, 10 mol% of quinaldine and 1 equivalent of copper was added after 24h**.** The residue was purified via silica gel flash chromatography (10% to 30% EtOAc/Hexane) to afford **3ma** as a yellow oil (32.6 mg, 64% yield)

^{1}H NMR (300 MHz, CDCl$_3$) δ 8.71 (d, J = 2.2 Hz, 1H), 7.96 (dd, J = 8.4, 2.3 Hz, 1H), 7.29 (m, 6H), 6.58 (dd, J = 15.9, 1.7 Hz, 1H), 6.08 (dd, J = 15.9, 5.4 Hz, 1H), 5.40 (q, J = 5.4 Hz, 1H), 2.91 (ddd, J = 16.8, 10.2, 6.1 Hz, 1H), 2.73 (dt, J = 17.3, 5.1 Hz, 1H), 2.12 (m, 2H).

^{19}F NMR (471 MHz, CDCl$_3$) δ -132.15 (m), -143.06 (m), -157.39 (m).

^{13}C NMR (126 MHz, CDCl$_3$) δ 147.2 (C), 145.3 (dm, J = 260.8 Hz, CF), 144.7 (dm, J = 264.3 Hz, CF), 138.1 (dm, J = 259.8 Hz, CF) 136.8 (C), 135.8 (C), 135.2 (C), 133.1 (CH), 130.3 (CH), 128.8 (CH), 128.4 (CH), 126.7 (CH), 126.0 (CH), 120.0 (CH), 118.3 (CH), 115.4 (m, C), 57.8 (CH), 27.1 (CH$_2$), 24.7 (CH$_2$).

HRMS (APCI+) *m/z* calcd. for $C_{23}H_{16}F_5N_2O_4S$ $[M+H]^+$: 511.0745; found: 511.0751

(*E*)-8-methyl-1-((perfluorophenyl)sulfonyl)-2-styryl-1,2,3,4-tetrahydroquinoline

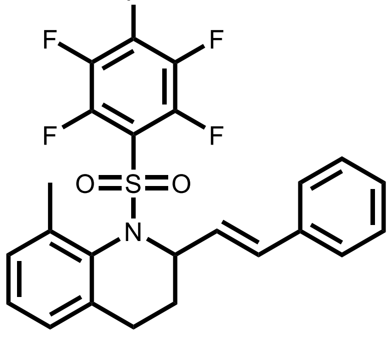

Another 5 mol% Pd(OAc)$_2$, 10 mol% of quinaldine and 1 equivalent of copper was added after 24h. The residue was purified via silica gel flash chromatography (5% to 20% EtOAc/Hexane) to afford **3na** as a yellow oil (25.4 mg, 53% yield)

^{1}H NMR (300 MHz, cdcl$_3$) δ 7.26 (m, 7H), 6.94 (d, *J* = 7.1 Hz, 1H), 6.68 (d, *J* = 15.9 Hz, 1H), 6.06 (dd, *J* = 15.8, 6.8 Hz, 1H), 5.09 (q, *J* = 7.9 Hz, 1H), 2.48 (m, 4H), 2.42 (m, 1H), 1.75 (s, 1H), 1.56 (m, 1H).

^{19}F NMR (471 MHz, CDCl$_3$) δ -132.77 (m), -145.13 (tt, *J* = 21.0, 6.5 Hz), -158.15 (m).

^{13}C NMR (126 MHz, CDCl$_3$) δ 144.9 (dm, *J* = 260.7 Hz, CF), 144.1 (dm, *J* = 262.0 Hz, CF), 138.9 (C), 138.8 (C), 137.9 (d, *J* = 262.5 Hz, CF), 136.4 (C), 133.0 (C), 131.8 (CH), 130.5 (CH), 129.0 (CH), 128.7 (CH), 128.09 (CH), 128.03 (CH), 126.7 (CH), 125.1 (CH), 116.4 (m, C), 60.0 (CH), 32.2 (CH$_2$), 27.3 (CH$_2$), 19.5 (CH$_3$).

HRMS (APCI+) *m/z* calcd. for $C_{24}H_{19}F_5NO_2S$ $[M+H]^+$:480.1051; found: 480.1050.

(*E*)-1-((perfluorophenyl)sulfonyl)-2-styryl-5-(trifluoromethyl)-1,2,3,4-tetrahydroquinoline

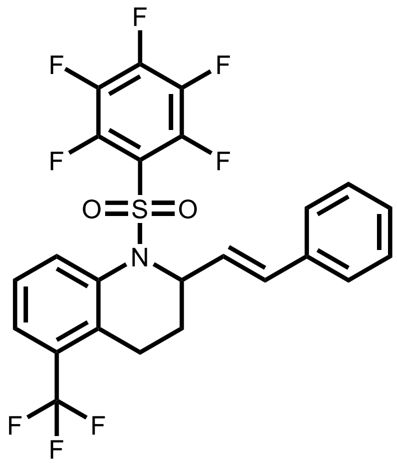

The residue was purified via silica gel flash chromatography (5% to 30% EtOAc/Hexane) to afford **3pa** as a yellow oil (28.8 mg, 54% yield)

^{1}H NMR (500 MHz, $CDCl_3$) δ 7.93 (d, *J* = 8.3 Hz, 1H), 7.46 (d, *J* = 7.9 Hz, 1H), 7.27 (m, 6H), 6.57 (d, *J* = 15.9 Hz, 1H), 6.05 (dd, *J* = 15.8, 6.1 Hz, 1H), 5.24 (q, *J* = 6.6 Hz, 1H), 2.93 (dt, *J* = 16.2, 6.1 Hz, 1H), 2.40 (ddd, *J* = 16.5, 8.6, 4.7 Hz, 1H), 2.28 (dtd, *J* = 14.0, 7.2, 4.7 Hz, 1H), 1.82 (m, 1H).

^{19}F NMR (471 MHz, $CDCl_3$) δ -60.60, -133.89 (m), -143.88 (tt, *J* = 21.1, 7.1 Hz), -157.89 (tt, *J* = 21.1, 6.7 Hz).

^{13}C NMR (126 MHz, $CDCl_3$) δ 145.0 (dm, *J* = 260.2 Hz, CF), 144.4 (dm, *J* = 263.4 Hz, CF), 138.0 (dm, *J* = 258.9 Hz, CF), 136.1 (C), 135.7 (C), 133.7 (C), 131.8 (C) 132.5 (CH), 129.0 (CH), 128.7 (CH), 128.3 (CH), 127.2 (CH), 127.1 (CH), 126.7 (CH), 123.96 (q, J = 273.8 Hz, CF), 123.76 (q, J = 5.7 Hz, CH), 115.6 (m, C), 58.5 (CH), 29.2 (CH_2), 21.7 (d, *J* = 2.7 Hz, CH_2).

HRMS (APCI+) *m/z* calcd. for $C_{24}H_{16}F_8NO_2S$ $[M+H]^+$: 534.0769; found: 534.0772.

(*E*)-2-(4-nitrostyryl)-1-((perfluorophenyl)sulfonyl)-1,2,3,4-tetrahydroquinoline

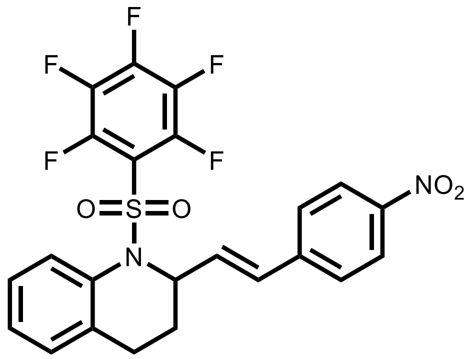

The residue was purified via silica gel flash chromatography (5% to 30% EtOAc/Hexane) to afford **3eb** as an orange oil (41.8 mg, 82% yield)

^{1}H NMR (500 MHz, $CDCl_3$) δ 8.14 (d, *J* = 8.9 Hz, 2H), 7.80 (d, *J* = 8.3 Hz, 1H), 7.46 (d, *J* = 8.8 Hz, 2H), 7.24 (m, 1H), 7.11 (m, 2H), 6.71 (d, *J* = 15.9 Hz, 1H), 6.35 (dd, *J* = 15.9, 5.5 Hz, 1H), 5.39 (q, *J* = 6.2 Hz, 1H), 2.72 (m, 1H), 2.47 (m, 1H), 2.28 (m, 1H), 1.91 (dq, *J* = 13.3, 6.6 Hz, 1H)

^{19}F NMR (471 MHz, $CDCl_3$) δ -133.79 (m), -144.16 (tt, *J* = 21.4, 7.0 Hz), -158.07 (tt, *J* = 21.2, 6.8 Hz).

^{13}C NMR (126 MHz, $CDCl_3$) δ 147.3 (C), 145.1 (d, J = 259.8 Hz, CF), 144.3 (dm, *J* = 263.4 Hz, CF), 142.8 (C), 137.9 (dm, *J* = 258.4 Hz, CF), 133.9 (C), 133.0 (CH), 131.1 (C), 130.0 (CH), 129.2 (CH), 127.7 (CH), 127.3 (CH), 126.1 (CH), 124.1 (CH), 123.7 (CH), 115.4 (m, C), 58.5 (CH), 29.0 (CH_2), 25.1 (CH_2).

HRMS (APCI+) *m/z* calcd. for $C_{23}H_{16}F_5N_2O_4S$ $[M+H]^+$:511.0745; found: 511.0746.

(*E*)-2-(4-fluorostyryl)-1-((perfluorophenyl)sulfonyl)-1,2,3,4-tetrahydroquinoline

Another 5 mol% $Pd(OAc)_2$, 10 mol% of quinaldine and 1 equiv of copper was added after 24h.

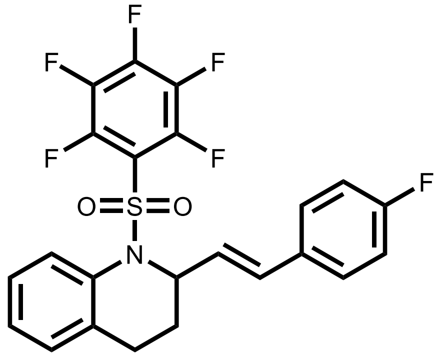

The residue was purified via silica gel flash chromatography (0% to 2% Et_2O/Pentane) to afford **3ec** as a yellow oil (19.3 mg, 40% yield)

^{1}H NMR (500 MHz, $CDCl_3$) δ 7.78 (d, *J* = 8.2 Hz, 1H), 7.28 (dd, *J* = 8.5, 5.4 Hz, 2H), 7.23 (ddd, *J* = 8.6, 6.8, 2.4 Hz, 1H), 7.10 (m, 2H), 6.96 (m, 2H), 6.60 (d, *J* = 15.8 Hz, 1H), 6.07 (dd, *J* = 15.9, 5.8 Hz, 1H), 5.34 (q, *J* = 6.1 Hz, 1H), 2.74 (ddd, *J* = 16.2, 8.8, 5.3 Hz, 1H), 2.47 (ddd, *J* = 16.1, 7.0, 4.9 Hz, 1H), 2.23 (ddt, *J* = 14.1, 9.1, 5.5 Hz, 1H), 1.89 (ddd, *J* = 13.8, 11.9, 6.2 Hz, 1H).

^{19}F NMR (471 MHz, $CDCl_3$) δ -113.99, -133.85 (m), -144.72 (tt, *J* = 20.8, 6.9 Hz), -158.34 (m).

^{13}C NMR (126 MHz, $CDCl_3$) δ 162.9 (d, *J* = 247.0 Hz, CF), 145.1 (ddq, J = 260.2, 13.2, 4.5 Hz, CF), 144.2 (dm, *J* = 262.5 Hz, CF), 137.9 (dm, *J* = 257.9 Hz, CF), 134.1 (C), 132.5 (d, *J* = 3.6 Hz, C), 131.2 (C), 130.9 (CH), 129.2 (CH), 128.3 (d, *J* = 8.2 Hz, CH), 127.6 (d, *J* = 2.3 Hz, CH), 127.4 (CH), 125.9 (CH), 123.9 (CH), 115.8 (m, CF), 115.6 (d, *J* = 21.3 Hz, CH), 58.5 (CH_3), 28.9 (CH_2), 24.9 (CH_2).

HRMS (APCI+) *m/z* calcd. for $C_{23}H_{16}F_6NO_2S$ $[M+H]^+$:484.0800; found: 484.0804.

(*E*)-1-((perfluorophenyl)sulfonyl)-2-(4-(trifluoromethyl)styryl)-1,2,3,4-tetrahydroquinoline

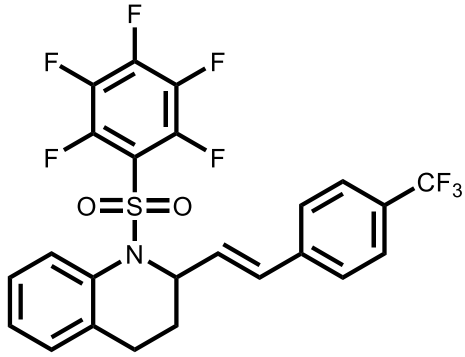

The residue was purified via silica gel flash chromatography (10% to 30% EtOAc/Hexane) to afford **3ed** as a yellow oil (40.5 mg, 76% yield)

^{1}H NMR (500 MHz, Chloroform-*d*) δ 7.77 (d, *J* = 8.3 Hz, 1H), 7.52 (d, *J* = 8.1 Hz, 2H), 7.40 (d, *J* = 8.1 Hz, 2H), 7.23 (m, 1H), 7.09 (m, 2H), 6.66 (d, *J* = 15.8 Hz, 1H), 6.24 (dd, *J* = 15.9, 5.6 Hz, 1H), 5.36 (q, *J* = 6.1 Hz, 1H), 2.71 (ddd, *J* = 16.2, 8.7, 5.2 Hz, 1H), 2.46 (ddd, *J* = 16.1, 7.1, 4.9 Hz, 1H), 2.24 (ddt, *J* = 14.0, 8.9, 5.7 Hz, 1H), 1.89 (dtd, *J* = 14.0, 6.9, 5.4 Hz, 1H).

^{19}F NMR (471 MHz, $CDCl_3$) δ -62.58, -133.80 (m), -144.43 (tt, *J* = 21.4, 6.9 Hz), -158.19 (m).

^{13}C NMR (126 MHz, $CDCl_3$) δ 145.2 (dm, *J* = 255.7 Hz, CF), 144.3 (dm, J = 263.4 Hz, CF), 139.8 (C), 137.9 (dm, *J* = 258.4 Hz, CF), 134.0 (C), 131.1 (C), 130.71 (CH), 130.68 (CH), 129.8 (q, *J* = 32.6 Hz, C), 129.2 (CH), 127.6 (CH), 126.9 (CH), 126.0 (CH), 125.6 (q, *J* = 3.8 Hz, CH), 124.2 (q, *J* = 271.6 Hz, CF_3), 123.8 (CH), 115.6 (m, C), 58.4 (CH), 28.9 (CH_2), 25.0 (CH_2).

HRMS (APCI+) *m/z* calcd. for $C_{24}H_{16}F_8NO_2S$ $[M+H]^+$:534.0769; found: 534.0776.

(*E*)-2-(4-methoxystyryl)-1-((perfluorophenyl)sulfonyl)-1,2,3,4-tetrahydroquinoline

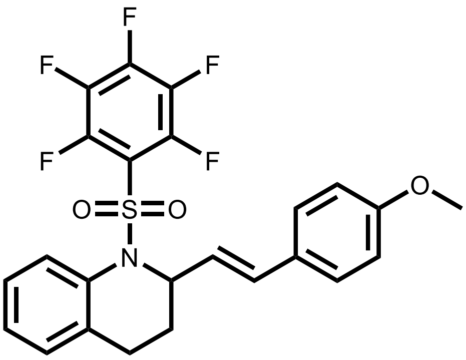

The residue was purified via silica gel flash chromatography (10% to 30% EtOAc/Hexane) to afford **3ee** as a yellow oil (34.6 mg, 70% yield)

^{1}H NMR (500 MHz, $CDCl_3$) δ 7.75 (d, *J* = 8.4 Hz, 1H), 7.21 (m, 3H), 7.07 (d, *J* = 6.6 Hz, 2H), 6.78 (d, *J* = 8.8 Hz, 2H), 6.55 (d, *J* = 14.4 Hz, 1H), 5.98 (dd, *J* = 15.8, 5.9 Hz, 1H), 5.29 (m, 1H), 3.76 (s, 3H), 2.72 (m, 1H), 2.45 (m, 1H), 2.19 (ddt, *J* = 14.1, 8.9, 5.4 Hz, 1H), 1.87 (ddd, *J* = 13.2, 11.7, 6.1 Hz, 1H).

^{19}F NMR (471 MHz, Chloroform-*d*) δ -133.87 (dq, *J* = 20.0, 8.1, 6.9 Hz), -145.26 (m), -158.43 (td, *J* = 20.6, 4.2 Hz).

^{13}C NMR (126 MHz, $CDCl_3$) δ 159.6 (C), 145.0 (dm, J = 260.2 Hz, CF), 144.1 (dm, *J* = 262.9 Hz, CF), 137.9 (dm, *J* = 258.4 Hz, CF), 134.2 (C), 131.5 (CH), 131.2 (C), 129.13 (CH), 129.06 (C), 127.9 (CH), 127.3 (CH), 125.8 (CH), 125.4 (CH), 124.0 (CH), 115.9 (m, C), 114.0 (CH), 58.6 (CH), 55.4 (CH_3), 28.9 (CH_2), 24.9 (CH_2).

HRMS (APCI+) *m/z* calcd. for $C_{24}H_{19}F_5NO_3S$ $[M+H]^+$: 496.1000; found: 496.0999.

(*E*)-2-(2-methoxystyryl)-1-((perfluorophenyl)sulfonyl)-1,2,3,4-tetrahydroquinoline

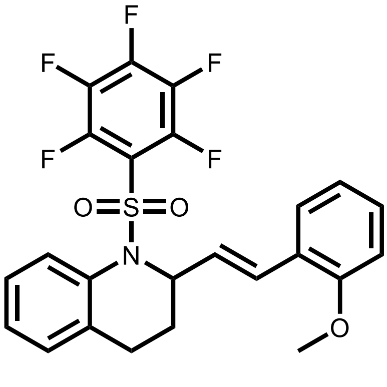

The residue was purified via silica gel flash chromatography (1% to 10% Et_2O/Hexane) to afford **3ef** as a yellow oil (26.3 mg, 53% yield)

^{1}H NMR (500 MHz, $CDCl_3$) δ 7.76 (m, 1H), 7.32 (dd, *J* = 7.6, 1.7 Hz, 1H), 7.22 (m, 2H), 7.11 (m, 2H), 6.88 (m, 2H), 6.83 (dd, *J* = 8.3, 1.0 Hz, 1H), 6.14 (dd, *J* = 16.0, 6.2 Hz, 1H), 5.31 (qd, *J* = 6.1, 1.5 Hz, 1H), 3.80 (s, 3H), 2.76 (ddd, *J* = 16.1, 8.8, 5.4 Hz, 1H), 2.52 (ddd, *J* = 16.1, 6.9, 5.1 Hz, 1H), 2.26 (dddd, *J* = 13.8, 8.8, 6.0, 5.1 Hz, 1H), 1.93 (m, 1H).

^{19}F NMR (471 MHz, $CDCl_3$) δ -133.85 (m), -145.19 (tt, *J* = 21.4, 6.8 Hz), -158.57 (m).

^{13}C NMR (126 MHz, $CDCl_3$) δ 156.9 (C), 1454.0 (dm, J = 260.2 Hz, CF), 144.1 (dm, *J* = 262.5 Hz, CF), 137.9 (dm, *J* = 258.1 Hz, CF), 134.3 (C), 131.5 (C), 129.10 (CH), 129.06 (CH), 127.9 (CH), 127.2 (CH), 127.08 (CH), 127.04 (CH), 125.8 (CH), 125.3 (C), 124.5 (CH), 120.7 (CH), 116.1 (m, C), 111.1 (CH), 58.9 (CH), 55.6 (CH_3), 29.1 (CH_2), 24.9 (CH_2).

HRMS (APCI+) *m/z* calcd. for $C_{24}H_{19}F_5NO_3S$ $[M+H]^+$:496.1000; found: 496.1002.

(*E*)-1-((perfluorophenyl)sulfonyl)-2-(2-phenylprop-1-en-1-yl)-1,2,3,4-tetrahydroquinoline

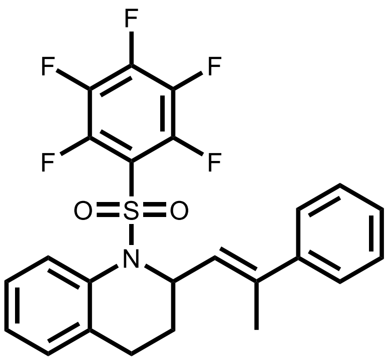

The residue was purified via silica gel flash chromatography (10% to 30% EtOAc/Hexane) to afford **3eg** as a yellow oil (24.9 mg, 52% yield)

^{1}H NMR (500 MHz, $CDCl_3$) δ 7.70 (d, *J* = 8.2 Hz, 1H), 7.31 (m, 3H), 7.28 (m, 1H), 7.23 (m, 2H), 7.12 (m, 2H), 5.67 (dd, *J* = 8.8, 1.6 Hz, 1H), 5.51 (dt, *J* = 8.9, 6.4 Hz, 1H), 2.76 (ddd, *J* = 16.0, 8.2, 5.3 Hz, 1H), 2.46 (ddd, *J* = 16.1, 7.6, 5.1 Hz, 1H), 2.22 (m, 4H), 1.78 (m, 1H).

^{19}F NMR (471 MHz, $CDCl_3$) δ -133.77 (m), -145.07 (tt, *J* = 21.0, 6.7 Hz), -158.47 (m).

^{13}C NMR (126 MHz, $CDCl_3$) δ 142.8 (C), 137.4 (C), 134.1 (C), 131.9 (C), 129.0 (CH), 128.4 (CH), 127.6 (CH), 127.4 (CH), 126.1 (CH), 126.0 (CH), 124.8 (CH), 56.0 (CH), 29.6 (CH_2), 25.2 (CH_2), 16.4 (CH_3).

HRMS (APCI+) *m/z* calcd. for $C_{24}H_{19}F_5NO_2S$ $[M+H]^+$:480.1051; found: 480.1052.

Ethyl (*E*)-3-(1-((perfluorophenyl)sulfonyl)-1,2,3,4-tetrahydroquinolin-2-yl)acrylate

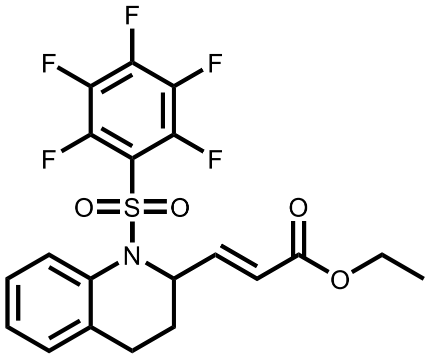

The residue was purified via silica gel flash chromatography (5% to 30% EtOAc/Hexane) to afford **3ei** as a yellow oil (23.9 mg, 52% yield)

^{1}H NMR (500 MHz, $CDCl_3$) δ 7.75 (m, 1H), 7.23 (ddd, *J* = 8.3, 7.3, 1.9 Hz, 1H), 7.10 (td, *J* = 7.4, 1.1 Hz, 1H), 7.07 (m, 1H), 6.88 (dd, *J* = 15.6, 4.6 Hz, 1H), 6.03 (dd, *J* = 15.6, 1.8 Hz, 1H), 5.35 (tdd, *J* = 6.2, 4.4, 1.8 Hz, 1H), 4.16 (qd, *J* = 7.1, 2.6 Hz, 2H), 2.67 (ddd, *J* = 16.3, 9.1, 5.3 Hz, 1H), 2.46 (ddd, *J* = 16.3, 7.1, 5.2 Hz, 1H), 2.22 (dddd, *J* = 13.8, 9.1, 6.2, 4.8 Hz, 1H), 1.88 (dtd, *J* = 13.4, 6.6, 5.3 Hz, 1H), 1.26 (t, *J* = 7.1 Hz, 3H).

^{19}F NMR (471 MHz, Chloroform-*d*) δ -133.78 (m), -144.11 (tt, *J* = 20.9, 7.0 Hz), -158.02 (m).

^{13}C NMR (126 MHz, $CDCl_3$) δ 166.0 (C), 145.4 (CH), 145.1 (dm, J = 268.1 Hz, CF), 144.4 (dm, J = 271.7 Hz, CF). 137.9 (dm, J = 258.7 Hz, CF), 133.7 (C), 130.5 (C), 129.3 (CH), 127.7 (CH), 126.0 (CH), 123.5 (CH), 123.0 (CH), 115.4 (m,CH), 60.8 (CH_2), 57.3 (CH), 28.0 (CH_2), 24.9 (CH_2), 14.3 (CH_3).

HRMS (APCI+) *m/z* calcd. for $C_{20}H_{17}F_5NO_4S$ $[M+H]^+$:462.0793; found: 462.0792.

(*E*)-2-(oct-1-en-1-yl)-1-((perfluorophenyl)sulfonyl)-1,2,3,4-tetrahydroquinoline

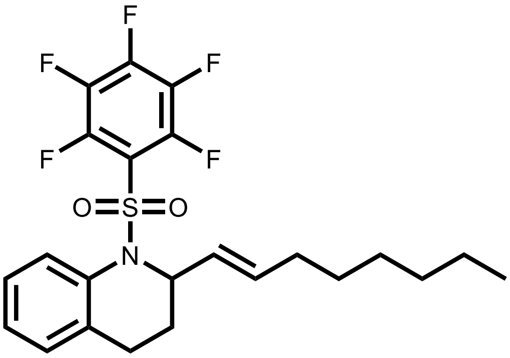

Another 5 mol% $Pd(OAc)_2$, 10 mol% of quinaldine and 1 equivalent of copper was added after 24h**.** 5 equivalents of diene were used**.**

The residue was purified via silica gel flash chromatography (10% to 30% EtOAc/Hexane) to afford **3ej** as a yellow oil (29.8 mg, 63% yield)

^{1}H NMR (500 MHz, $CDCl_3$) δ 7.62 (m, 1H), 7.11 (m, 1H), 6.99 (m, 2H), 5.61 (dtd, *J* = 15.2, 6.8, 1.4 Hz, 1H), 5.31 (ddt, *J* = 15.3, 5.6, 1.5 Hz, 1H), 5.05 (qd, *J* = 5.8, 1.3 Hz, 1H), 2.62 (ddd, *J* = 16.4, 9.3, 5.6 Hz, 1H), 2.38 (m, 1H), 2.02 (ddt, *J* = 13.7, 9.3, 5.5 Hz, 1H), 1.89 (m, 2H), 1.71 (dq, *J* = 13.6, 5.8 Hz, 1H), 1.15 (m, 8H), 0.77 (t, *J* = 7.1 Hz, 3H).

^{19}F NMR (471 MHz, $CDCl_3$) δ -133.93 (m), -145.27 (tt, *J* = 21.4, 6.7 Hz), -158.61 (tt, *J* = 21.1, 6.8 Hz).

^{13}C NMR (126 MHz, $CDCl_3$) δ 145.0 (dm, *J* = 260.2 Hz, CF), 144.1 (dm, J = 262.0 Hz, CF), 137.9 (dm, *J* = 257.9 Hz, CF), 134.3 (C), 133.7 (CH), 131.0 (C), 129.1 (CH), 127.8 (CH), 127.2 (CH), 125.6 (CH), 123.8 (CH), 116.1 (m, C), 58.14 (CH), 32.2 (CH2), 31.7 (CH2), 29.0 (CH2), 28.7 (CH_2), 28.6 (CH_2), 24.6 (CH_2), 22.7 (CH_2), 14.2 (CH_3).

HRMS (APCI+) *m/z* calcd. for $C_{23}H_{25}F_5NO_2S$ $[M+H]^+$:474.1521; found: 474.1518.

General procedure for the formal C-H activation/annulation between *ortho*-methylbenzenesulfamides and acrylates/styrene

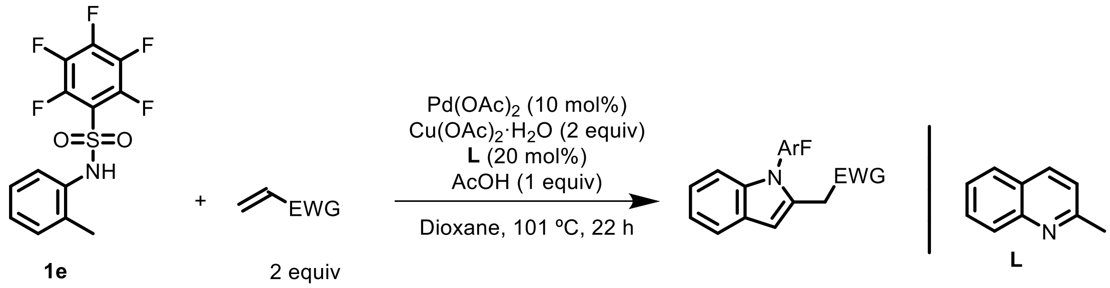

2,3,4,5,6-pentafluoro-*N*-(*o*-tolyl)benzenesulfonamide **1e** (33.7 mg, 0.10 mmol, 1.0 equiv), $Pd(OAc)_2$ (2.2 mg, 10 mol%), $Cu(OAc)_2·H_2O$ (39.9 mg, 2.0 equiv) were weighed and added into a Schlenk flask under air. Then, 1,4-dioxane (2.0 mL, 0.05 M), quinaldine (2.8 µL, 20 mol%), AcOH (5.7 µL, 1 equiv) and the alkene (0.20 mmol, 2.0 equiv) were added. The mixture was refluxed at 101 ºC in a Schlenk heating block for 20-24 h using a cold finger setup. After cooling to rt, the reaction mixture was diluted with DCM and filtered through a Fluorisil-silica gel pad, washing the flask and the pad with more DCM (x3). The filtrate was concentrated under reduced pressure and the resulting residue was purified by flash column chromatography to afford the corresponding products.

(*E*)-2-benzyl-1-((perfluorophenyl)sulfonyl)-2-styrylindoline

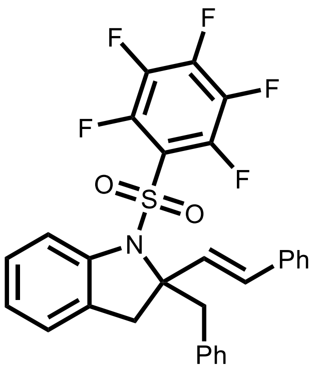

The residue was purified by preparative reverse-phase HPLC 20.0 mL/min, gradient 55 to 80% B over 14 min (A: H_2O 0.1% TFA, B: MeCN 0.1% TFA) on a preparative Agilent 1260 Infinity II using a Luna 5u C18(2) 100A (250 x 10 mm, 5mm) reverse-phase column from Phenomenex to afford **6** as a white solid (4.3 mg, 8% yield)

^{1}H NMR (500 MHz, Chloroform-*d*) δ 7.63 (d, *J* = 8.3 Hz, 1H), 7.31 (d, *J* = 6.2 Hz, 2H), 7.26 (s, 2H), 7.21 (m, 4H), 7.15(m, 3H), 7.09 (d, *J* = 7.5 Hz, 1H), 7.00 (t, *J* = 7.4 Hz, 1H), 6.68 (d, *J* = 16.4 Hz, 1H), 6.42 (d, *J* = 16.4 Hz, 1H), 3.52 (d, *J* = 13.6 Hz, 1H), 3.40 (m, 2H), 3.21 (d, *J* = 16.1 Hz, 1H).

^{19}F NMR (471 MHz, Chloroform-*d*) δ -134.06 (m), -147.09 (tt, *J* = 20.8, 6.1 Hz), -159.18 (m).

^{13}C NMR (126 MHz, Chloroform-*d*) δ 141.4 (C), 135.4 (C), 135.1 (C), 133.6 (CH), 130.8 (CH), 129.0 (CH), 128.9 (CH), 128.4 (C), 128.2 (CH), 128.0 (CH), 127.8 (CH), 127.1 (CH) 126.4 (CH), 124.8 (CH), 124.4 (CH), 116.0 (CH), 74.9 (C), 45.8 (CH_2), 38.7 (CH_2).

HRMS (APCI+) m/z calcd. for $C_{29}H_{21}F_5NO_2S$ $[M+H]^+$:542.1208; found: 542.1215.

Benzyl 2-(1-((perfluorophenyl)sulfonyl)-1*H*-indol-2-yl)acetate

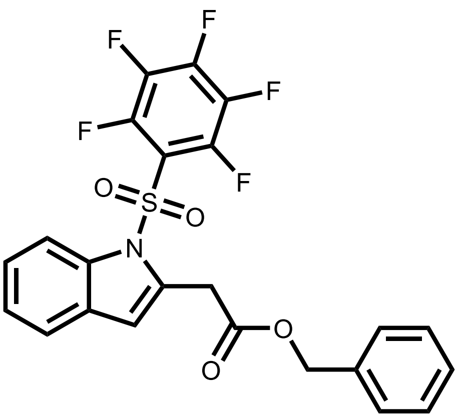

The residue was purified via silica gel flash chromatography (10% to 50% EtOAc/Hexane) to afford **7ea** as a yellow oil (41.6 mg, 84% yield)

^{1}H NMR (300 MHz, $CDCl_3$) δ 7.88 (d, *J* = 8.0 Hz, 1H), 7.52 (m, 1H), 7.36 (m, 5H), 7.31 (ddd, *J* = 8.9, 7.4, 1.4 Hz, 2H), 6.66 (s, 1H), 5.18 (s, 2H), 4.18 (s, 2H).

^{19}F NMR (282 MHz, $CDCl_3$) δ -135.03 (m), -142.94 (tt, *J* = 21.0, 7.4 Hz), -157.61 (m).

^{13}C NMR (75 MHz, $CDCl_3$) δ 169.6 (C), 144.9 (dm, J = 256.6 Hz, CF), 138.0 (dm, J = 259.5 Hz, CF), 136.5 (C), 135.6 (C), 134.1 (C), 129.3 (C), 128.7 (CH), 128.5 (CH), 128.4 (CH), 125.4 (CH), 124.7 (CH), 121.4 (CH), 113.9 (CH), 113.1 (CH), 67.3 (CH_2), 34.9 (CH_2).

HRMS (APCI+) *m/z* calcd. for $C_{23}H_{15}F_5NO_4S$ $[M+H]^+$:496.0636; found: 496.0633.

Methyl 2-(1-((perfluorophenyl)sulfonyl)-1*H*-indol-2-yl)acetate

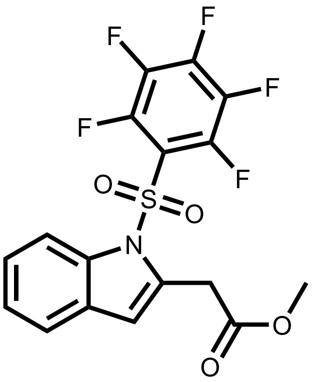

The residue was purified via silica gel flash chromatography (10% to 20% EtOAc/Hexane) to afford **7eb** as a yellow oil (25.6 mg, 61% yield)

^{1}H NMR (500 MHz, $CDCl_3$) δ 7.87 (d, *J* = 8.0 Hz, 1H), 7.52 (d, *J* = 7.3 Hz, 1H), 7.30 (q, *J* = 7.6 Hz, 2H), 6.65 (s, 1H), 4.12 (s, 2H), 3.74 (s, 3H).

^{19}F NMR (471 MHz, $CDCl_3$) δ -135.05 (m), 142.77 (tt, *J* = 20.9, 7.5 Hz), -157.51 (m).

^{13}C NMR (126 MHz, $CDCl_3$) δ 170.1 (C), 136.5 (C), 134.2 (C), 129.3 (C), 125.4 (CH), 124.7 (CH), 121.4 (CH), 113.9 (CH), 113.0 (CH), 52.5 (CH_3), 34.7 (CH_2).

HRMS (APCI+) *m/z* calcd. for $C_{17}H_{11}F_5NO_4S$ $[M+H]^+$:420.0323; found: 420.0323.

***N*,*N*-dimethyl-2-(1-((perfluorophenyl)sulfonyl)-1*H*-indol-2-yl)acetamide**

The residue was purified by preparative reverse-phase HPLC 20.0 mL/min, gradient 55 to 80% B over 14 min (A: H_2O 0.1% TFA, B: MeCN 0.1% TFA) on a preparative Agilent 1260 Infinity II using a Luna 5u C18(2) 100A (250 x 10 mm, 5mm) reverse-phase column from Phenomenex to afford **7ec** as a yellow oil (19.0 mg, 44% yield)

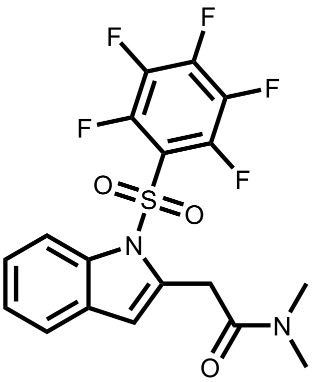

^{1}H NMR (500 MHz, $CDCl_3$) δ 7.96 (d, *J* = 8.1 Hz, 1H), 7.49 (m, 1H), 7.29 (m, 2H), 6.57 (s, 1H), 4.12 (s, 2H), 3.10 (s, 3H), 2.94 (s, 3H).

^{19}F NMR (471 MHz, $CDCl_3$) δ -134.77 (m), 143.54 (tt, *J* = 20.9, 7.3 Hz), -158.28 (m).

^{13}C NMR (126 MHz, $CDCl_3$) δ 168.8 (C), 136.9 (C), 135.3 (C), 129.3 (C), 125.0 (CH), 124.5 (CH), 121.1 (CH), 114.4 (CH), 112.7 (CH), 37.6 (CH_2), 35.7 (CH_3), 34.3 (CH_3).

HRMS (APCI+) *m/z* calcd. for $C_{18}H_{14}F_5N_2O_3S$ $[M+H]^+$:433.0640; found: 433.0642.

1-((perfluorophenyl)sulfonyl)-2-((phenylsulfonyl)methyl)-1*H*-indole

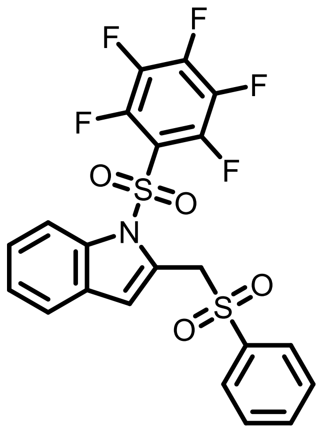

The residue was purified via silica gel flash chromatography (10% to 20% Et_2O/Hexane) to afford **7ed** as a yellow oil (27.6 mg, 55% yield) using only 0.5 equivalents of (vinylsulfonyl)benzene.

1H NMR (300 MHz, CDCl3) δ 7.85 (d, *J* = 7.8 Hz, 1H), 7.77 (d, J = 8.6 Hz, 2H), 7.66 (t, *J* = 7.5 Hz, 1H), 7.52 (q, *J* = 8.4 Hz, 3H), 7.34 (q, *J* = 6.4 Hz, 2H), 6.90 (s, 1H), 5.01 (s, 2H).

19F NMR (282 MHz, CDCl3) δ -134.99 (m), -142.07 (tt, *J* = 21.1, 7.5 Hz), -157.32 (m).

13C NMR (75 MHz, CDCl3) δ 145.0 (dm, *J* = 265.3 Hz, CF), 138.3 (C), 138.1 (dm, *J* = 269.6 Hz, CF)., 136.9 (C), 134.3 (CH), 131.0 (C), 129.4 (CH), 129.0 (C), 128.8 (CH), 128.1 (CH), 126.4 (CH), 125.2 (CH), 122.0 (CH), 117.0 (CH), 114.4 (CH), 55.3 (CH_2).

HRMS (APCI+) m/z calcd. for $C_{21}H_{13}F_5NO_4S_2$ $[M+H]^+$: 502.0201; found: 502.0205.

1-(1-((perfluorophenyl)sulfonyl)indolin-2-yl)propan-2-one

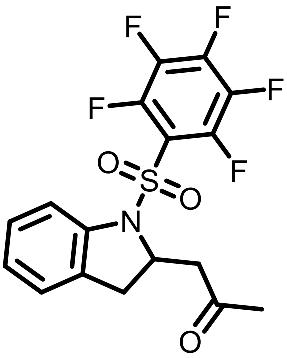

Another 5 mol% Pd(OAc)2, 10 mol% of quinaldine and 1 equiv of copper were added since the begging and let for extra 24h.The residue was purified via silica gel flash chromatography (10% to 20% Et_2O/Hexane) to afford **7ee** as a yellow oil (53.4 mg, 66% yield)

^{1}H NMR (500 MHz, $CDCl_3$) δ 7.46 (d, *J* = 8.0 Hz, 1H), 7.21 – 7.14 (m, 2H), 7.07 (td, *J* = 7.5, 1.1 Hz, 1H), 4.97 (tt, *J* = 10.3, 2.9 Hz, 1H), 3.45 (dd, *J* = 16.8, 9.5 Hz, 1H), 3.24 (dd, *J* = 17.8, 3.2 Hz, 1H), 2.94 (dd, *J* = 17.9, 10.5 Hz, 1H), 2.72 (dd, *J* = 16.8, 2.9 Hz, 1H), 2.19 (s, 3H).

^{19}F NMR (471 MHz, $CDCl_3$) δ -133.63 (m), -144.38 (tt, *J* = 20.8, 6.9 Hz), -157.91 (m).

^{13}C NMR (126 MHz, $CDCl_3$) δ 206.2 (C), 145.1 (dm, *J* = 259.3 Hz, CF), 144.3 (dm, *J* = 262.9 Hz, CF), 139.0 (C), 138.0 (dm, *J* = 258.4 Hz ,CF), 130.8 (C), 128.2 (CH), 126.0 (CH), 125.5 (CH), 115.5 (CH), 114.8 (m, C) 59.4 (CH), 50.6 (CH_2), 35.32 (CH_2), 30.6 (CH_3).

HRMS (APCI+) *m/z* calcd. for $C_{17}H_{13}F_5NO_3S$ $[M+H]^+$: 406.0531; found: 406.0539.

Compound **7ee** is proposed to be formed through C-H olefination, followed by a Michael addition.

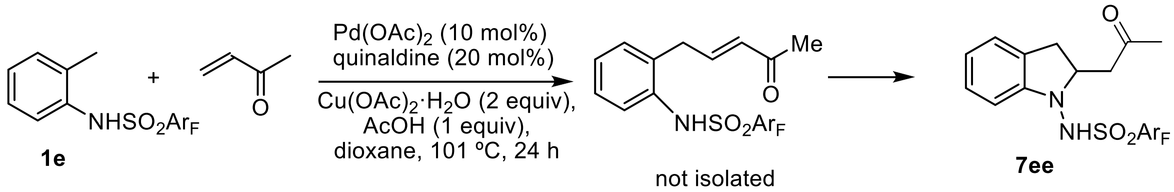

1e **7ee**

3.6 Pd-*I* isolation and reaction with diene

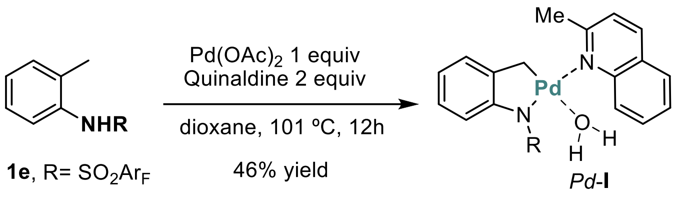

2,3,4,5,6-pentafluoro-*N*-(*o*-tolyl)benzenesulfonamide **1e** (67.5 mg, 0.20 mmol, 1.0 equiv), $Pd(OAc)_2$ (44.9 mg, 0.20 mmol 1.0 equiv) and quinaldine (60.3 mg, 2.0 equiv) were weighed and added into a Schlenk flask under air. Then, 1,4-dioxane (4.0 mL, 0.05 M), was added. The mixture was refluxed at 101 ºC in a Schlenk heating block for 12h using a cold finger setup. After cooling to rt, it was concentrated under reduced pressure and the resulting residue was purified by flash column chromatography (AcOEt/Pentane 30:70 to AcOEt/Pentane/DCM 6:4:2) to afford the corresponding product ***Pd-I*** as a white yellowish solid (60 mg, 46% yield).

Pd-I

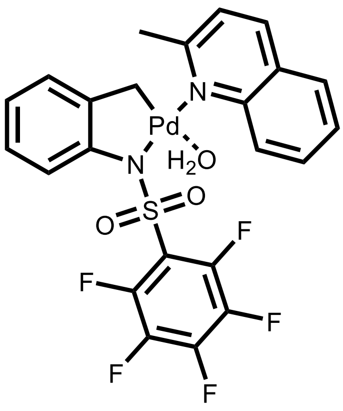

^{1}H NMR (500 MHz, $CDCl_3$) δ 9.92 (d, *J* = 8.6 Hz, 1H), 8.24 (d, *J* = 8.4 Hz, 1H), 7.87 (m, 2H), 7.61 (t, *J* = 7.5 Hz, 1H), 7.50 (dd, *J* = 8.5, 3.6 Hz, 2H), 6.98 (t, *J* = 7.8 Hz, 1H), 6.82 (d, *J* = 7.4 Hz, 1H), 6.69 (t, *J* = 7.3 Hz, 1H), 3.55 (s, 3H), 2.96 (s, 2H), 2.14 (s, 2H).

^{19}F NMR (471 MHz, $CDCl_3$) δ -134.18 (m), -149.89 (tt, *J* = 21.5, 5.5 Hz), -160.43 (m).

^{13}C NMR (126 MHz, $CDCl_3$) δ 163.3 (C), 148.8 (C), 147.9 (C), 140.5 (C), 139.0 (CH), 131.2 (CH), 129.9 (CH), 128.2 (CH), 127.7 (C), 127.5 (CH), 126.3 (CH), 126.2 (CH), 123.3 (CH), 121.5 (CH), 118.8 (CH), 28.9 (CH_3), 22.9 (CH_2).

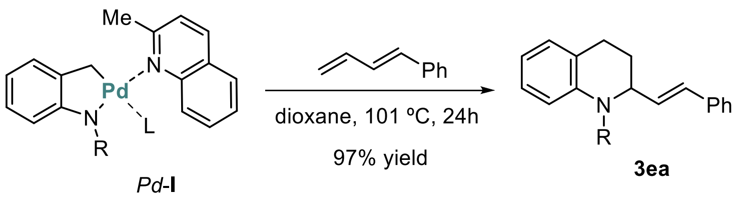

Pd-**I** (20 mg, 0.033 mmol, 1 equiv) was weighed and added into a Schlenk flask under air. Then, 1,4-dioxane (2.0 mL, 0.05 M), and the diene **2a** (10 µL, 0.066, 2 equiv) were added. The mixture was refluxed at 101 ºC in a Schlenk heating block for 24 h using a cold finger setup. After cooling to rt, the reaction mixture was diluted with DCM and filtered through a Fluorisil-silica gel pad, washing the flask and the pad with more DCM (x3). The filtrate was concentrated under reduced pressure and the resulting residue was

purified by flash column chromatography (AcOEt/Pentane 5:95 to 30:70) to afford the corresponding product **3ea** in a 97% yield

3.7 Deprotection of sulfonamides 3ea and 7eb

Deprotection was performed following and adapted version of a reported method. [7]

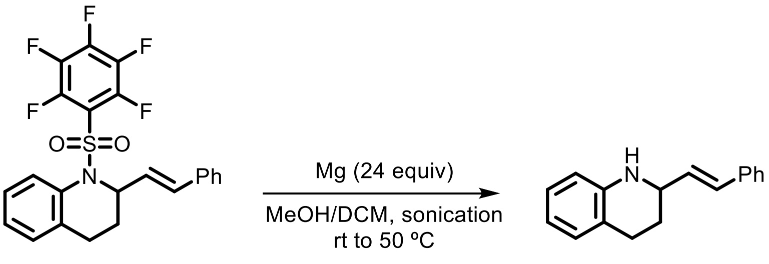

3ea **4**

Magnesium turnings from older source were washed with diluted aqueous muriatic acid till silver like appearance occurred (around 10 minutes), filtered, washed with ethanol and finally with ether. Dried at 20 mbar at 50°C for 30 minutes.

To a dry Schlenk tube equipped with a magnetic stir bar, was added **3ea** (40 mg , 0,086 mmol, 1 equiv) and Mg-powder (50.1 mg, 24 equiv.). The tube was capped with a septum, evacuated and refilled with Ar for 3 times. Then, absolute MeOH (1.7 mL) and DCM (0.2 ml) were added and the reaction was sonicated at with gradual heating (from rt to 50 ºC) until no more starting material remained monitored by TLC (around 2 h). The solution was cooled to 0 °C and slowly quenched with saturated NH_4Cl. The aqueous phases were extracted with dichloromethane (3 × 10 mL). The combined organic phases were washed with saturated brine, then dried over anhydrous $MgSO_4$, concentrated under reduced pressure. The residue was purified by silica gel column chromatography (Pentane/Eter = 9:1) to afford **4** (12.0 mg, 60%) as light yellow oil.

(*E*)-2-styryl-1,2,3,4-tetrahydroquinoline

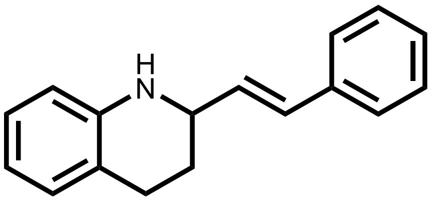

^{1}H NMR (500 MHz, $CDCl_3$) δ 7.41 (m, 2H), 7.35 (m, 2H), 7.27 (m, 1H), 7.02 (m, 2H), 6.67 (td, *J* = 7.4, 1.2 Hz, 1H), 6.62 (dd, *J* = 15.8, 1.0 Hz, 1H), 6.56 (dd, *J* = 7.9, 1.2 Hz, 1H), 6.29 (dd, *J* = 15.8, 7.2 Hz, 1H), 4.05 (dddd, *J* = 8.6, 7.2, 3.5, 1.1 Hz, 1H), 3.93 (s, 1H), 2.85 (m, 2H), 2.10 (dtd, *J* = 12.9, 5.3, 3.4 Hz, 1H), 1.89 (m, 1H).

^{13}C NMR (126 MHz, $CDCl_3$) δ 144.2 (C), 137.0 (C), 132.3 (CH), 130.5 (CH), 129.4 (CH), 128.7 (CH), 127.7 (CH), 127.0 (CH), 126.5 (CH), 121.1 (C), 117.3 (CH), 114.3 (CH), 54.2 (CH), 28.7 (CH_2), 25.9 (CH_2).

HRMS (APCI+) *m/z* calcd. for $C_{17}H_{18}N$ $[M+H]^+$: 236.1434; found: 236.1419.

Methyl 2-(1*H*-indol-2-yl)acetate

8 was obtained as a pale yellow oil (29.7 mg, 70%) from 94 mg of **7eb**. Spectral data recorded was in agreement with the previously reported. [8]

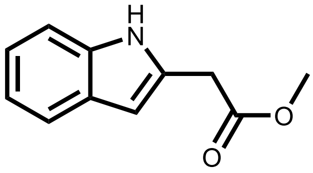

^{1}H NMR (500 MHz, $CDCl_3$) δ 8.65 (s, 1H), 7.57 (dt, *J* = 7.8, 0.9 Hz, 1H), 7.35 (m, 1H), 7.18 (ddd, *J* = 8.2, 7.1, 1.2 Hz, 1H), 7.11 (ddd, *J* = 8.0, 7.1, 1.0 Hz, 1H), 6.38 (dt, *J* = 1.7, 0.9 Hz, 1H), 3.85 (d, *J* = 0.7 Hz, 2H), 3.77 (s, 3H).

^{13}C NMR (126 MHz, $CDCl_3$) δ 171.1 (C), 136.5 (C), 130.5 (C), 128.3 (C), 121.9 (CH), 120.36 (CH), 120.0 (CH), 110.9 (CH), 102.0 (CH), 52.5 (CH_3), 33.9 (CH_2).

HRMS (APCI+) *m/z* calcd. for $C_{11}H_{12}NO_2$ $[M+H]^+$: 190.0863; found: 190.0863.

3.8 1 mmol scale

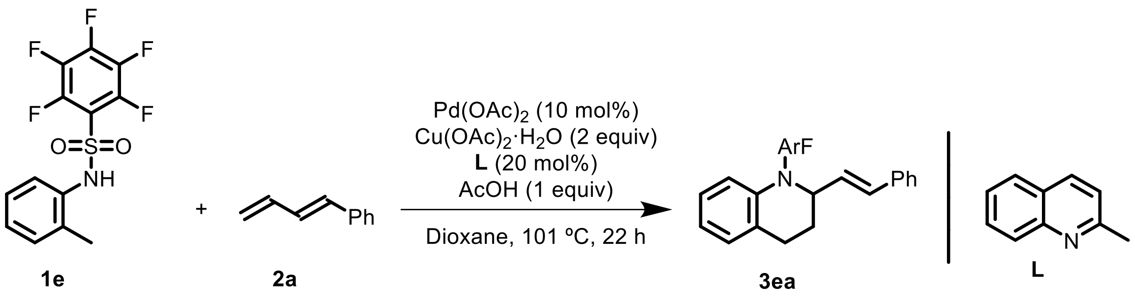

2,3,4,5,6-pentafluoro-*N*-(*o*-tolyl)benzenesulfonamide **1e** (337.3 mg, 1 mmol, 1.0 equiv), $Pd(OAc)_2$ (22.5 mg, 10 mol%), $Cu(OAc)_2{\cdot}H_2O$ (399.3 mg, 2.0 equiv) were weighed and added into a Schlenk flask under air. Then, 1,4-dioxane (20 mL, 0.05 M), quinaldine (28 µL, 20 mol%), AcOH (57 µL, 1 equiv) and the diene **2a** (274.1 mg, 2 mmol, 2.0 equiv) were added. The mixture was refluxed at 101 ºC in a Schlenk heating block for 20-24 h using a cold finger setup. After cooling to rt, the reaction mixture was diluted with DCM and filtered through a Fluorisil-silica gel pad, washing the flask and the pad with more DCM (x3). The filtrate was concentrated under reduced pressure and the resulting residue was purified by flash column chromatography (AcOEt/Pentane 5:95 to 30:70) to afford the corresponding product **3ea** with a 95% yield (441.8 mg)

4. Crystallographic Data

X-Ray diffraction data for compound 3ea

Crystal of **3ea** was obtained after a crystallization process in an rmn tube by dissolving the product in the right amount of dichloromethane and adding heptane from the top slowly to leave a two-phase system. The mixture was left overnight until crystals grew.

Structure of the cycloadduct was determined by X-ray crystallography. The structure was deposited in the Cambridge Structural Database; **Deposition Number: 2364146.**

ORTEP plot is given with a 50% probability level

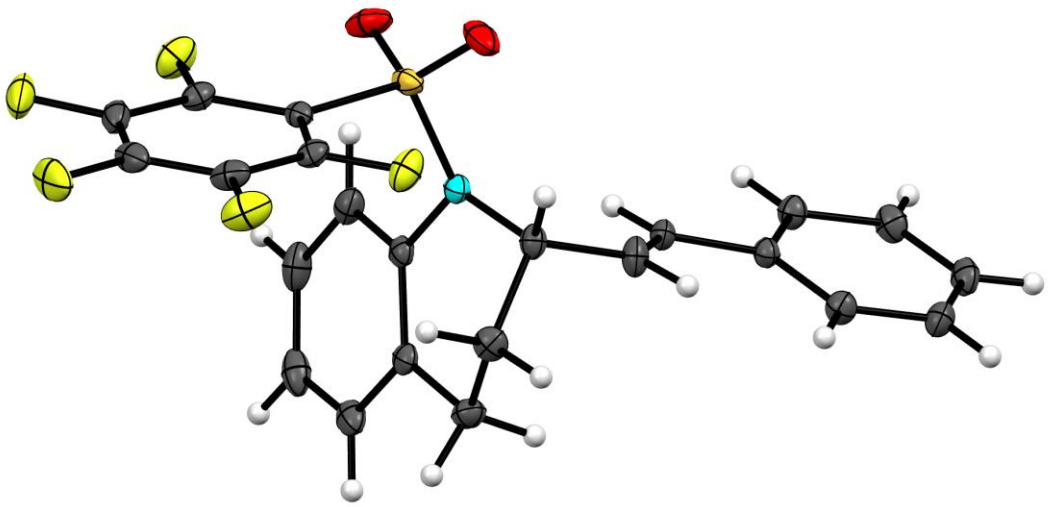

Empirical formula	$C_{23}H_{16}F_5NO_2S$
Formula weight	465.43
Temperature/K	100(2)
Crystal system	monoclinic
Space group	Cc
a/Å	6.4054(3)
b/Å	22.1123(13)
c/Å	14.0622(10)
α/°	90
β/°	98.605(3)
γ/°	90
Volume/$Å^3$	1969.3(2)
Z	4
ρ_{calc}g/cm^3	1.57
μ/mm^{-1}	0.233
F(000)	952
Crystal size/mm^3	0.423 × 0.276 × 0.116
Radiation	MoK\a (λ = 0.71073)
2Θ range for data collection/°	2.35 to 28.28
Reflections collected	29930
Independent reflections	29930 [R_{int} = 0.0529, R_{sigma} = 0.031]
Data/restrains/parameters	29930/2/289
Goodness-of-fit on F^2	1.045
Final R indexes [I>=2σ (I)] R1	R_1 = 0.0285, wR_2 = 0.0747
Final R indexes [all data] R1	R_1 = 0.0290, wR_2 = 0.0753
Largest diff. peak/hole / e $Å^{-3}$	0.286/-0.27

X-Ray diffraction data for compound *Pd*-I

Crystal of *Pd-I* was obtained after a crystallization process in an rmn tube by dissolving the product in the right amount of dichloromethane and adding heptane from the top slowly to leave a two-phase system. The mixture was left overnight until crystals grew.

Structure of the cycloadduct was determined by X-ray crystallography. The structure was deposited in the Cambridge Structural Database; **Deposition Number: 2364145.**

ORTEP plot is given with a 50 % probability.

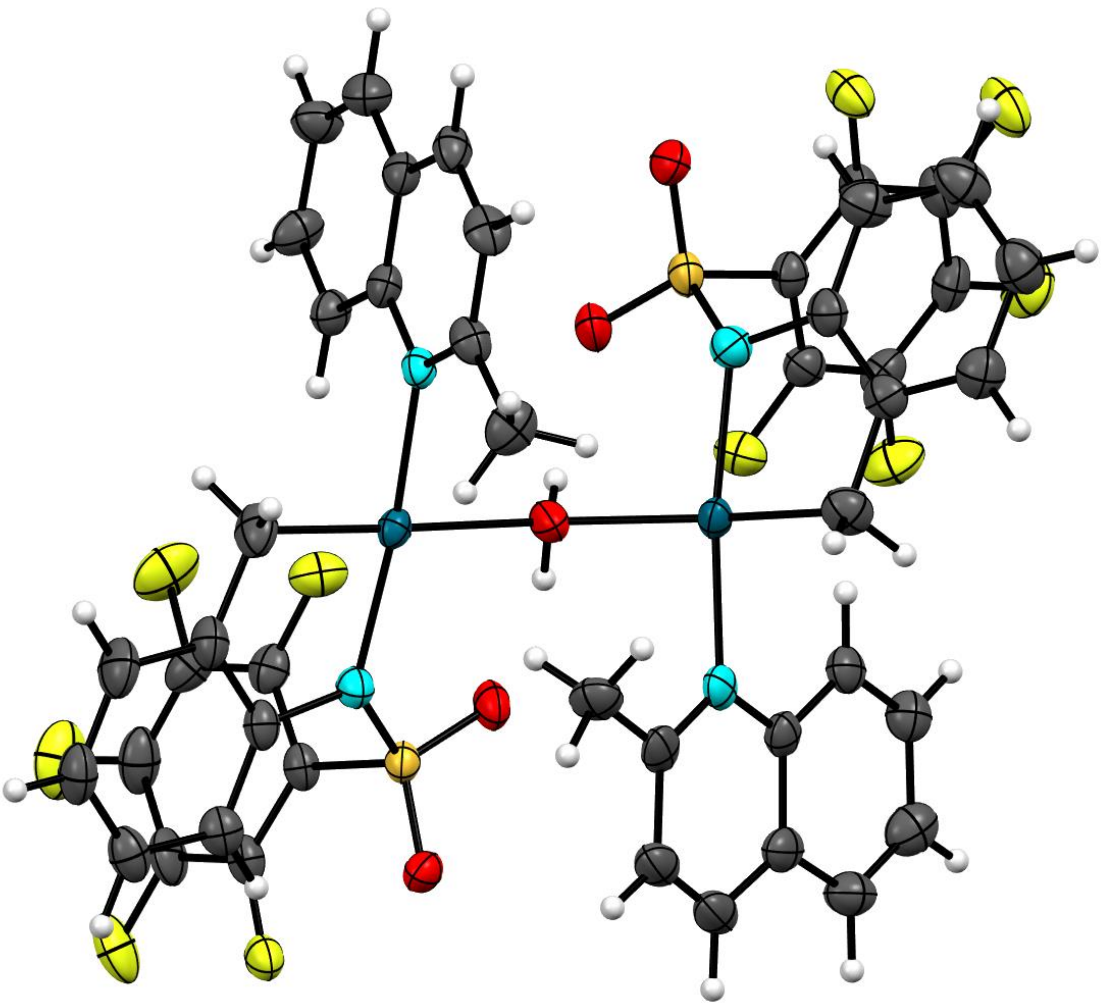

Empirical formula	$C_{46}H_{32}F_{10}N_4O_5Pd_2S_2$
Formula weight	1187.67
Temperature/K	100(2)
Crystal system	monoclinic
Space group	P 2_1
a/Å	13.1044(6)
b/Å	9.3261(5)
c/Å	18.5269(9)
α/°	90
β/°	107.051(2)
γ/°	90
Volume/Å^3	2164.70(19)
Z	2
ρ_{calc}g/cm^3	1.822
μ/mm^{-1}	1.025
F(000)	1180
Crystal size/mm^3	0.269 × 0.108 × 0.025
Radiation	MoK\a (λ = 0.71073)
2Θ range for data collection/°	2.30 to 31.11
Reflections collected	91941
Independent reflections	91941 [R_{int} = 0.0586, R_{sigma} = 0.0404]
Data/restrains/parameters	91941/501/ 916
Goodness-of-fit on F^2	1.099
Final R indexes [I>=2σ (I)] R1	R_1 = 0.0444, wR_2 = 0.0865
Final R indexes [all data] R1	R_1 = 0.0557, wR_2 = 0.0913
Largest diff. peak/hole / e Å^{-3}	1.237/-0.976

5. References

1 Chaturvedi J. et al. *J. Am. Chem. Soc.* **2021**, 143, 7604–7611

2 Yasukawa N. et al., *Green Chem*., **2018**, 20, 1213-1217

3 Hongyuan B. et al., *Macromolecules,* **2021**, 54, 1183–1191

4 Fernandes A. *J. Org. Chem.* **2023**, 88, 10339–10354

5 Zhao, D. et al.. *Chem. Sci.* **2014**, 5, 2869-2873.

6 Yu J-Q. et al., *J. Am. Chem. Soc*. **2023**, 145, 13003–13007

7 Zhang, Z. F. et al., *Tetrahedron,* **2021**, 94.

8 Touge, T. et al, *J. Am. Chem. Soc.* **2016**, 138, 11299–11305

6. NMR Spectra

^{1}H NMR (500 MHz, $CDCl_3$)

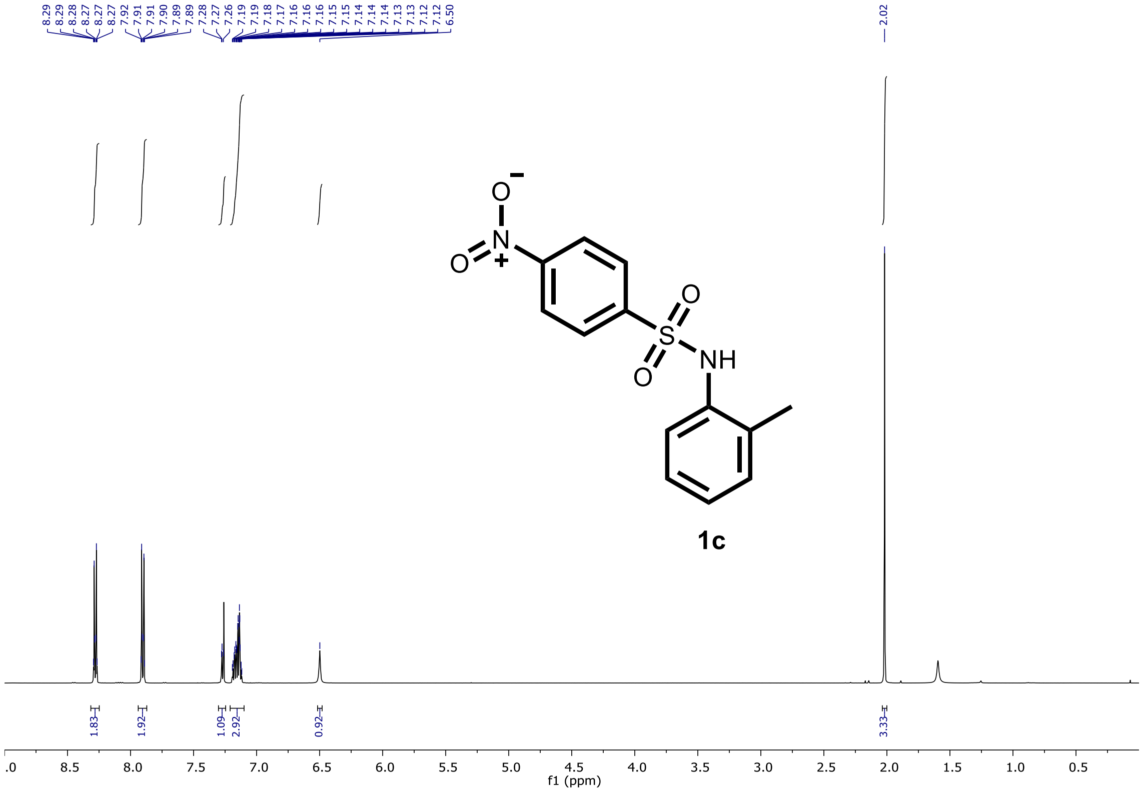

DEPT-135

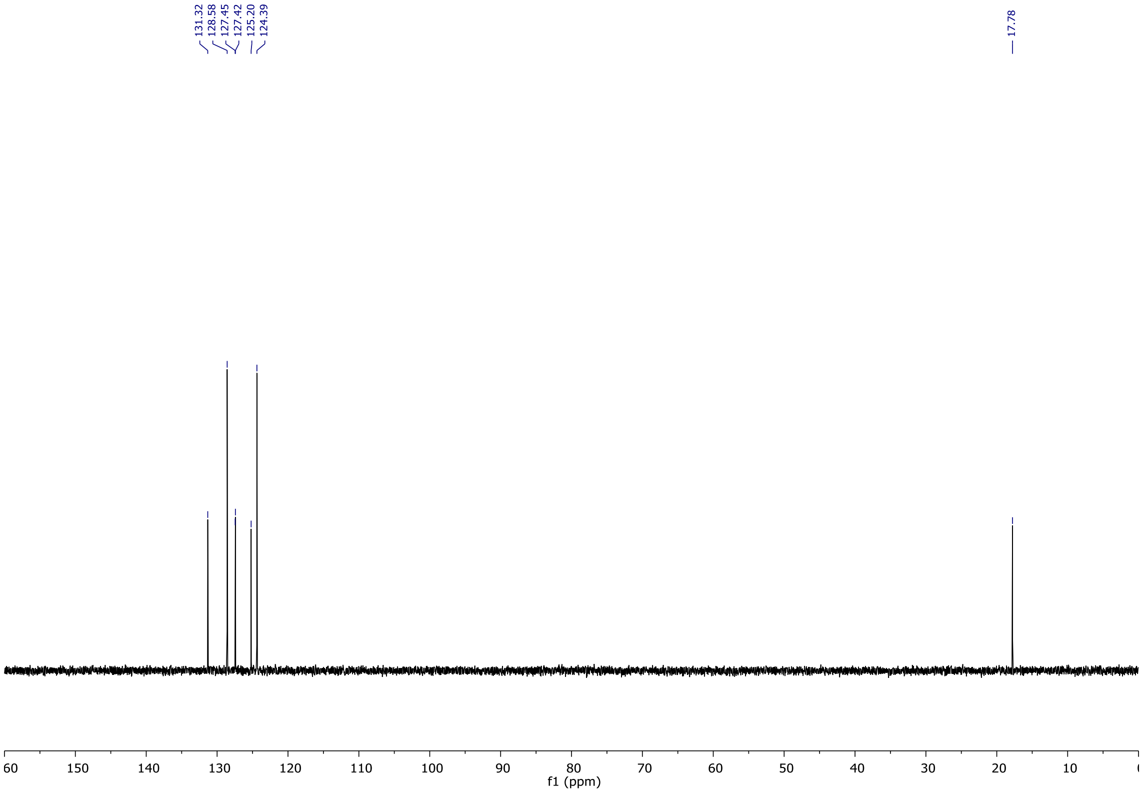

^{13}C NMR (126 MHz, CDCl$_3$)

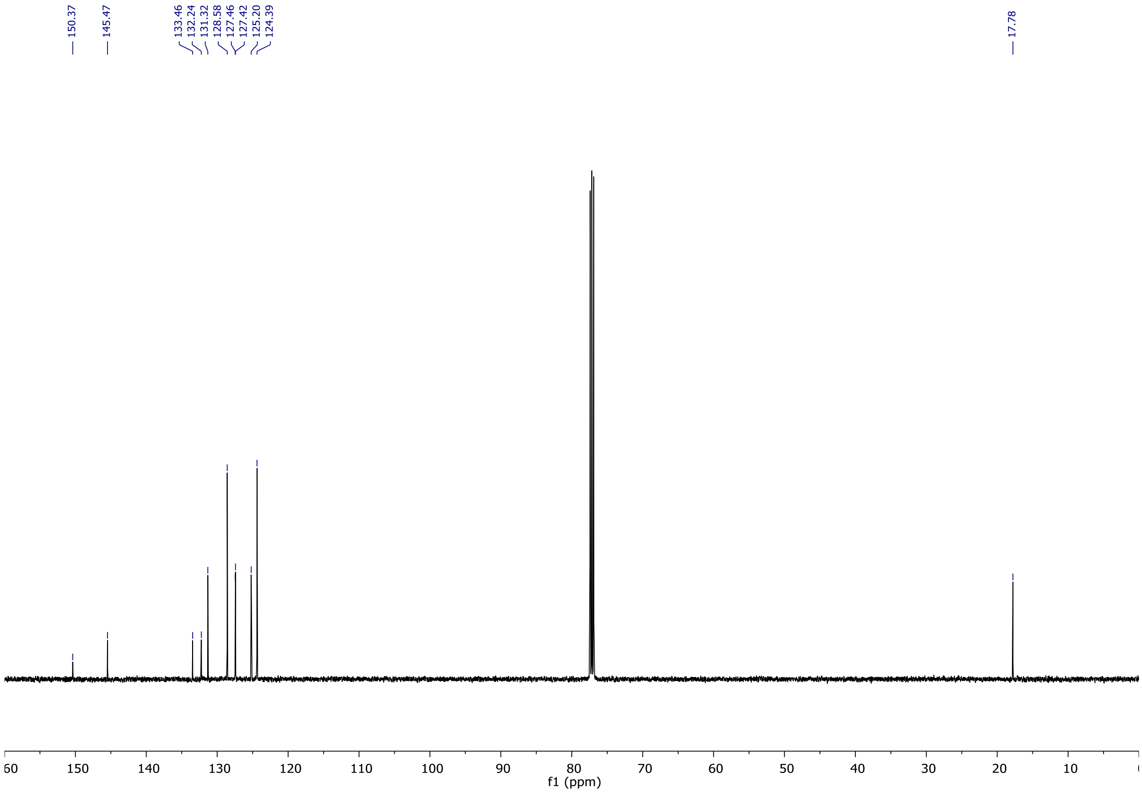

^{1}H NMR (300 MHz, CDCl$_3$)

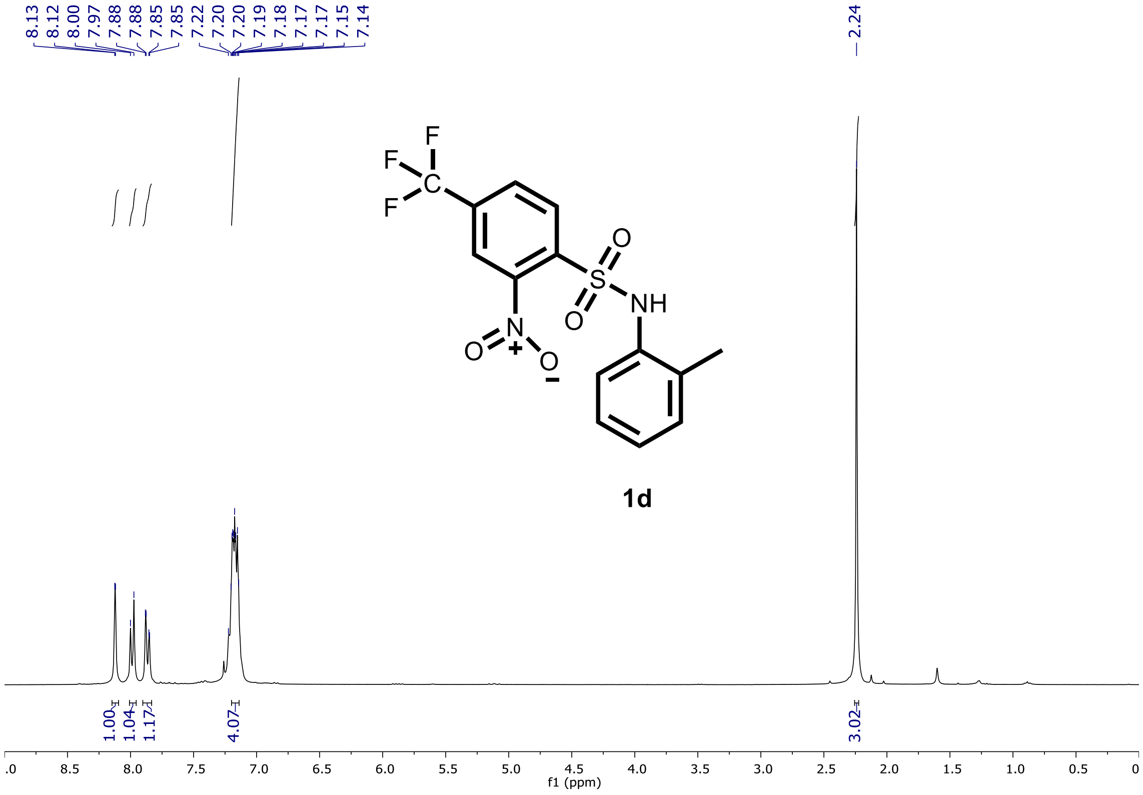

^{19}F NMR (282 MHz, CDCl$_3$)

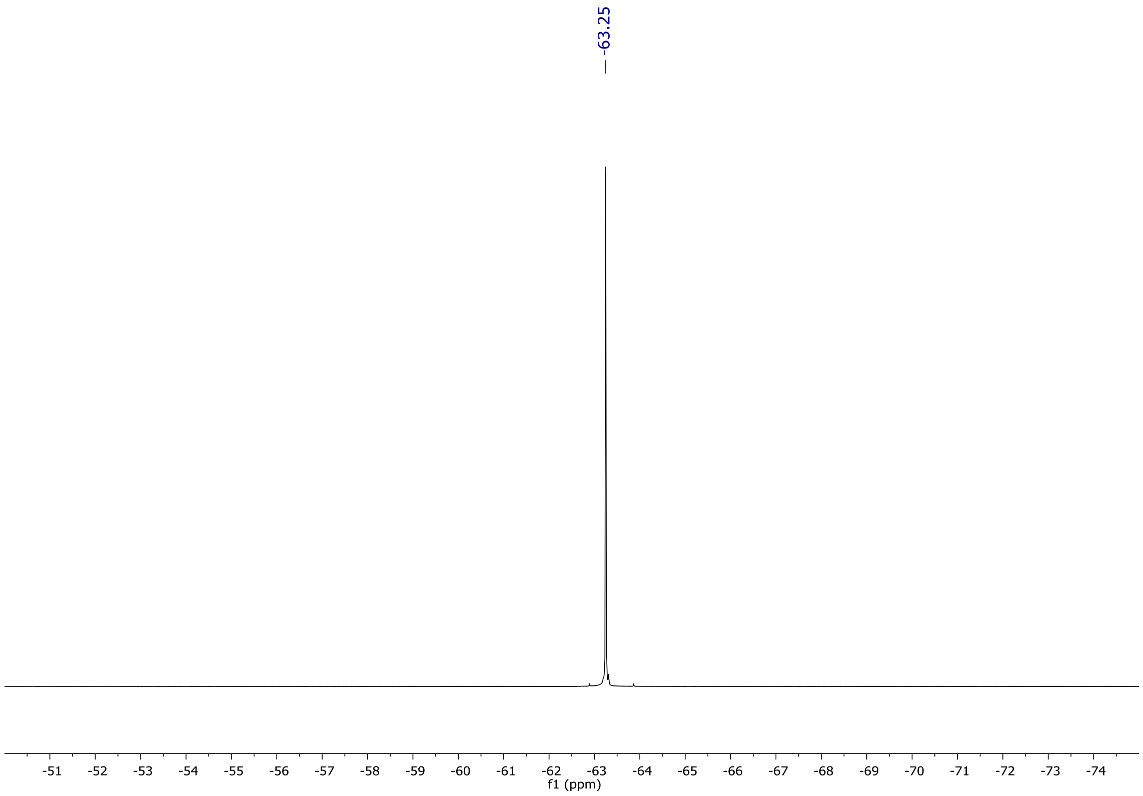

DEPT-135

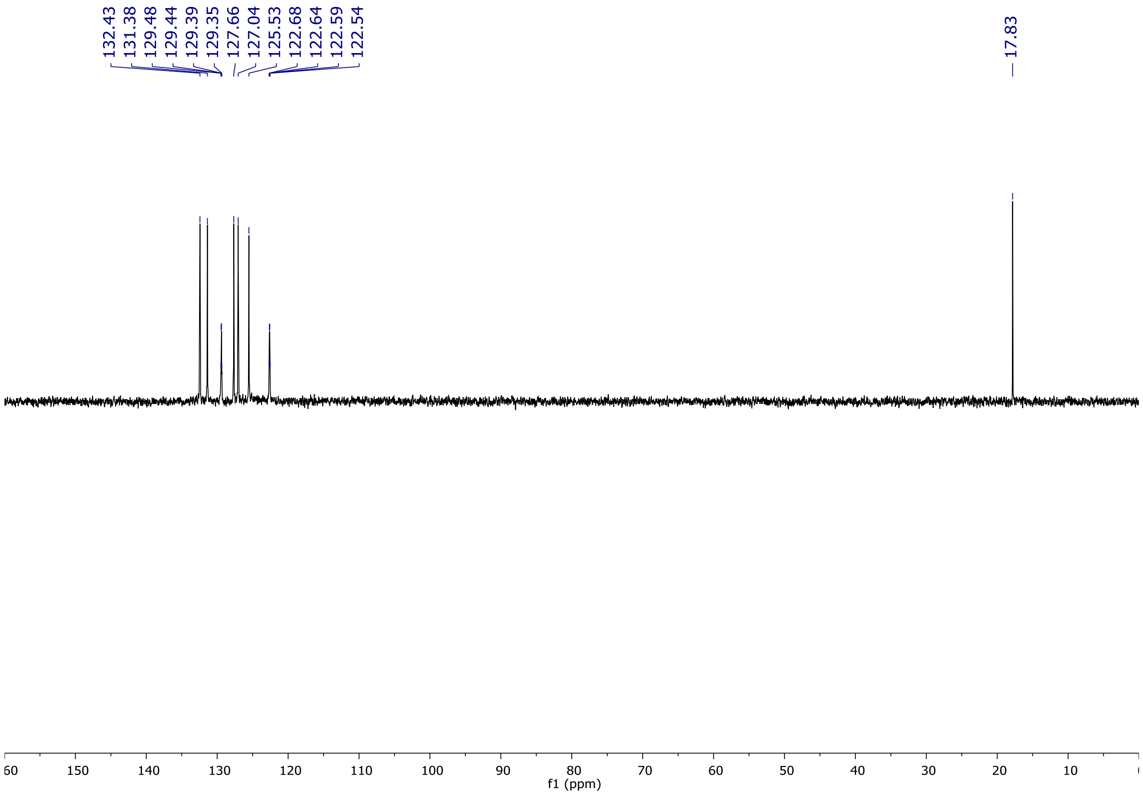

^{13}C NMR (75 MHz, $CDCl_3$)

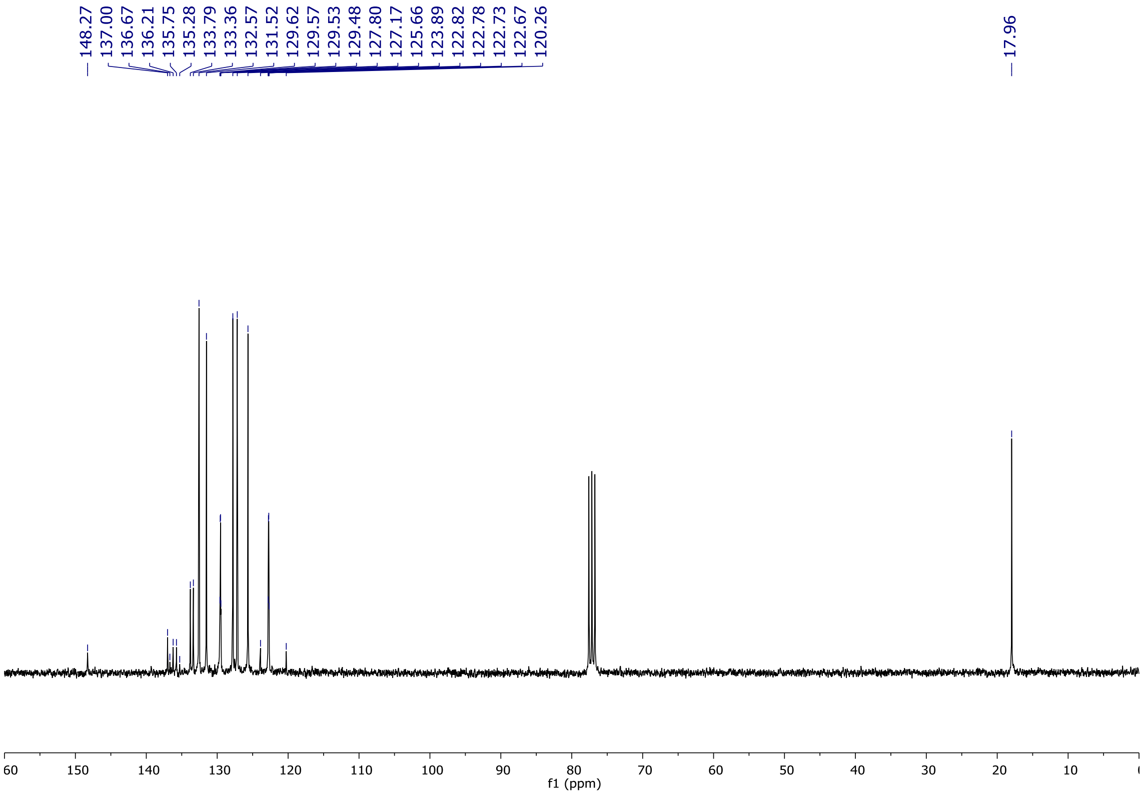

^{1}H NMR (500 MHz, CDCl$_3$)

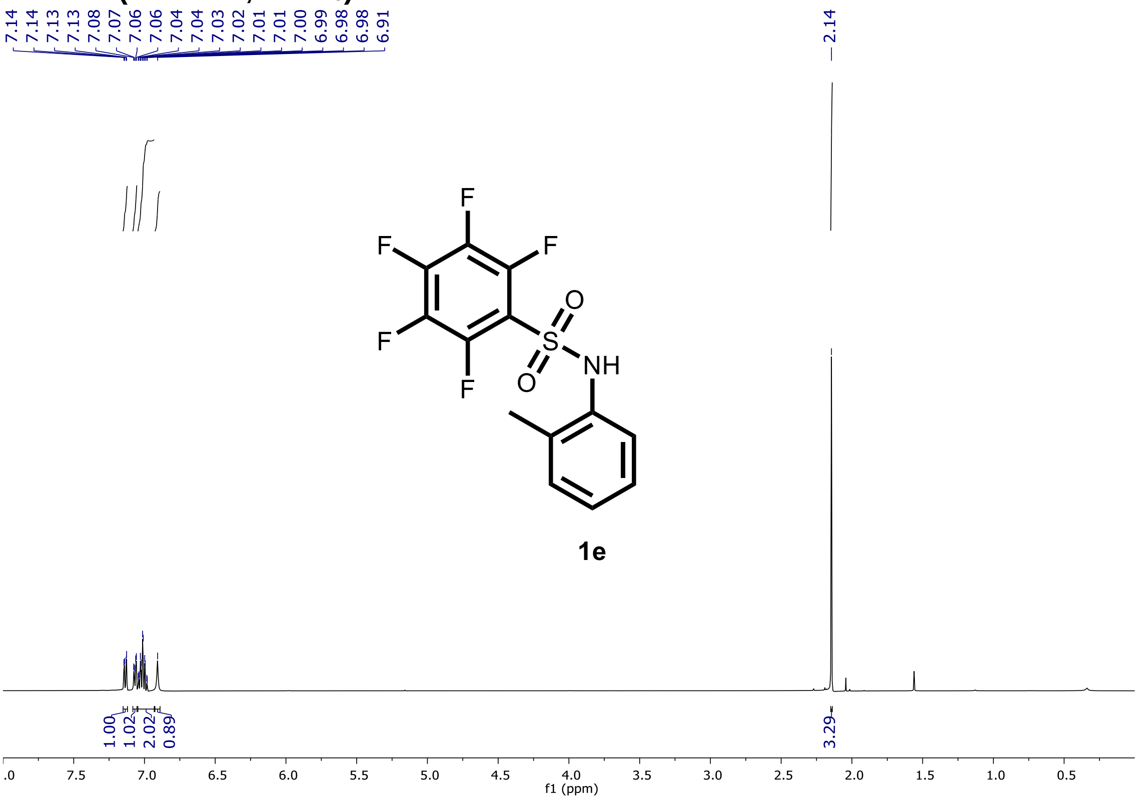

^{19}F NMR (471 MHz, CDCl$_3$)

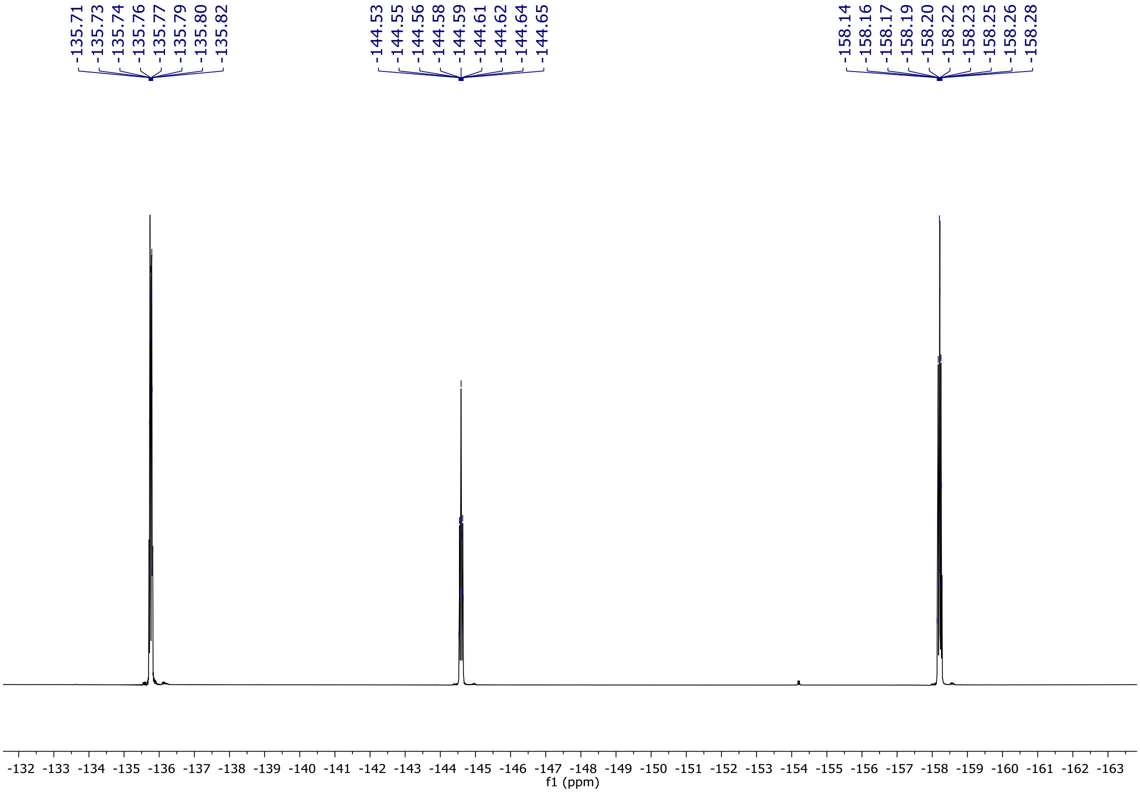

DEPT-135

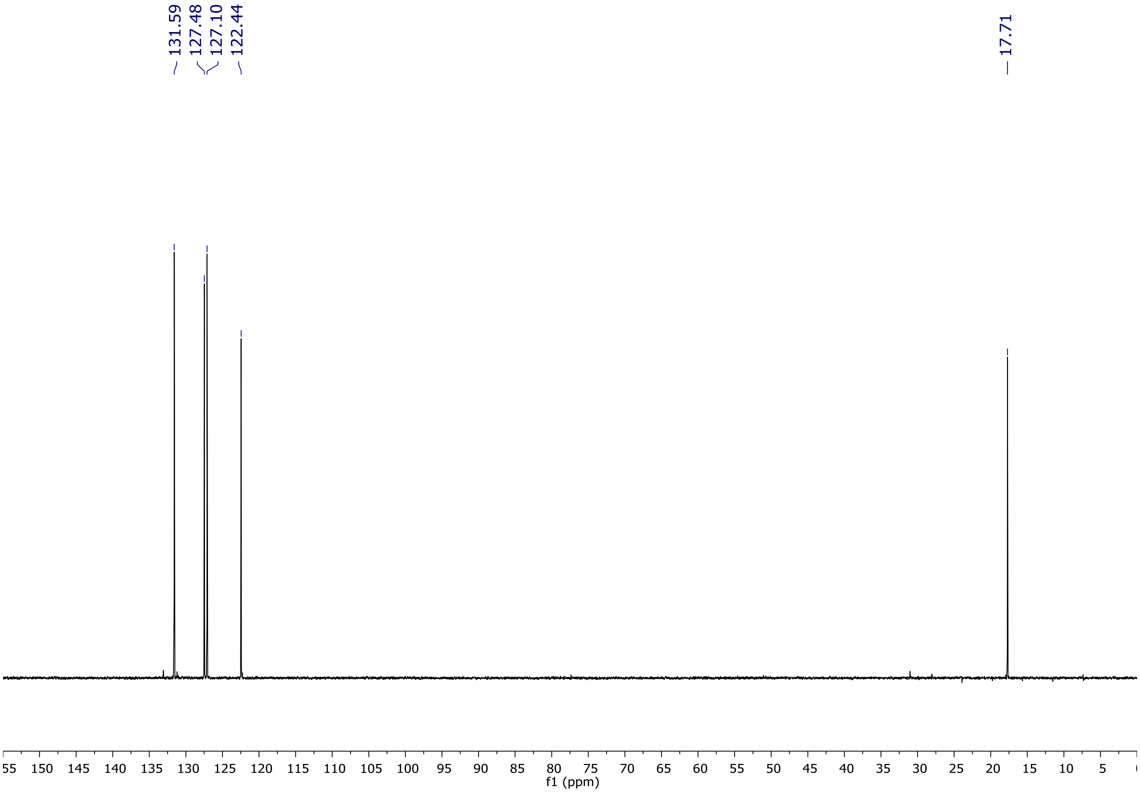

^{13}C NMR (126 MHz, $CDCl_3$)

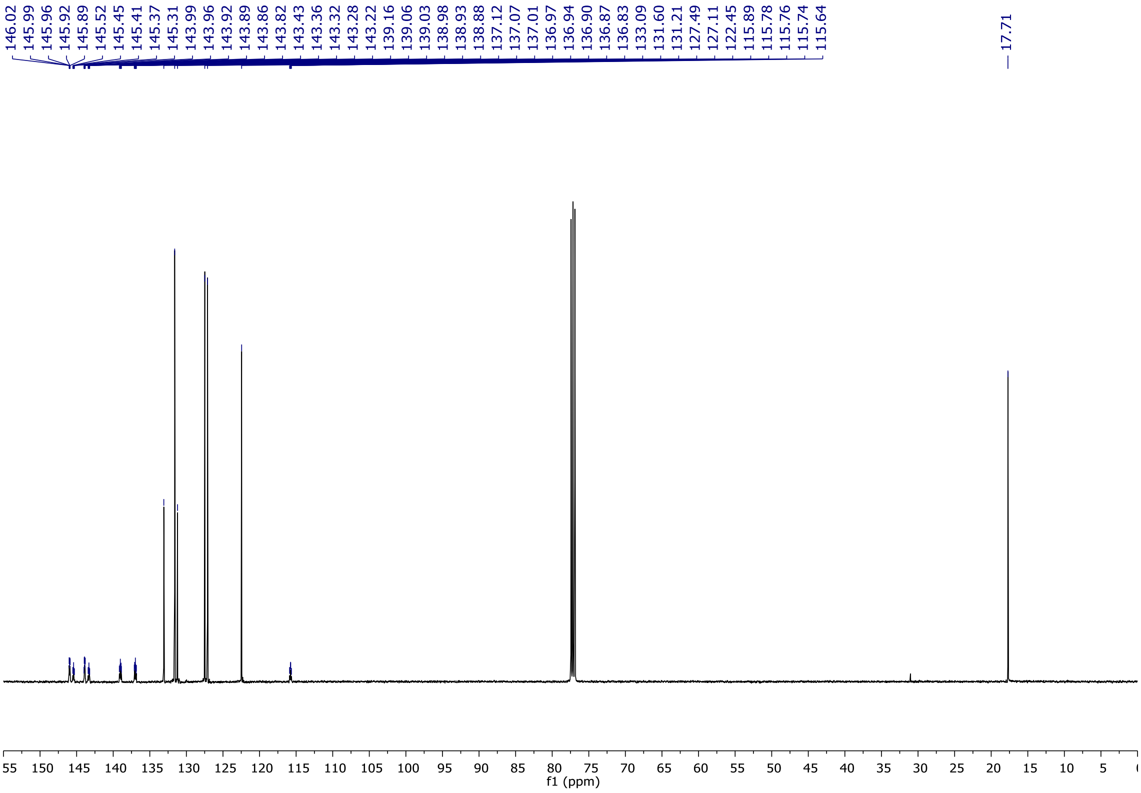

^{1}H NMR (500 MHz, $CDCl_3$)

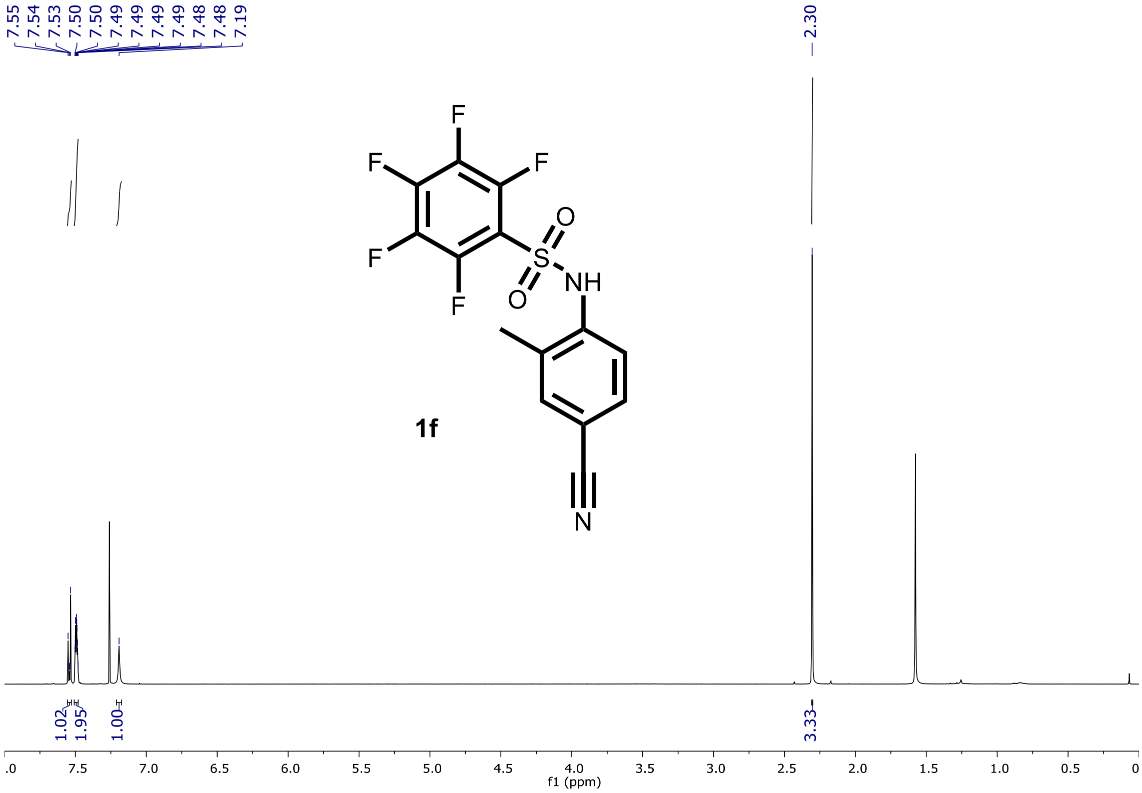

^{19}F NMR (471 MHz, $CDCl_3$)

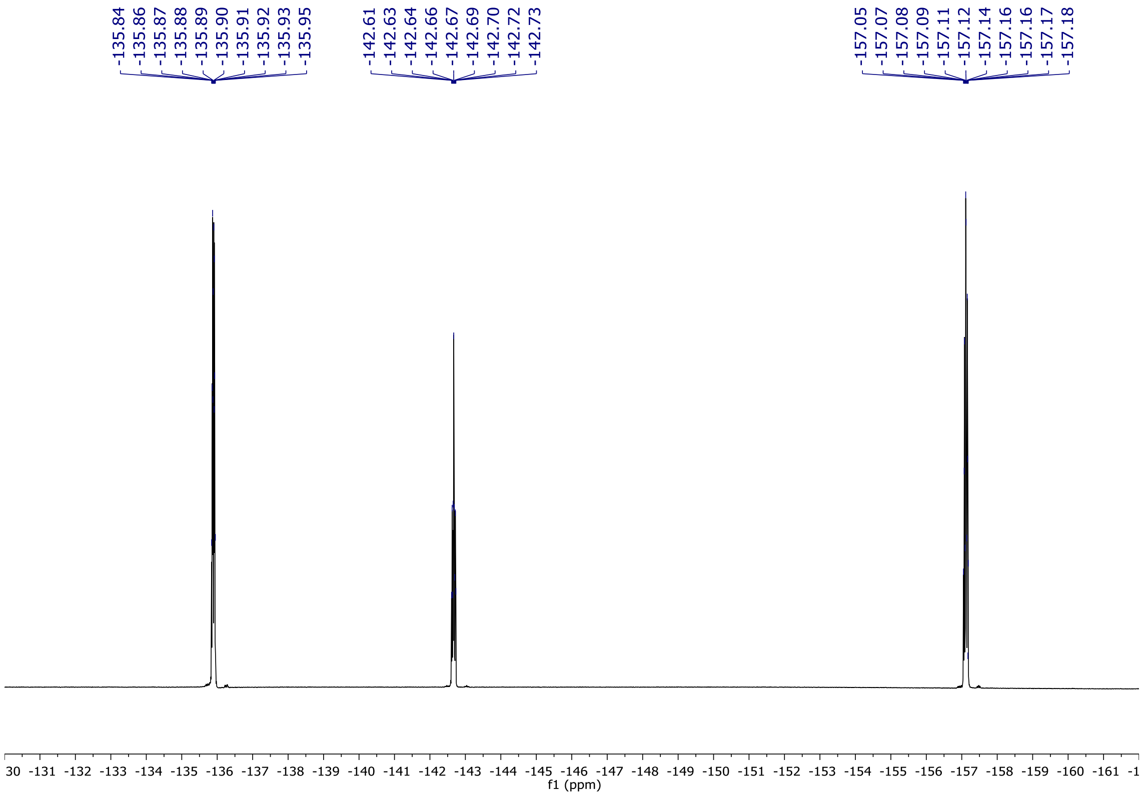

DEPT-135

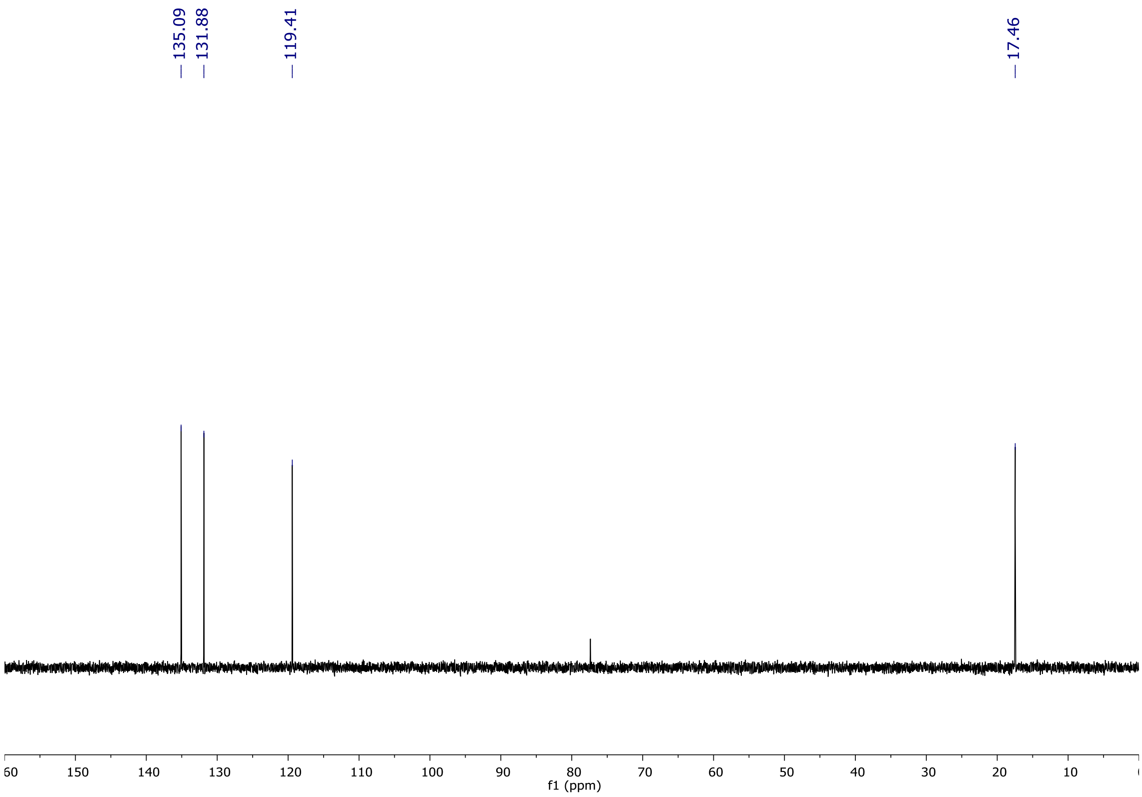

^{13}C NMR (126 MHz, $CDCl_3$)

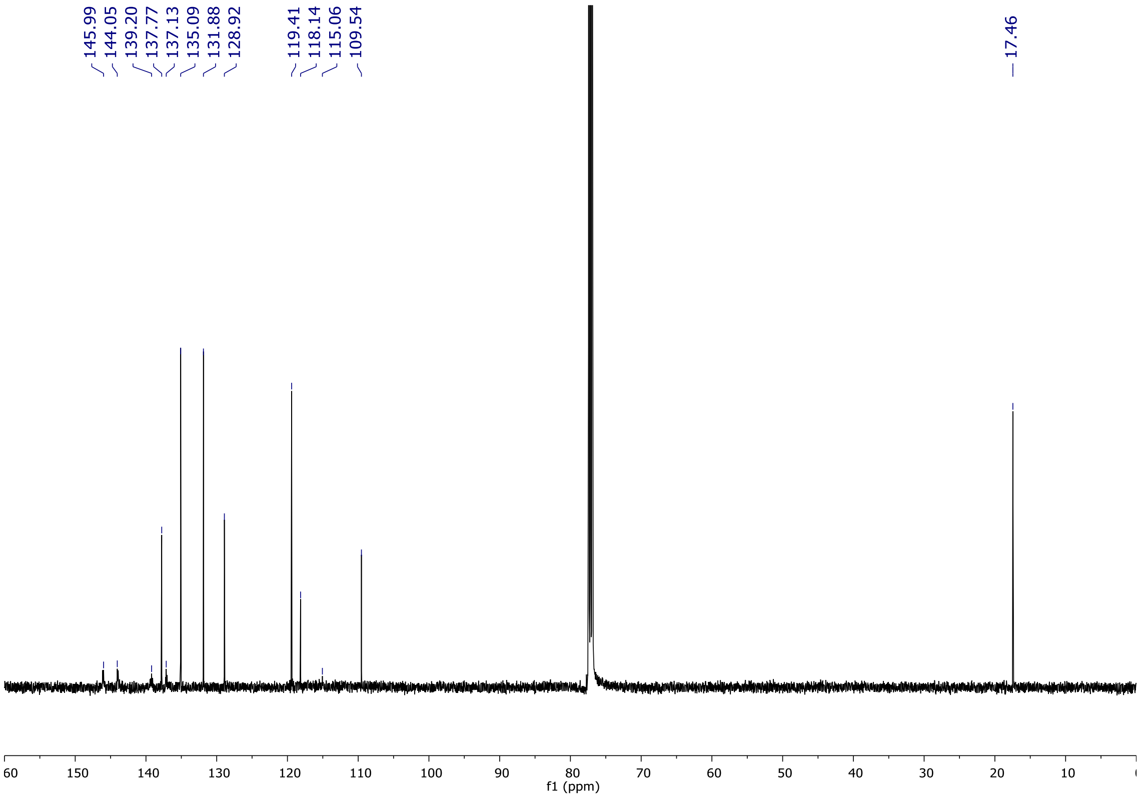

^{1}H NMR (300 MHz, $CDCl_3$)

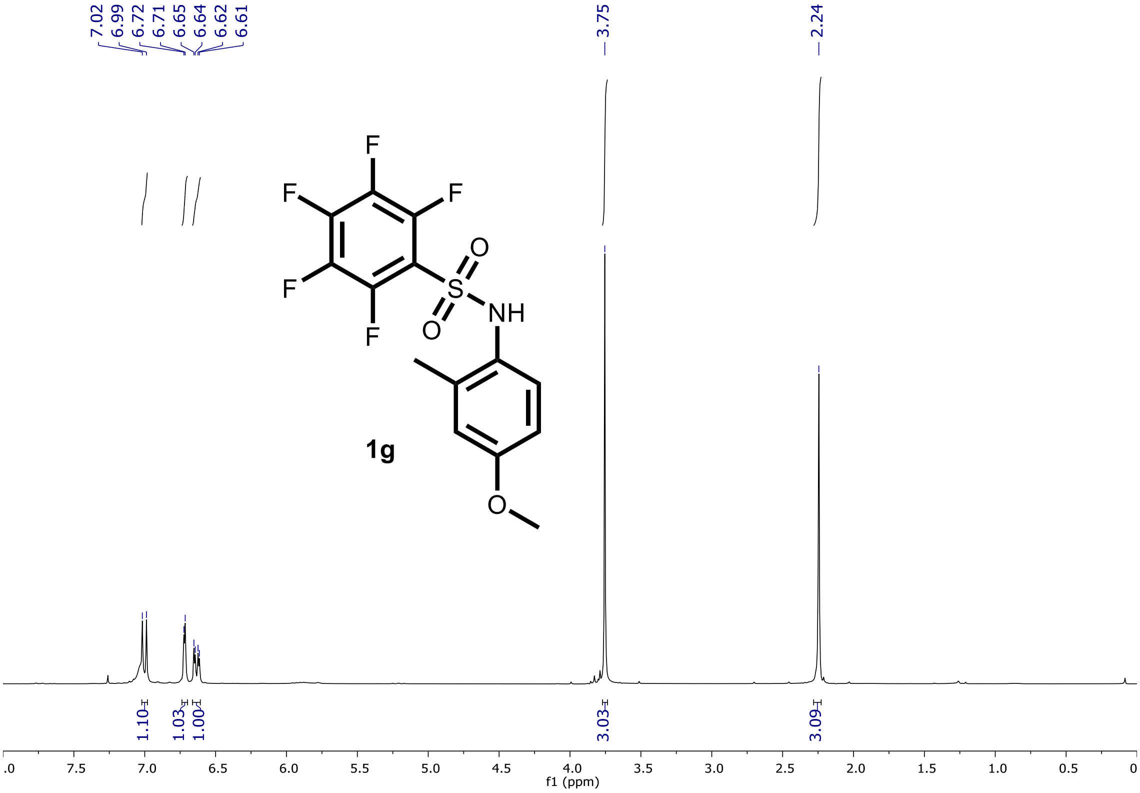

^{19}F NMR (282 MHz, $CDCl_3$)

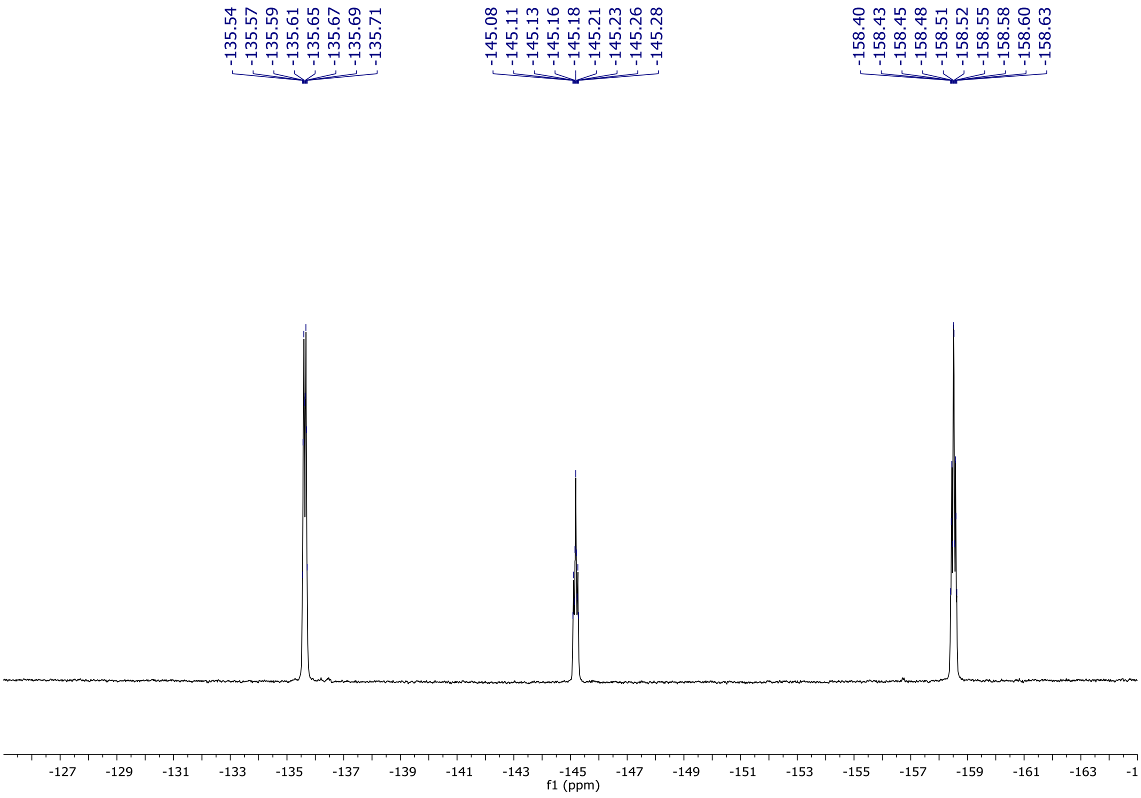

DEPT-135

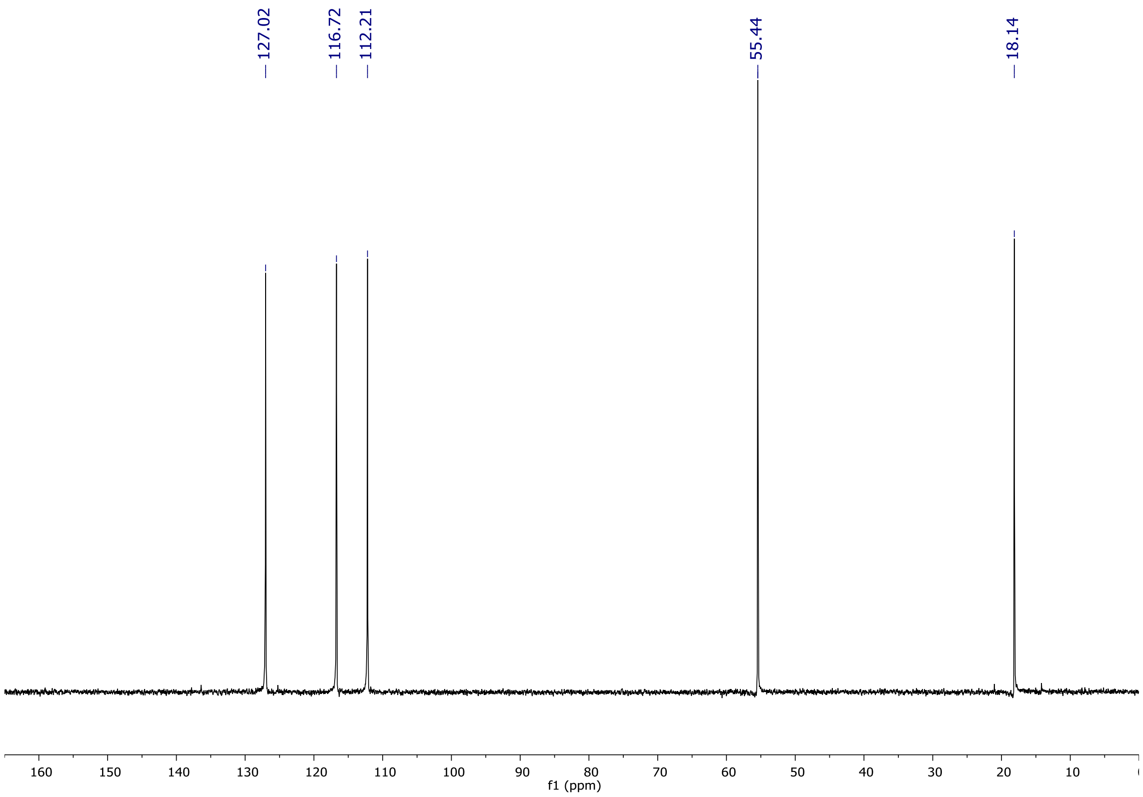

^{13}C NMR (75 MHz, $CDCl_3$)

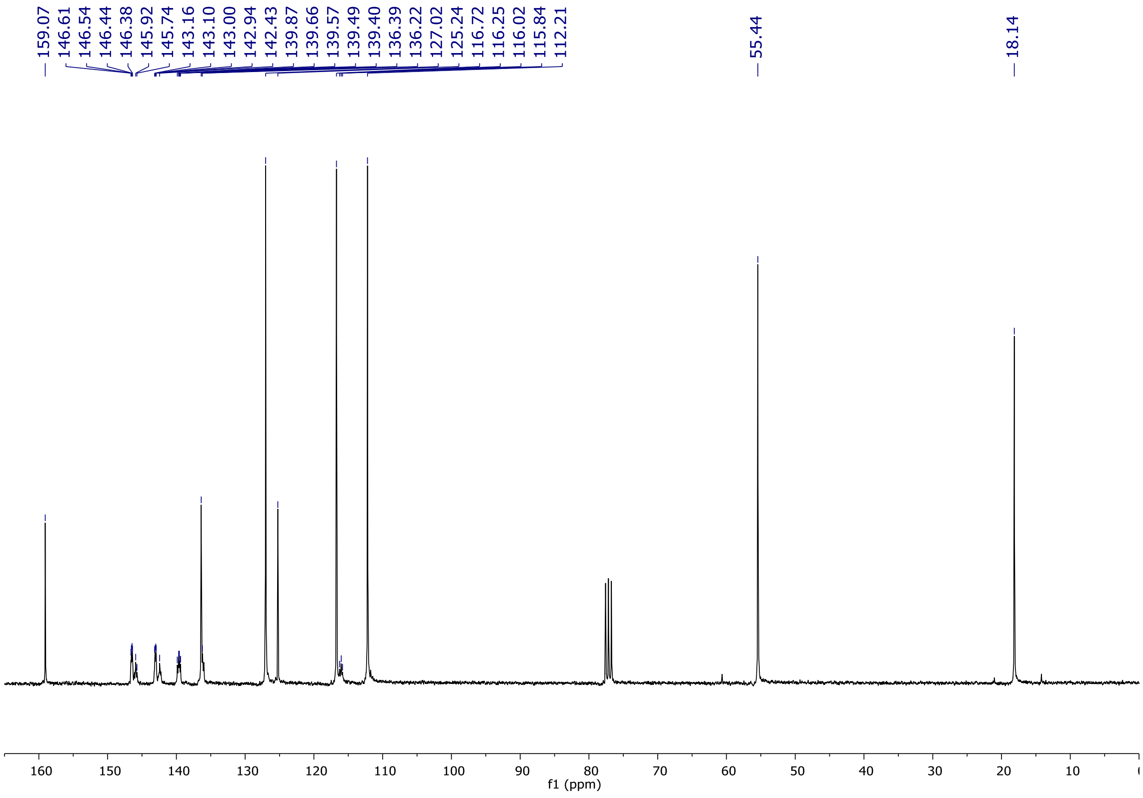

^1H NMR (500 MHz, $CDCl_3$)

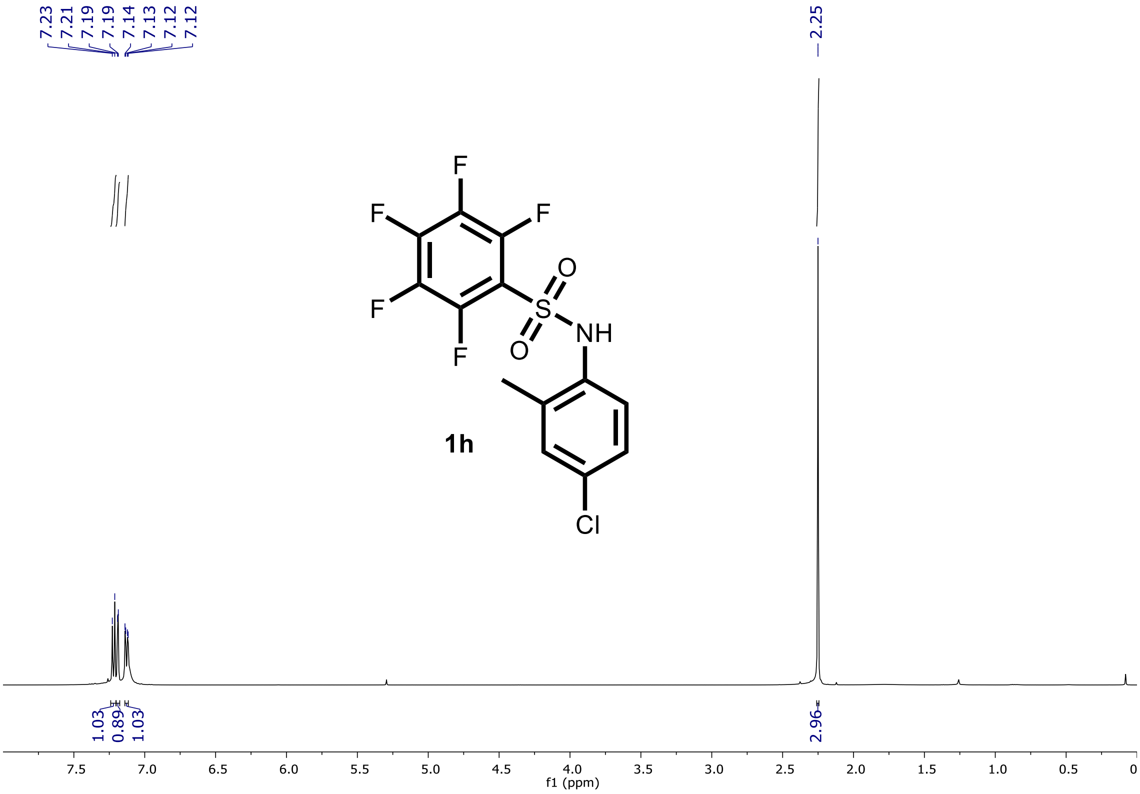

^{19}F NMR (471 MHz, $CDCl_3$)

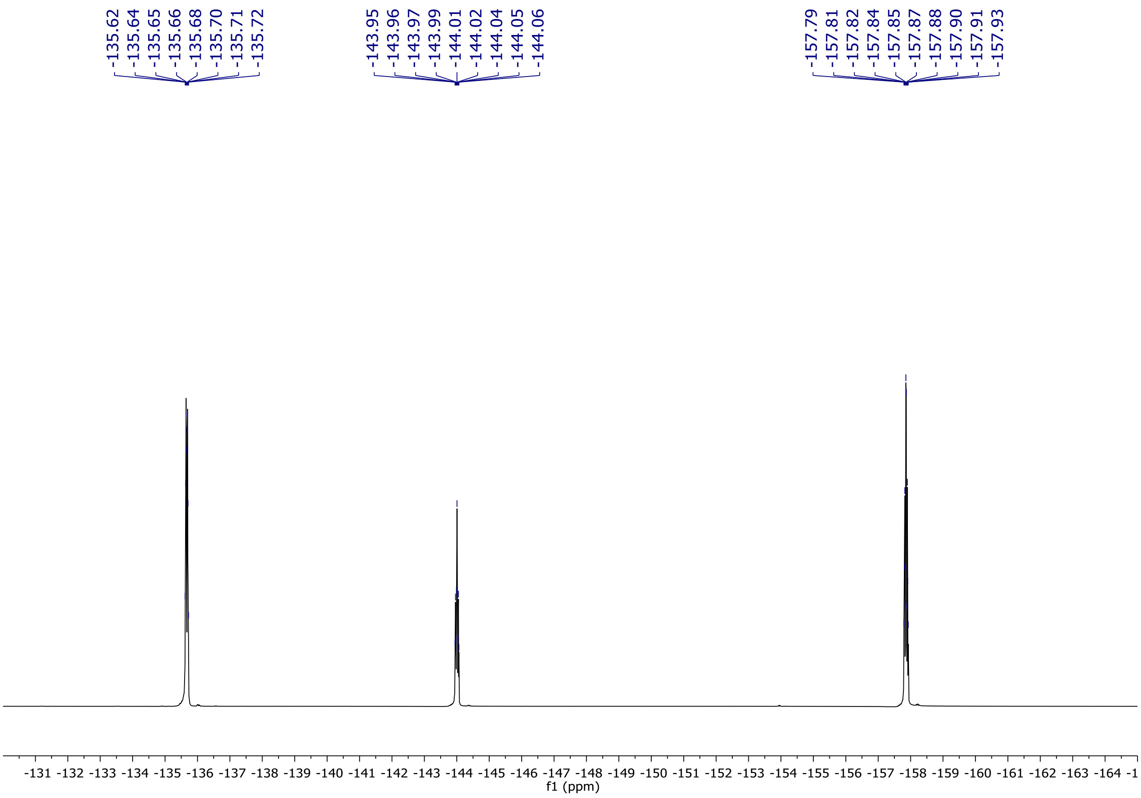

DEPT-135

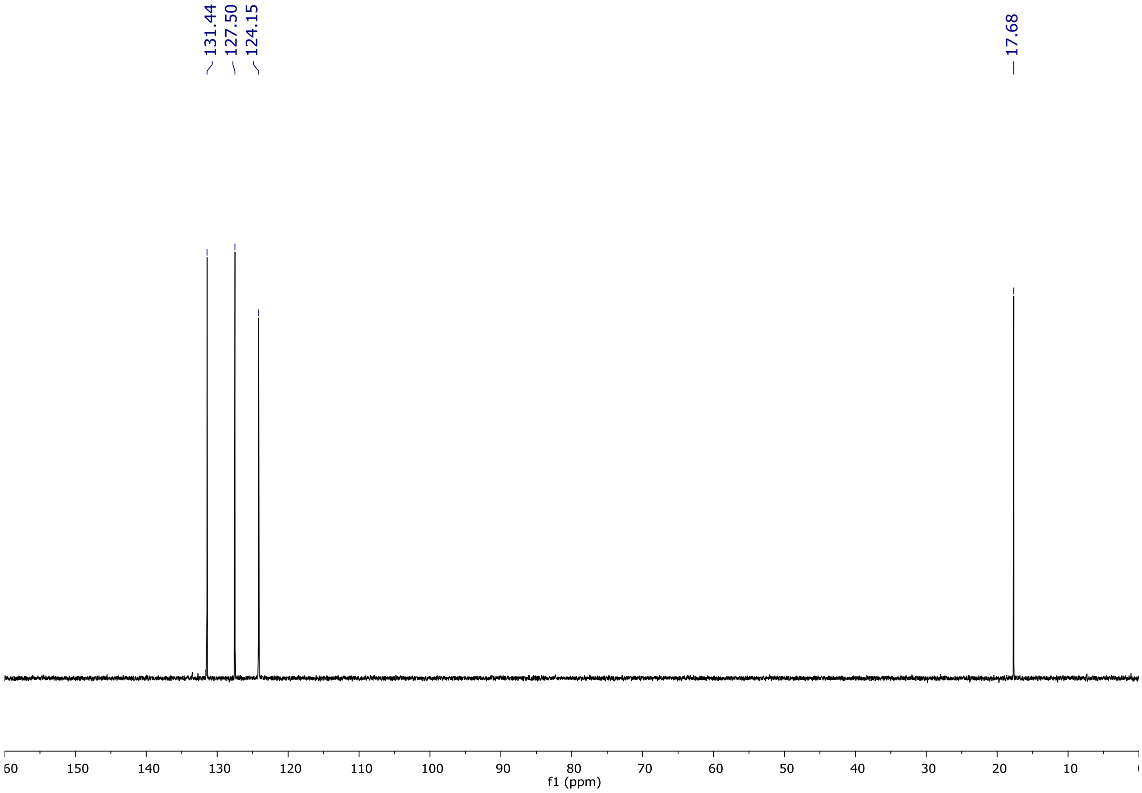

^{13}C NMR (126 MHz, $CDCl_3$)

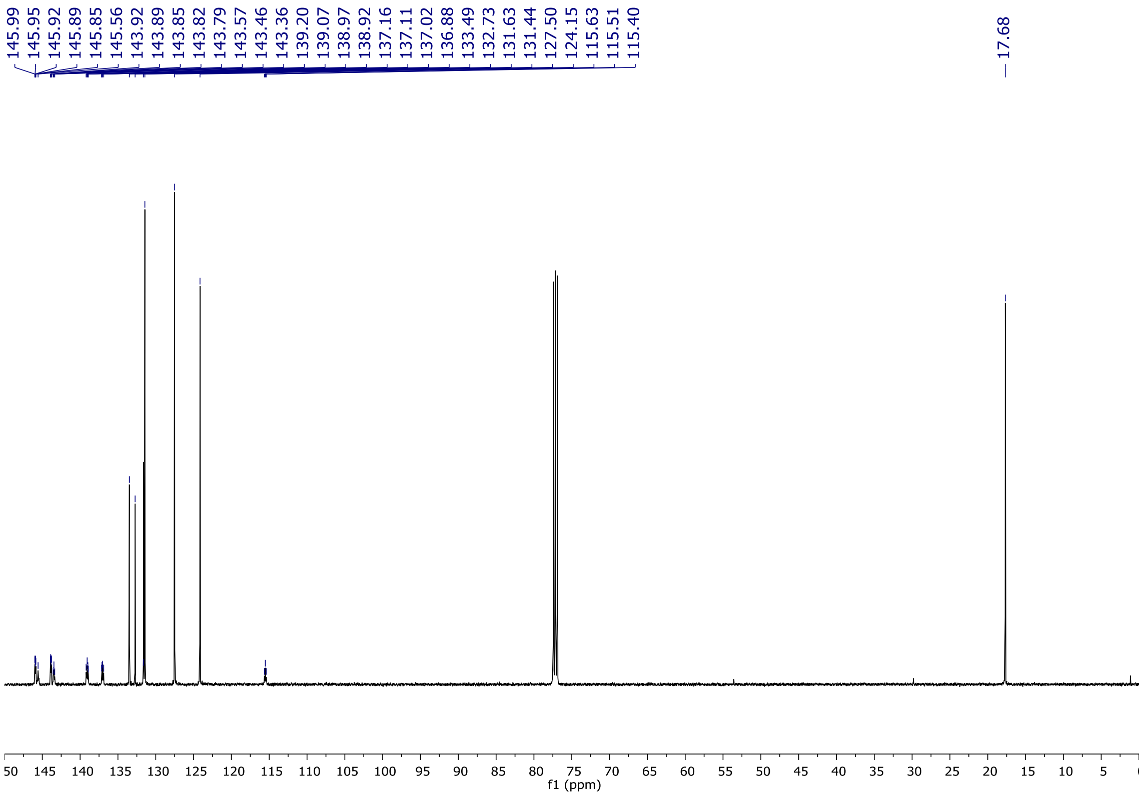

^{1}H NMR (500 MHz, $CDCl_3$)

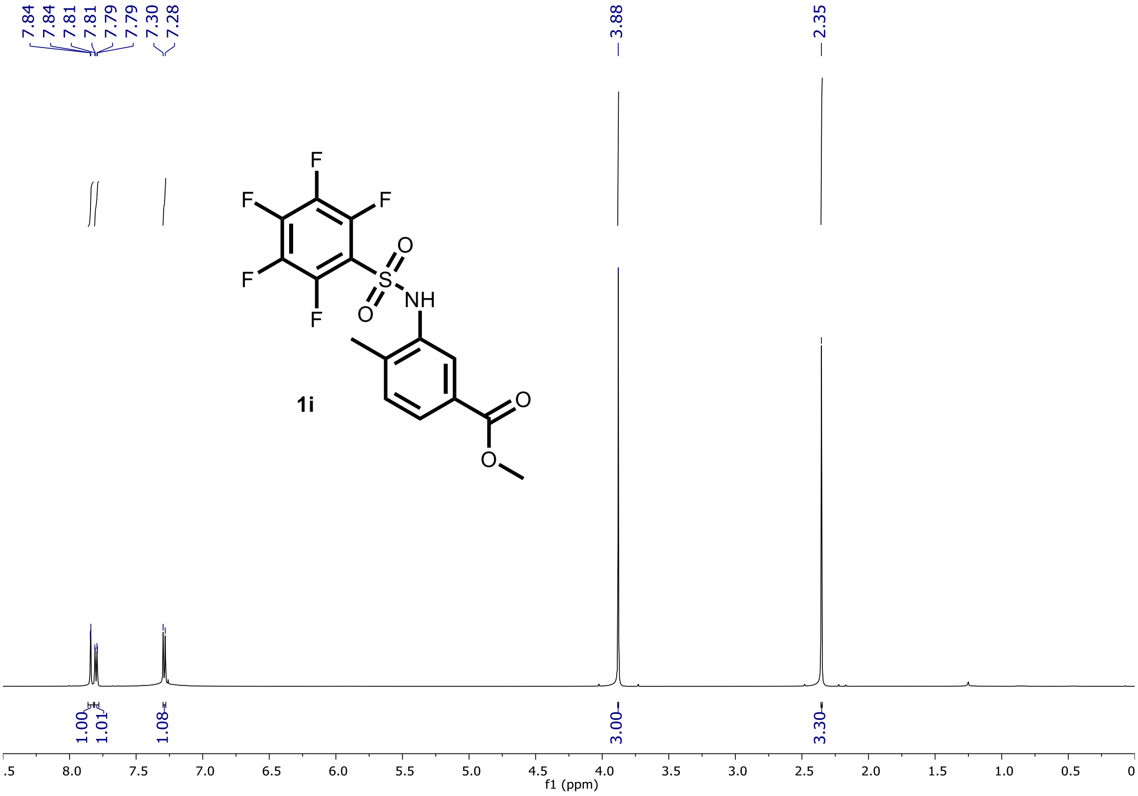

^{19}F NMR (471 MHz, $CDCl_3$)

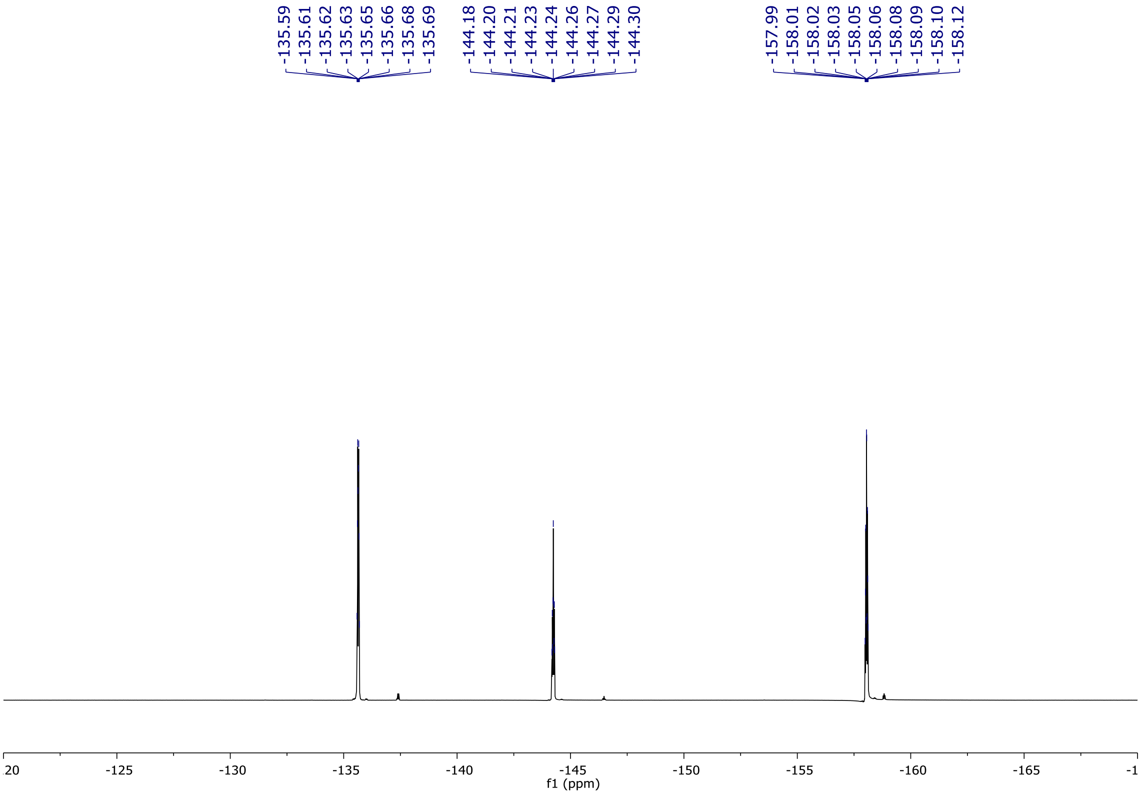

DEPT-135

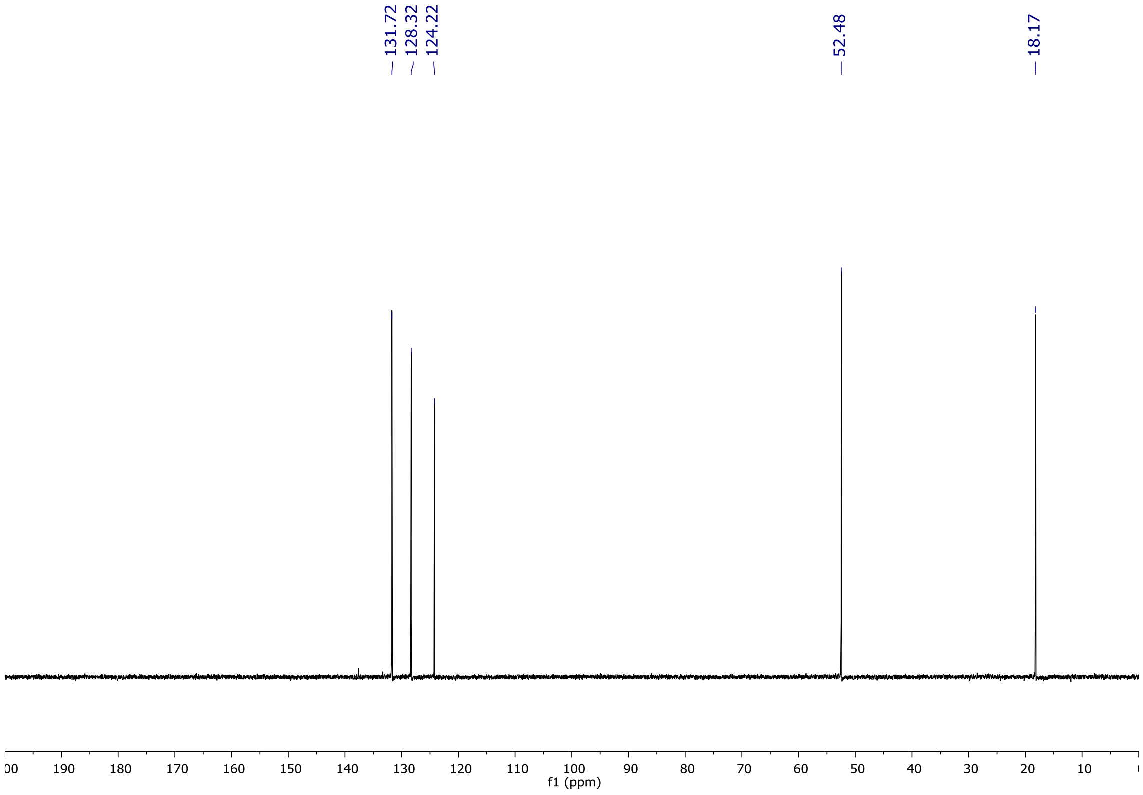

^{13}C NMR (126 MHz, $CDCl_3$)

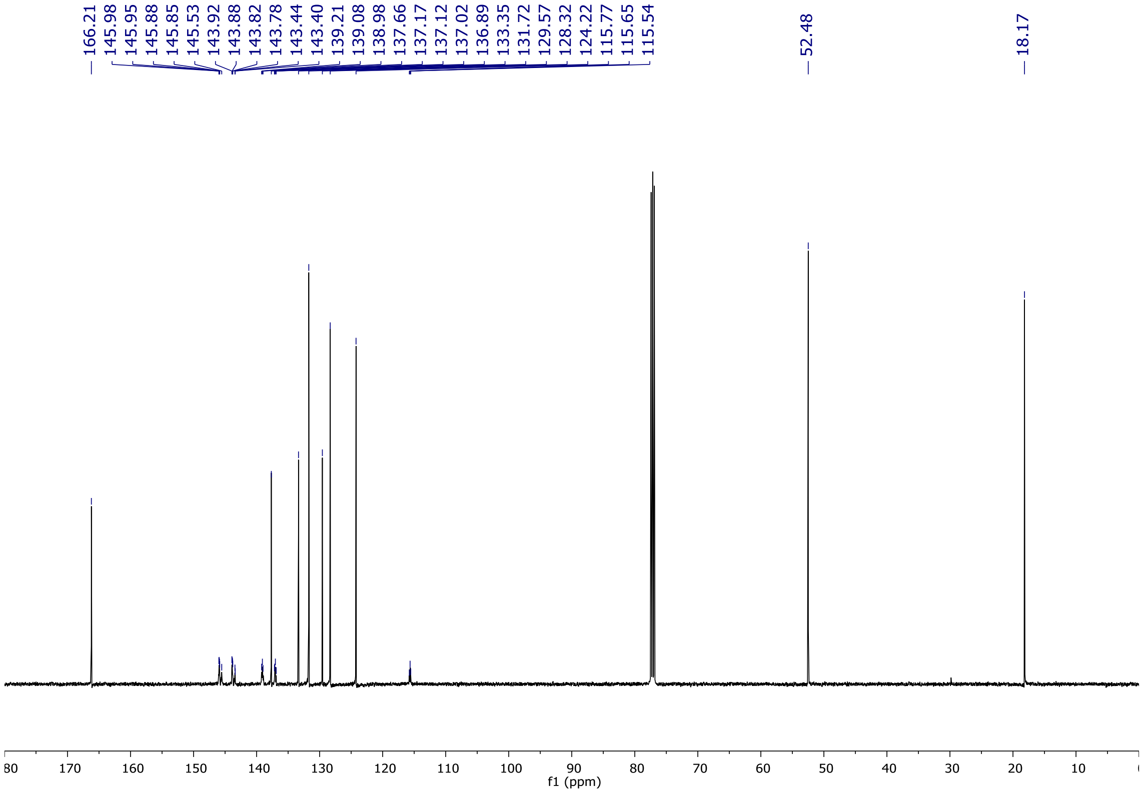

^{1}H NMR (500 MHz, $CDCl_3$)

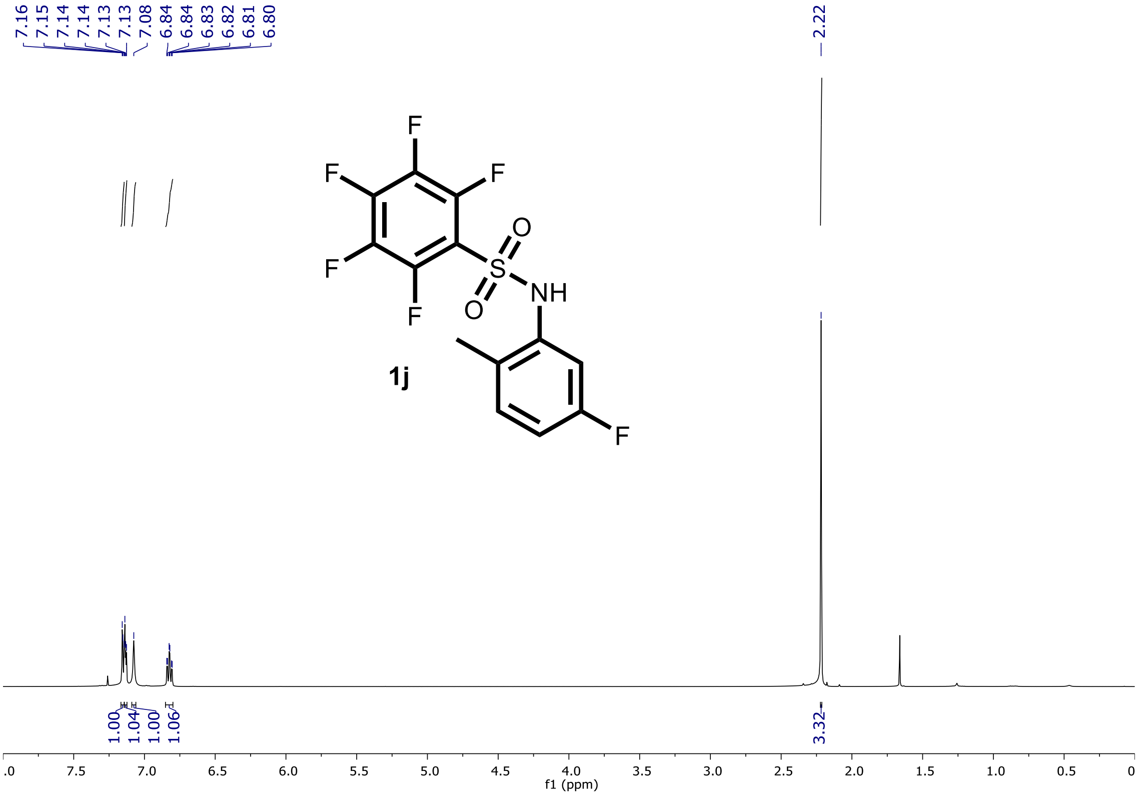

^{19}F NMR (471 MHz, $CDCl_3$)

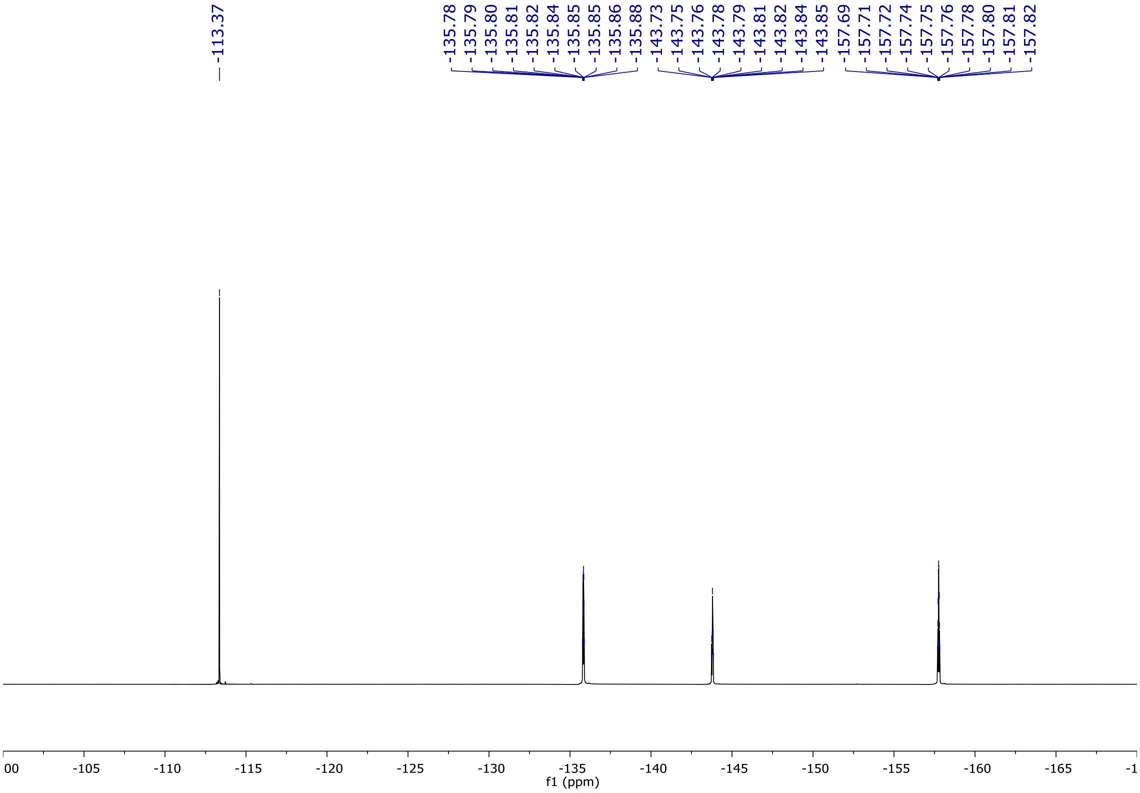

DEPT-135

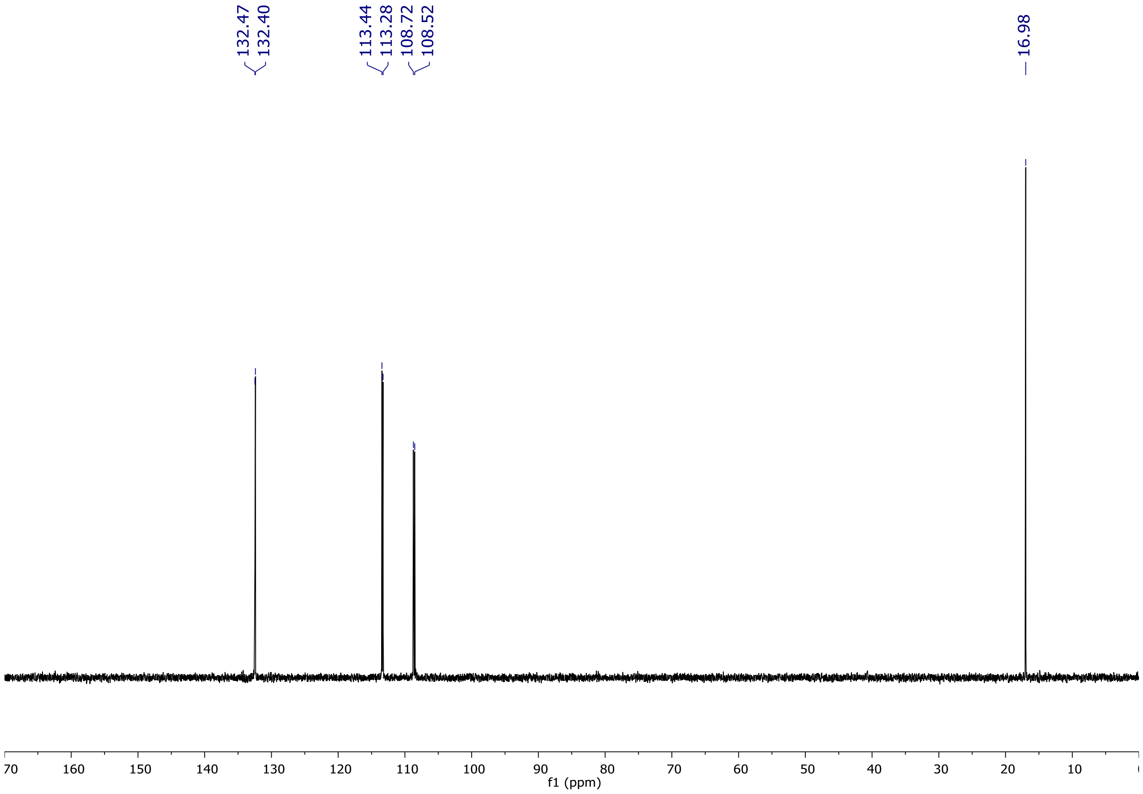

^{13}C NMR (126 MHz, $CDCl_3$)

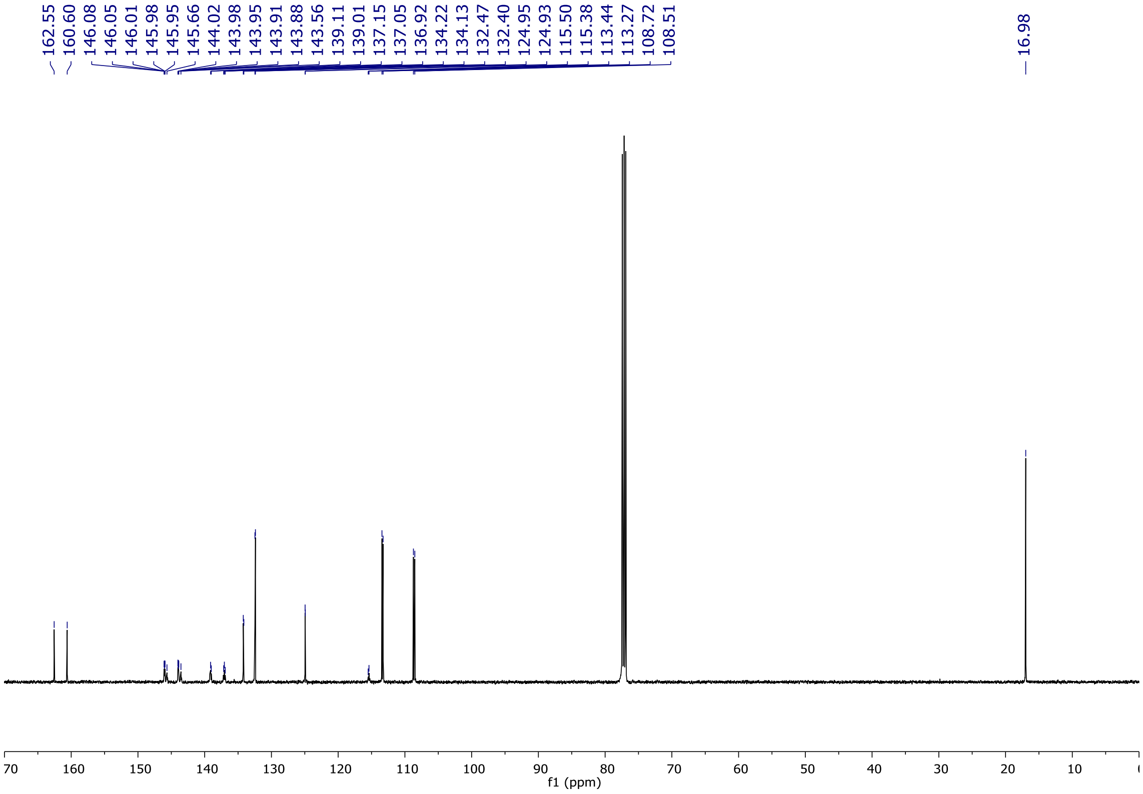

^{1}H NMR (500 MHz, $CDCl_3$)

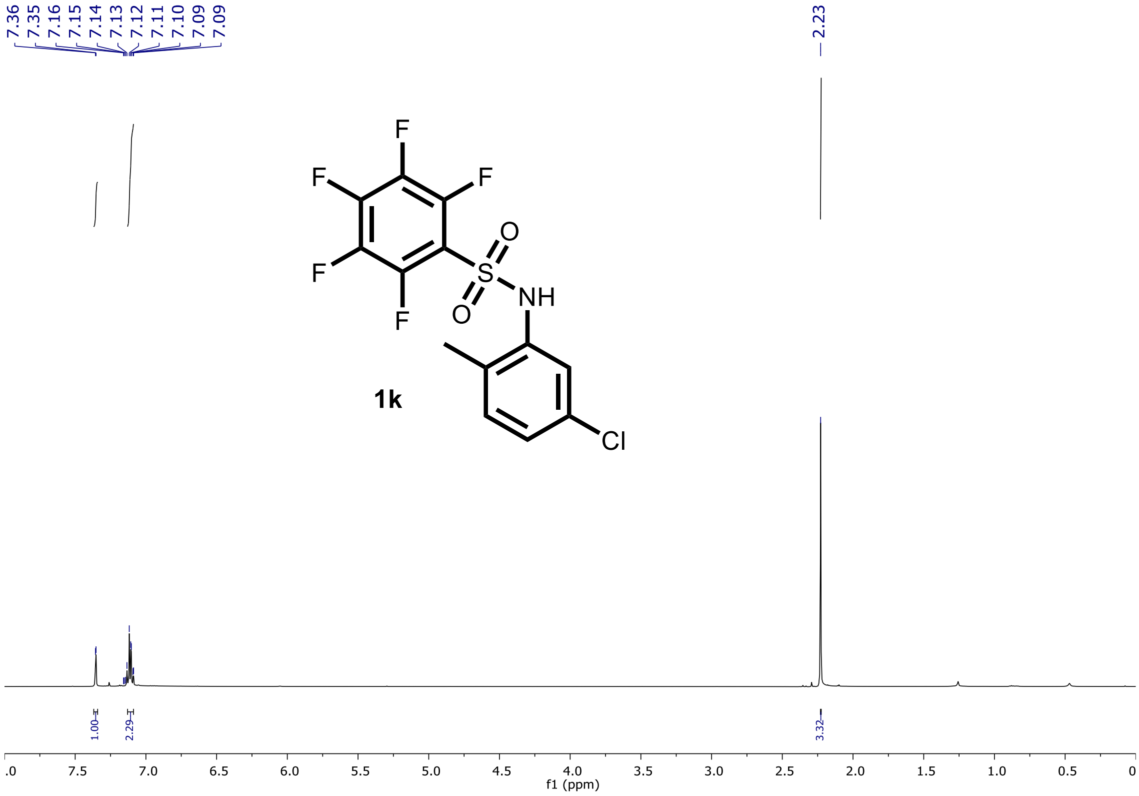

^{19}F NMR (471 MHz, $CDCl_3$)

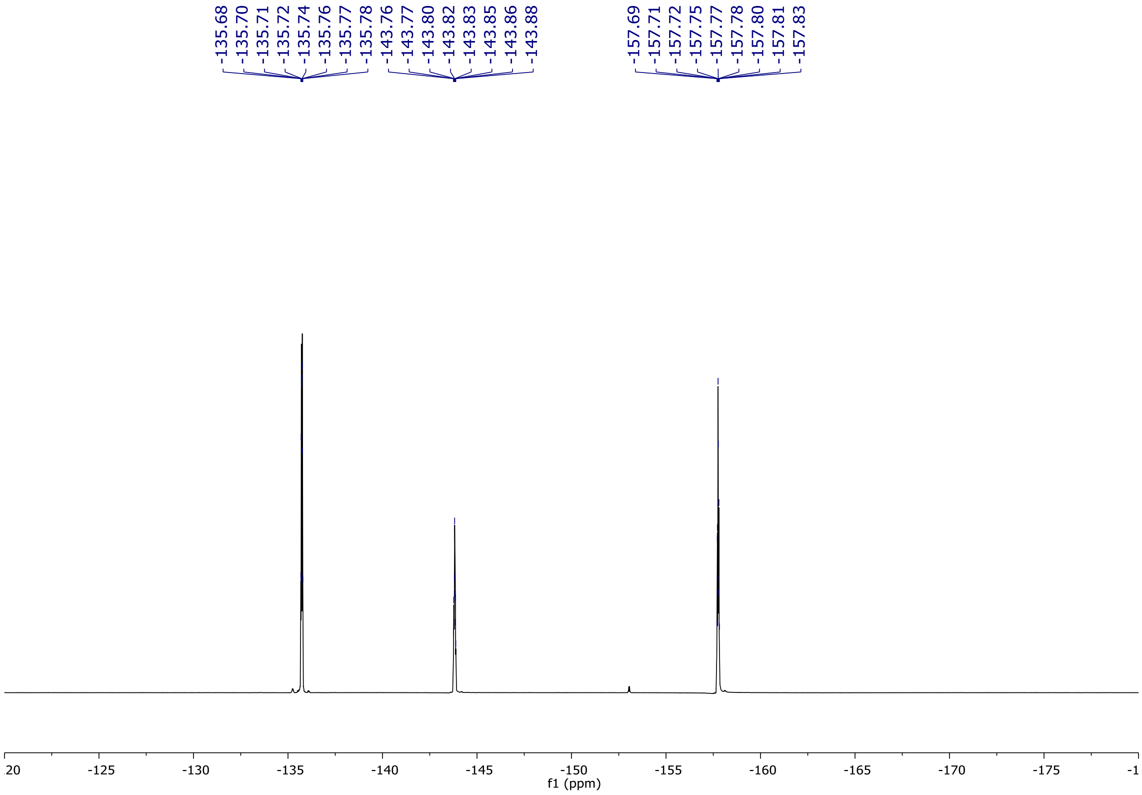

DEPT-135

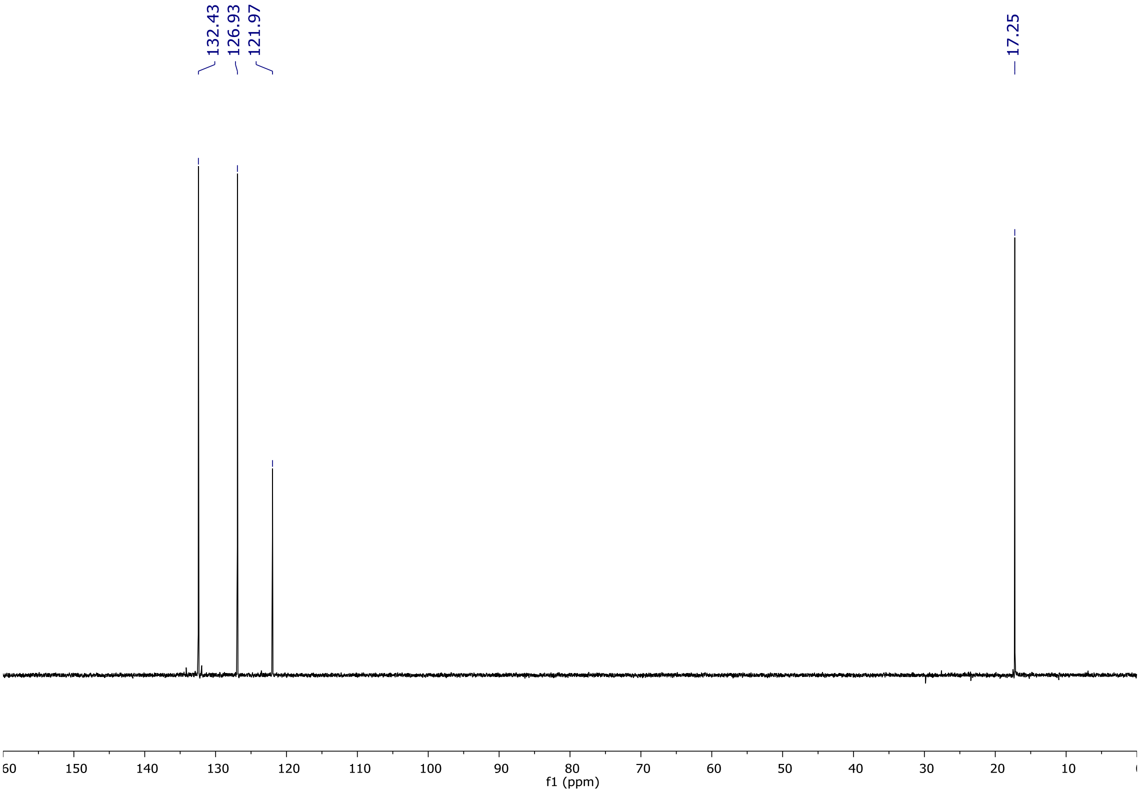

^{13}C NMR (126 MHz, $CDCl_3$)

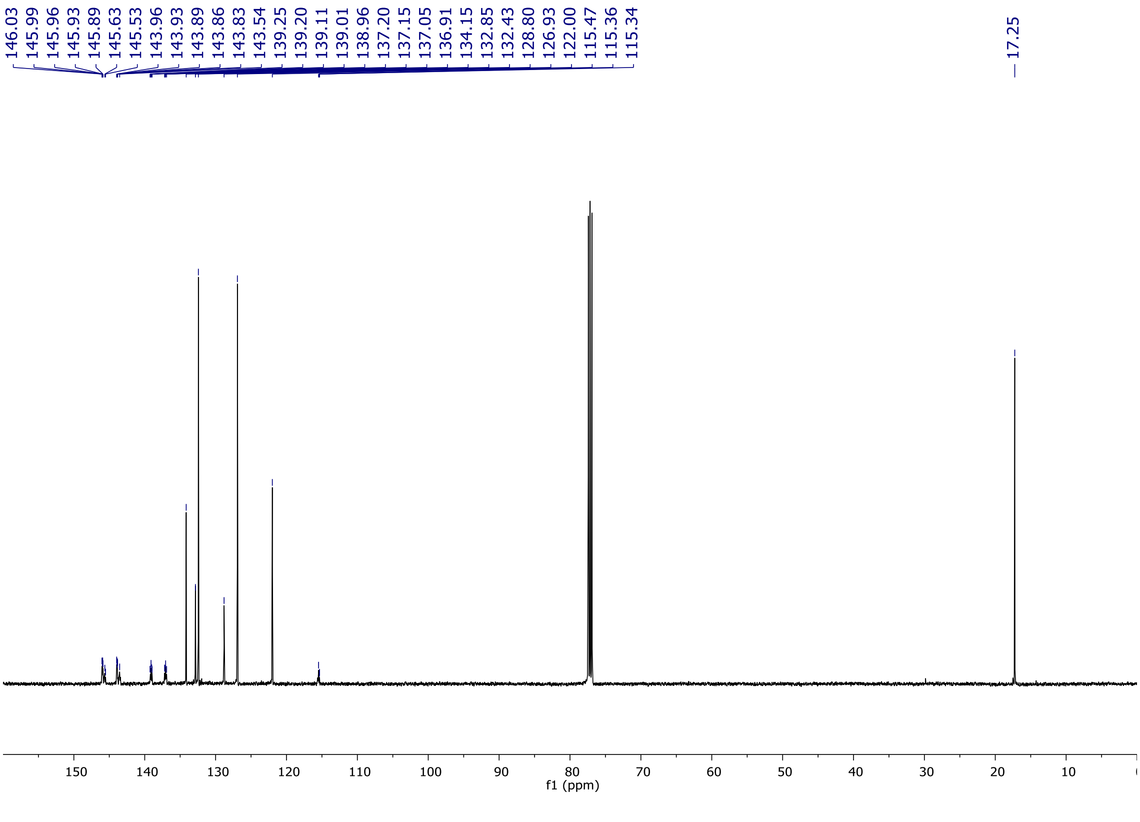

^{1}H NMR (500 MHz, CDCl$_3$)

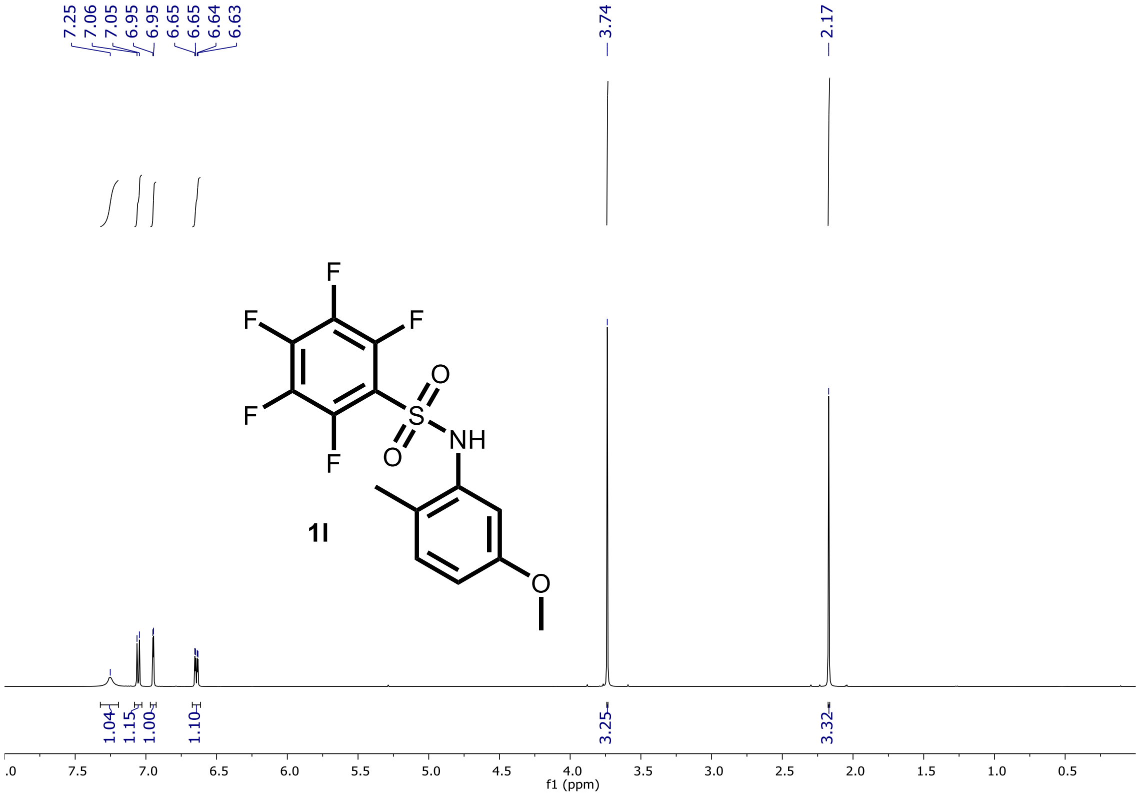

^{19}F NMR (471 MHz, CDCl$_3$)

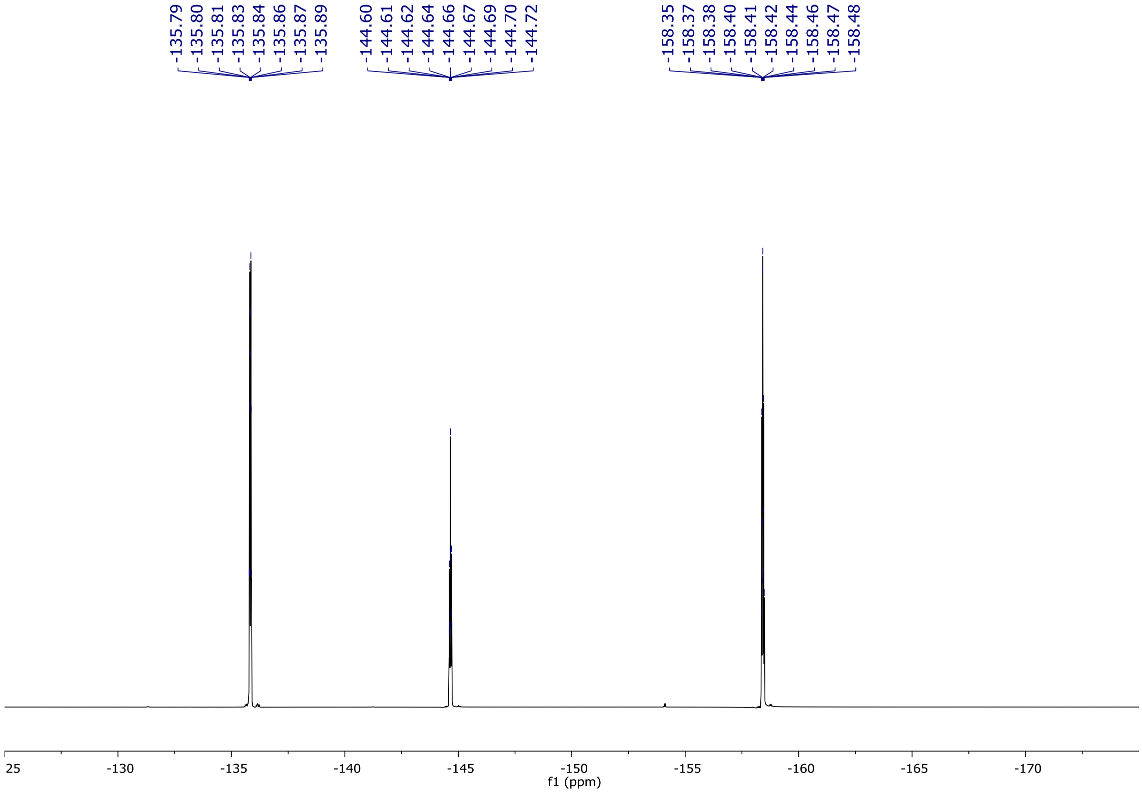

DEPT-135

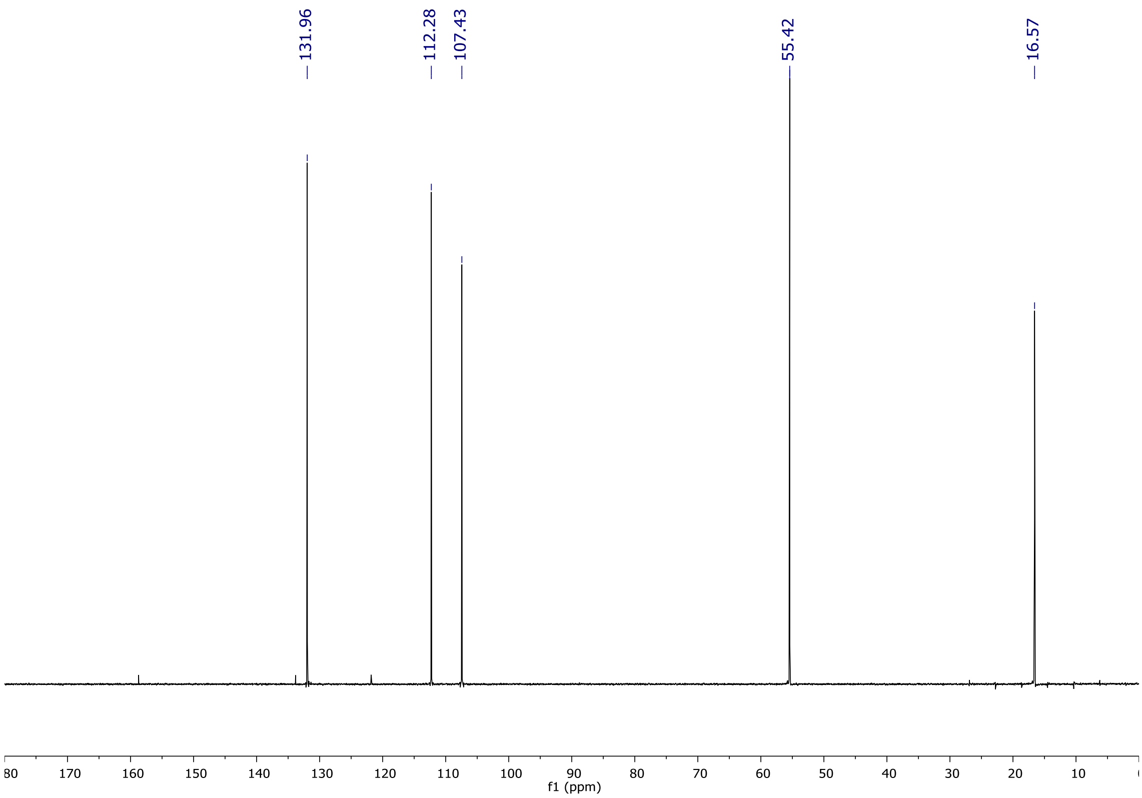

^{13}C NMR (126 MHz, $CDCl_3$)

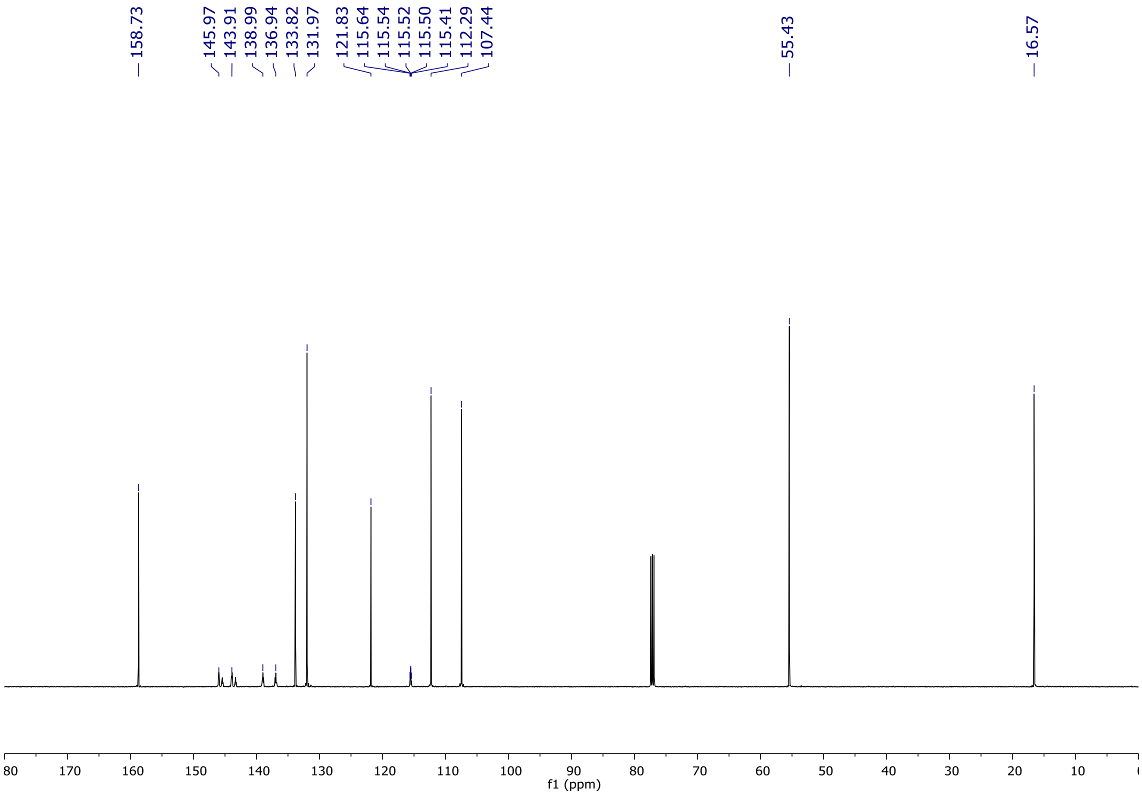

¹H NMR (500 MHz, CDCl₃)

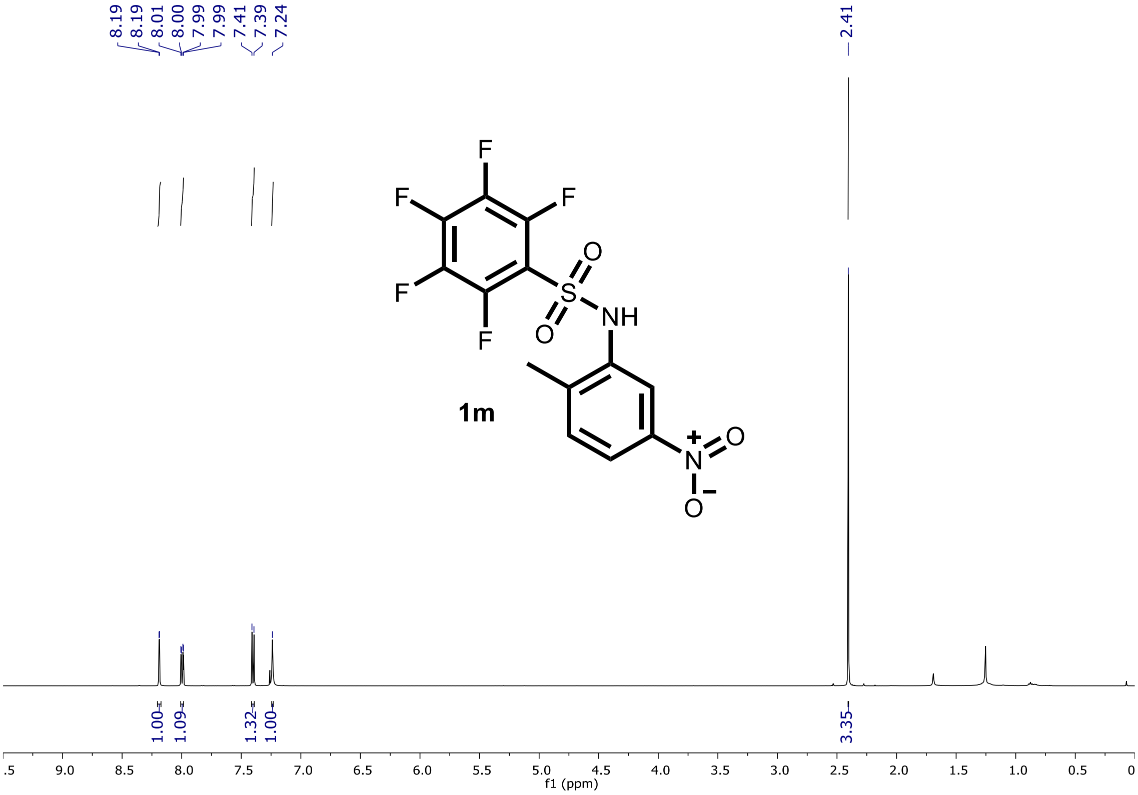

¹⁹F NMR (471 MHz, CDCl₃)

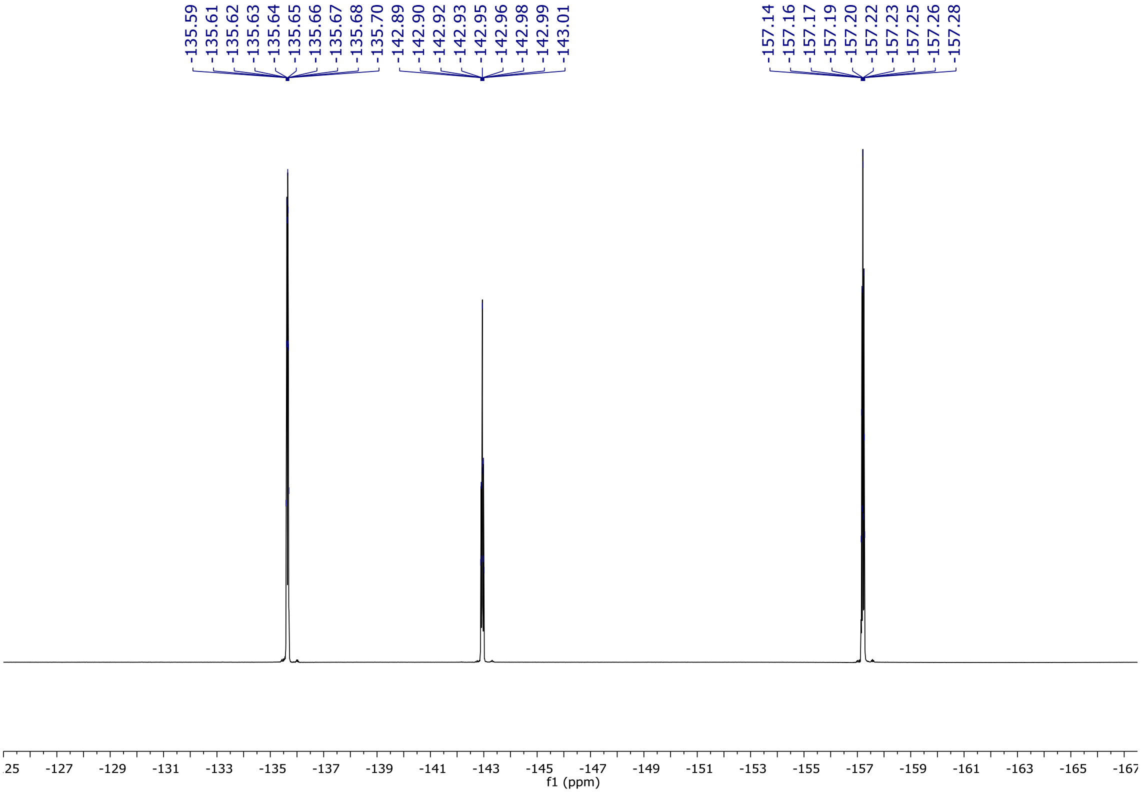

DEPT-135

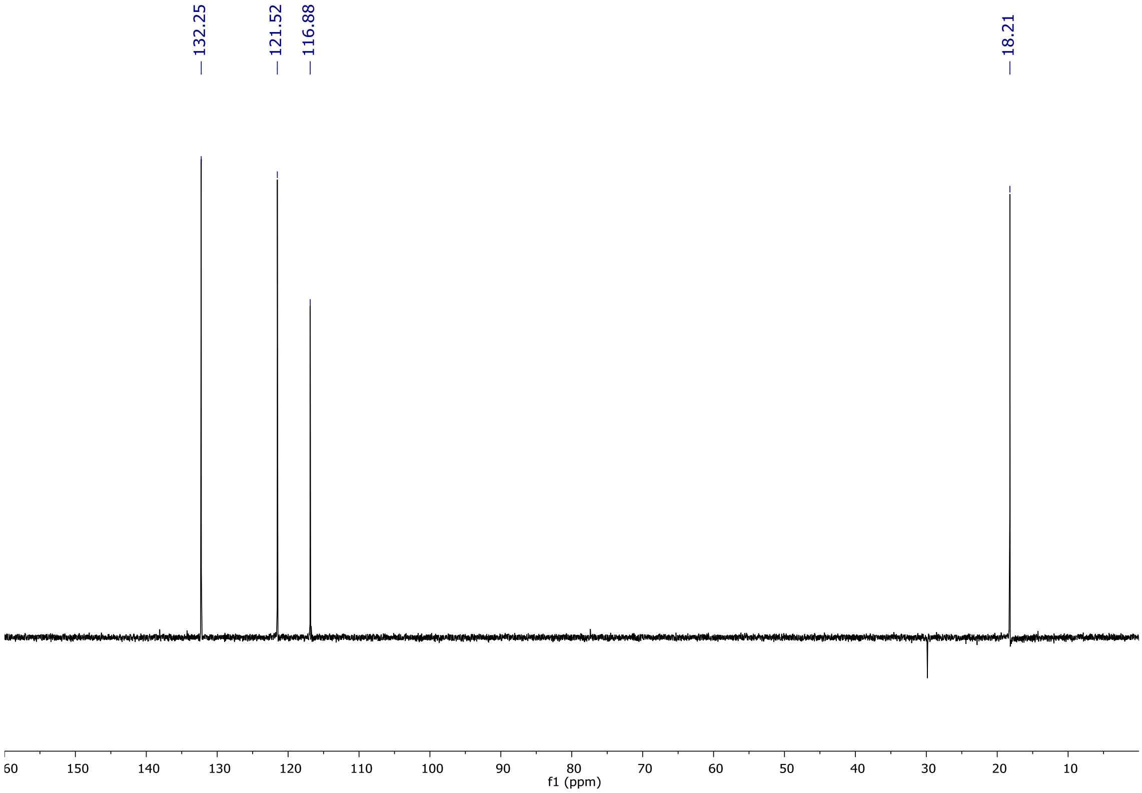

^{13}C NMR (126 MHz, $CDCl_3$)

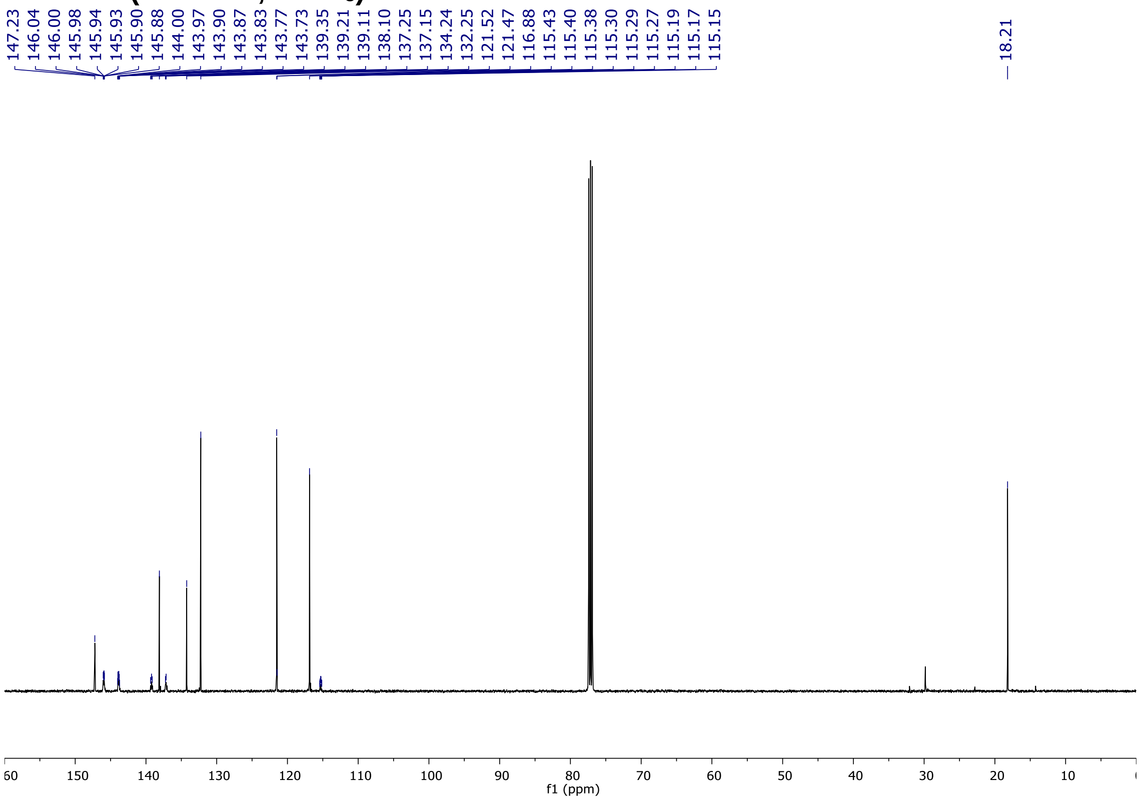

^{1}H NMR (500 MHz, $CDCl_3$)

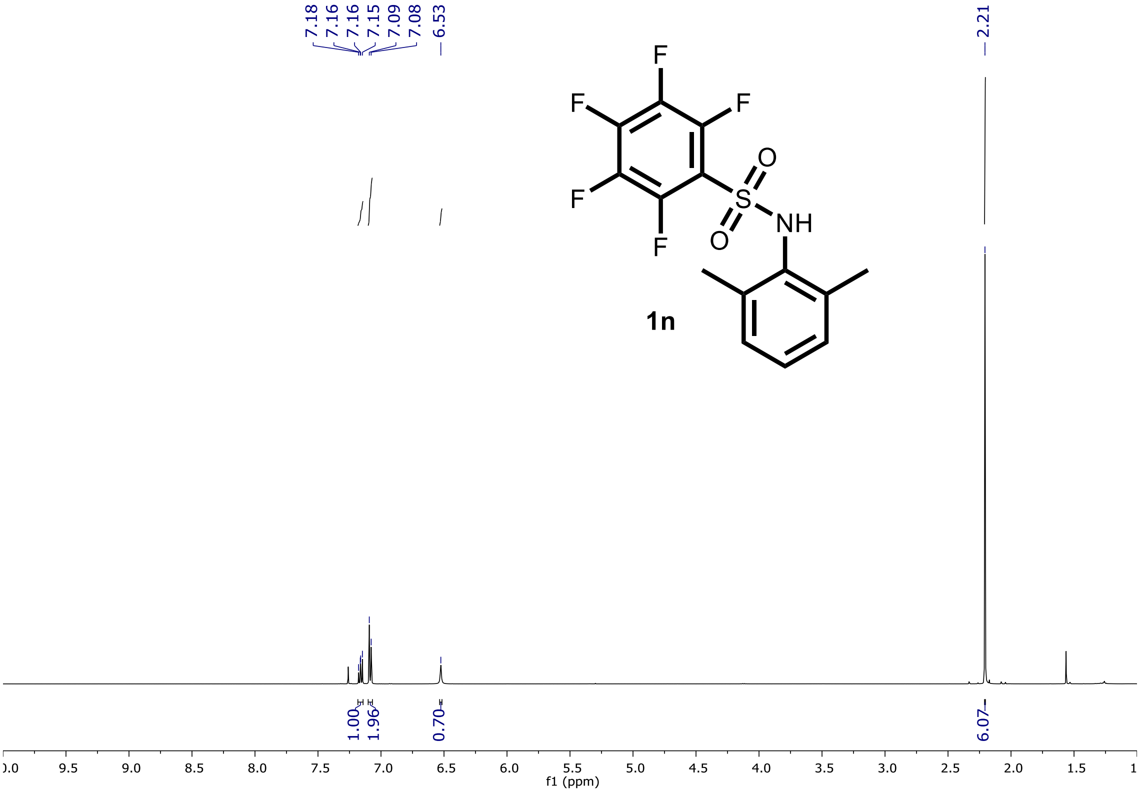

^{19}F NMR (471 MHz, $CDCl_3$)

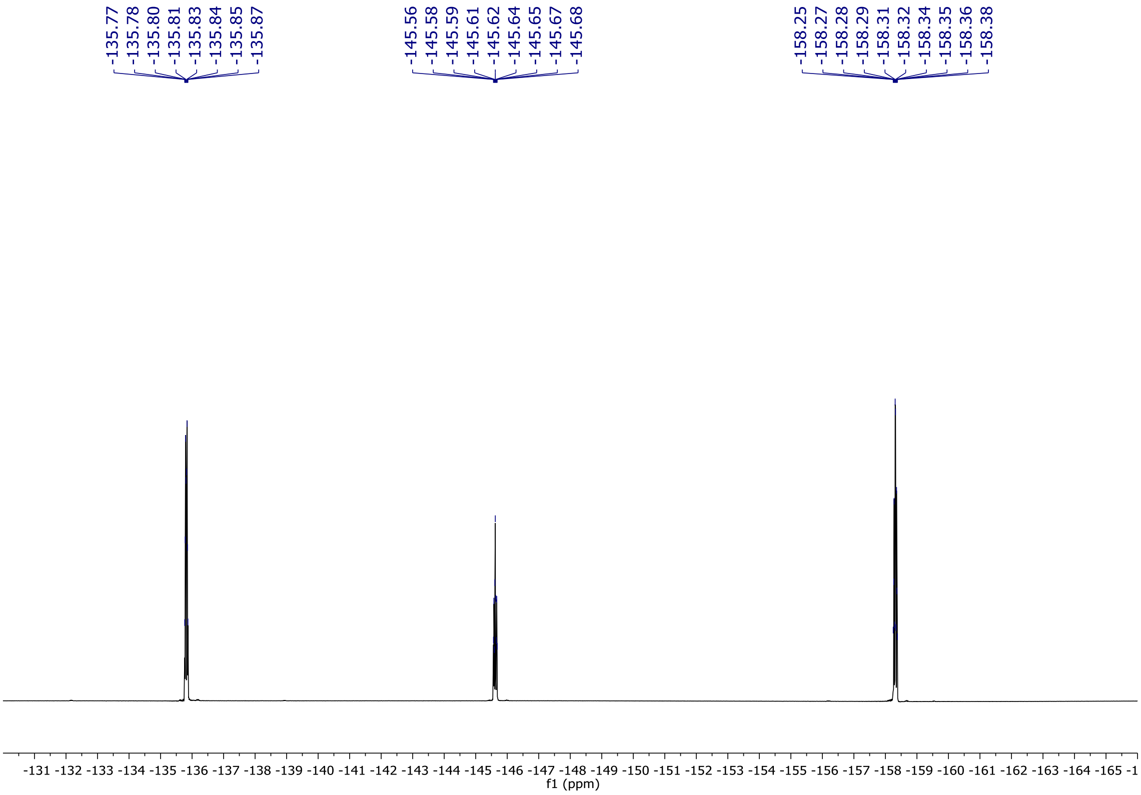

DEPT-135

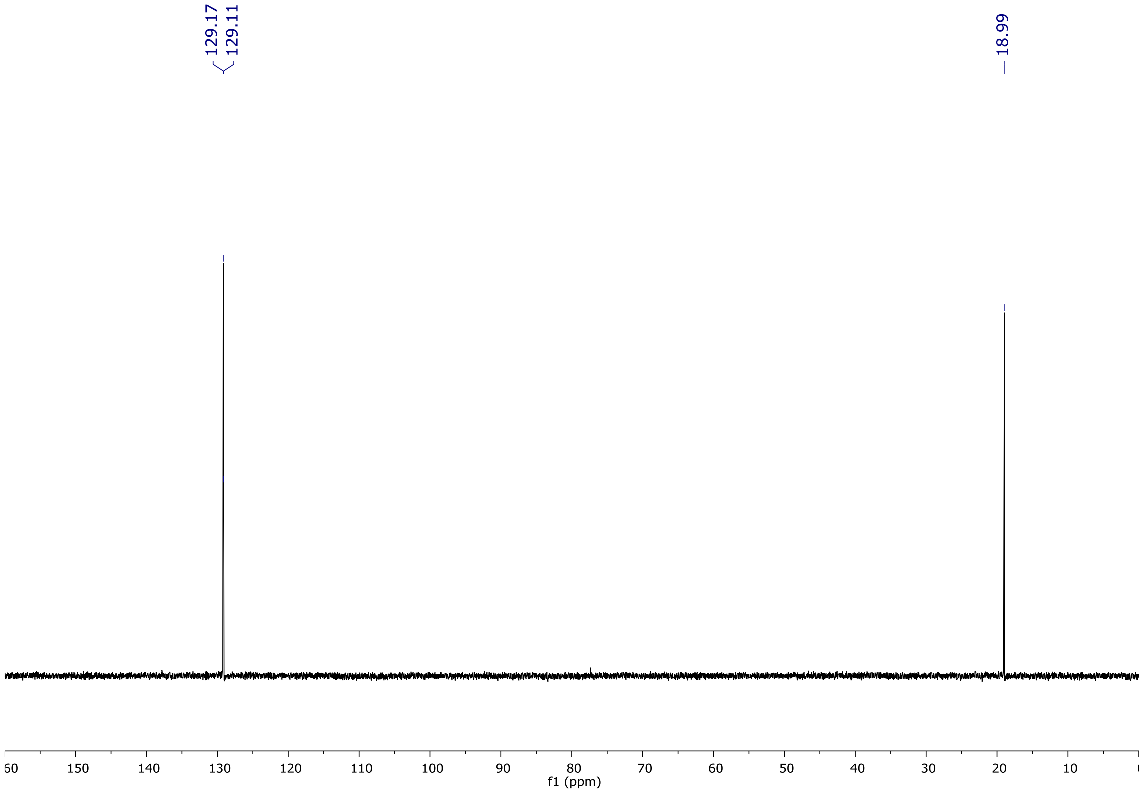

^{13}C NMR (126 MHz, $CDCl_3$)

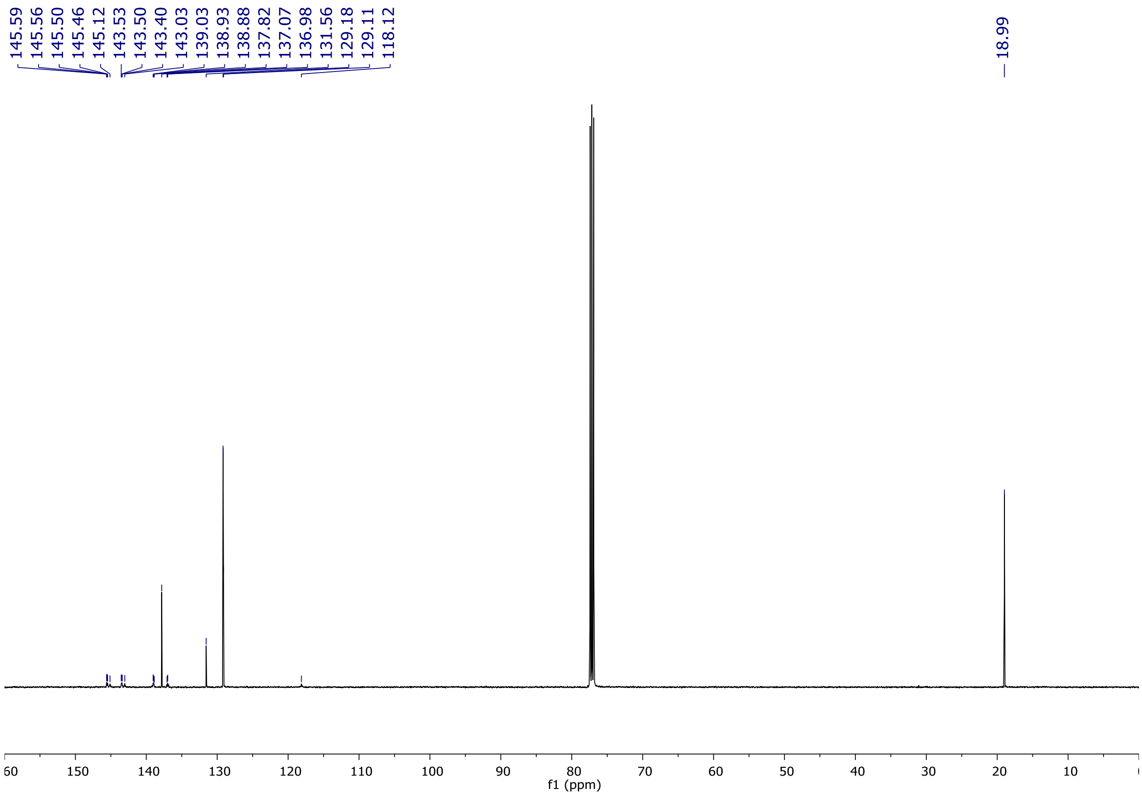

^{1}H NMR (500 MHz, $CDCl_3$)

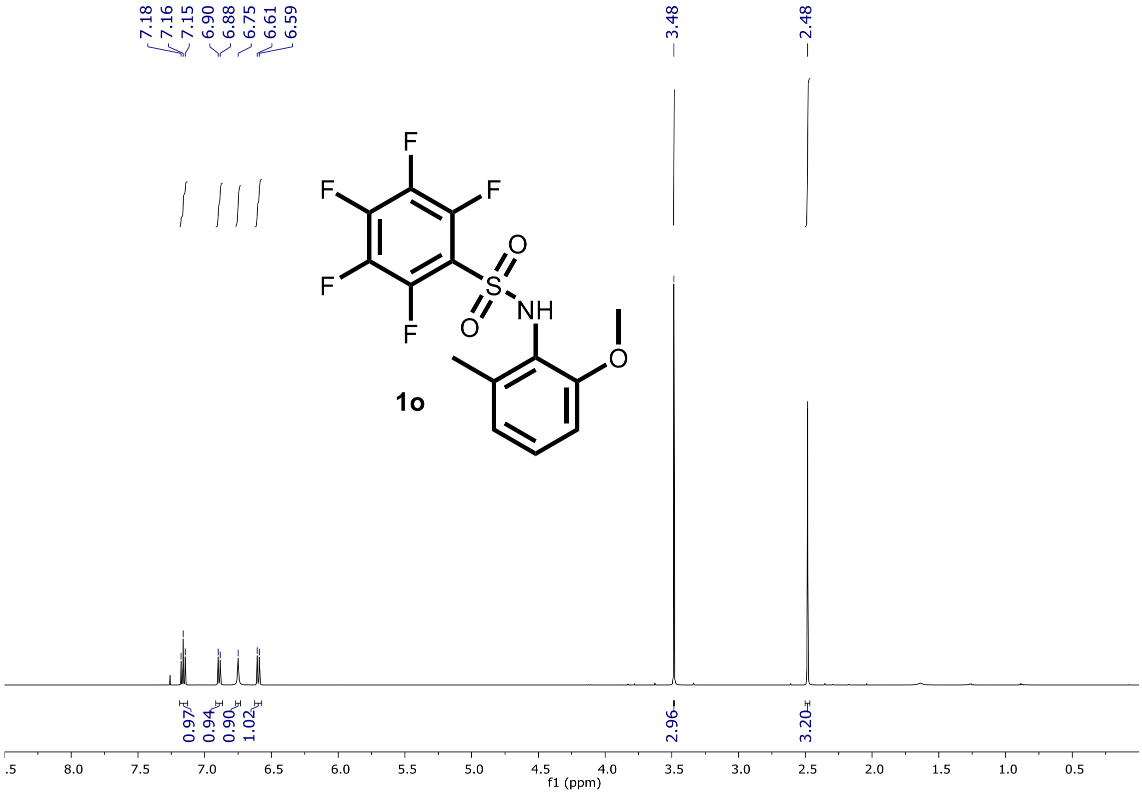

^{19}F NMR (471 MHz, $CDCl_3$)

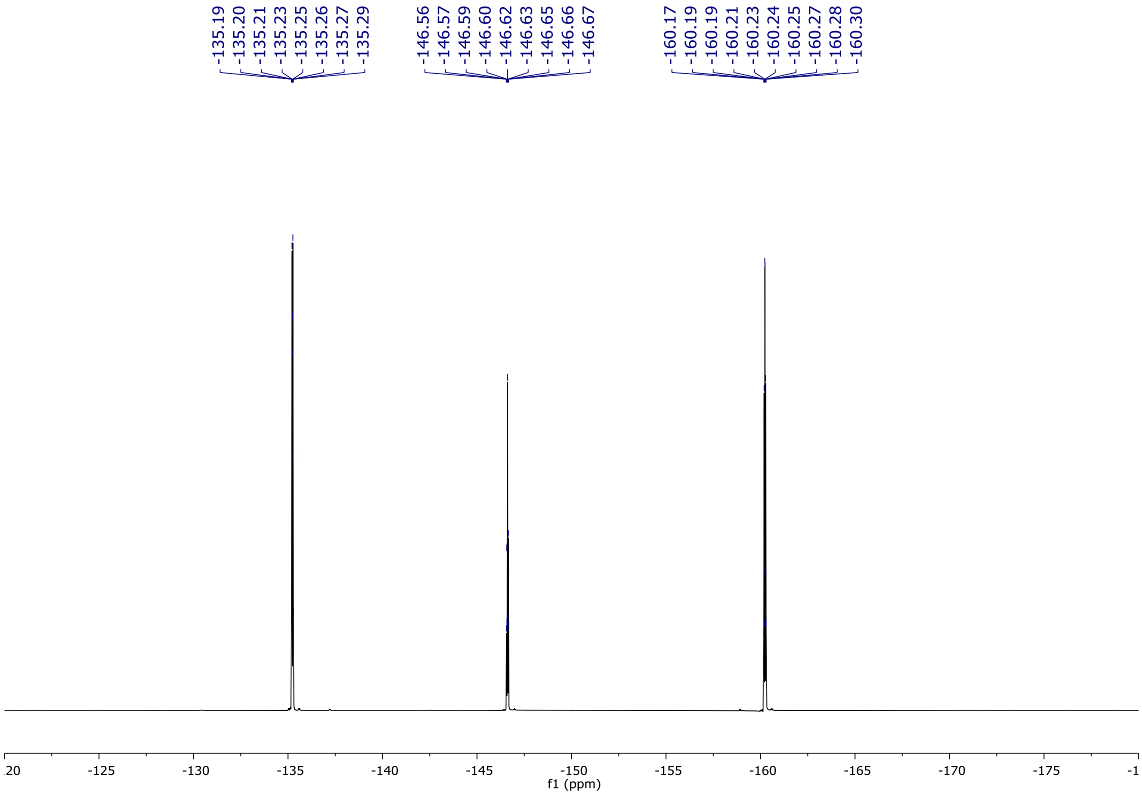

DEPT-135

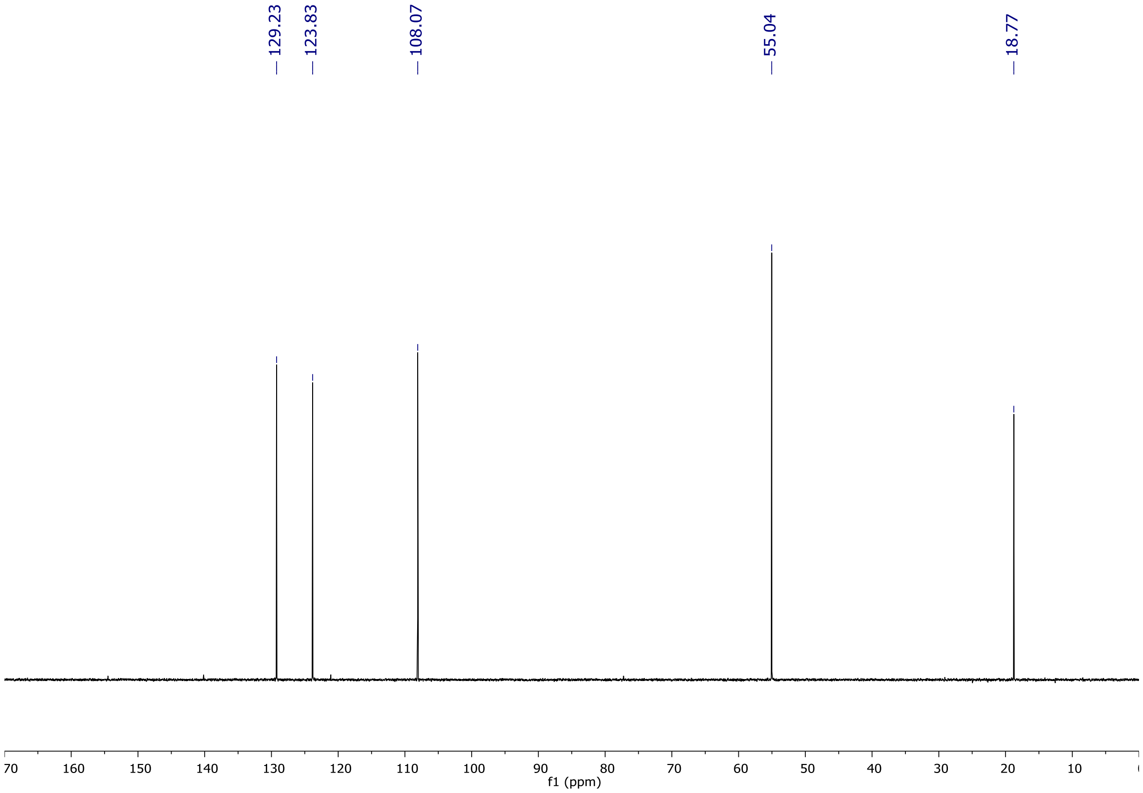

^{13}C NMR (126 MHz, $CDCl_3$)

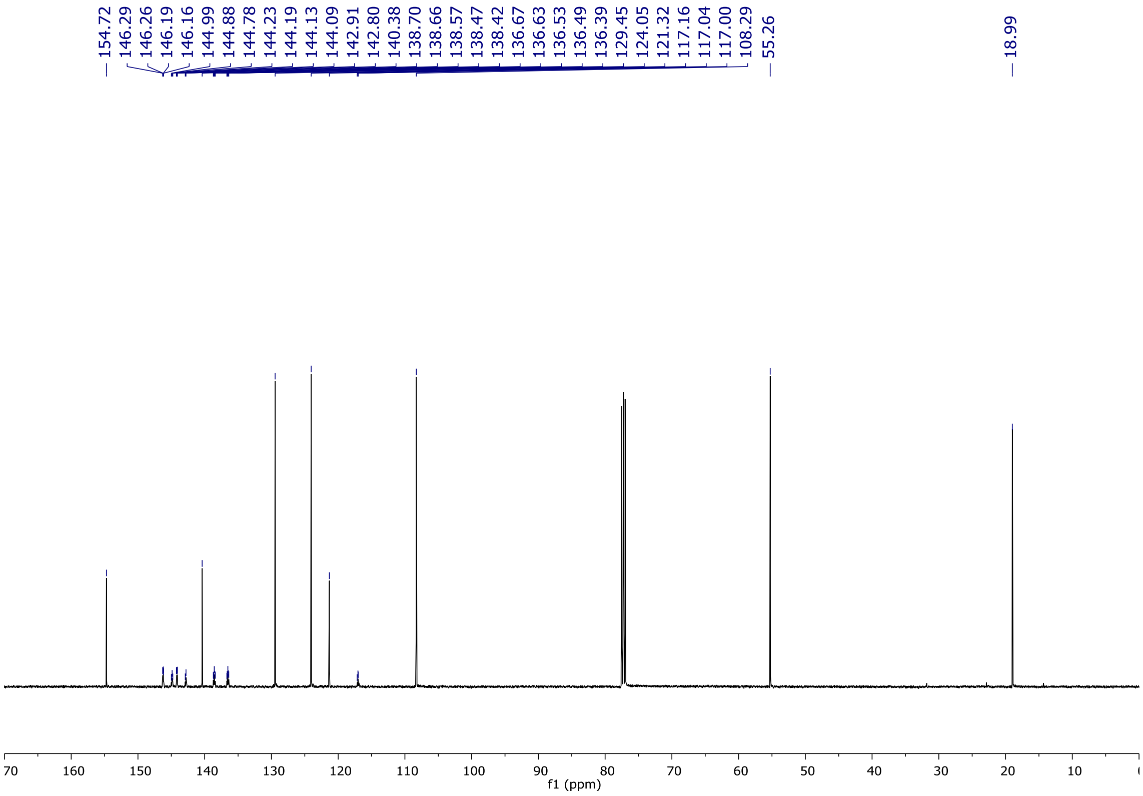

^{1}H NMR (500 MHz, CDCl$_3$)

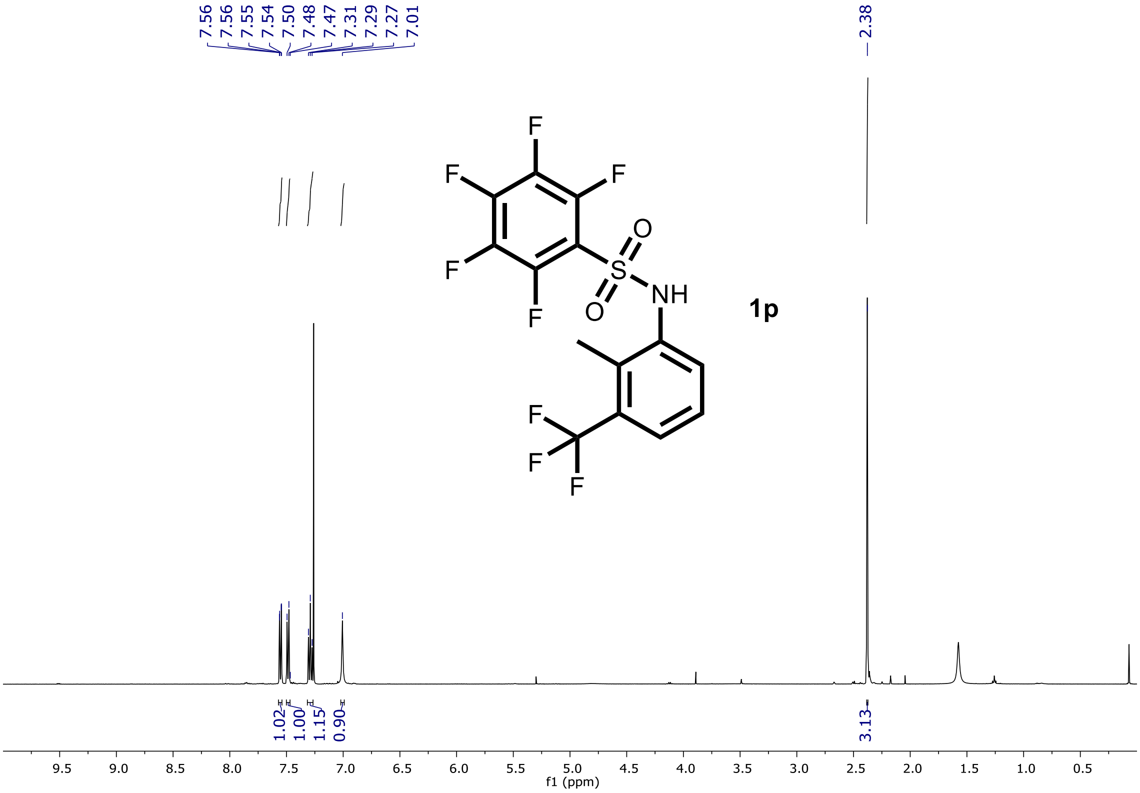

^{19}F NMR (471 MHz, CDCl$_3$)

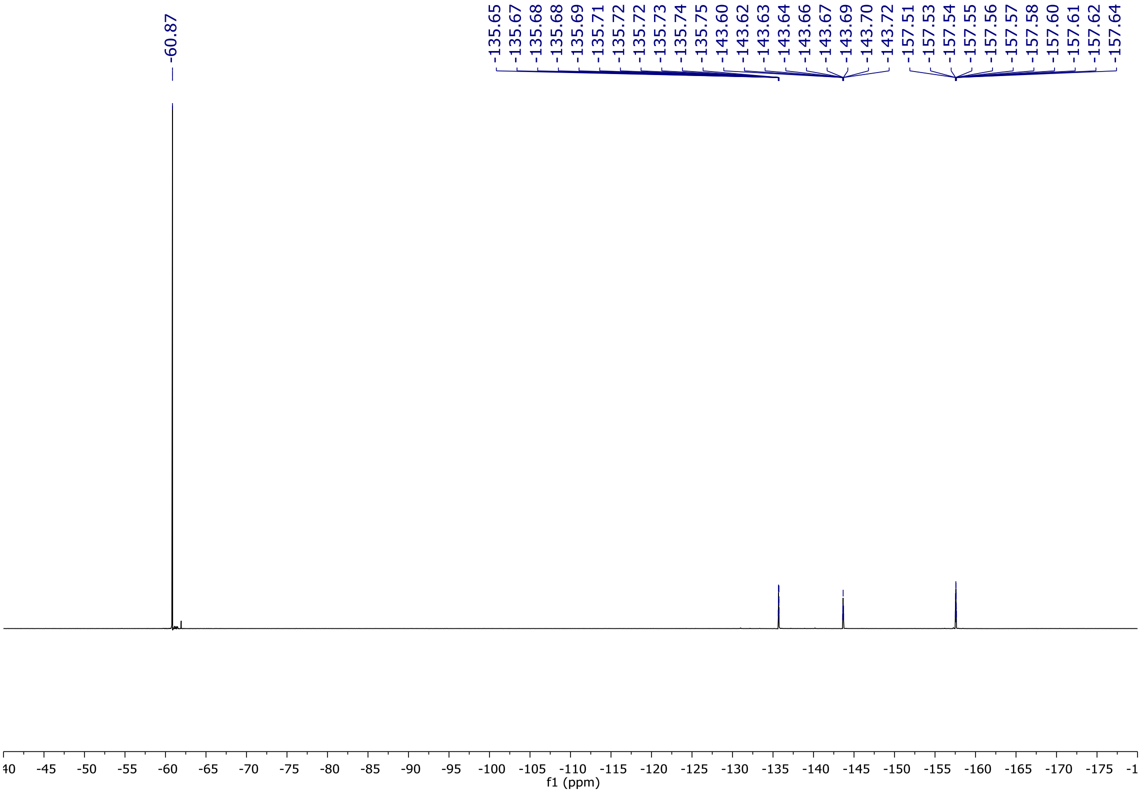

DEPT-135

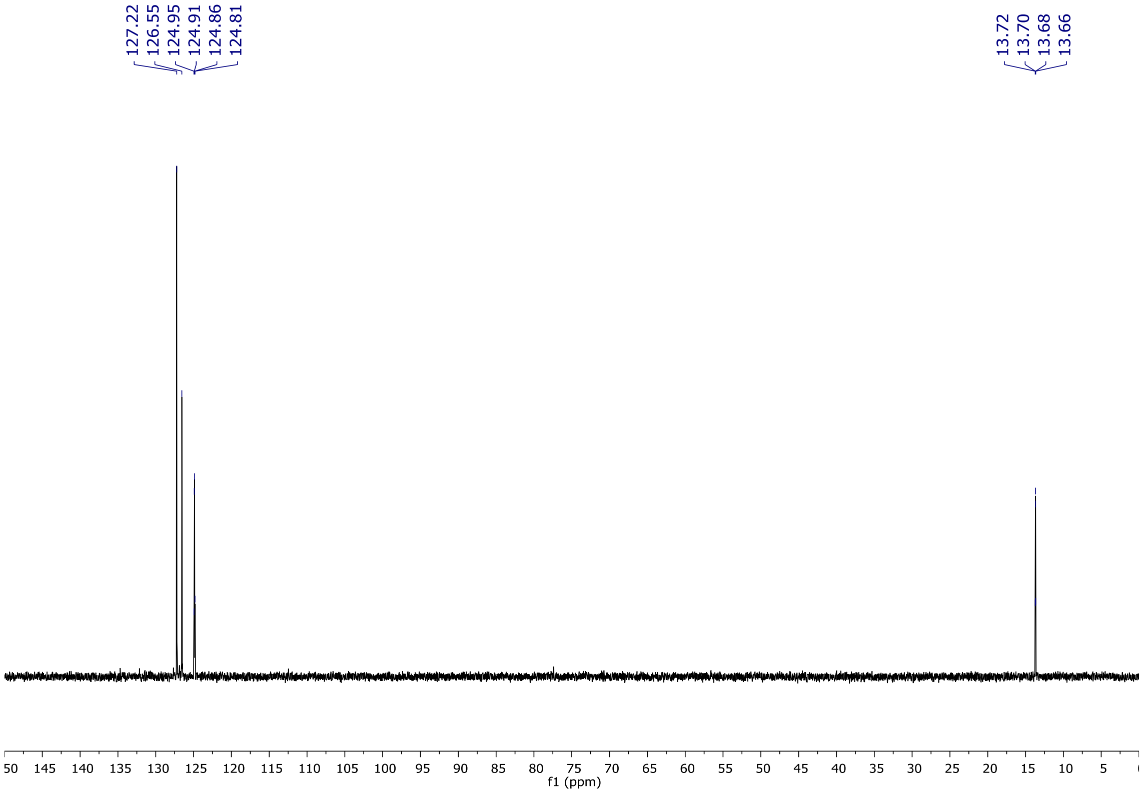

^{13}C NMR (126 MHz, $CDCl_3$)

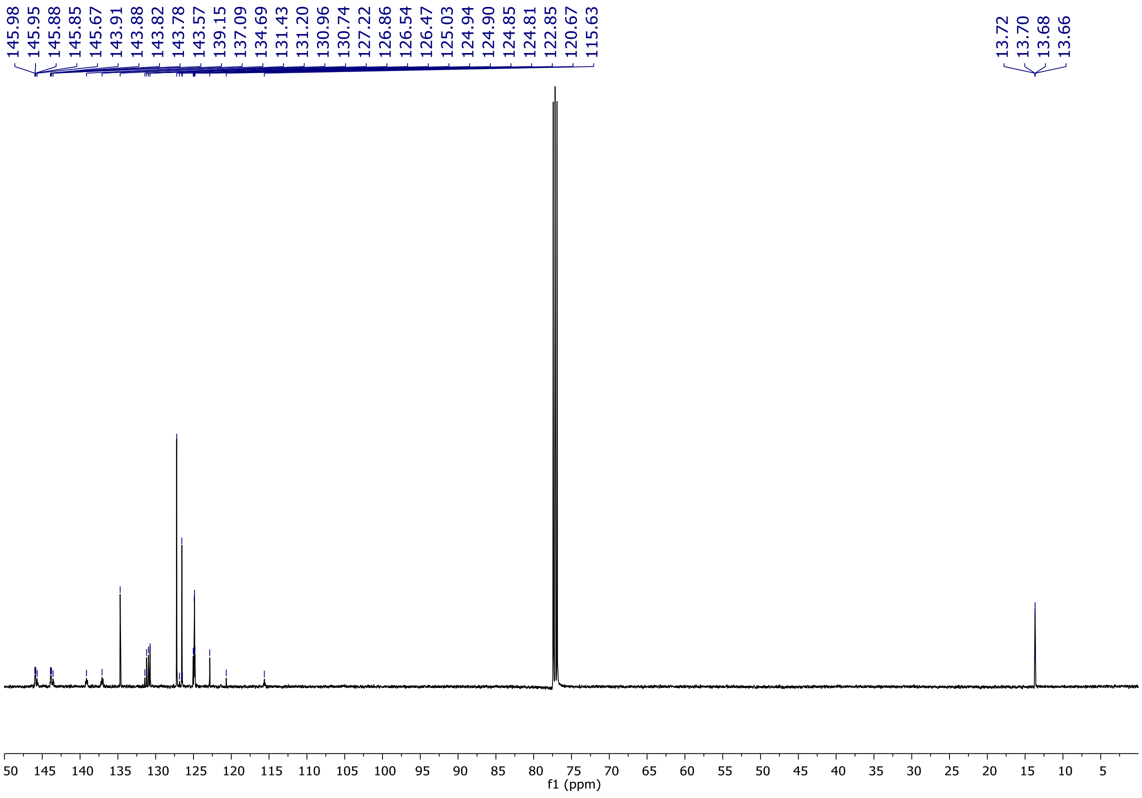

^{1}H NMR (500 MHz, $CDCl_3$)

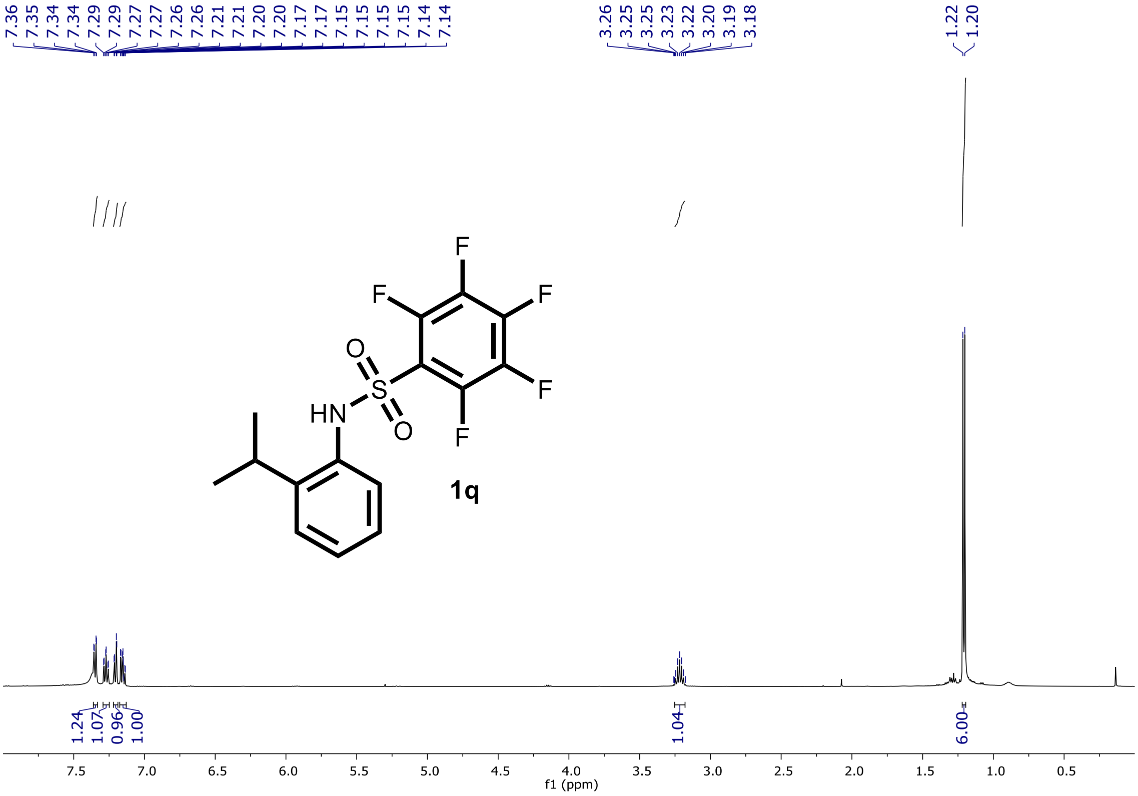

^{19}F NMR (471 MHz, $CDCl_3$)

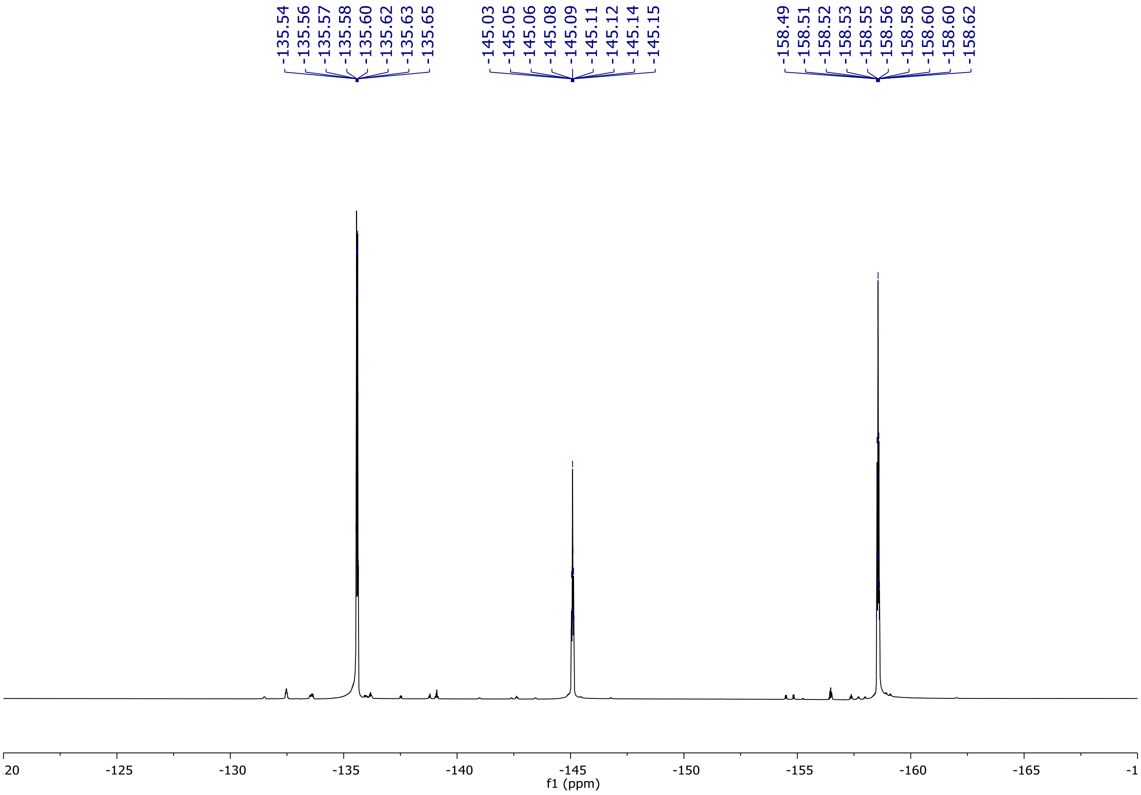

DEPT-135

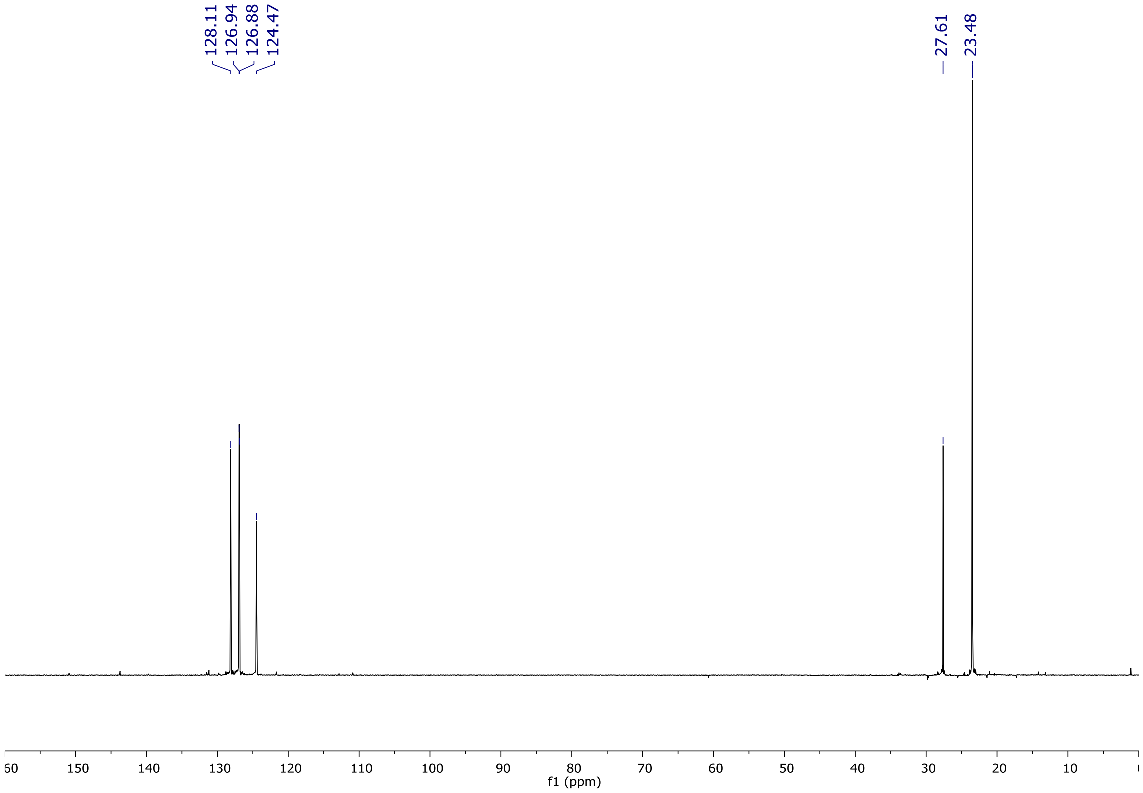

^{13}C NMR (126 MHz, $CDCl_3$)

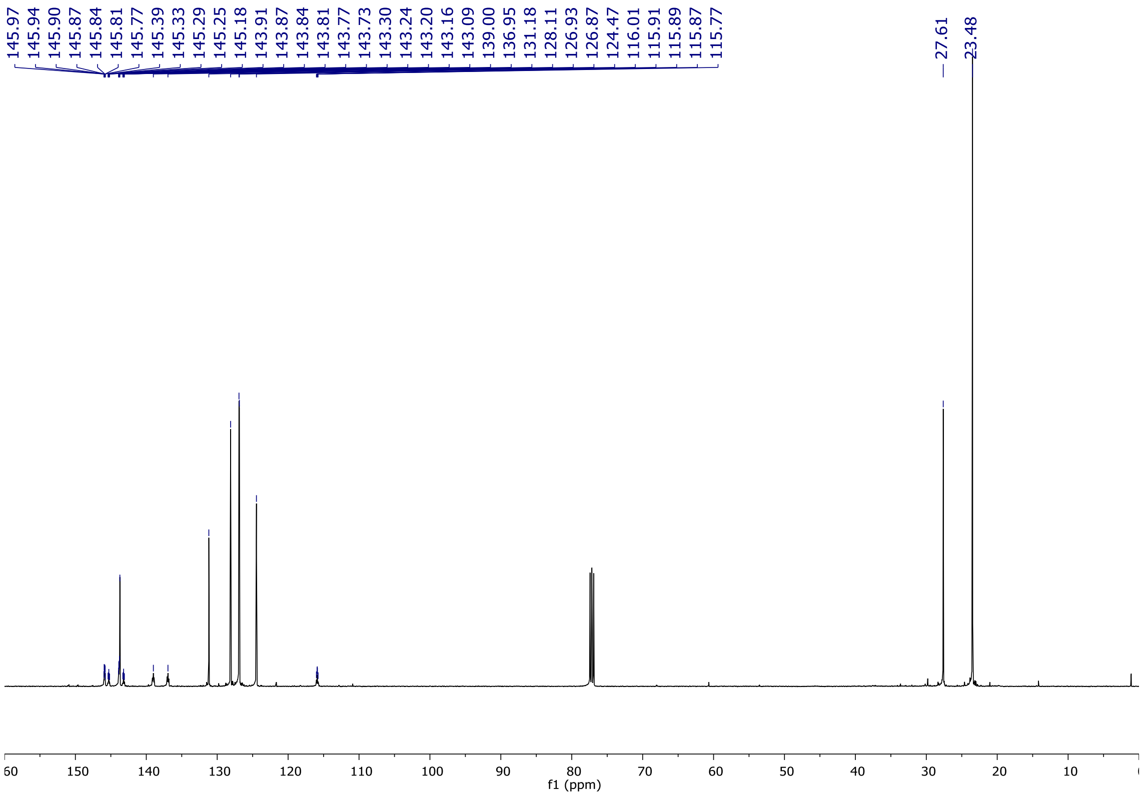

^{1}H NMR (300 MHz, $CDCl_3$)

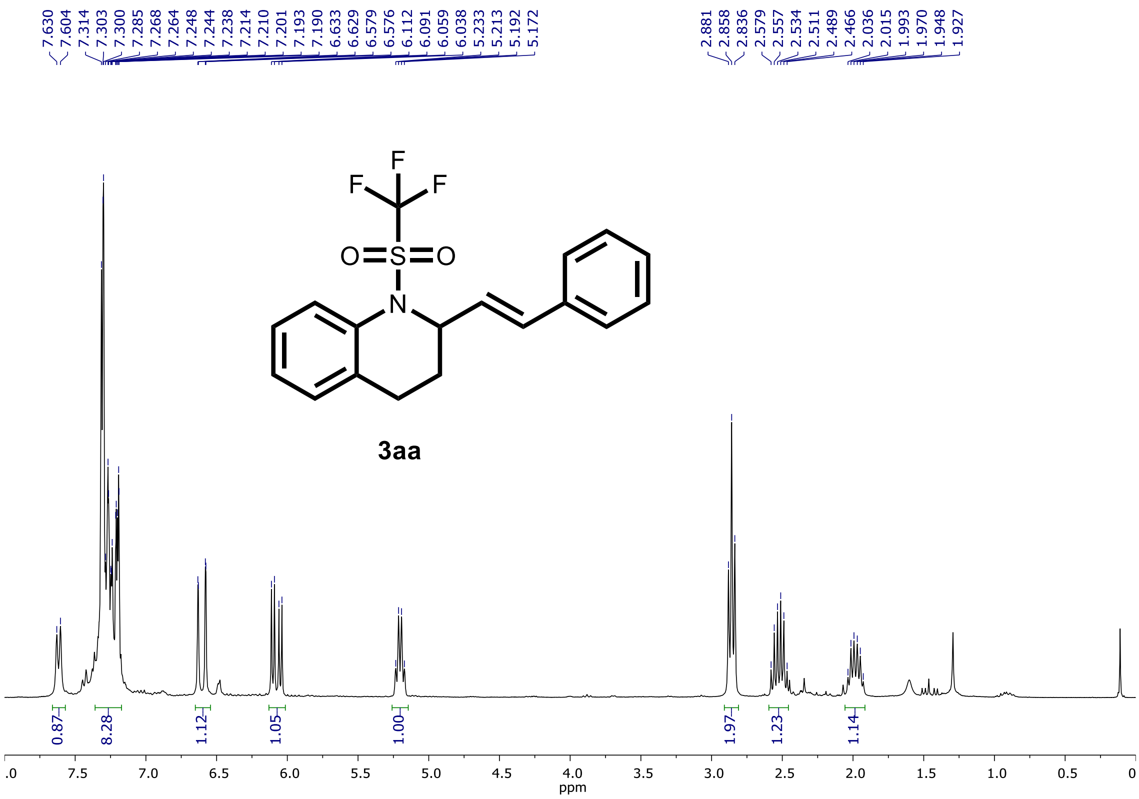

^{19}F NMR (282 MHz, $CDCl_3$)

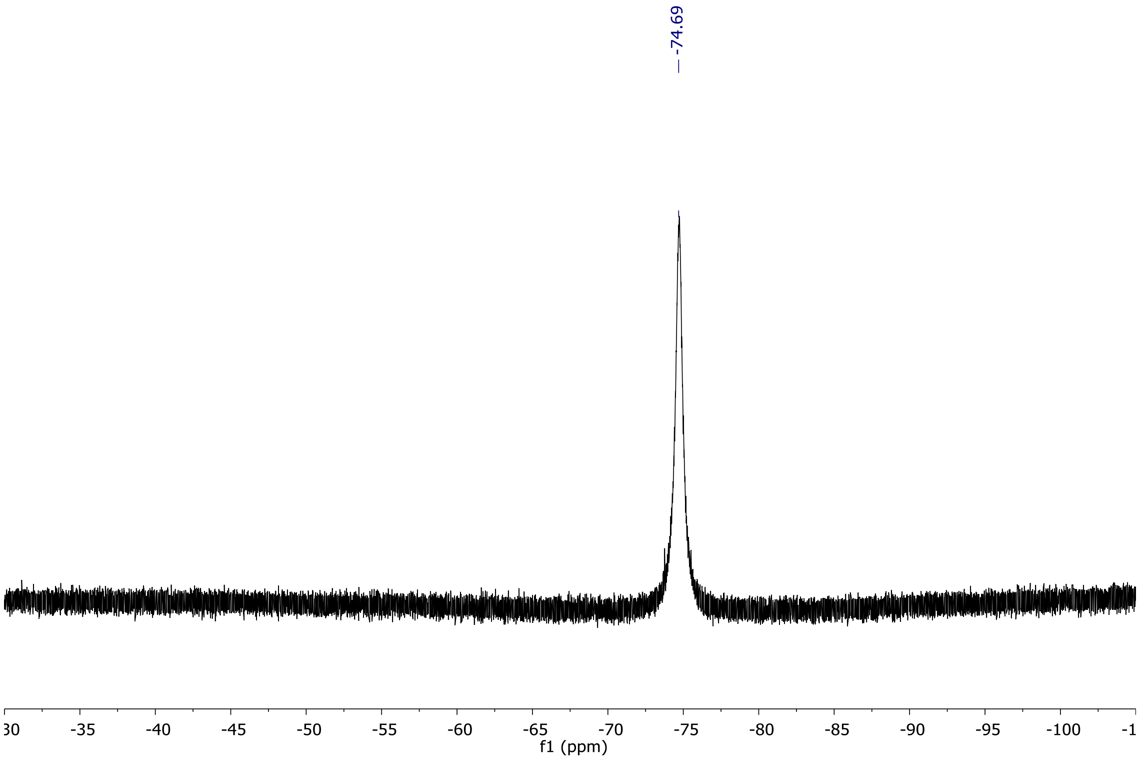

DEPT-135

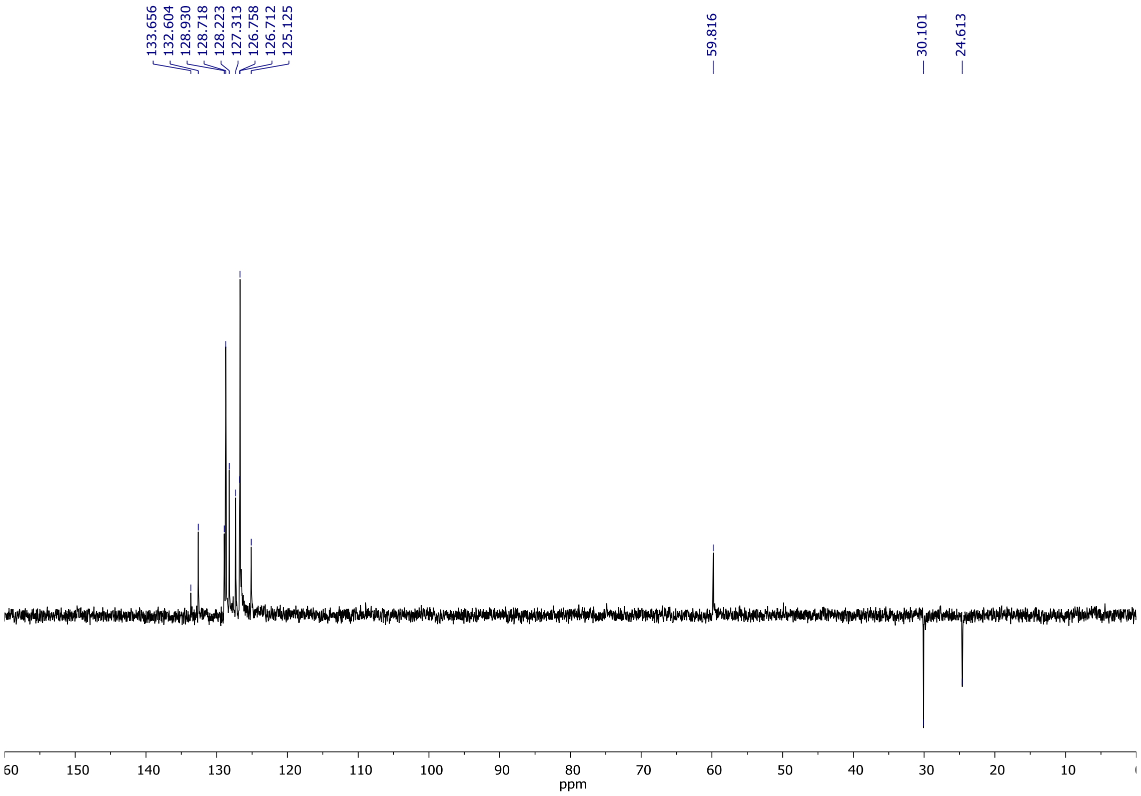

^{13}C NMR (75 MHz, $CDCl_3$)

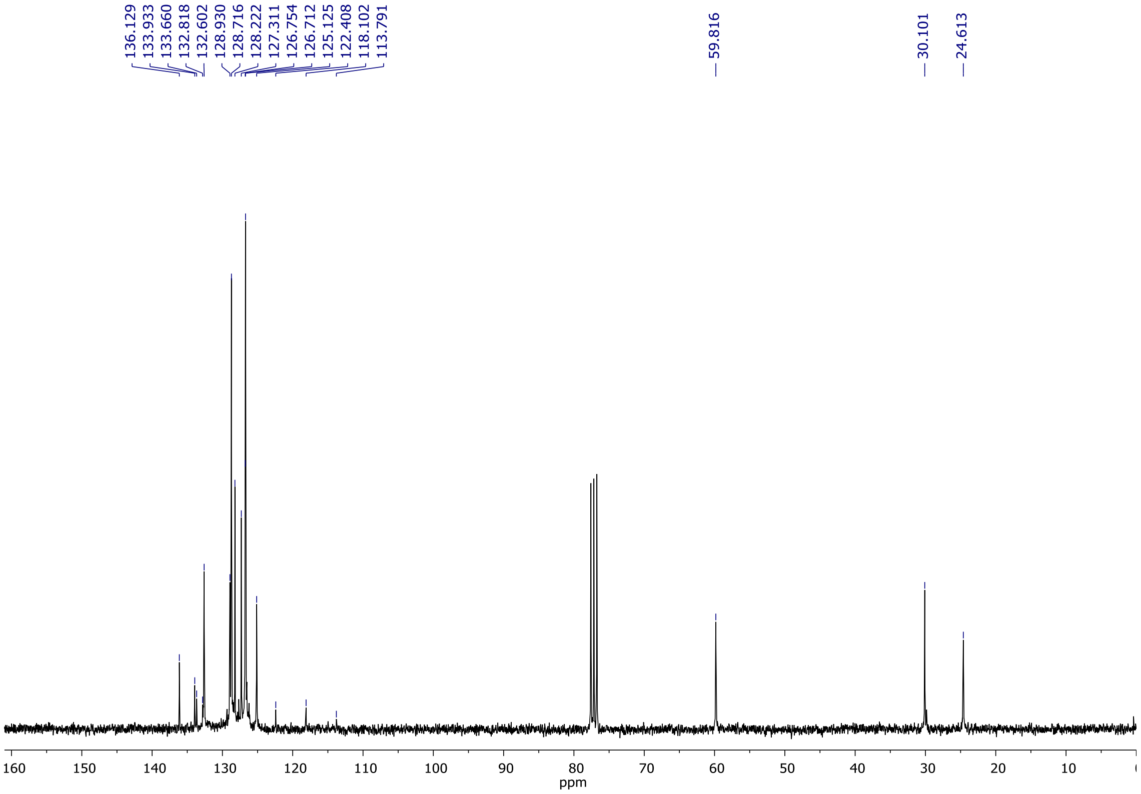

^{1}H NMR (500 MHz, $CDCl_3$)

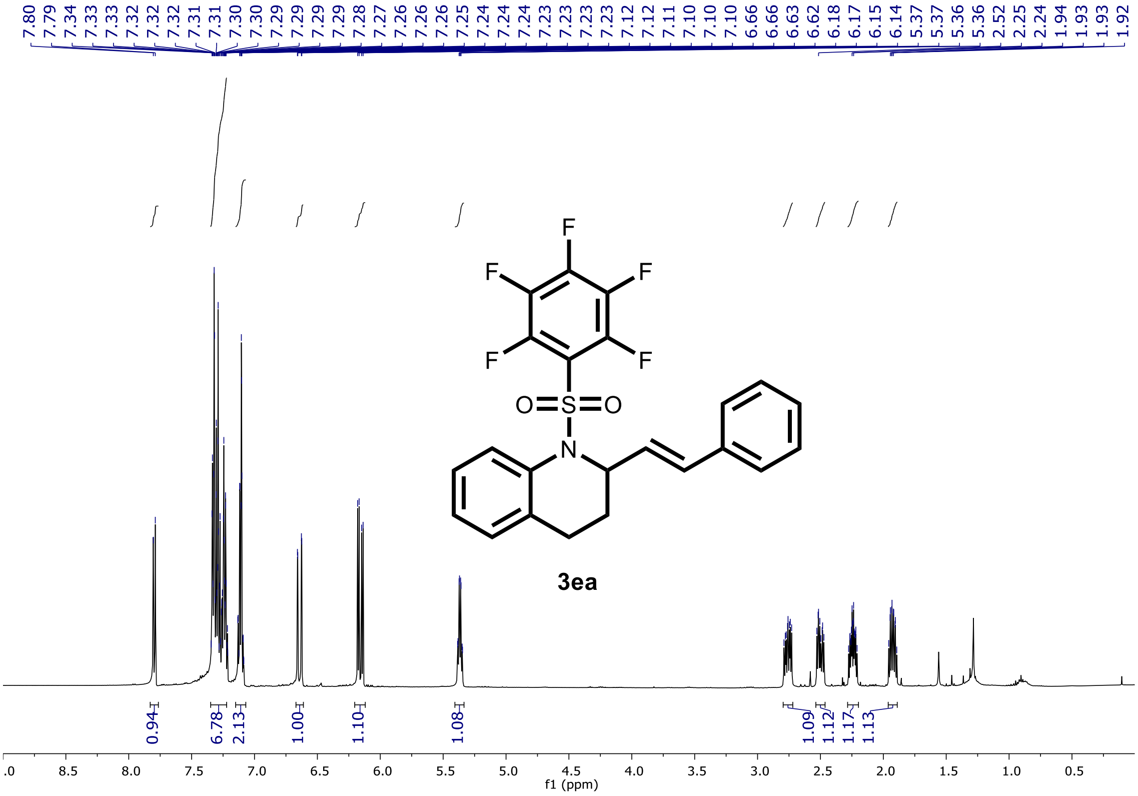

^{19}F NMR (471 MHz, $CDCl_3$)

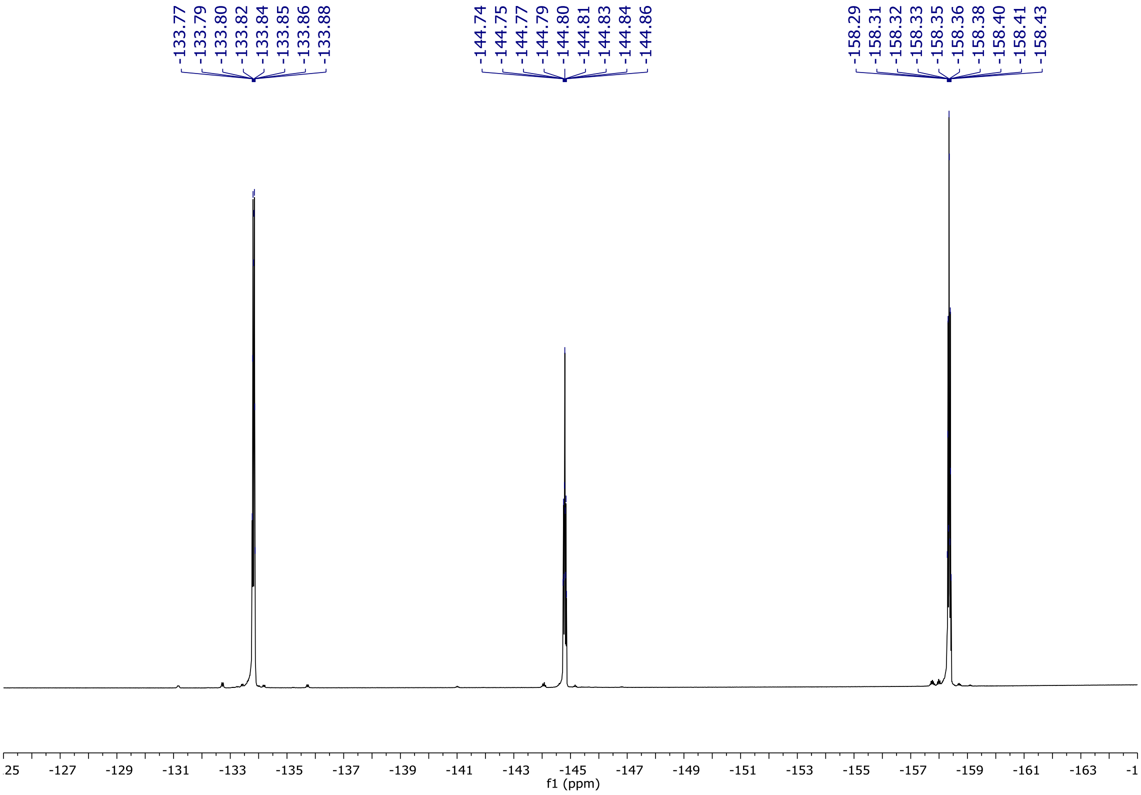

DEPT-135

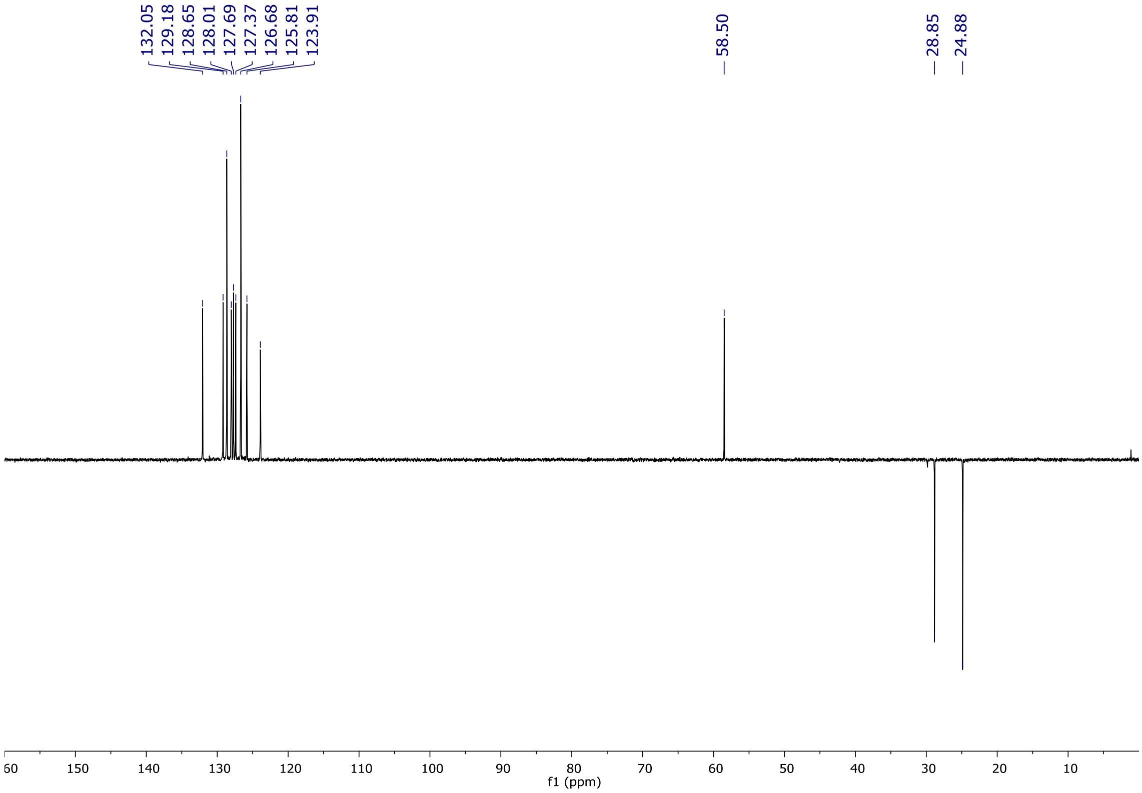

^{13}C NMR (126 MHz, $CDCl_3$)

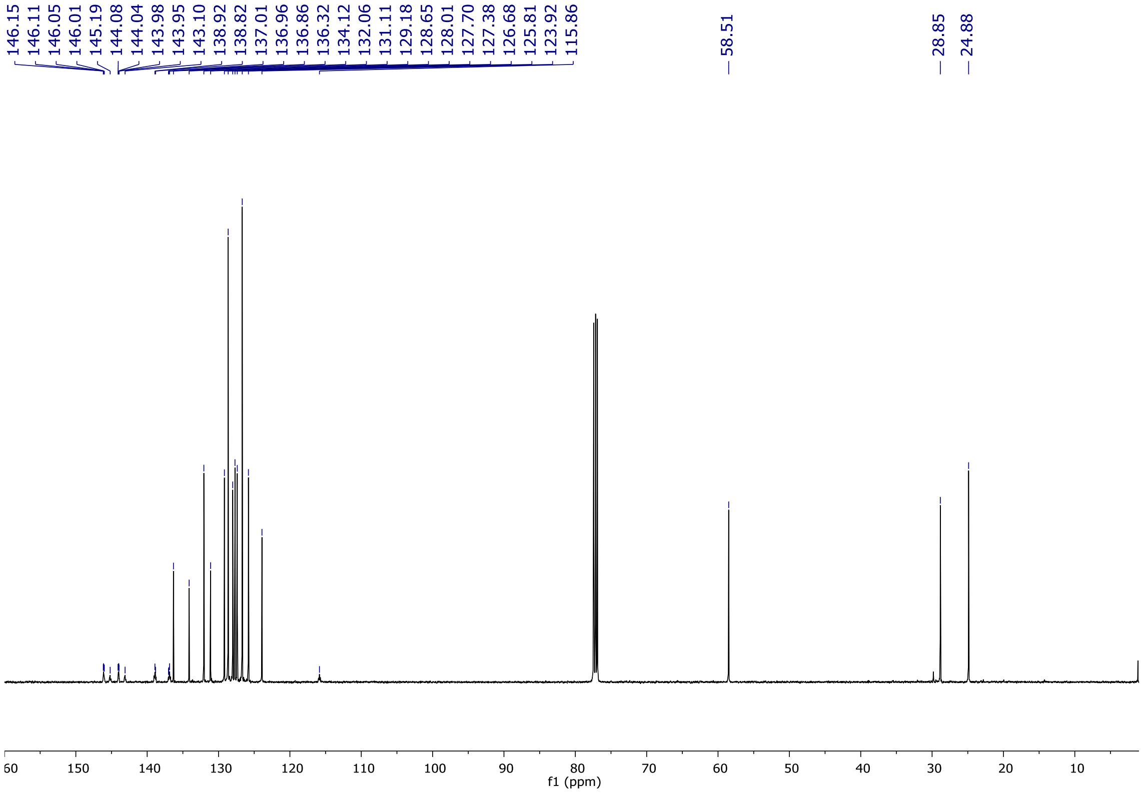

^{1}H NMR (300 MHz, $CDCl_3$)

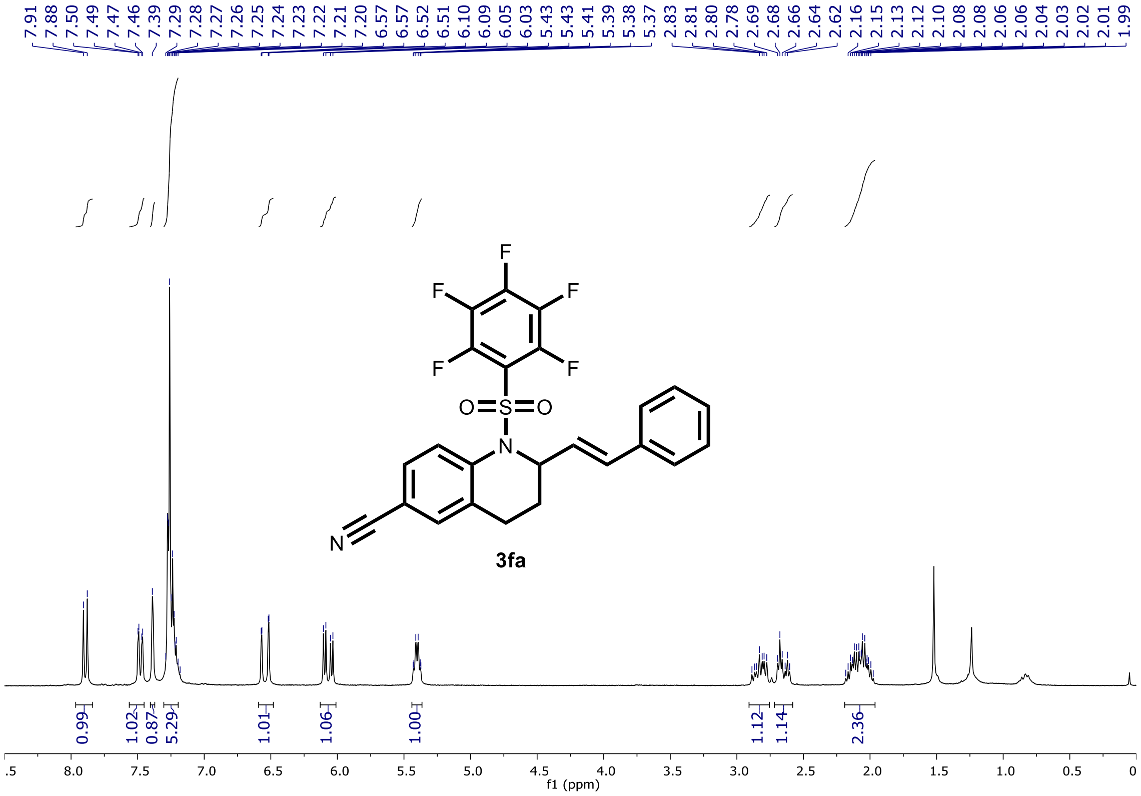

^{19}F NMR (471 MHz, $CDCl_3$)

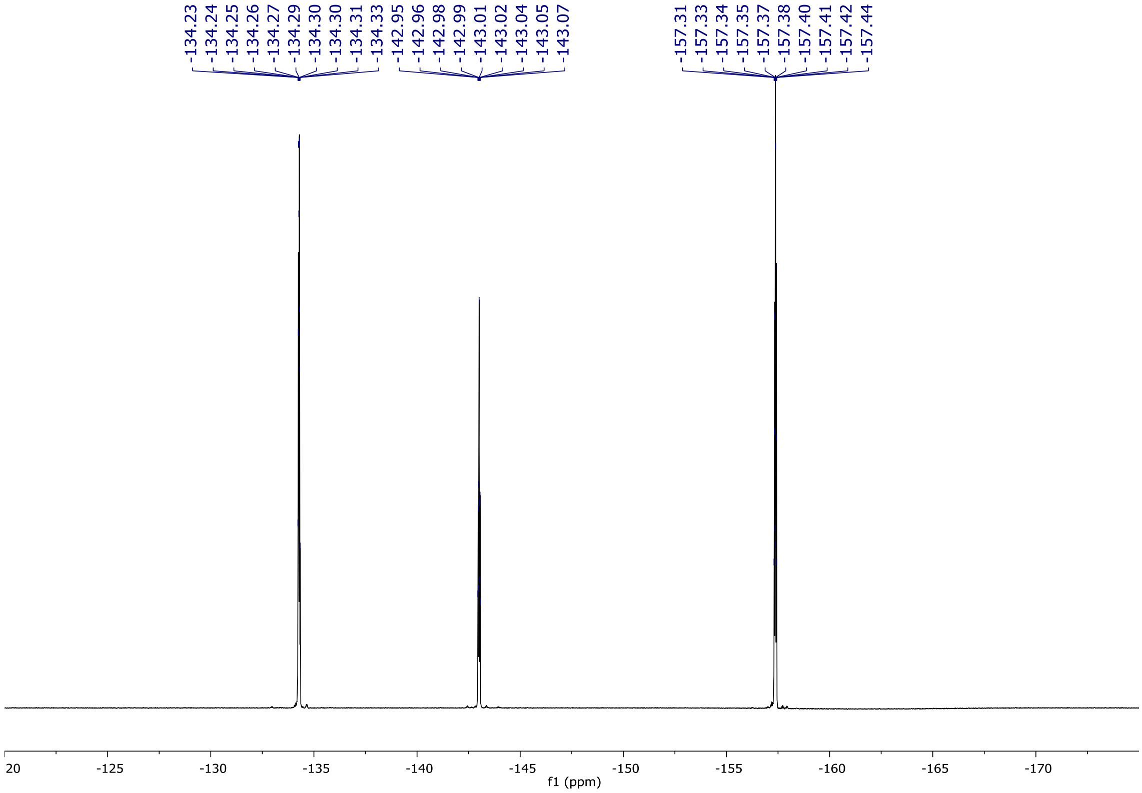

DEPT-135

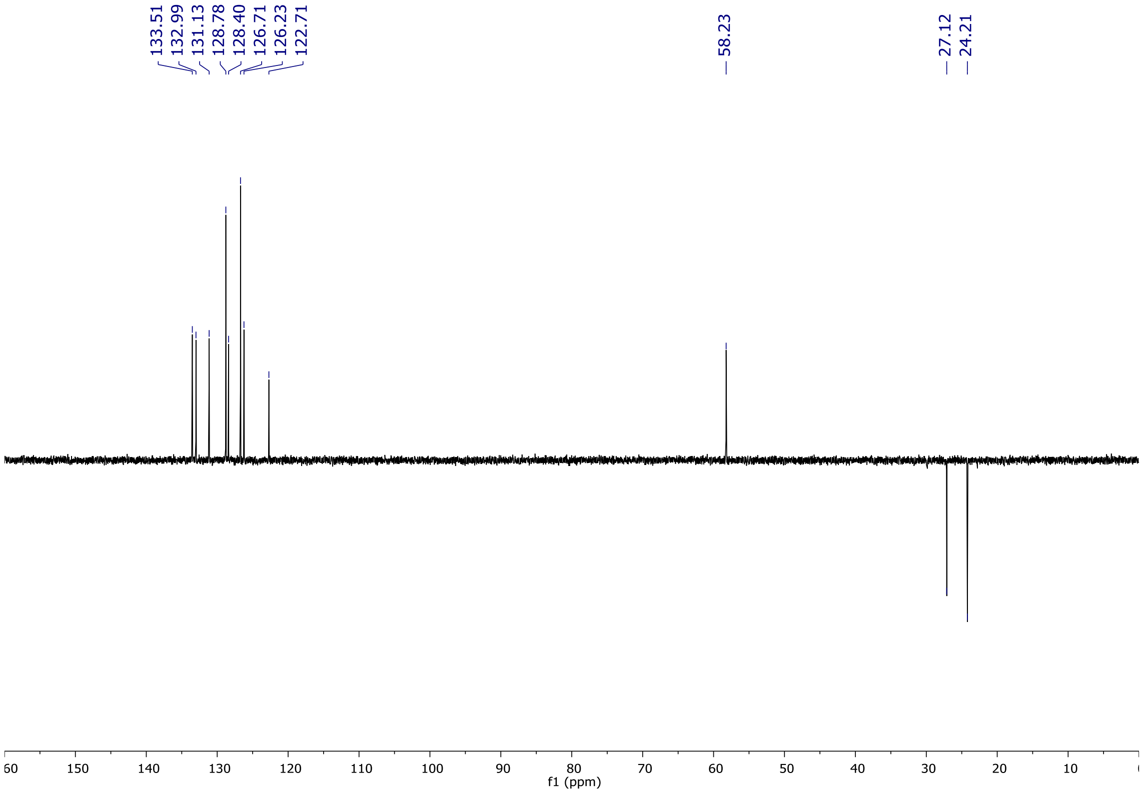

^{13}C NMR (126 MHz, $CDCl_3$)

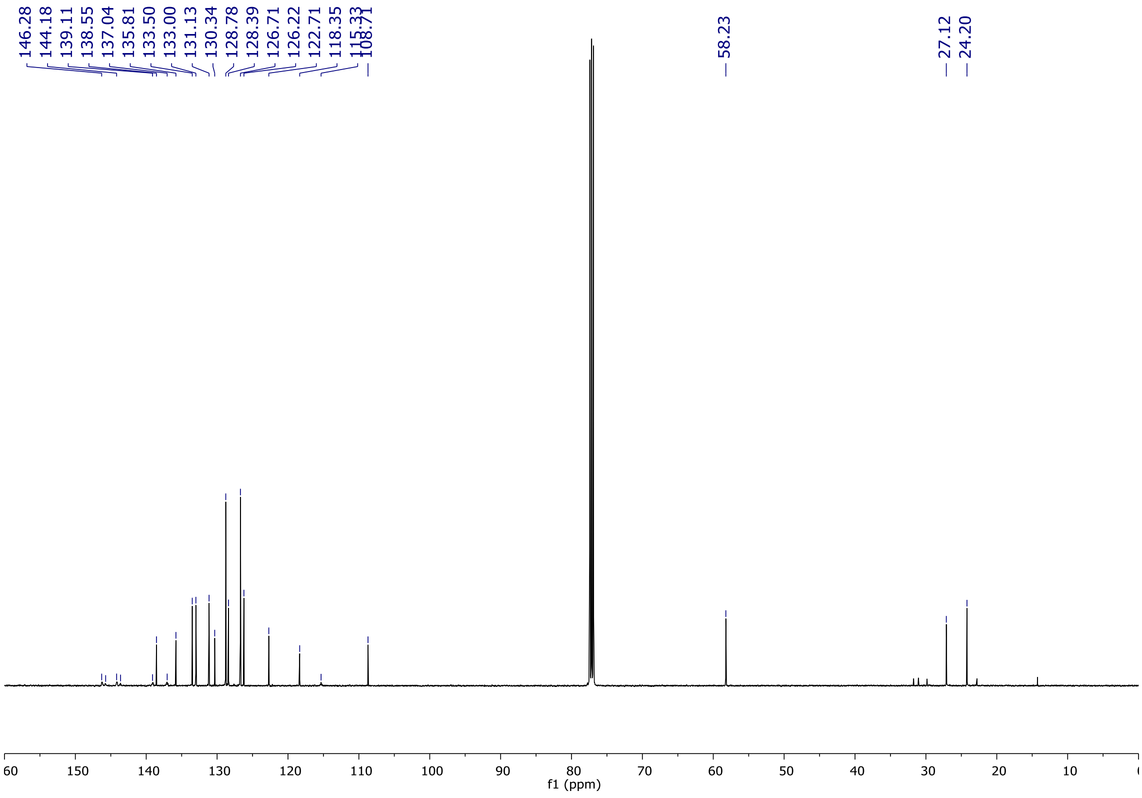

^{1}H NMR (500 MHz, $CDCl_3$)

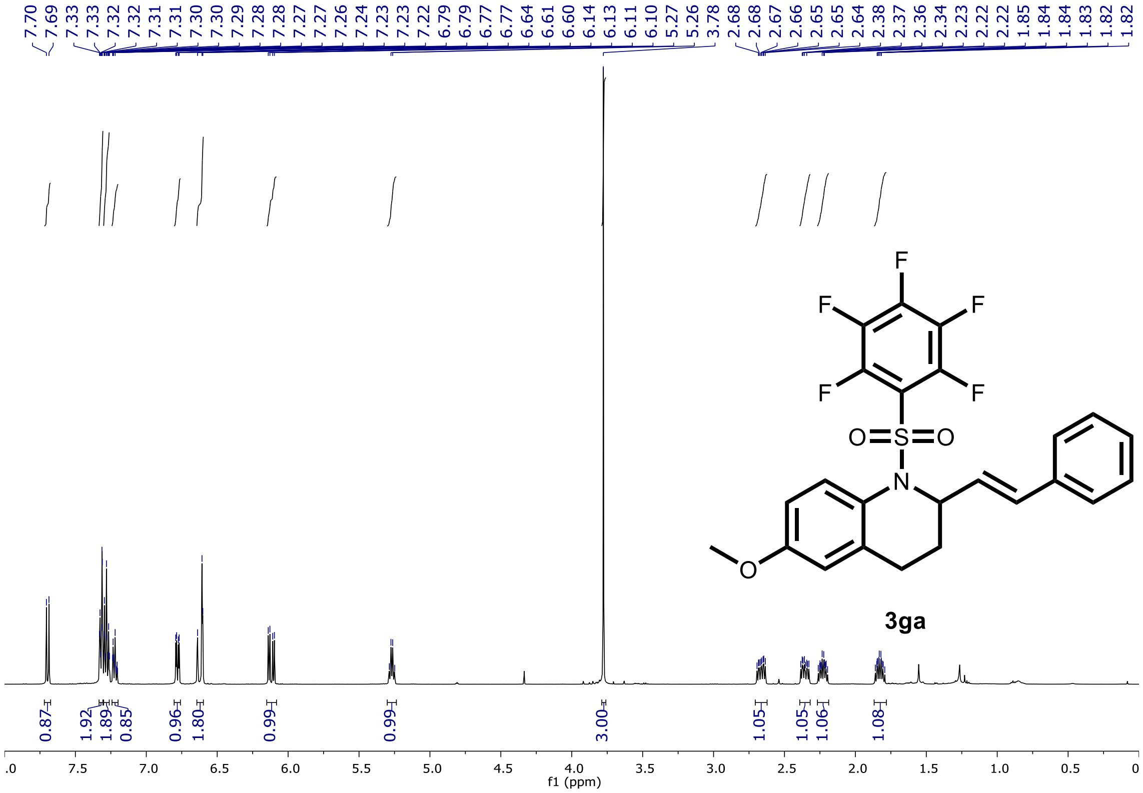

^{19}F NMR (471 MHz, $CDCl_3$)

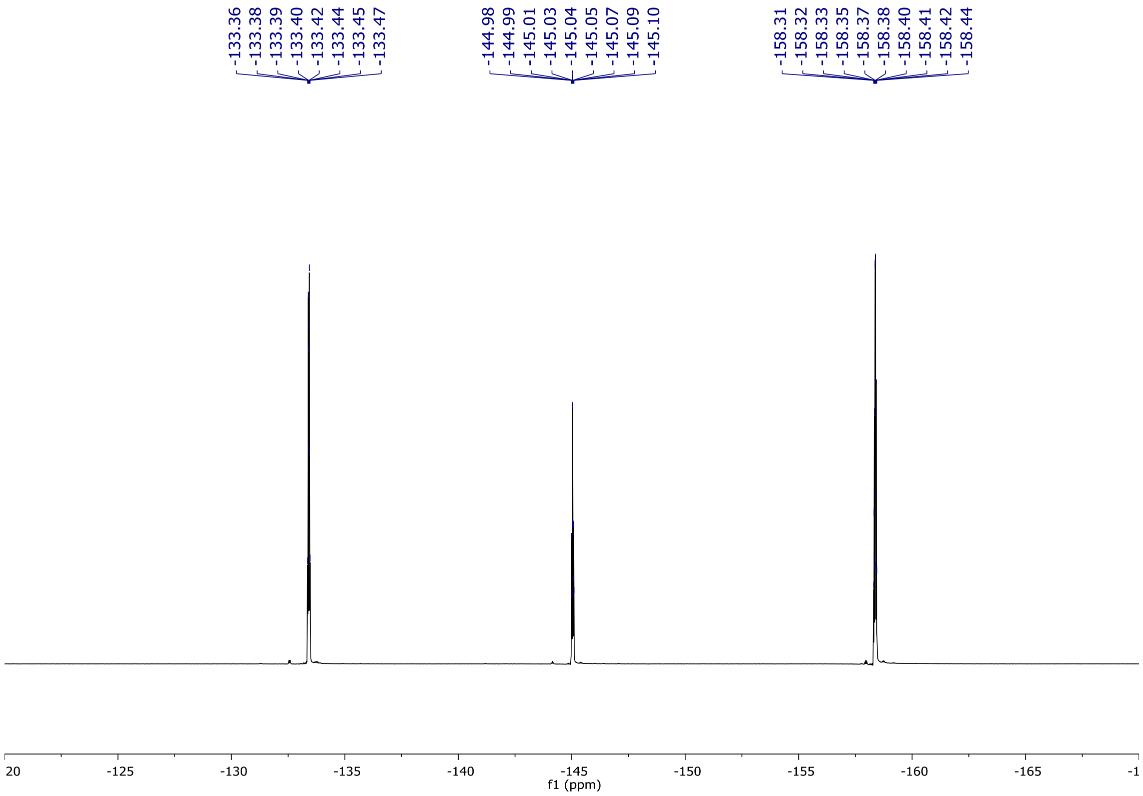

DEPT-135

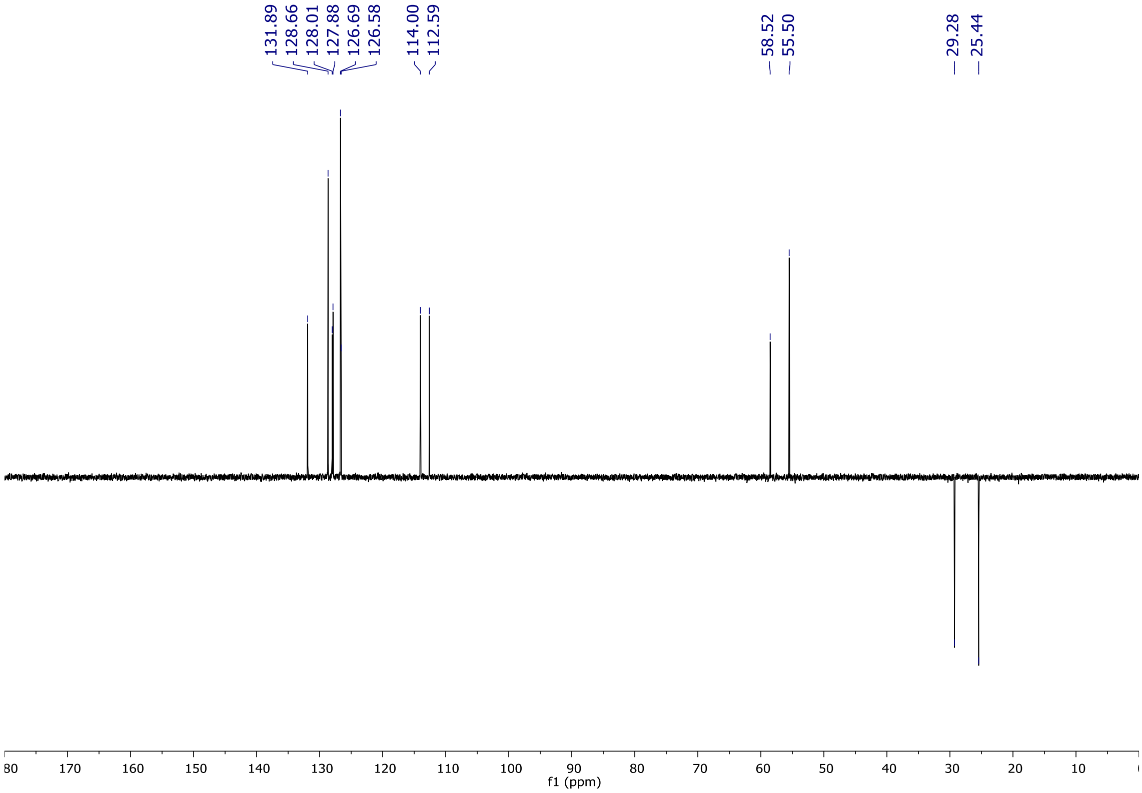

^{13}C NMR (126 MHz, $CDCl_3$)

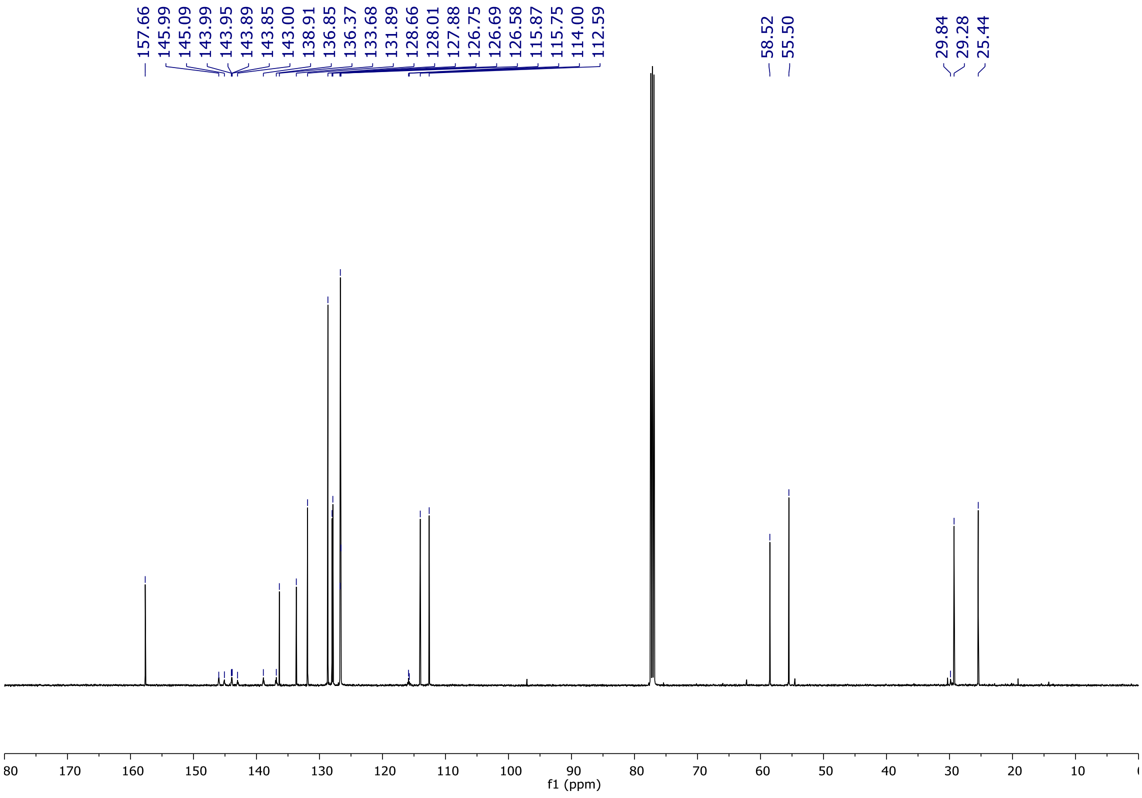

^{1}H NMR (500 MHz, $CDCl_3$)

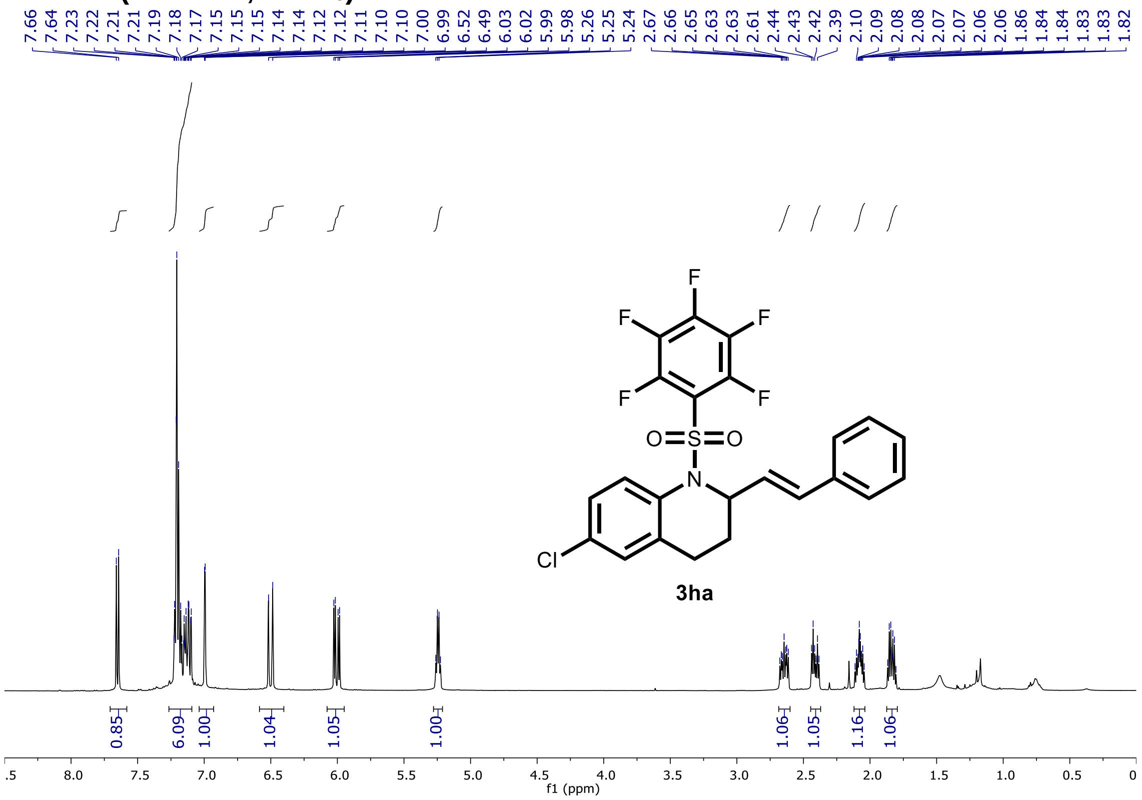

^{19}F NMR (471 MHz, $CDCl_3$)

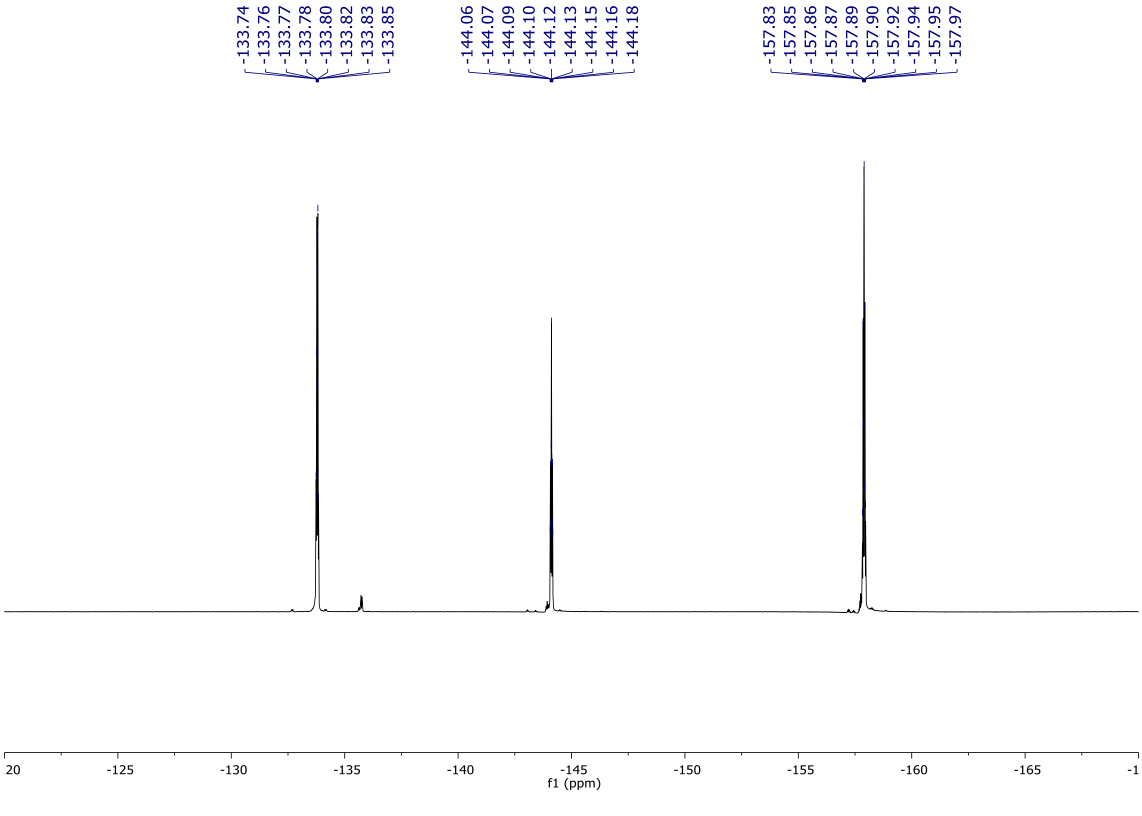

DEPT-135

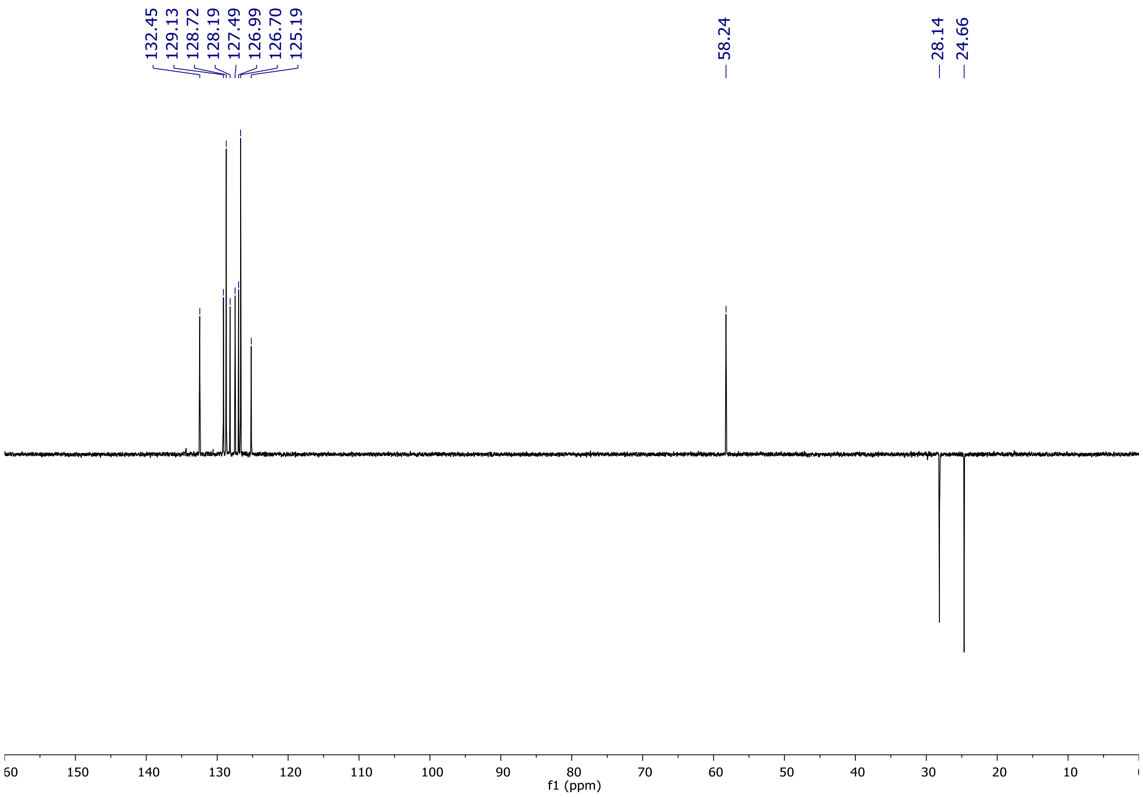

^{13}C NMR (126 MHz, $CDCl_3$)

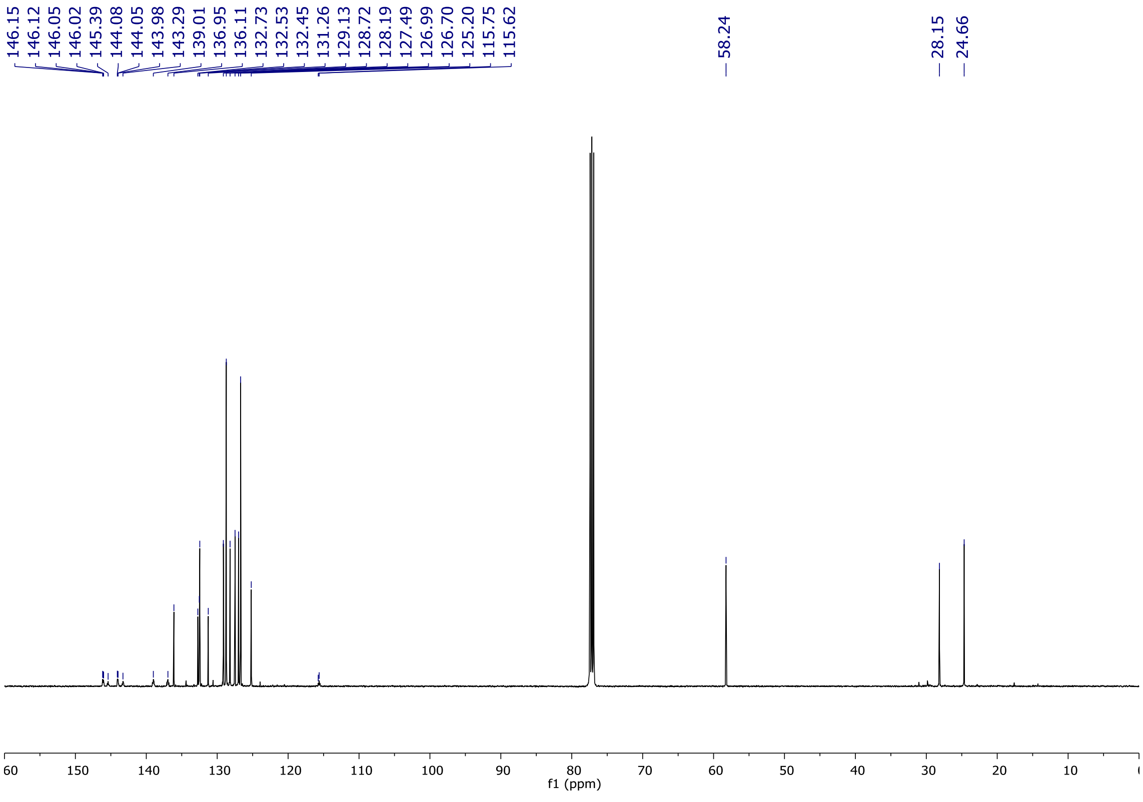

^{1}H NMR (500 MHz, $CDCl_3$)

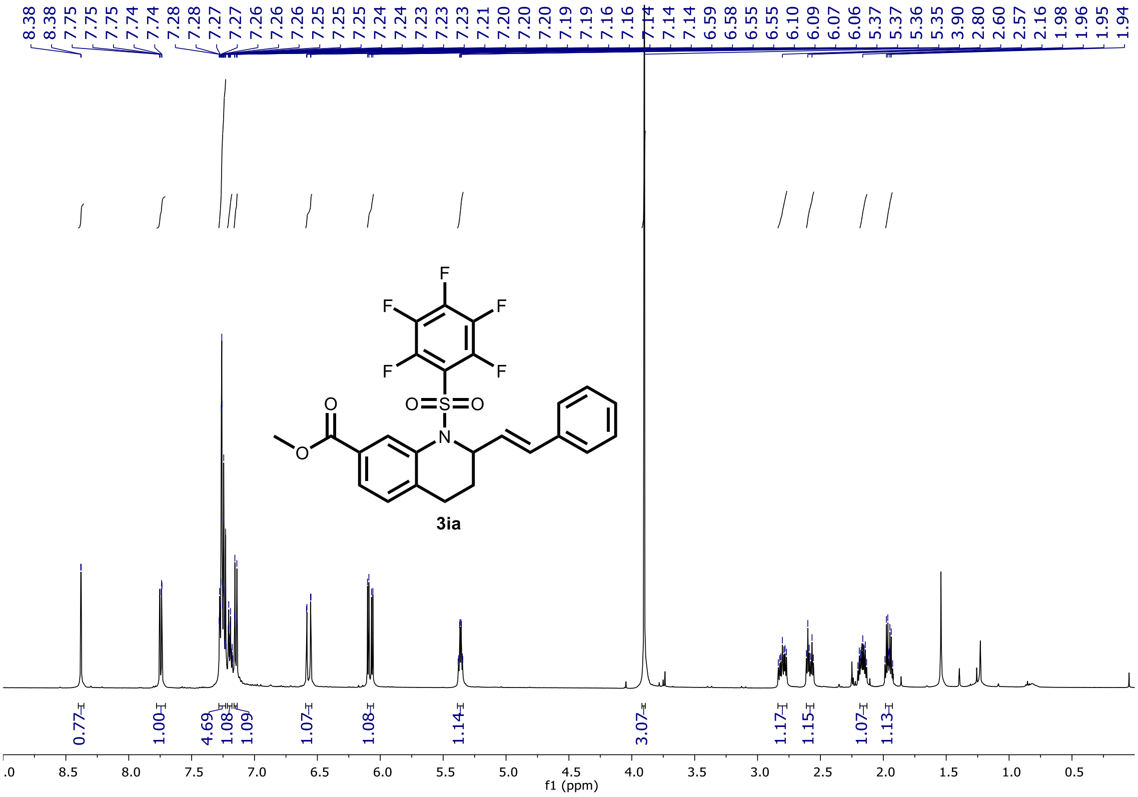

^{19}F NMR (282 MHz, $CDCl_3$)

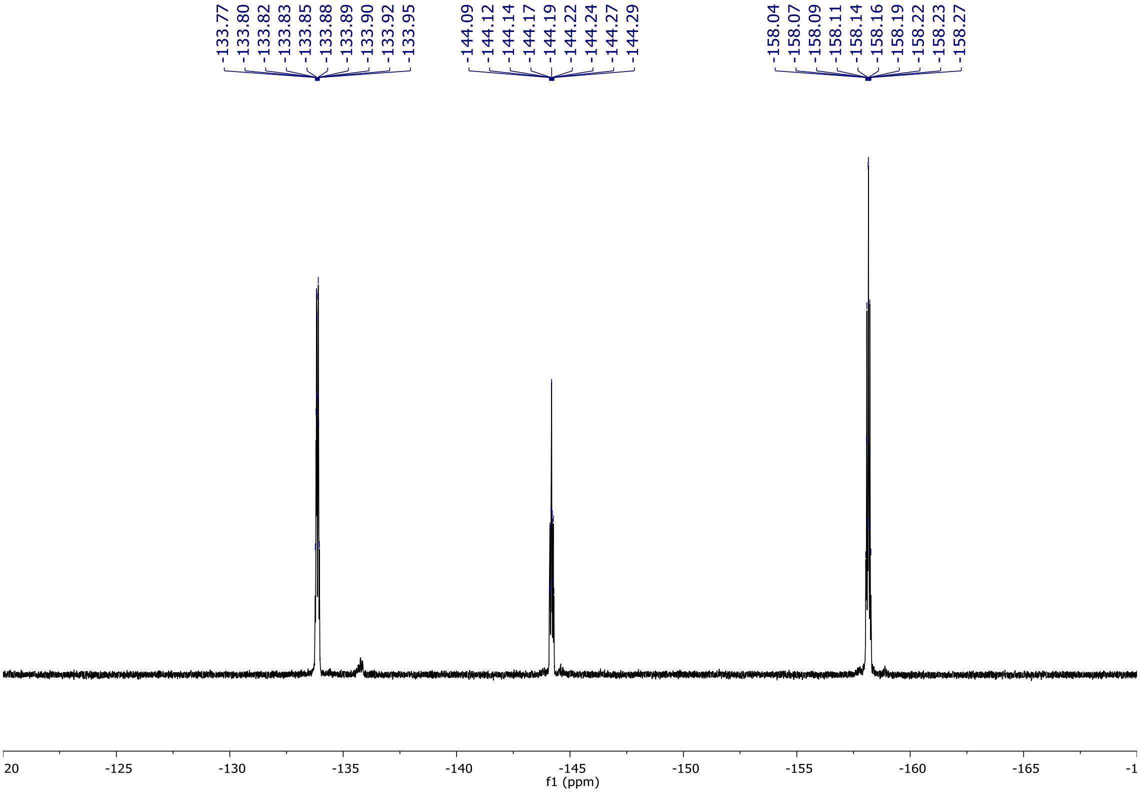

DEPT-135

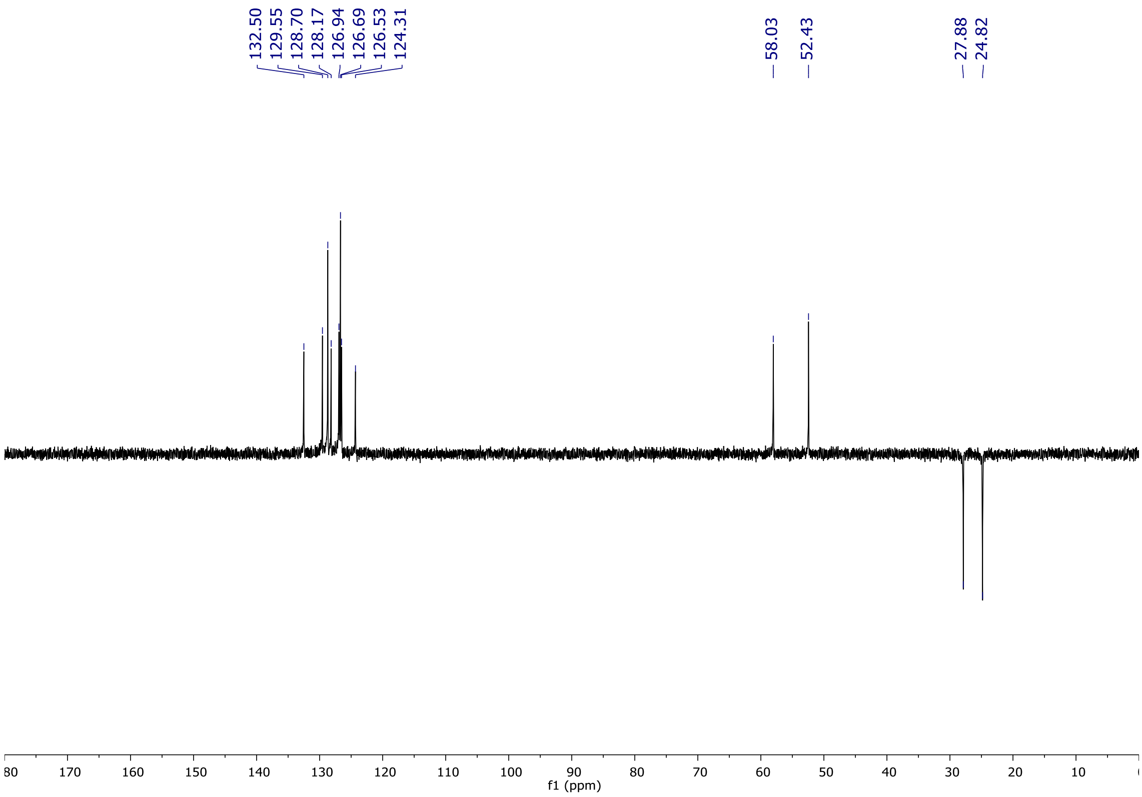

^{13}C NMR (126 MHz, $CDCl_3$)

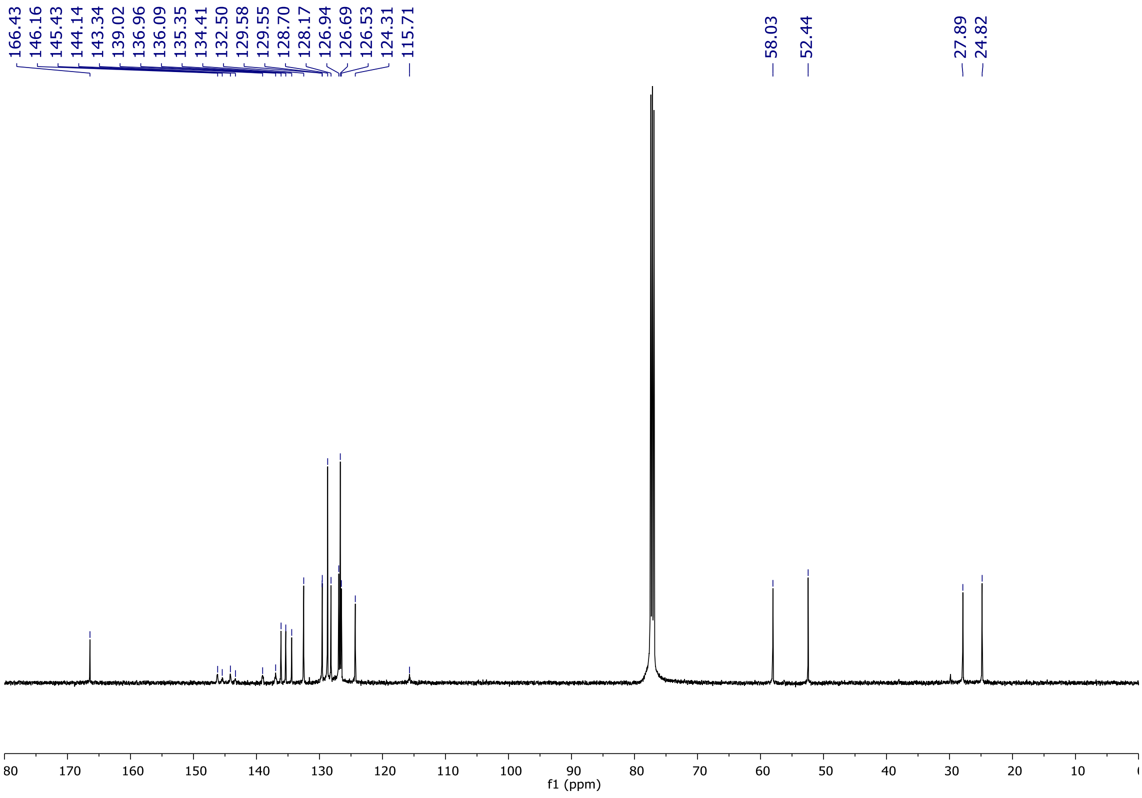

^{1}H NMR (500 MHz, $CDCl_3$)

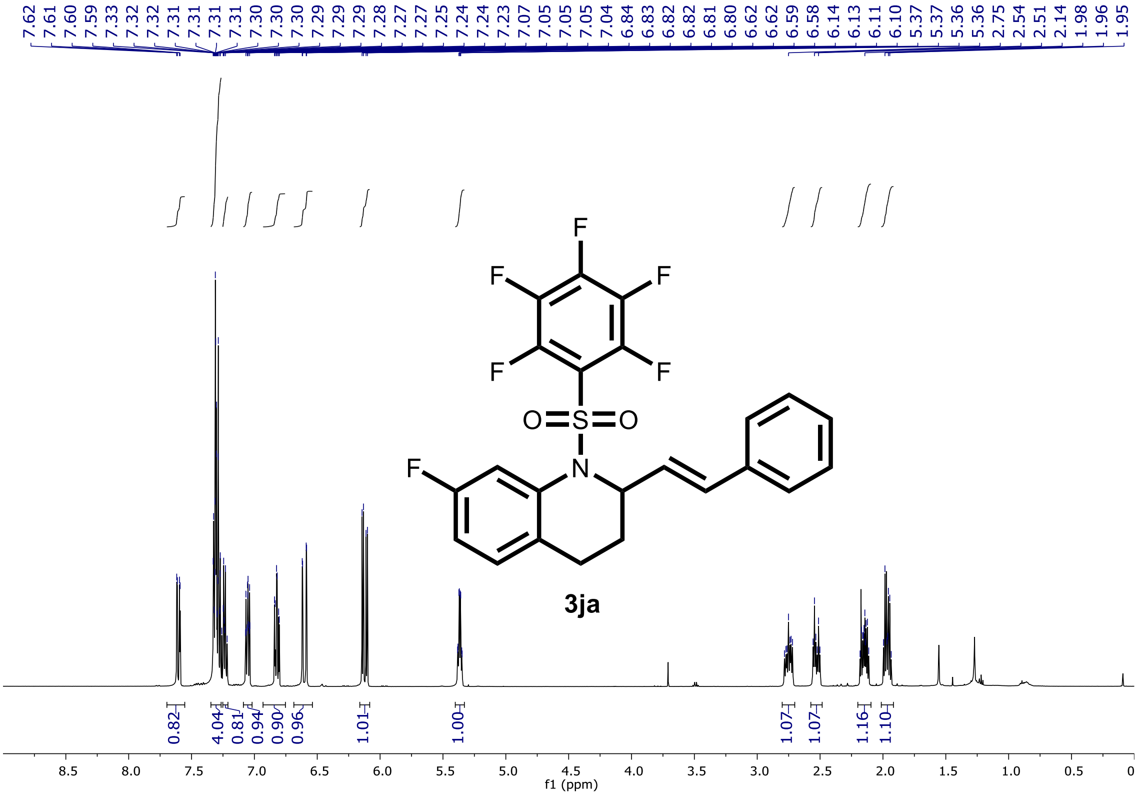

^{19}F NMR (471 MHz, $CDCl_3$)

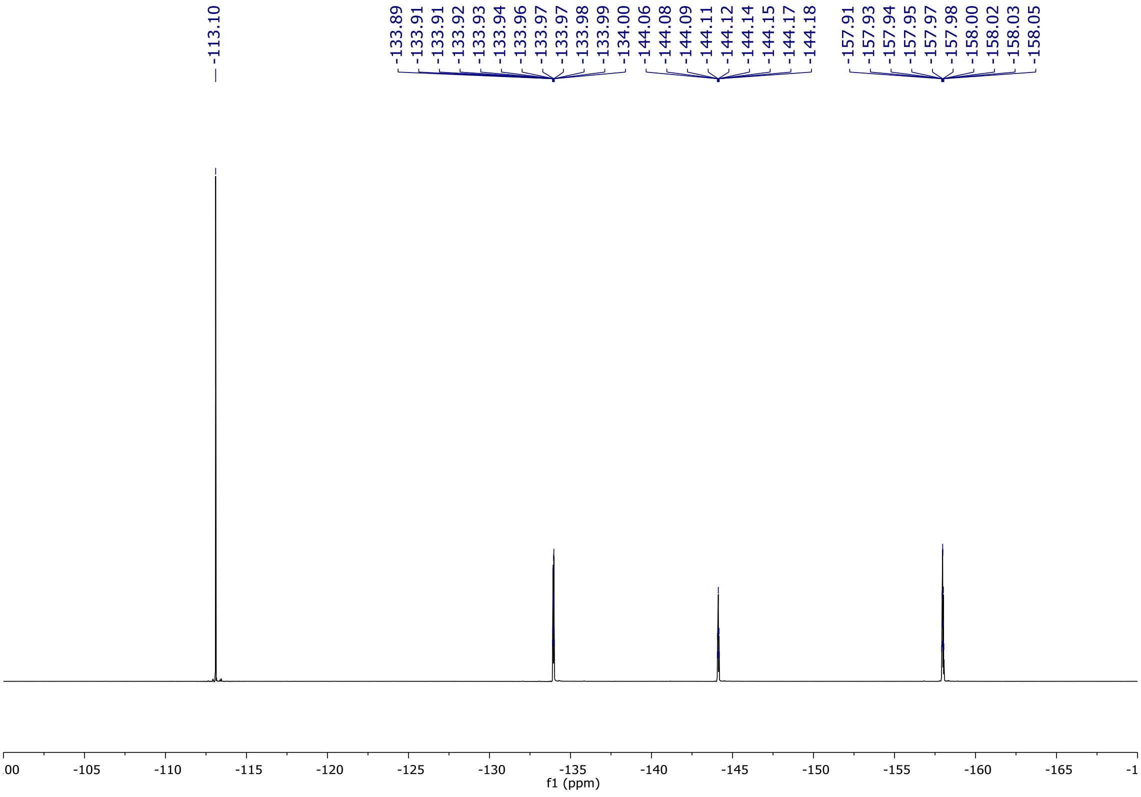

DEPT-135

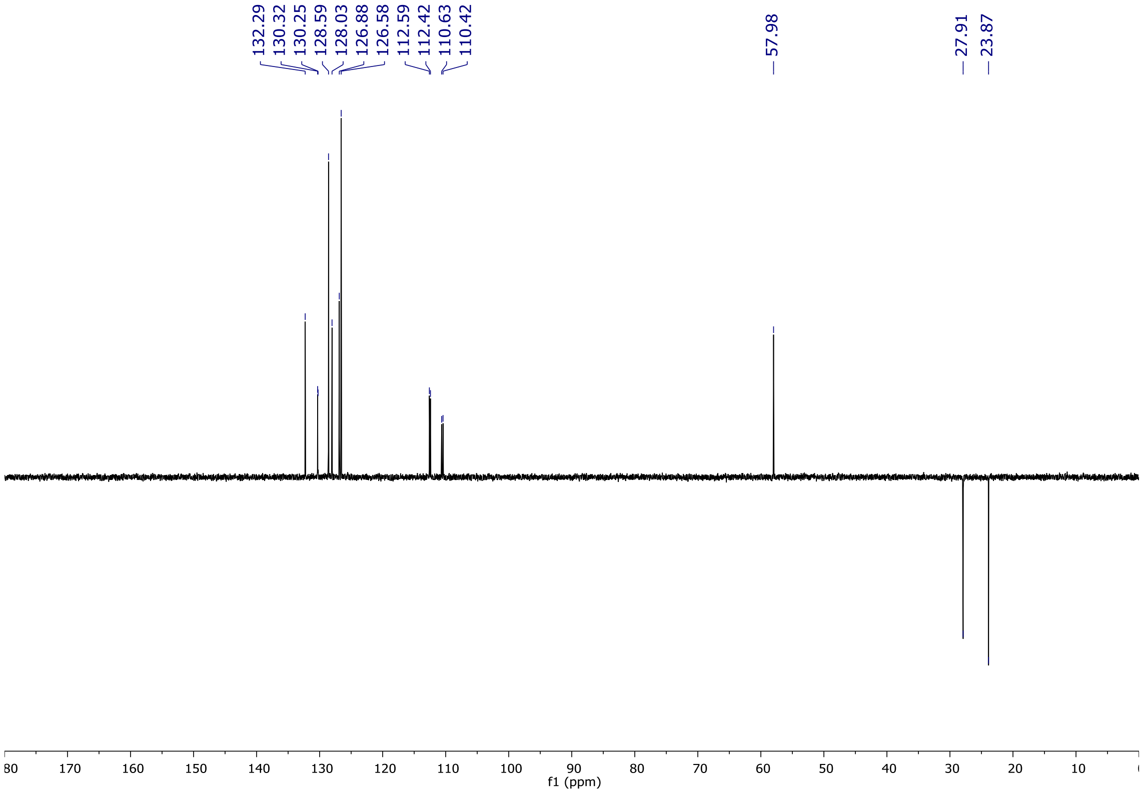

^{13}C NMR (126 MHz, $CDCl_3$)

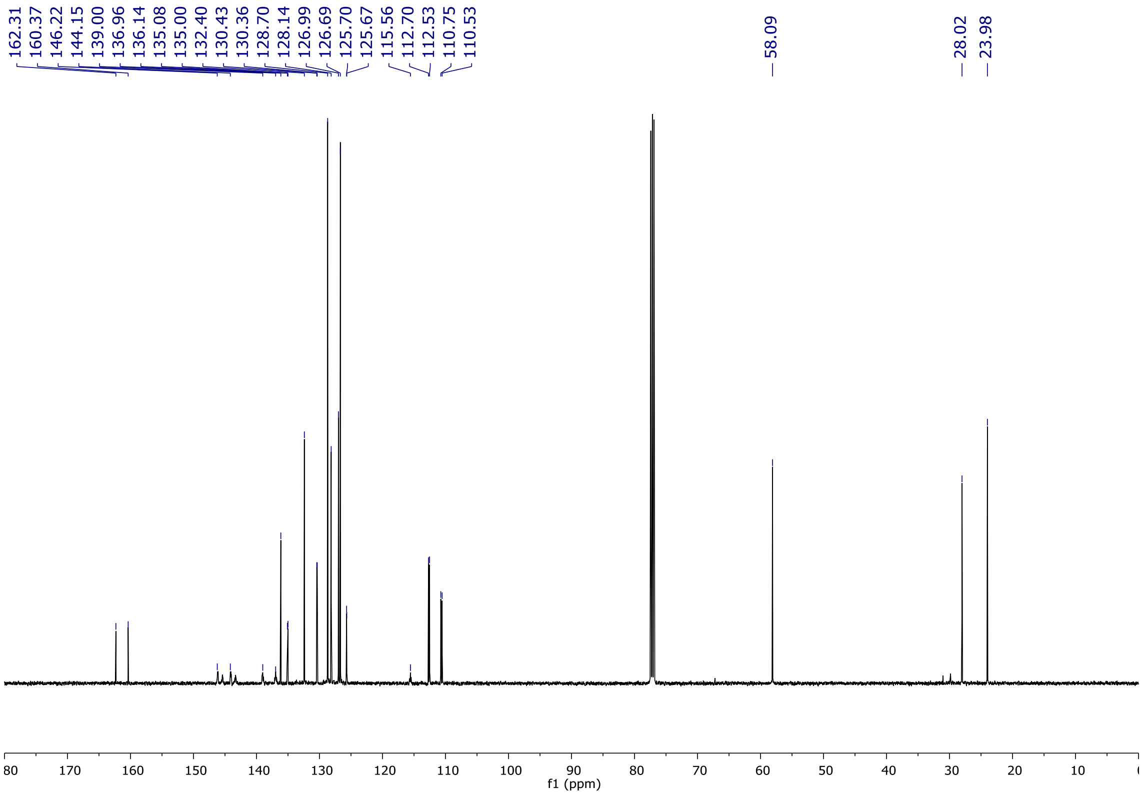

^{1}H NMR (500 MHz, $CDCl_3$)

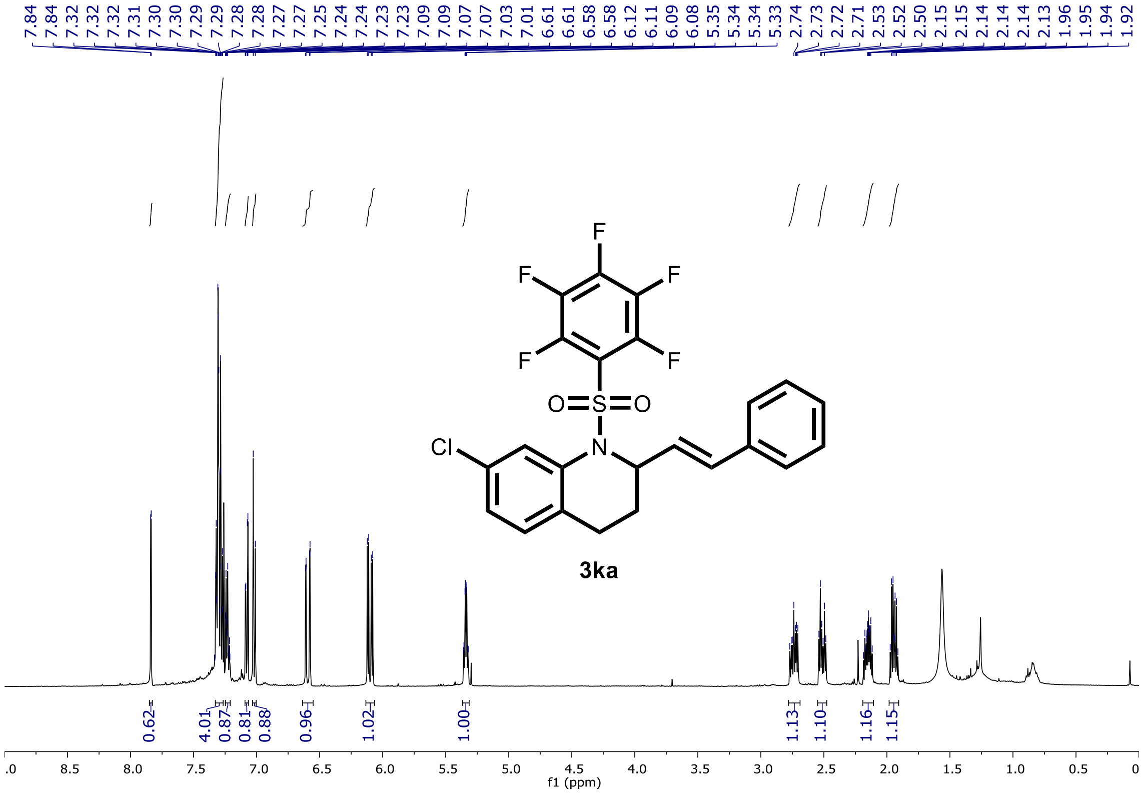

^{19}F NMR (471 MHz, $CDCl_3$)

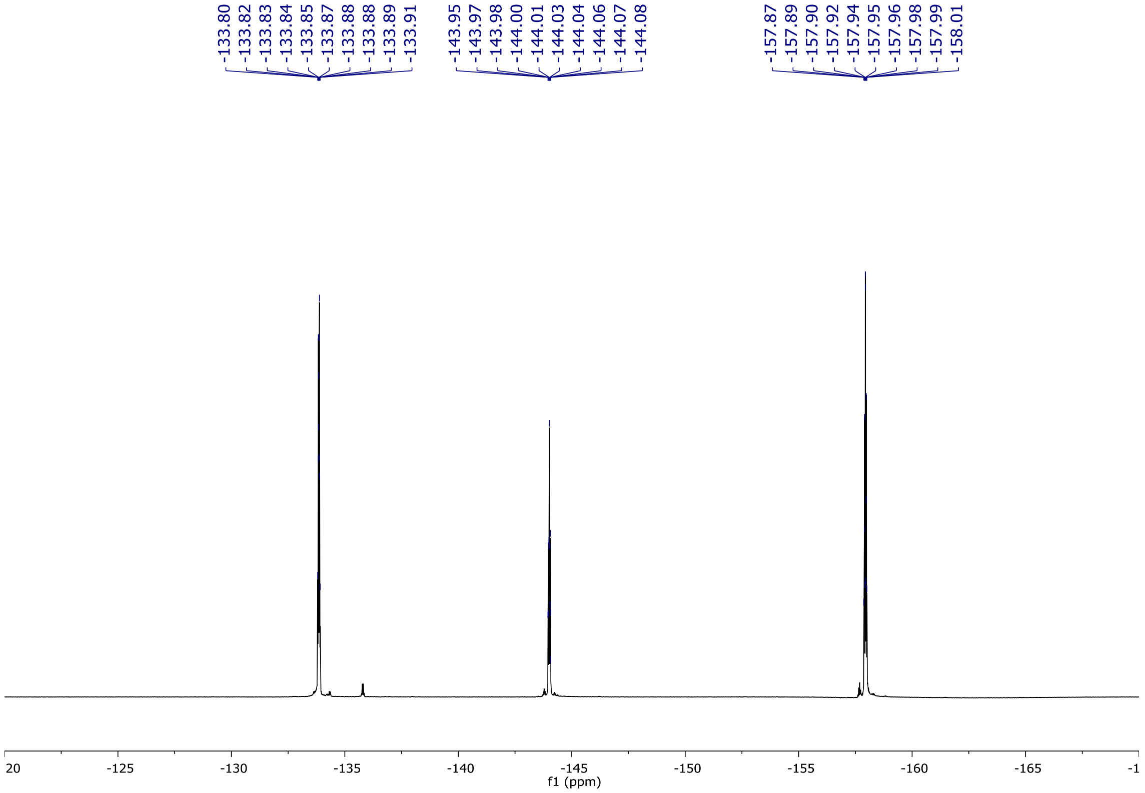

DEPT-135

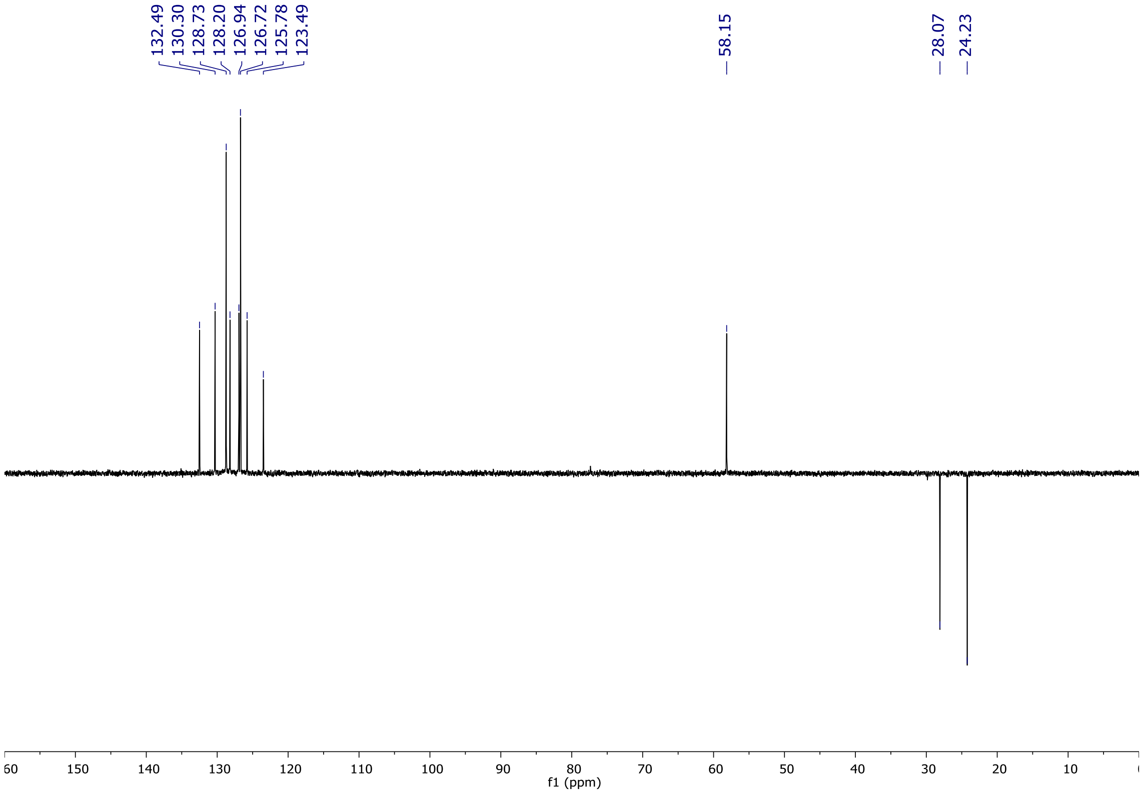

^{13}C NMR (126 MHz, $CDCl_3$)

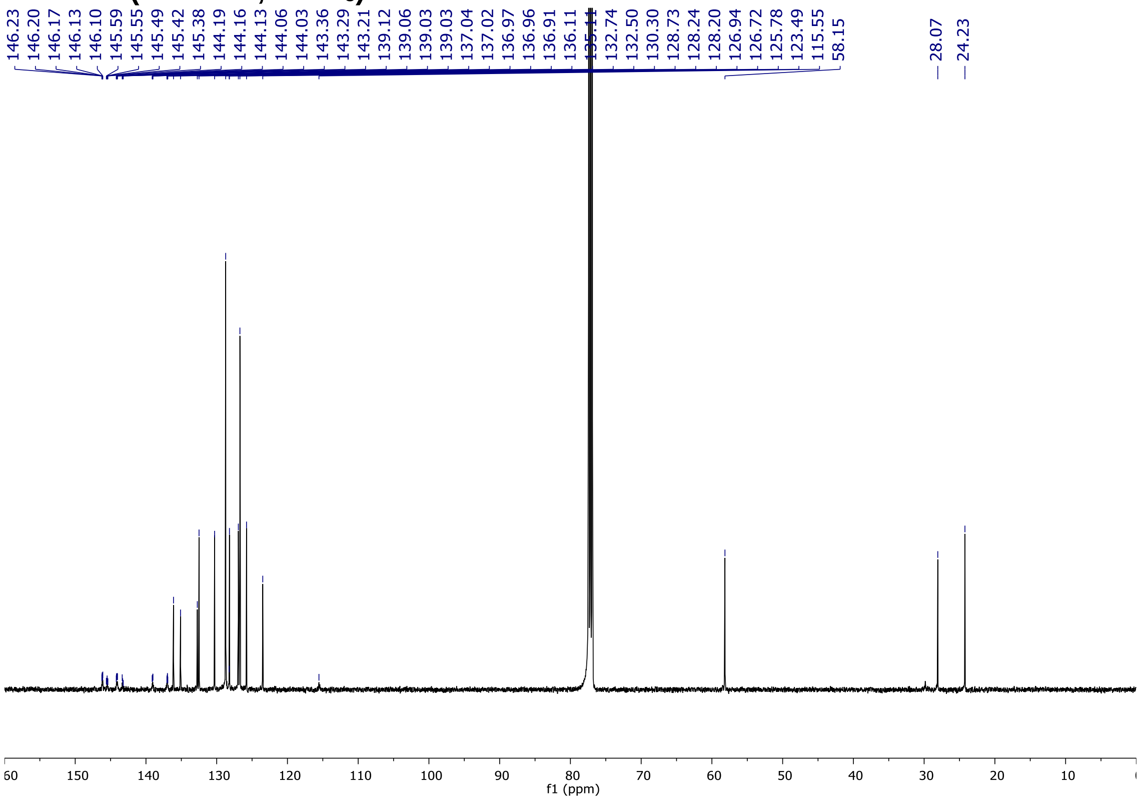

^{1}H NMR (500 MHz, $CDCl_3$)

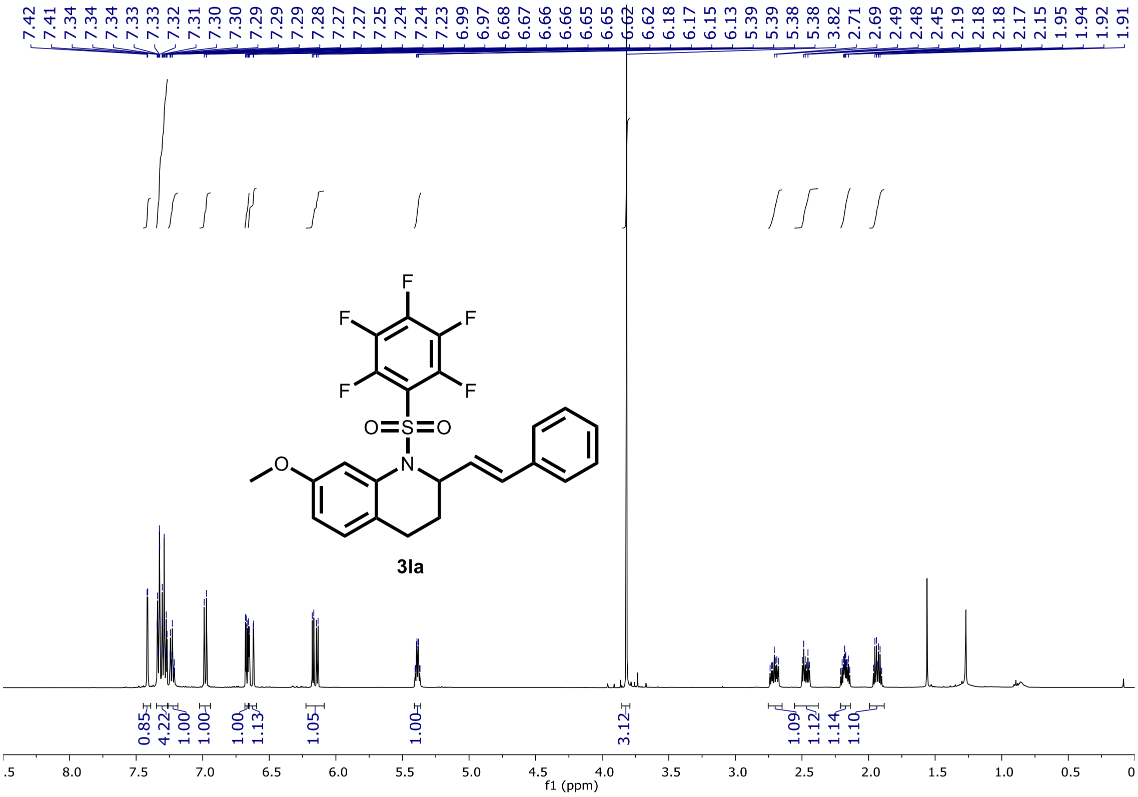

^{19}F NMR (471 MHz, $CDCl_3$)

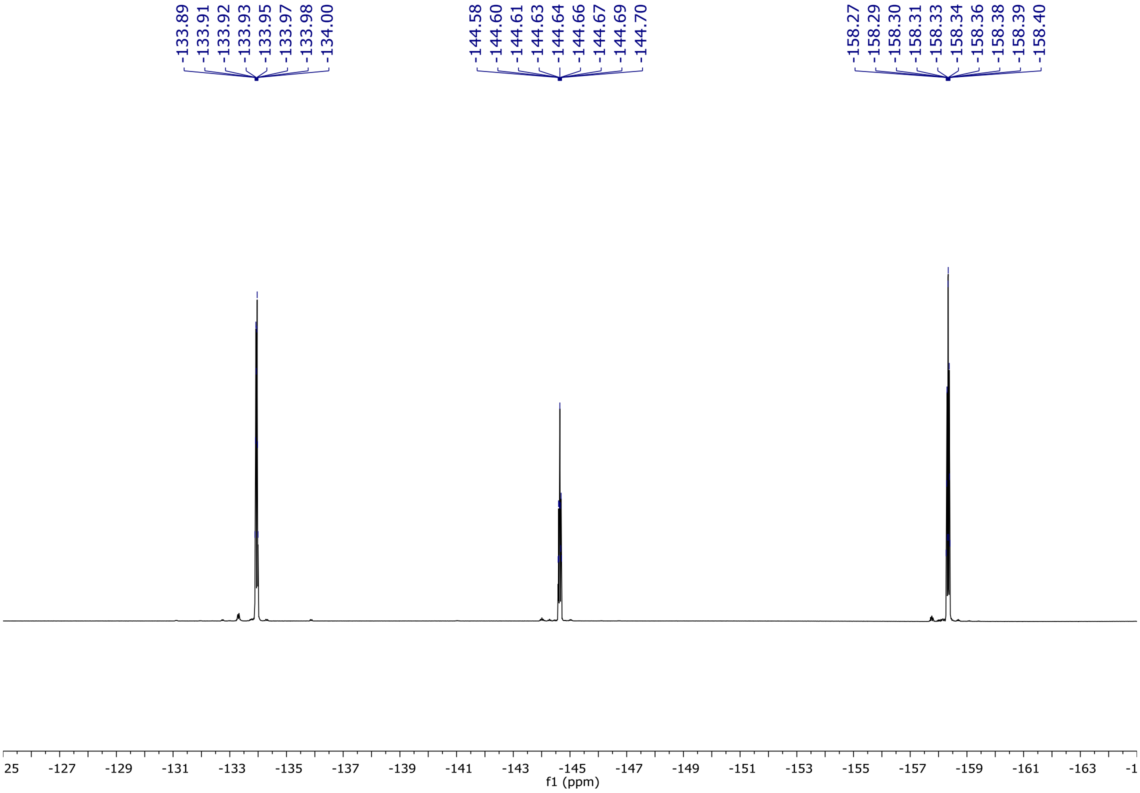

DEPT-135

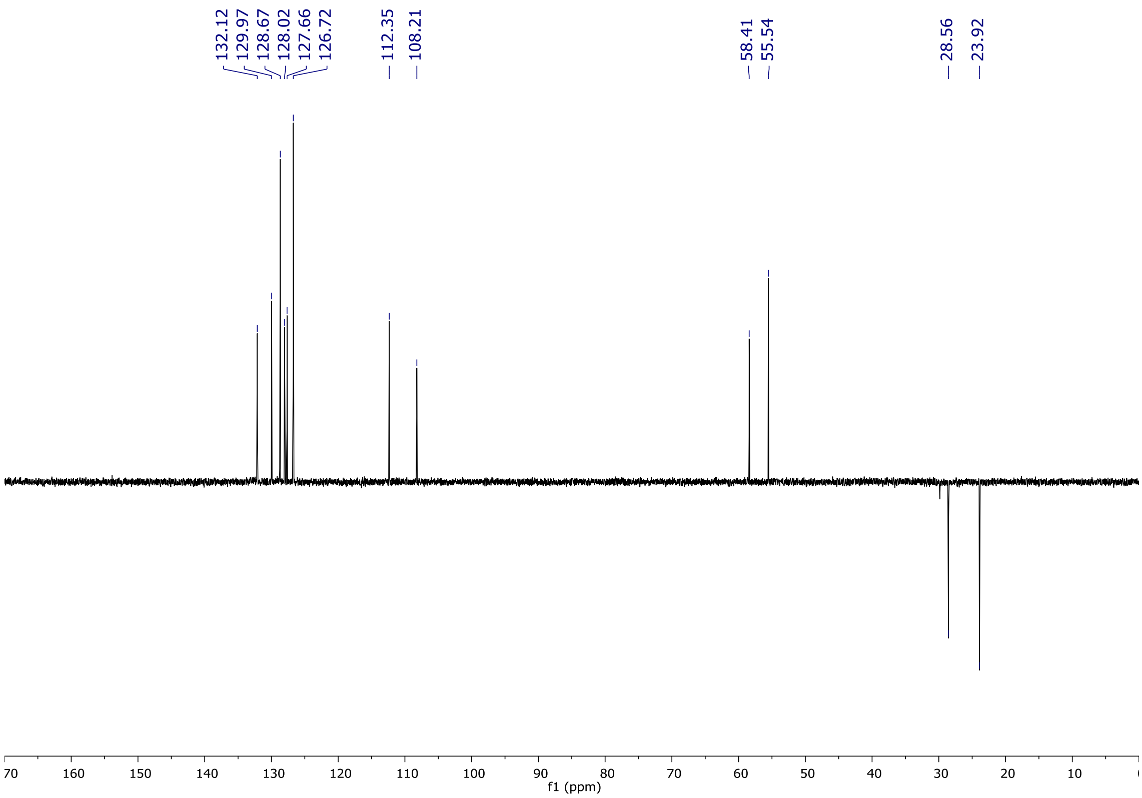

^{13}C NMR (126 MHz, $CDCl_3$)

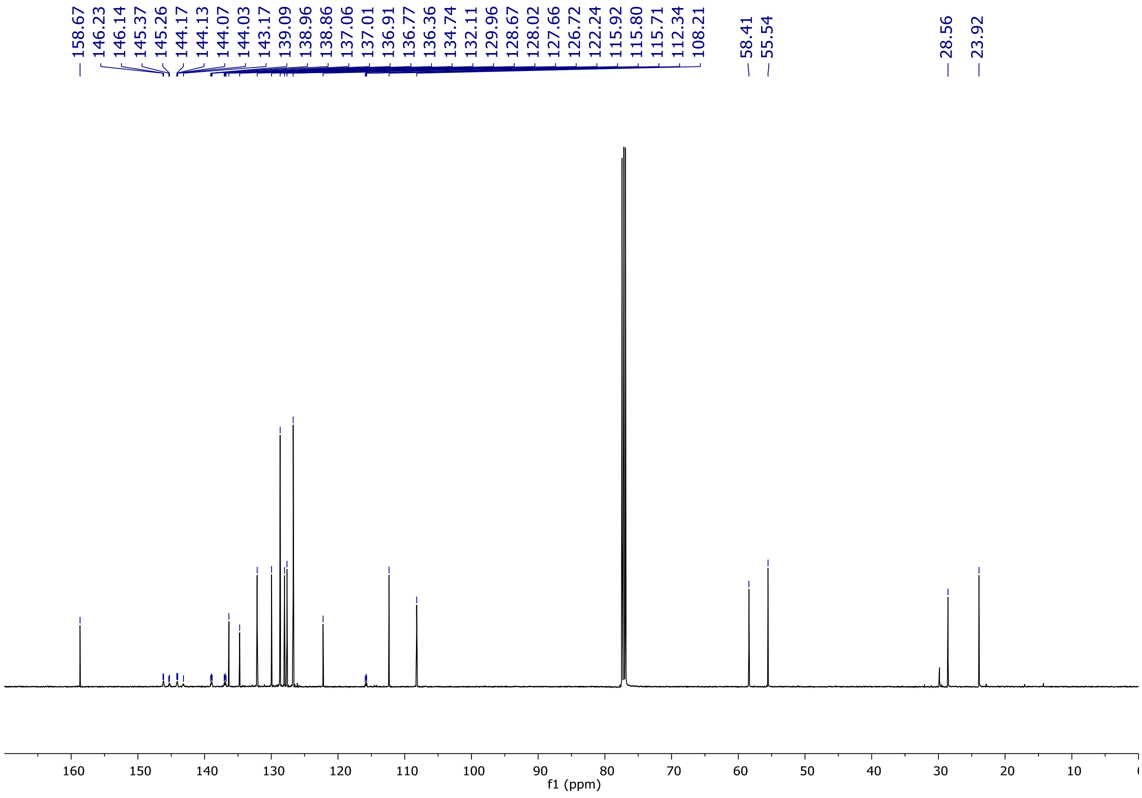

^{1}H NMR (300 MHz, $CDCl_3$)

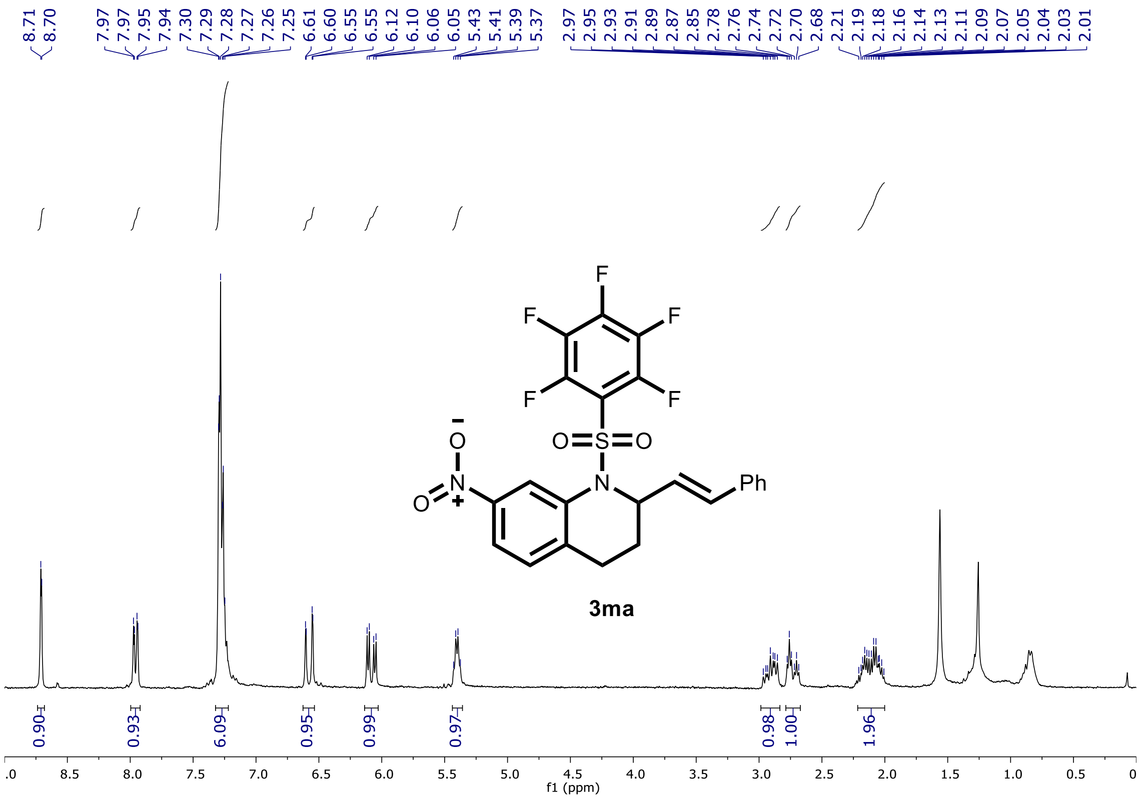

^{19}F NMR (471 MHz, $CDCl_3$)

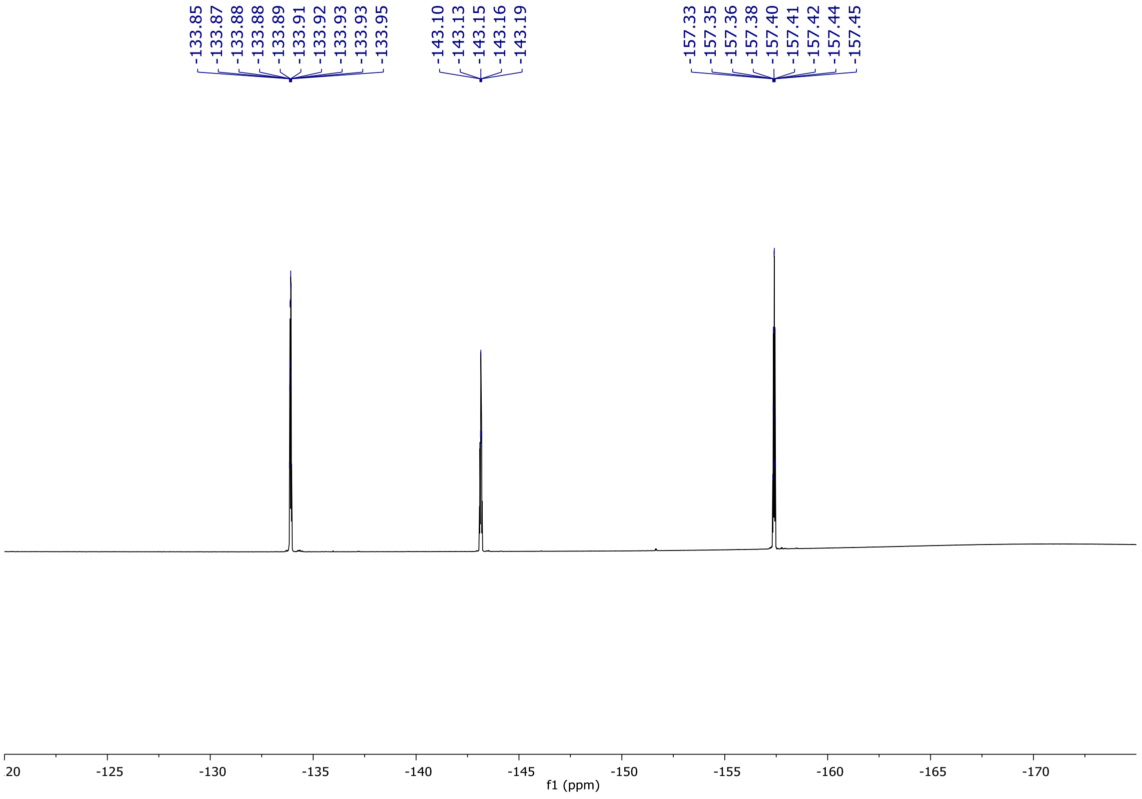

DEPT-135

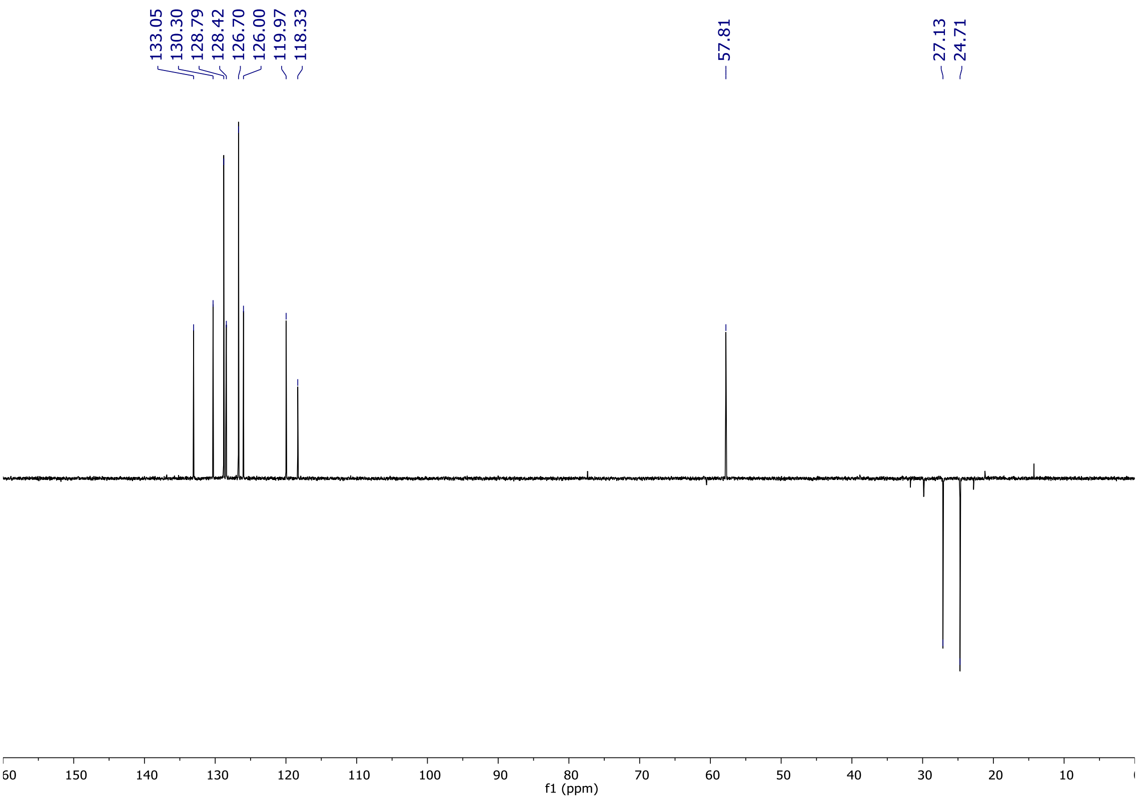

^{13}C NMR (126 MHz, $CDCl_3$)

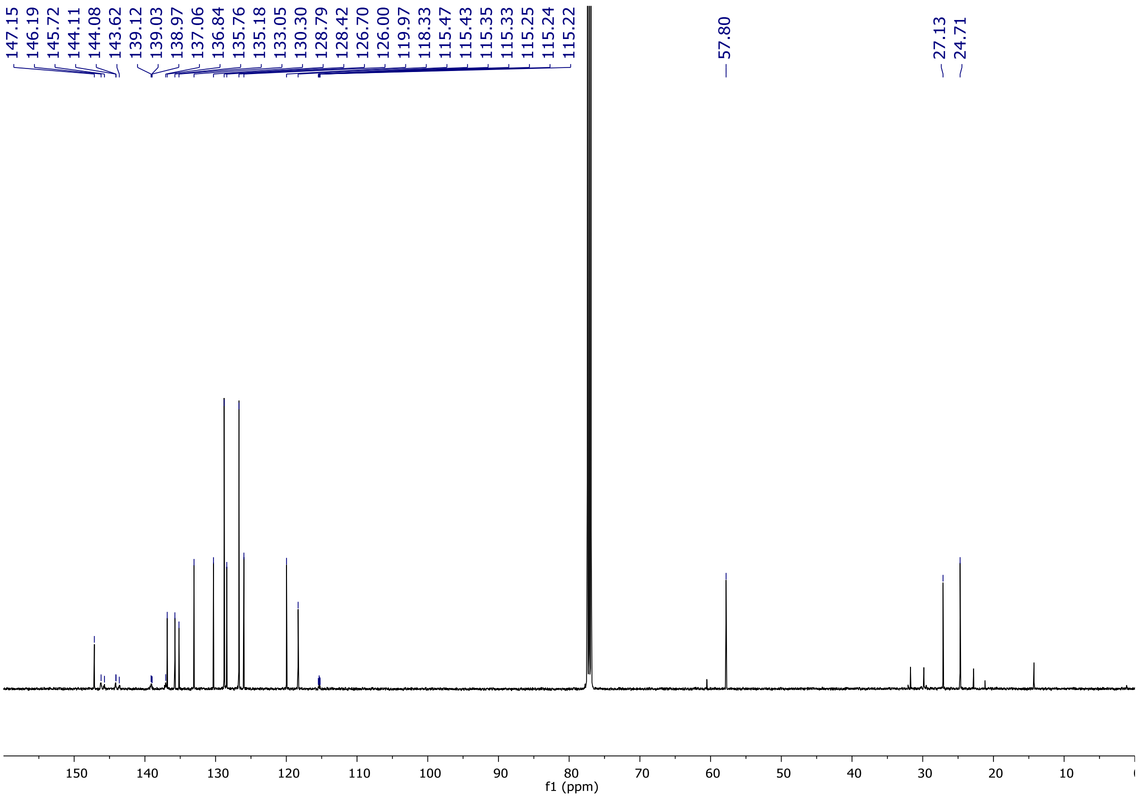

^{1}H NMR (300 MHz, $CDCl_3$)

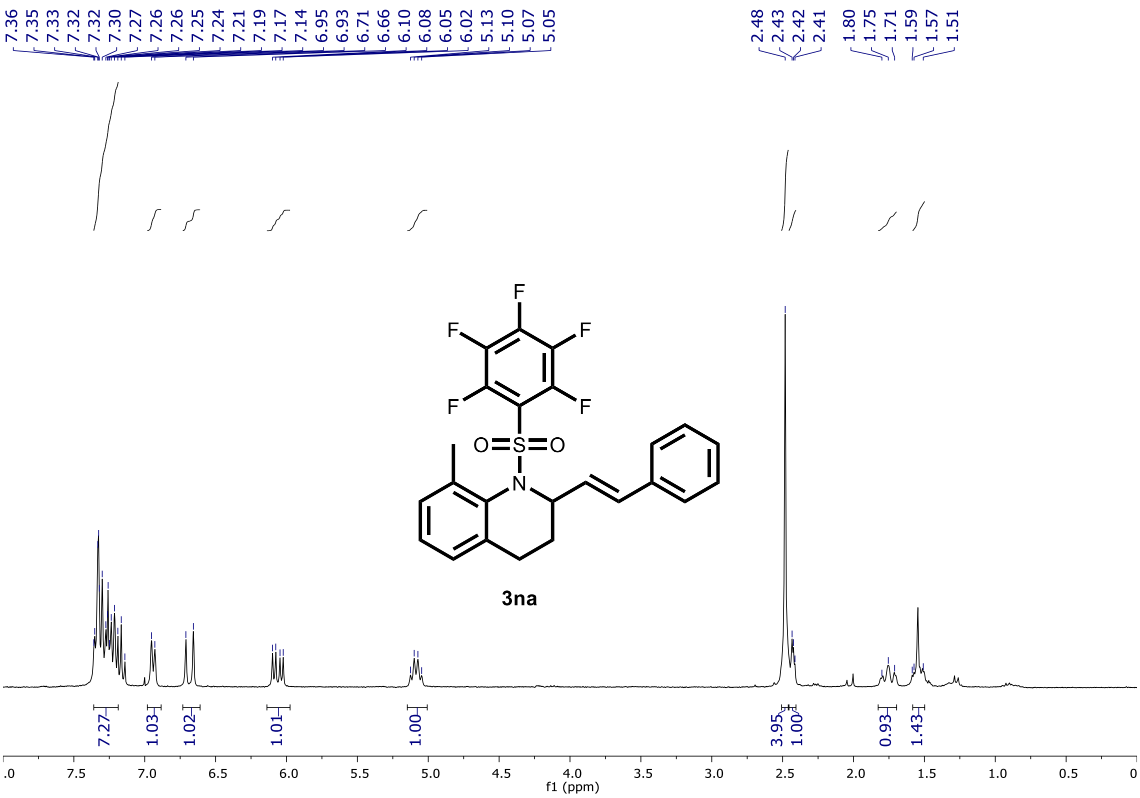

^{19}F NMR (471 MHz, $CDCl_3$)

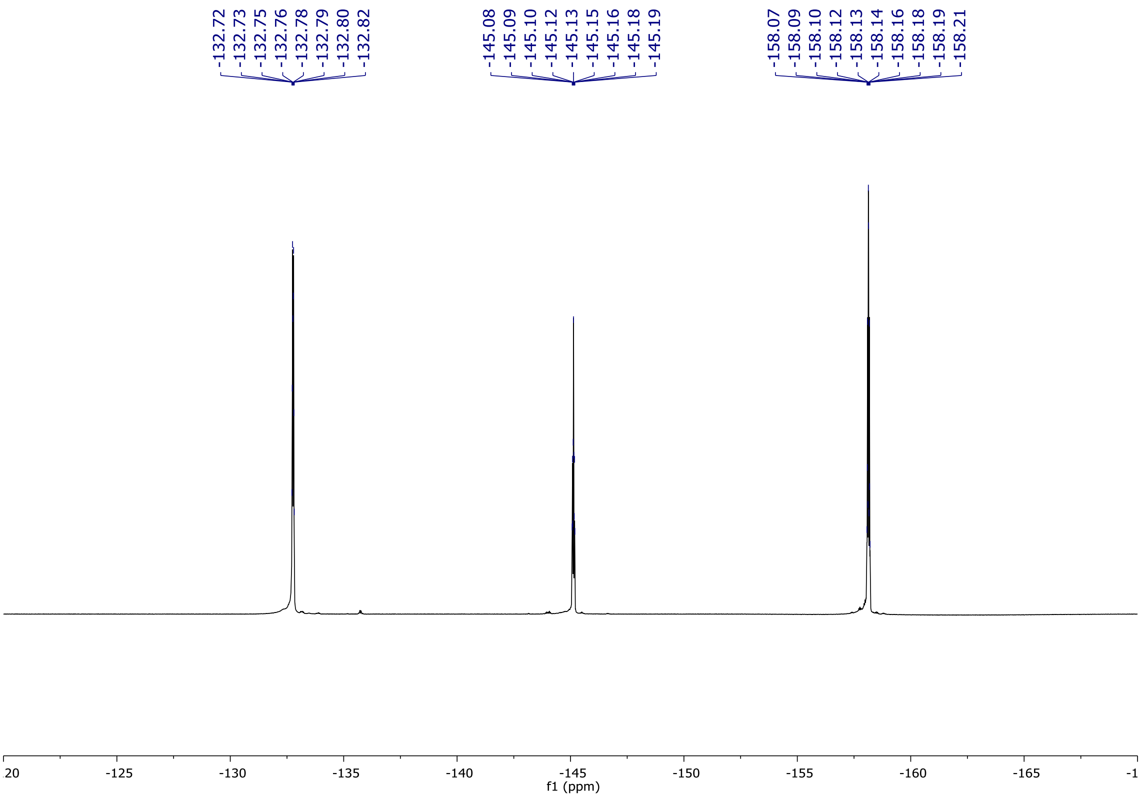

DEPT-135

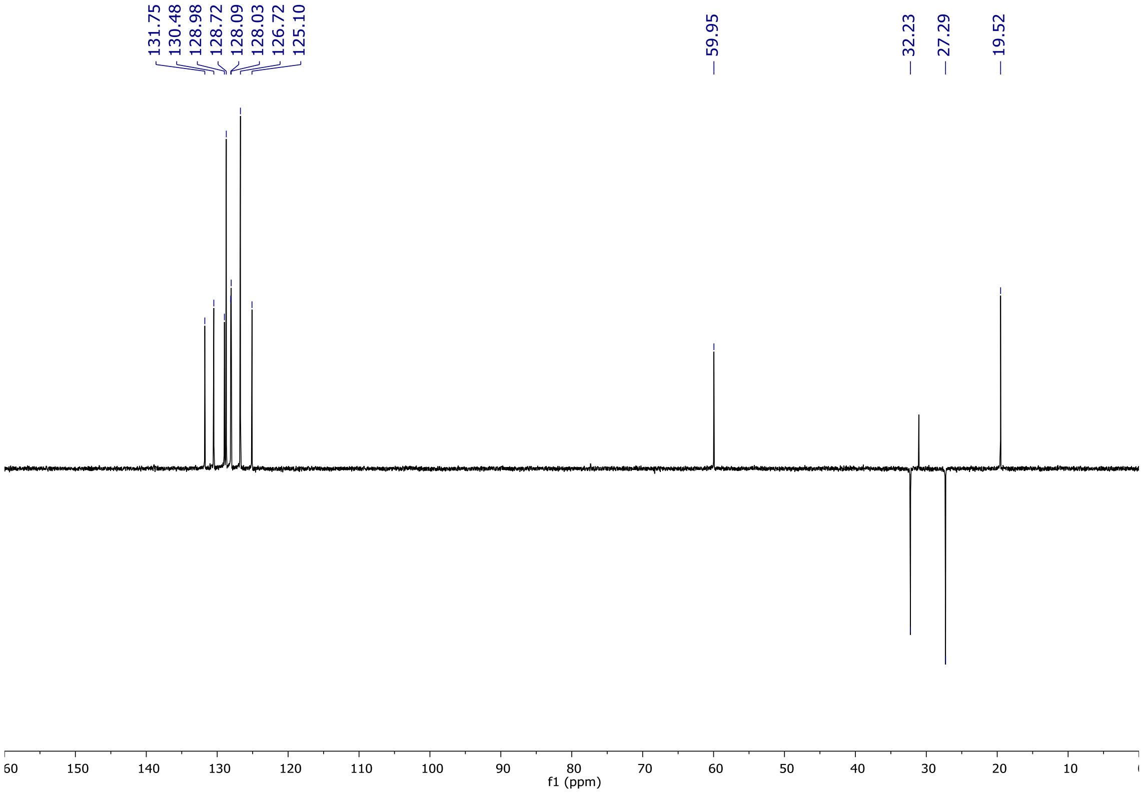

^{13}C NMR (126 MHz, CDCl$_3$)

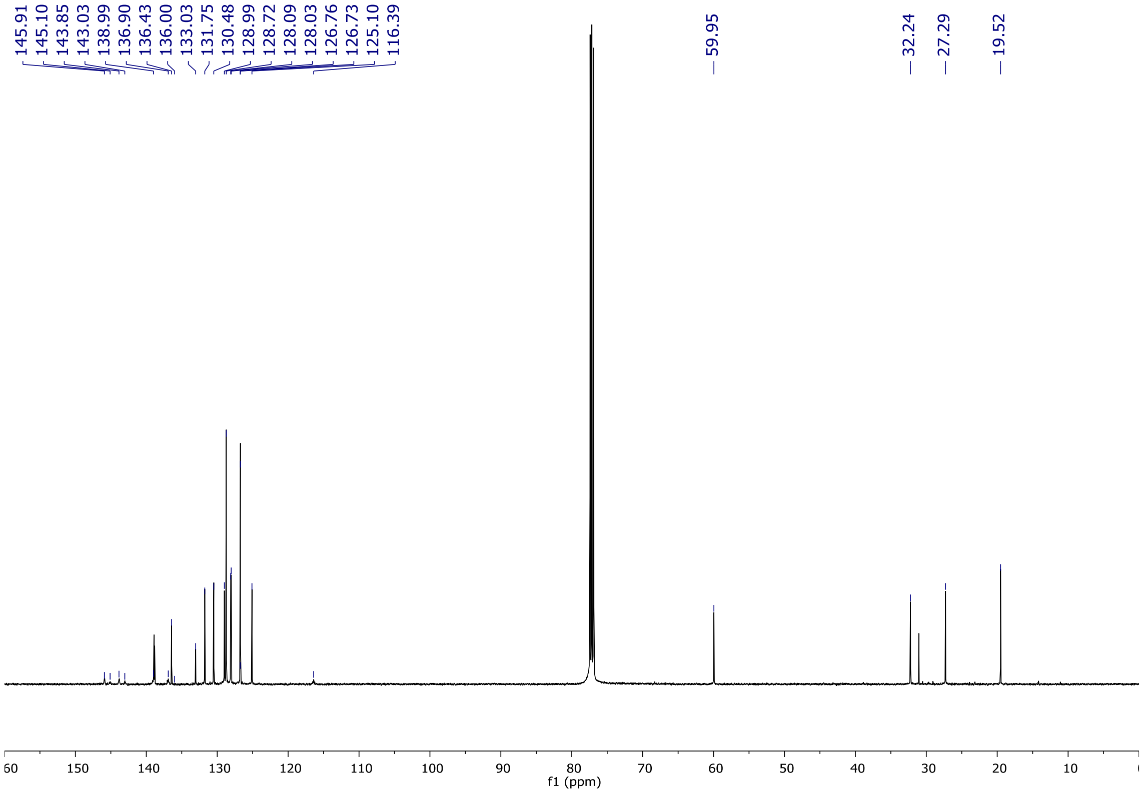

^{1}H NMR (500 MHz, $CDCl_3$)

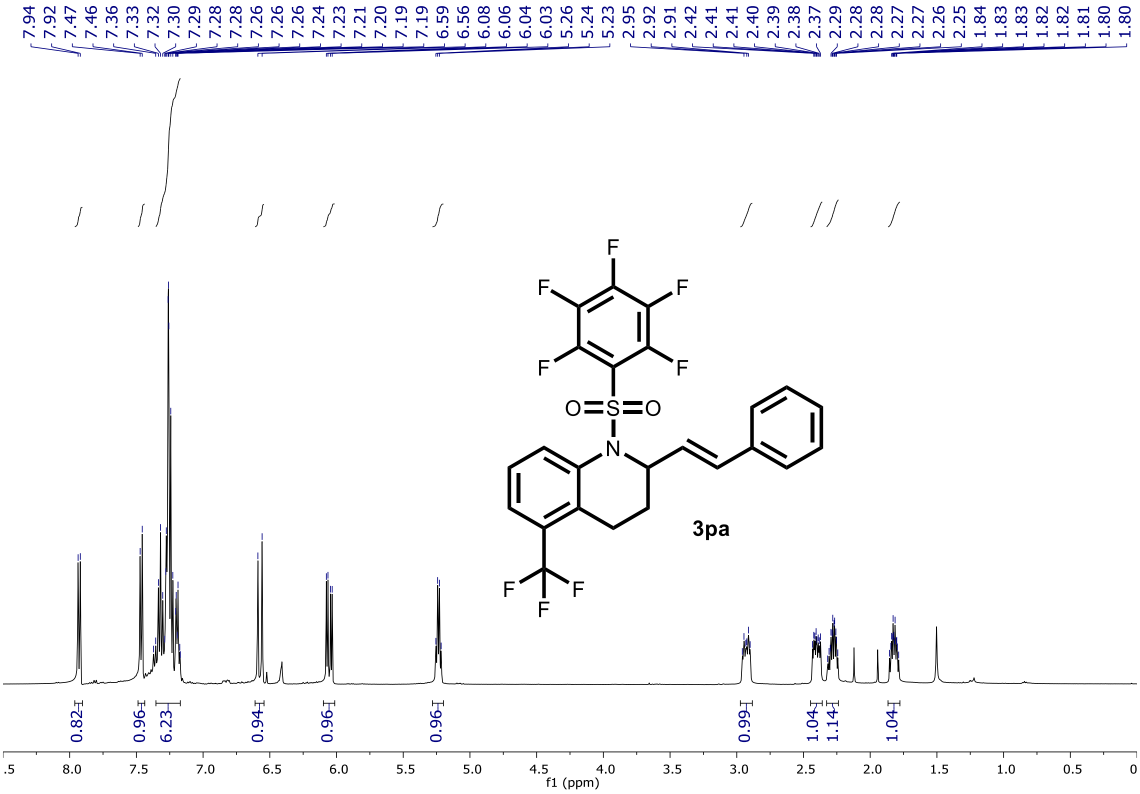

^{19}F NMR (471 MHz, $CDCl_3$)

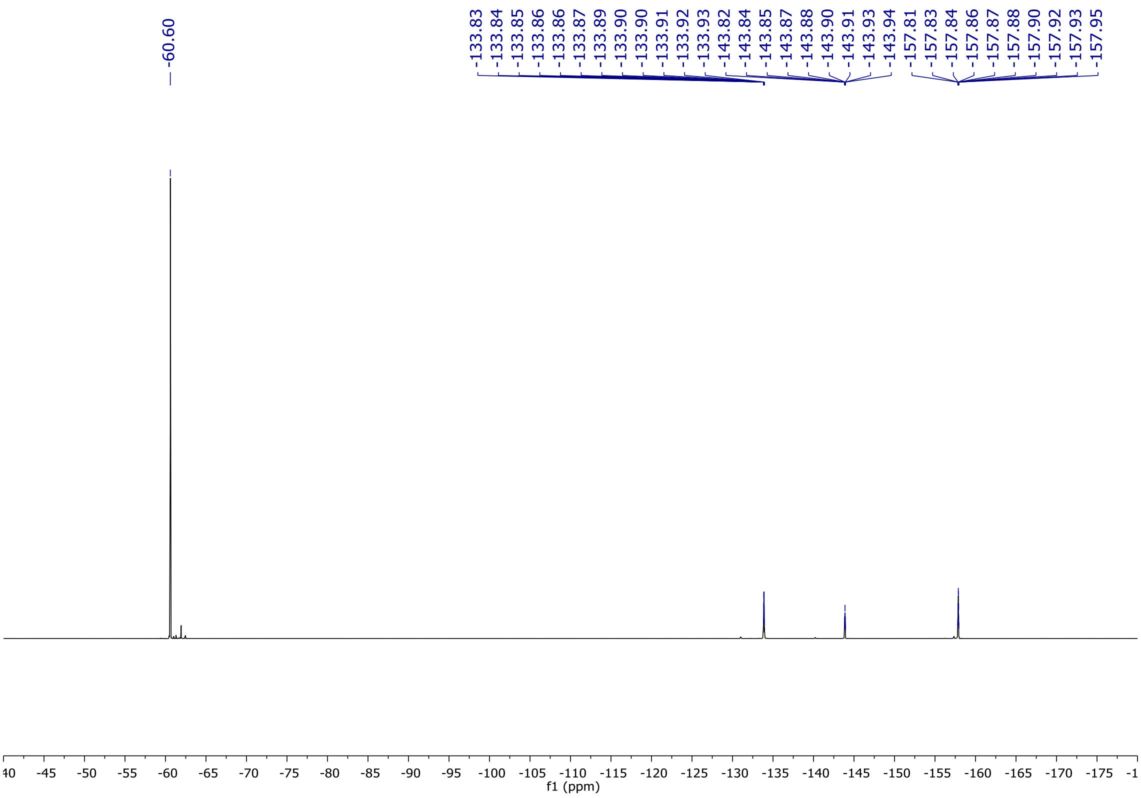

DEPT-135

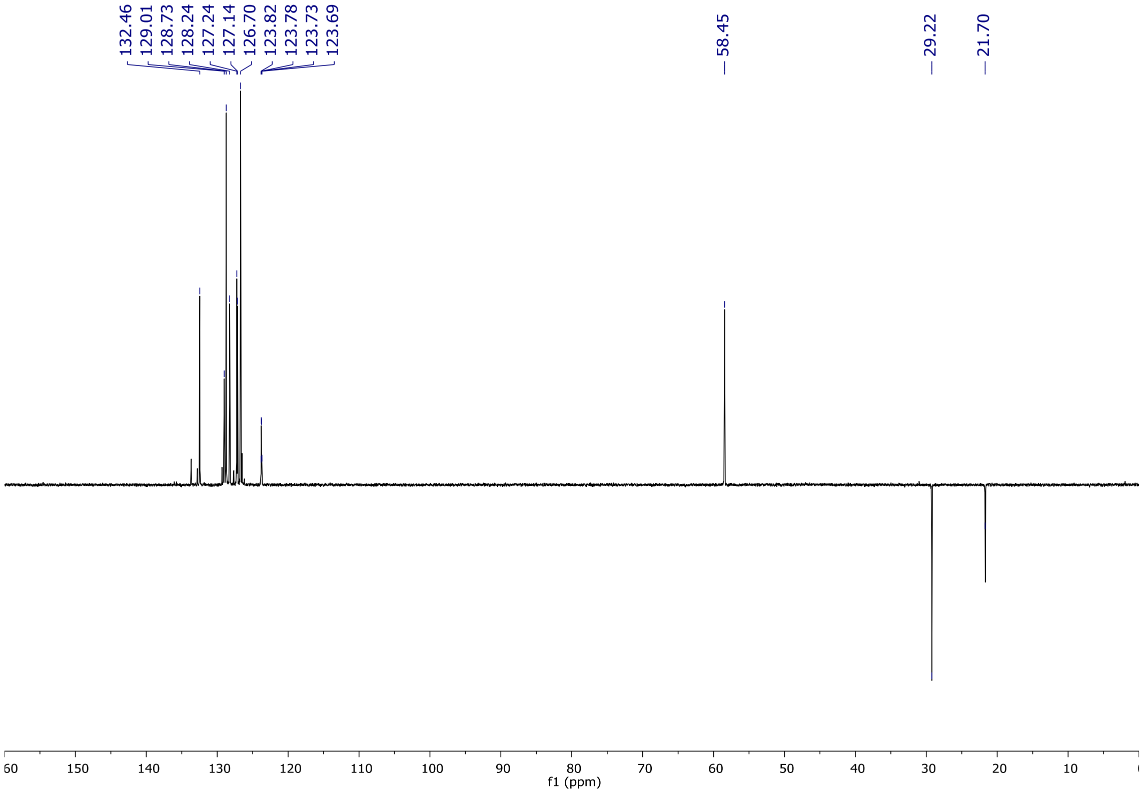

^{13}C NMR (126 MHz, $CDCl_3$)

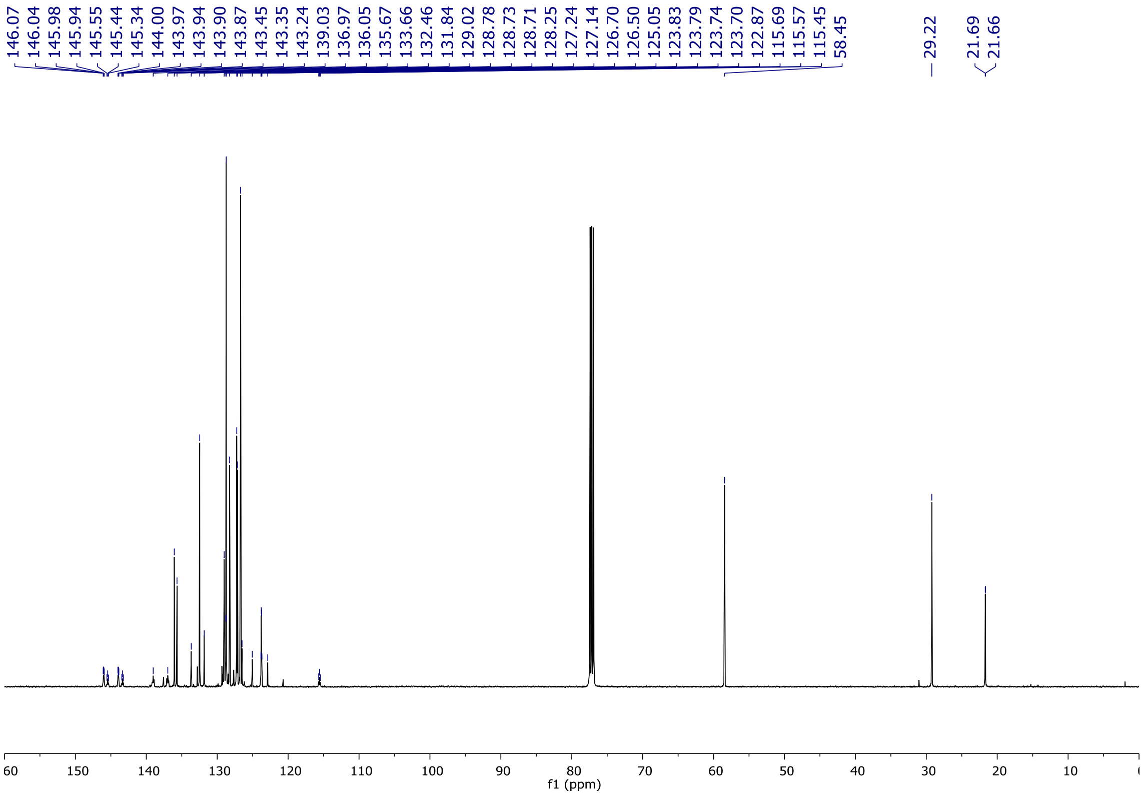

^{1}H NMR (500 MHz, $CDCl_3$)

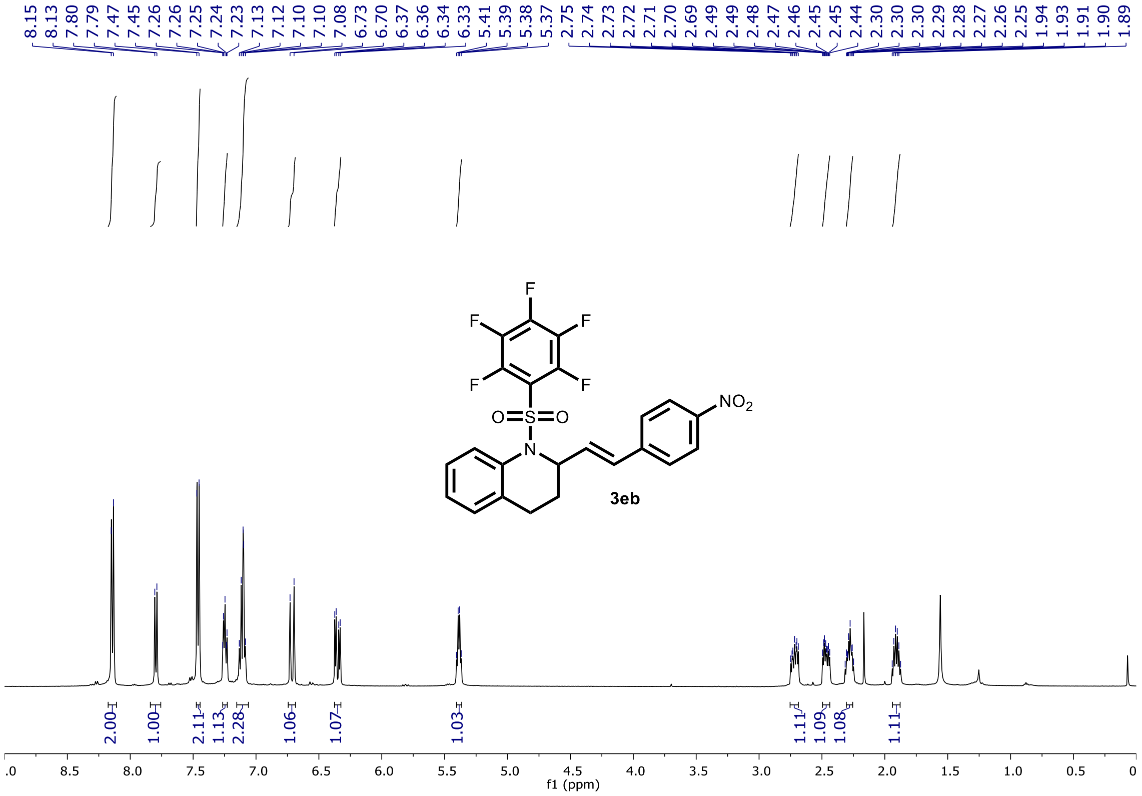

^{19}F NMR (471 MHz, $CDCl_3$)

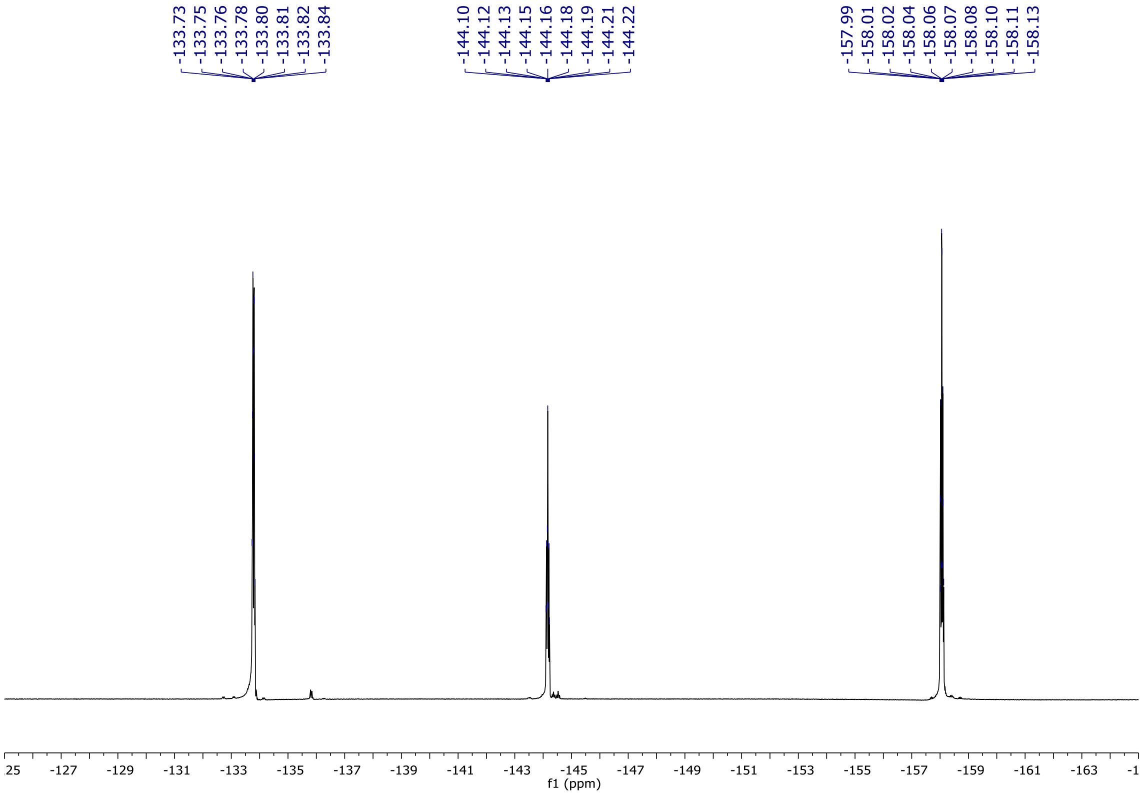

DEPT-135

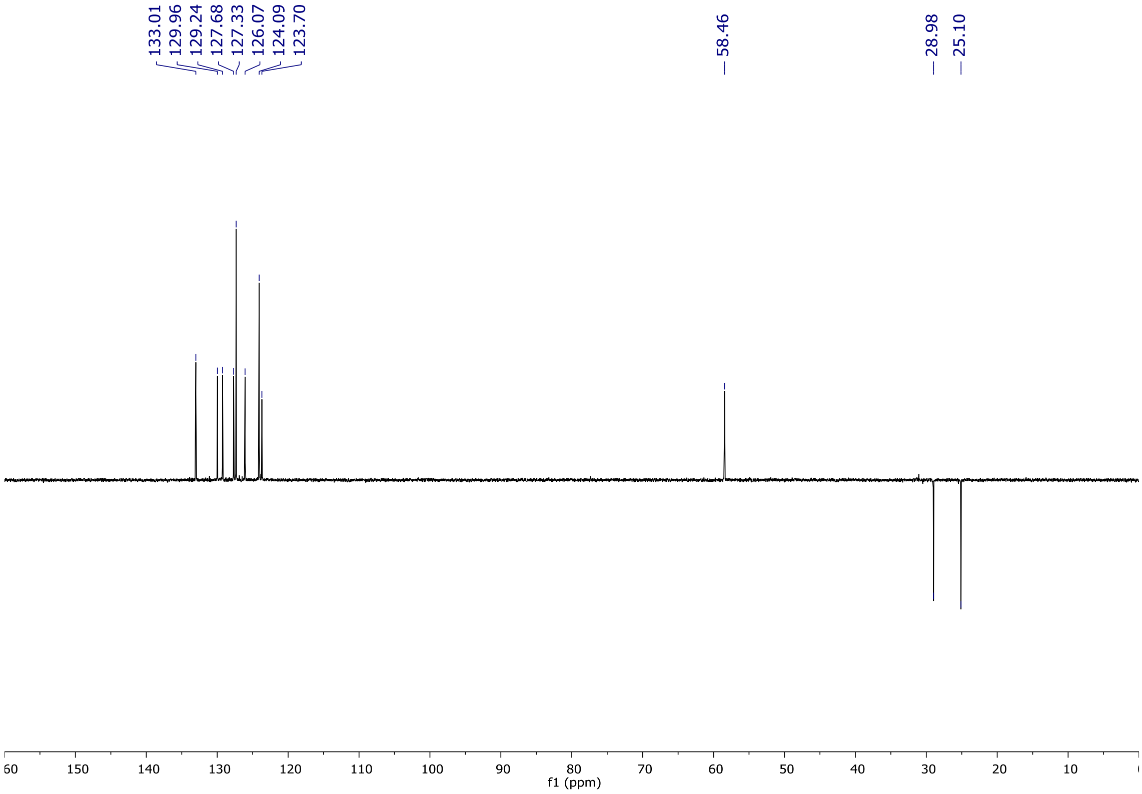

^{13}C NMR (126 MHz, $CDCl_3$)

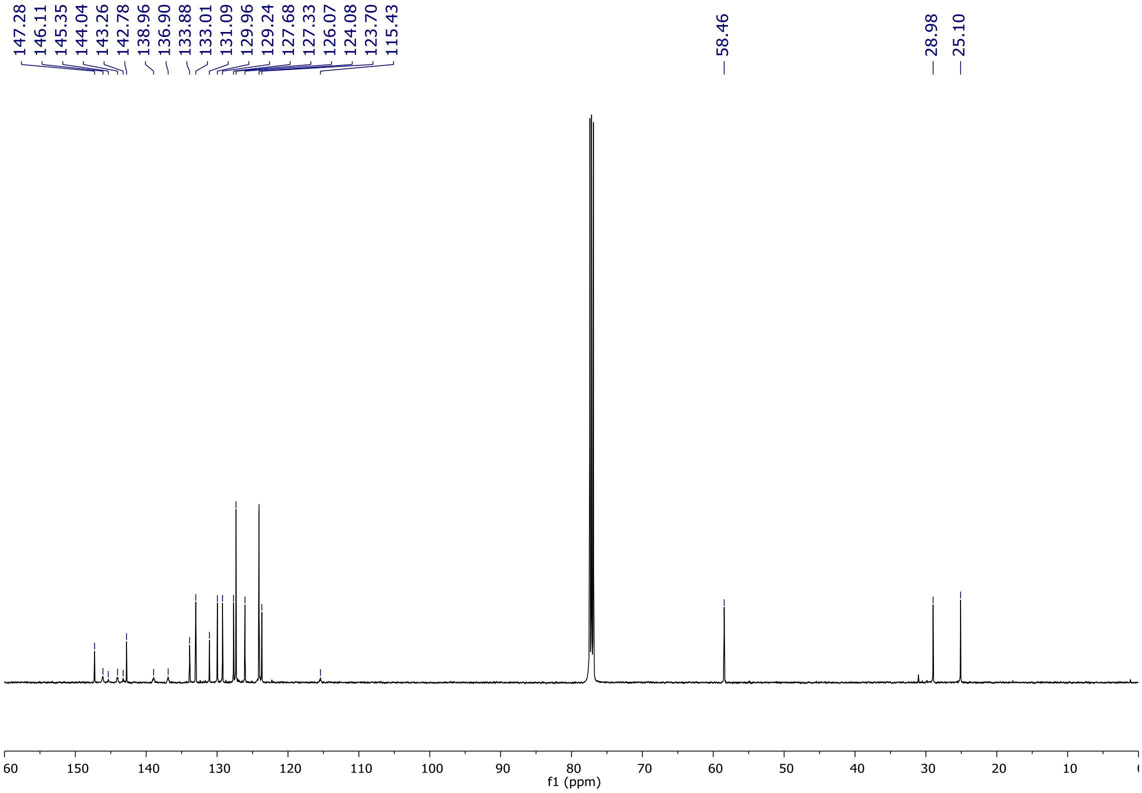

^{1}H NMR (500 MHz, $CDCl_3$)

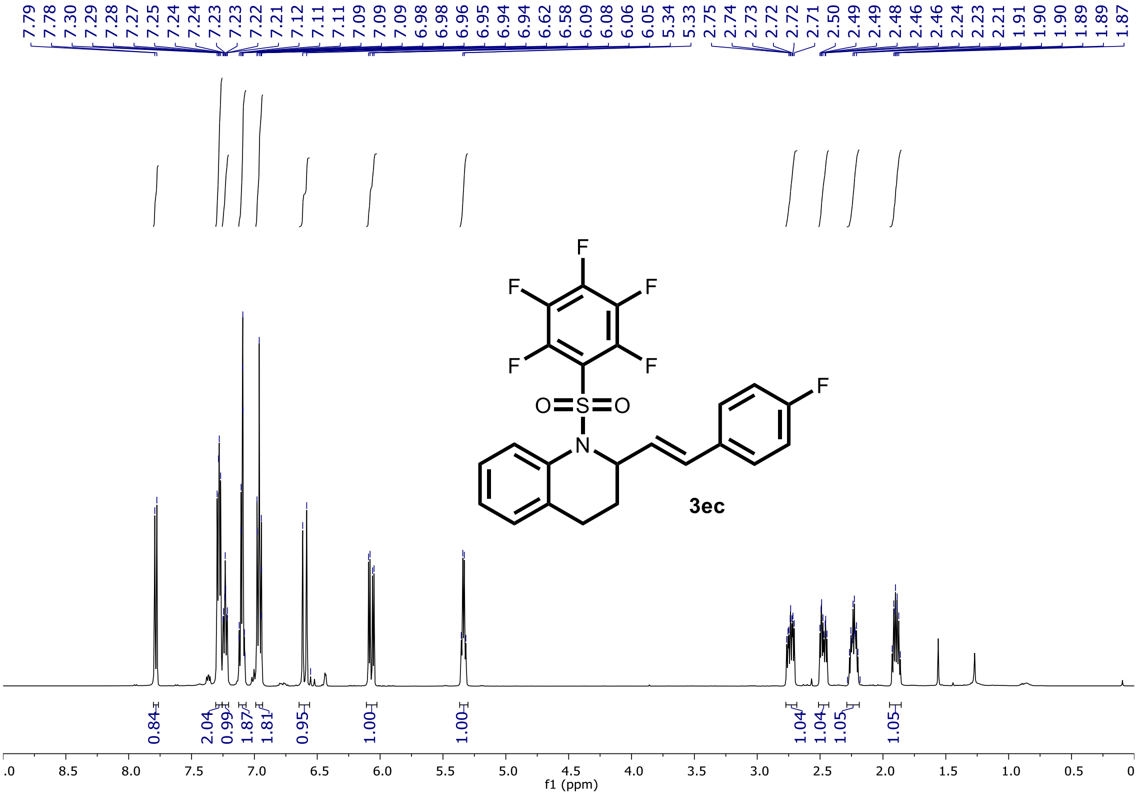

^{19}F NMR (471 MHz, $CDCl_3$)

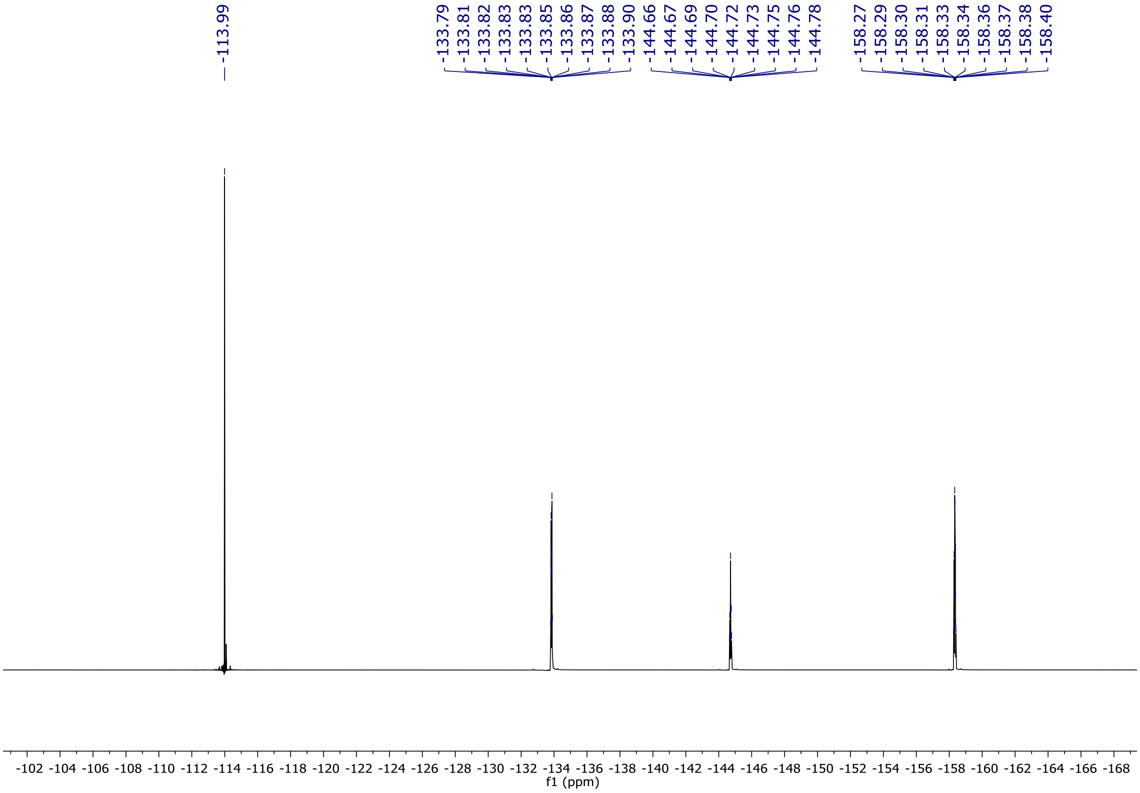

DEPT-135

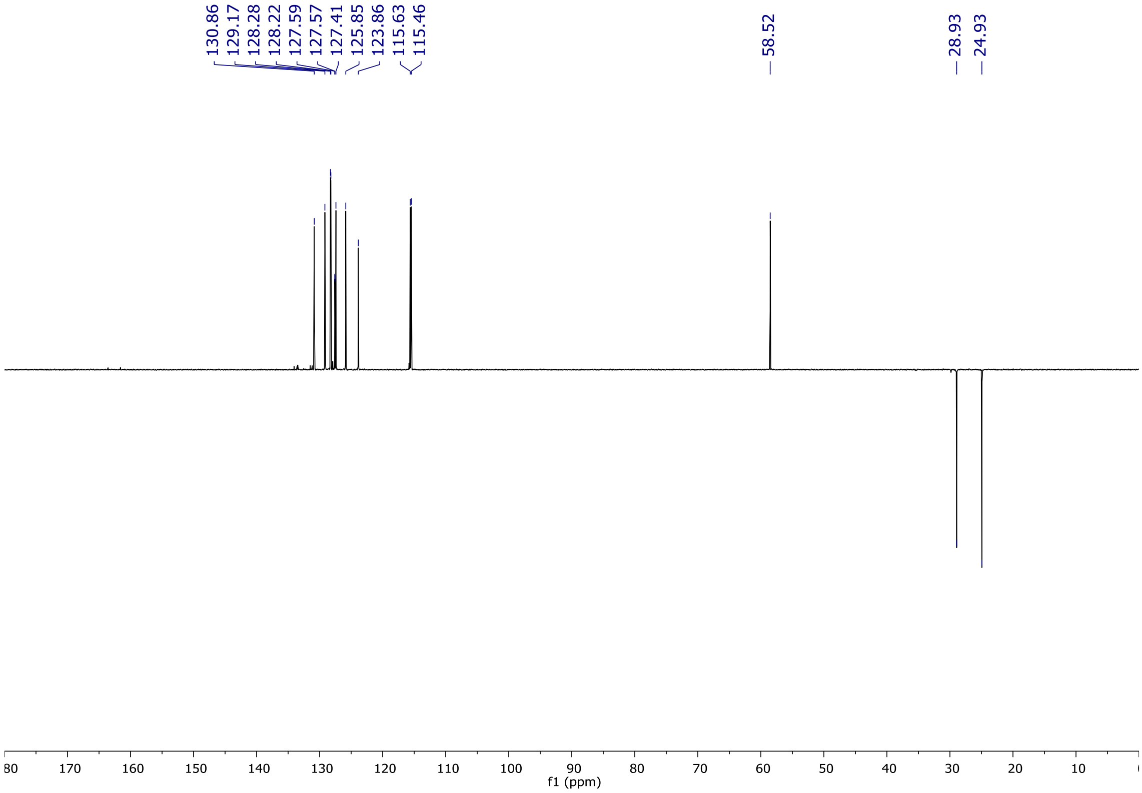

^{13}C NMR (126 MHz, $CDCl_3$)

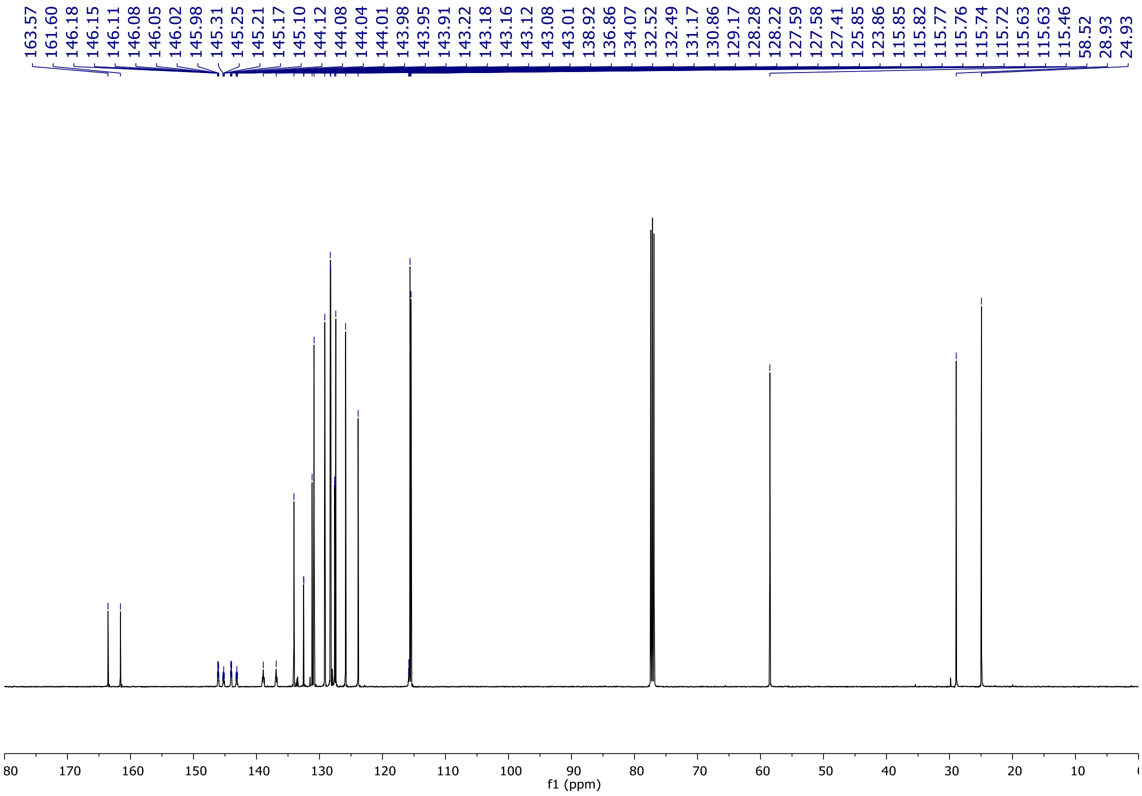

^{1}H NMR (500 MHz, $CDCl_3$)

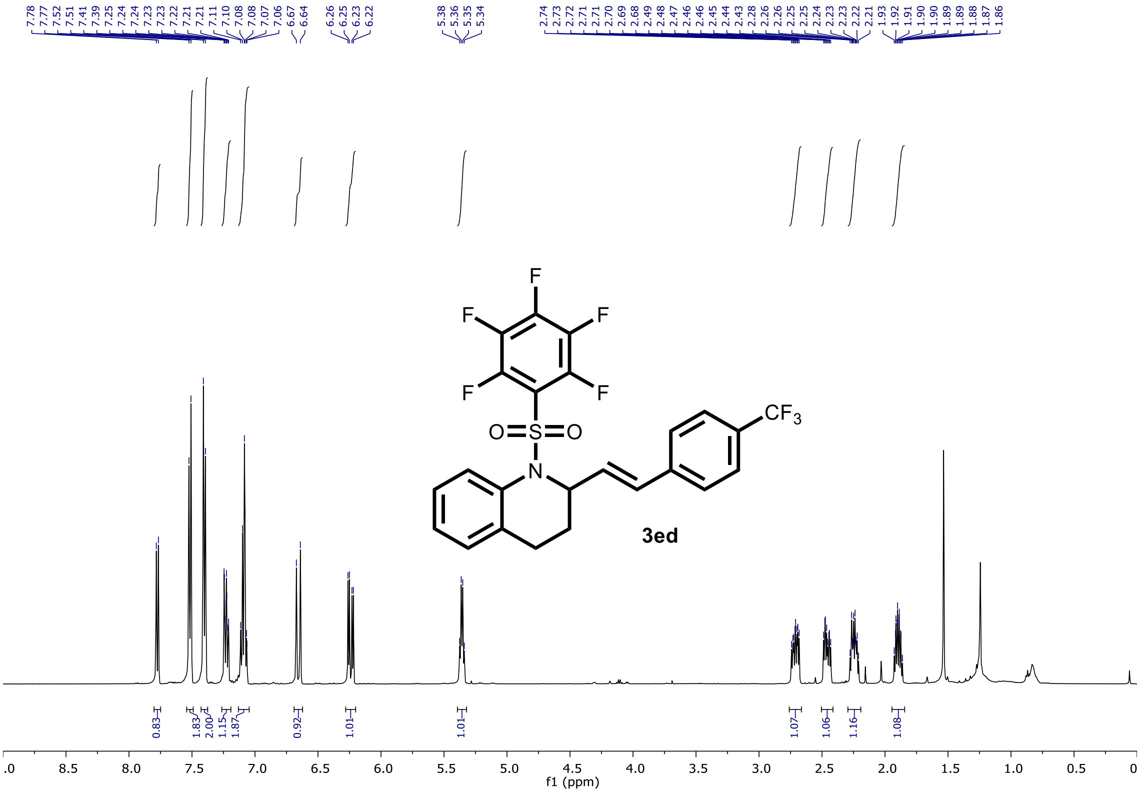

^{19}F NMR (471 MHz, $CDCl_3$)

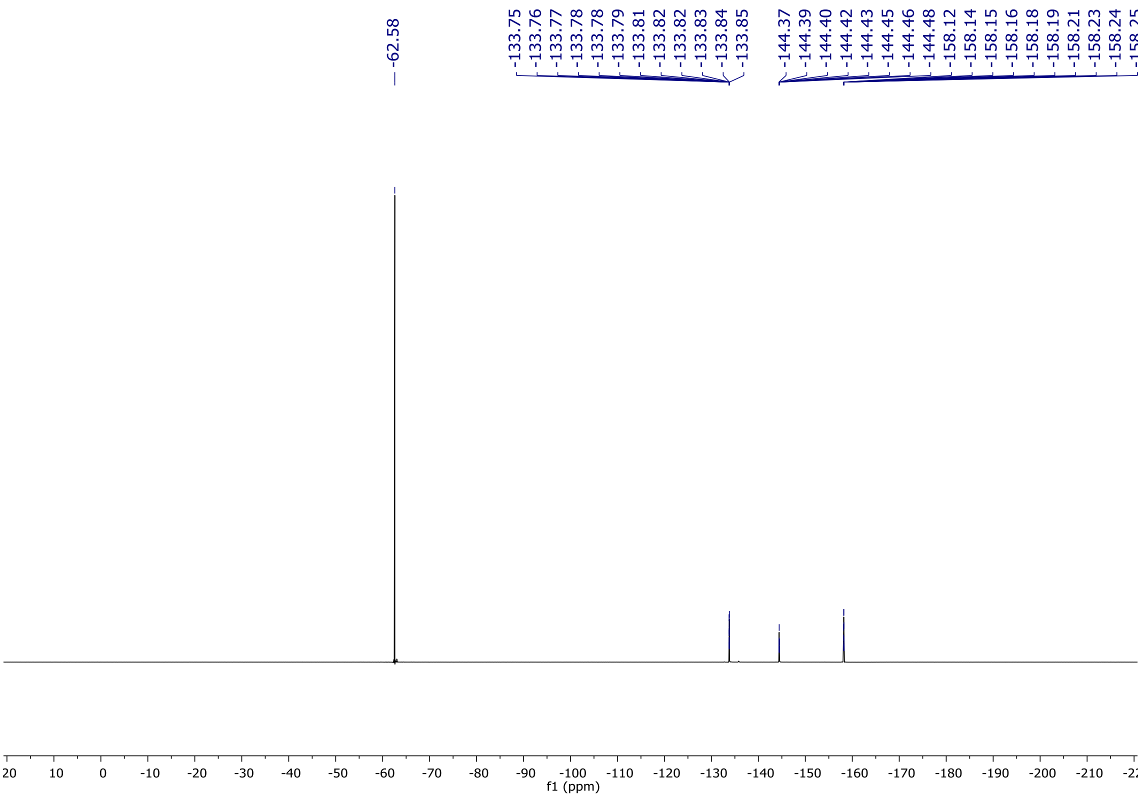

DEPT-135

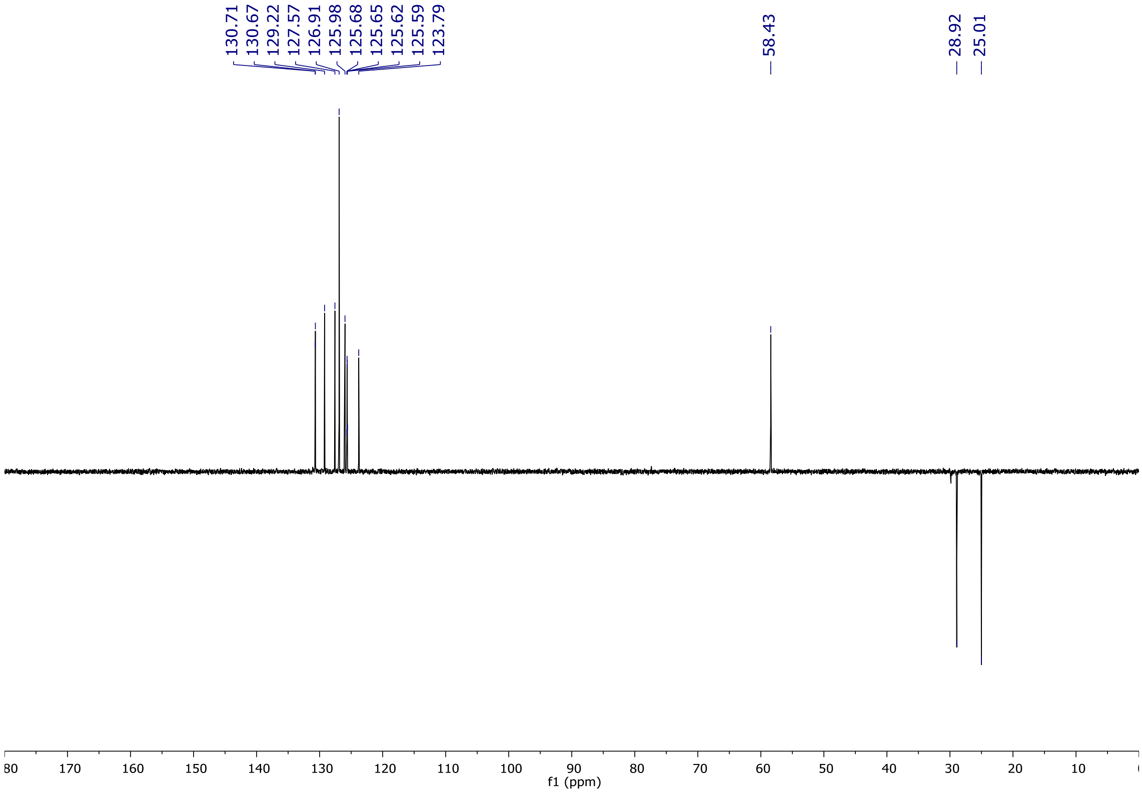

^{13}C NMR (126 MHz, $CDCl_3$)

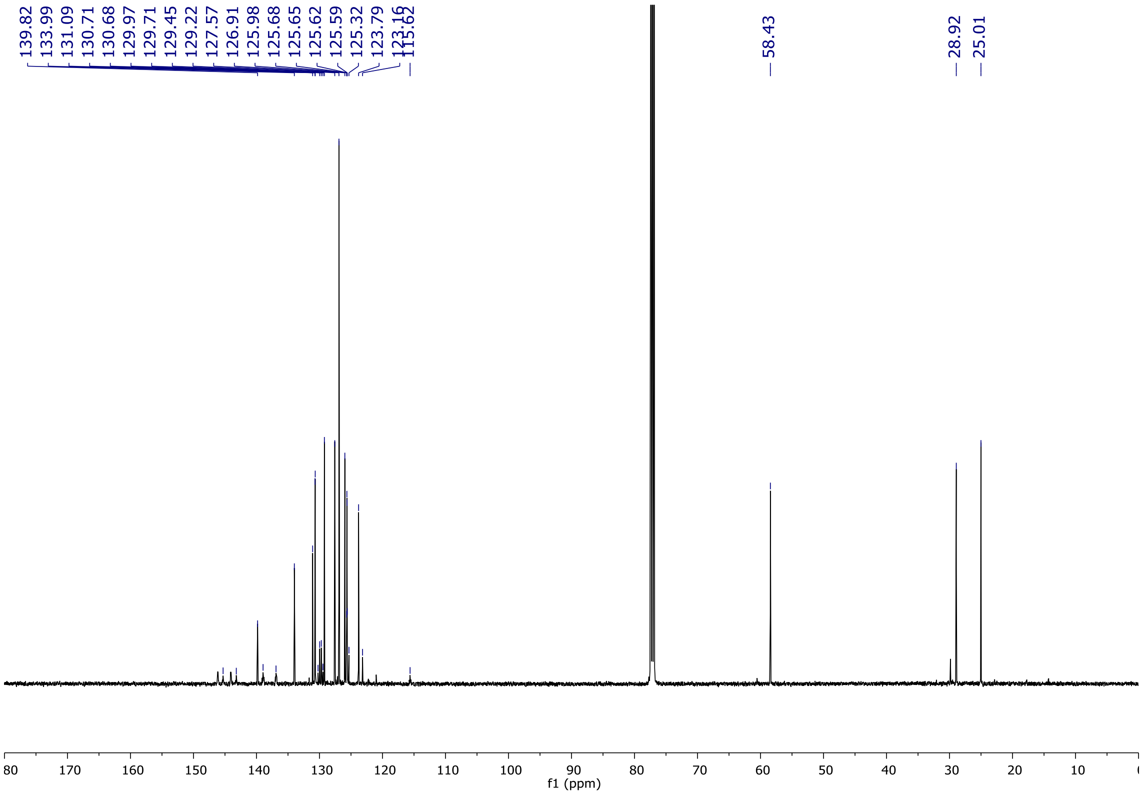

^{1}H NMR (500 MHz, $CDCl_3$)

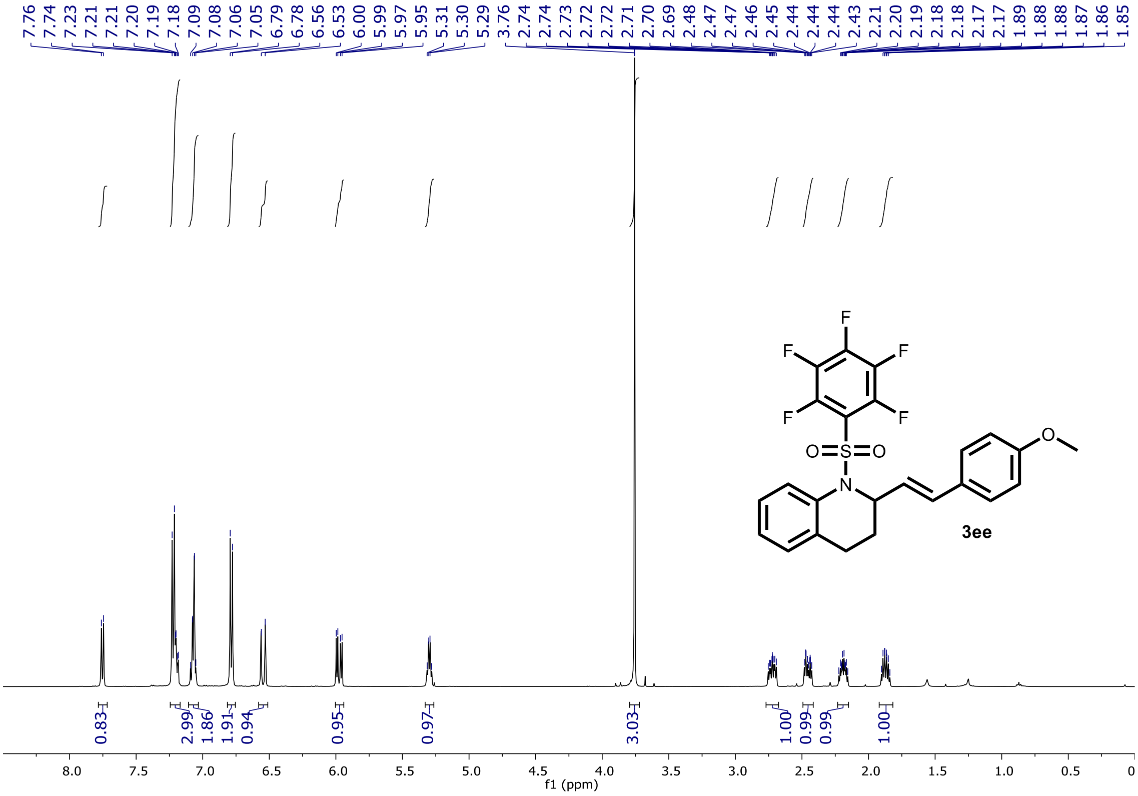

^{19}F NMR (471 MHz, $CDCl_3$)

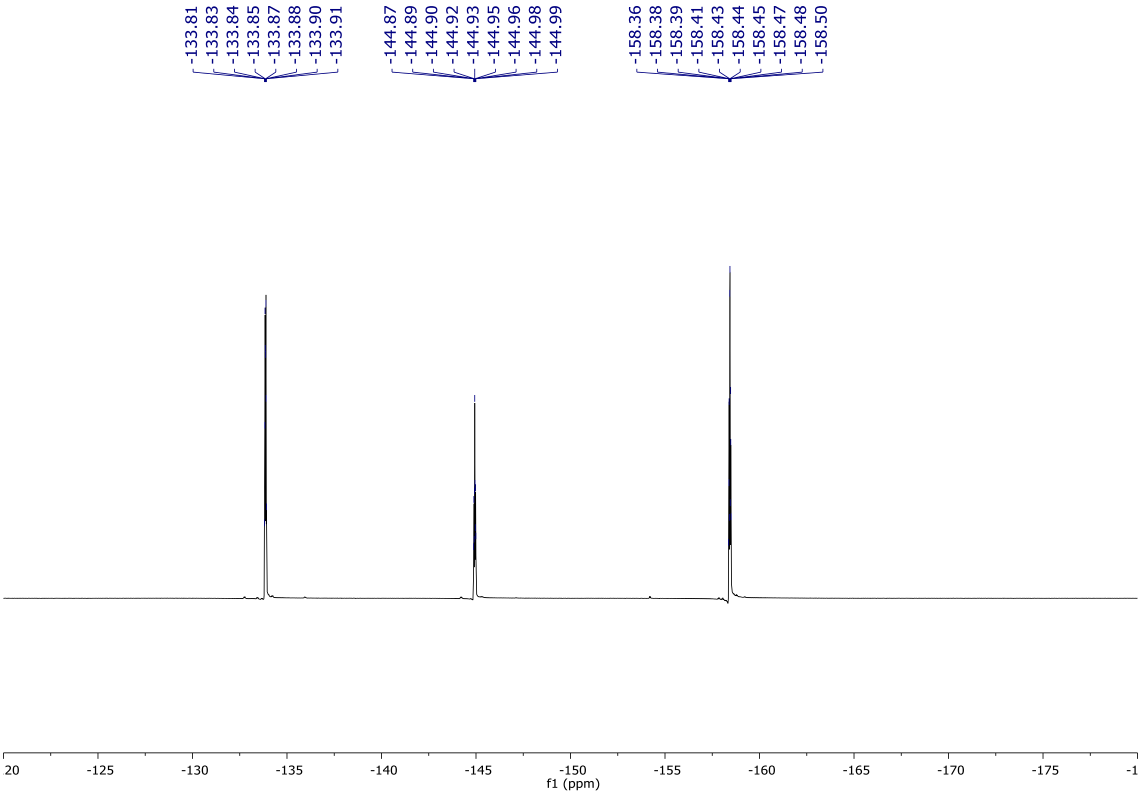

DEPT-135

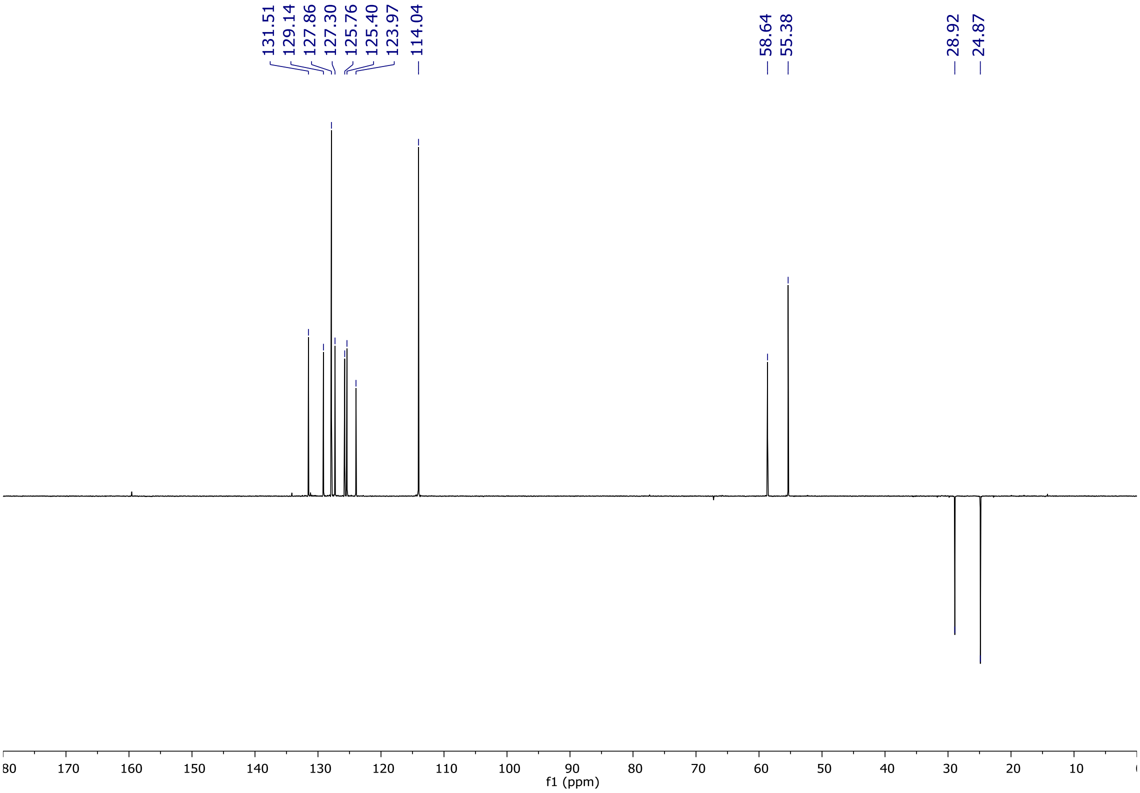

^{13}C NMR (126 MHz, $CDCl_3$)

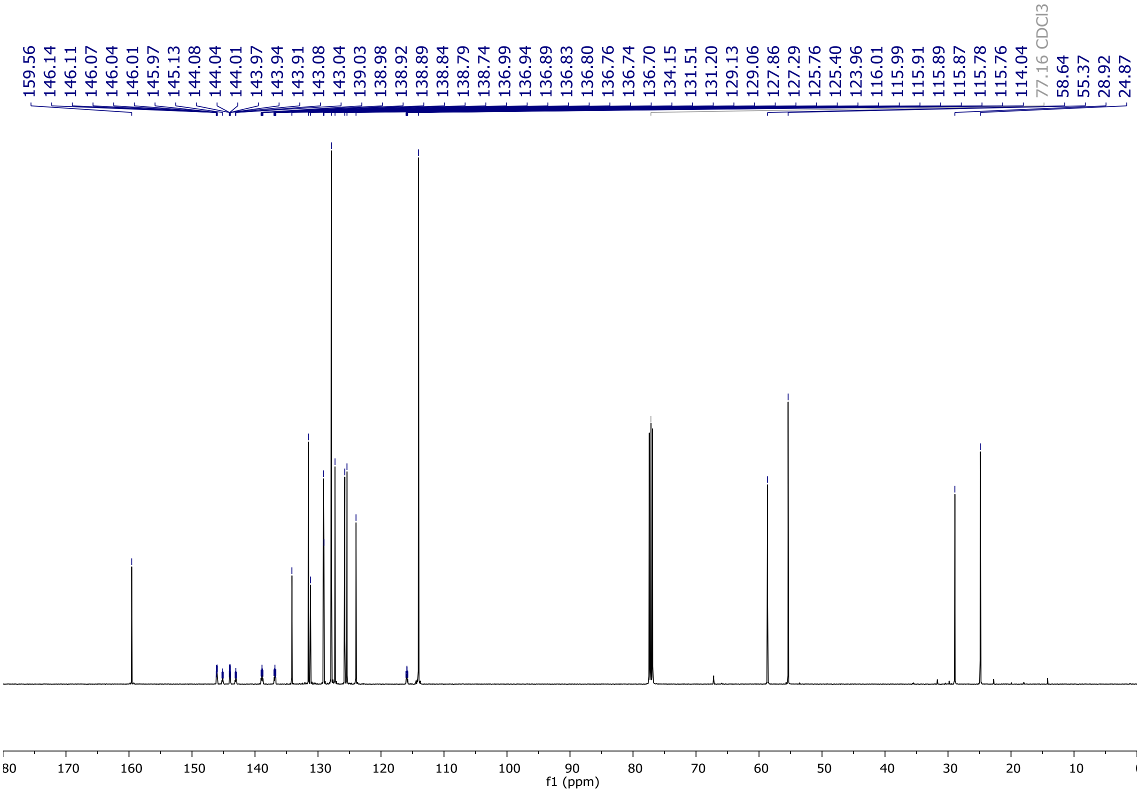

^{1}H NMR (500 MHz, $CDCl_3$)

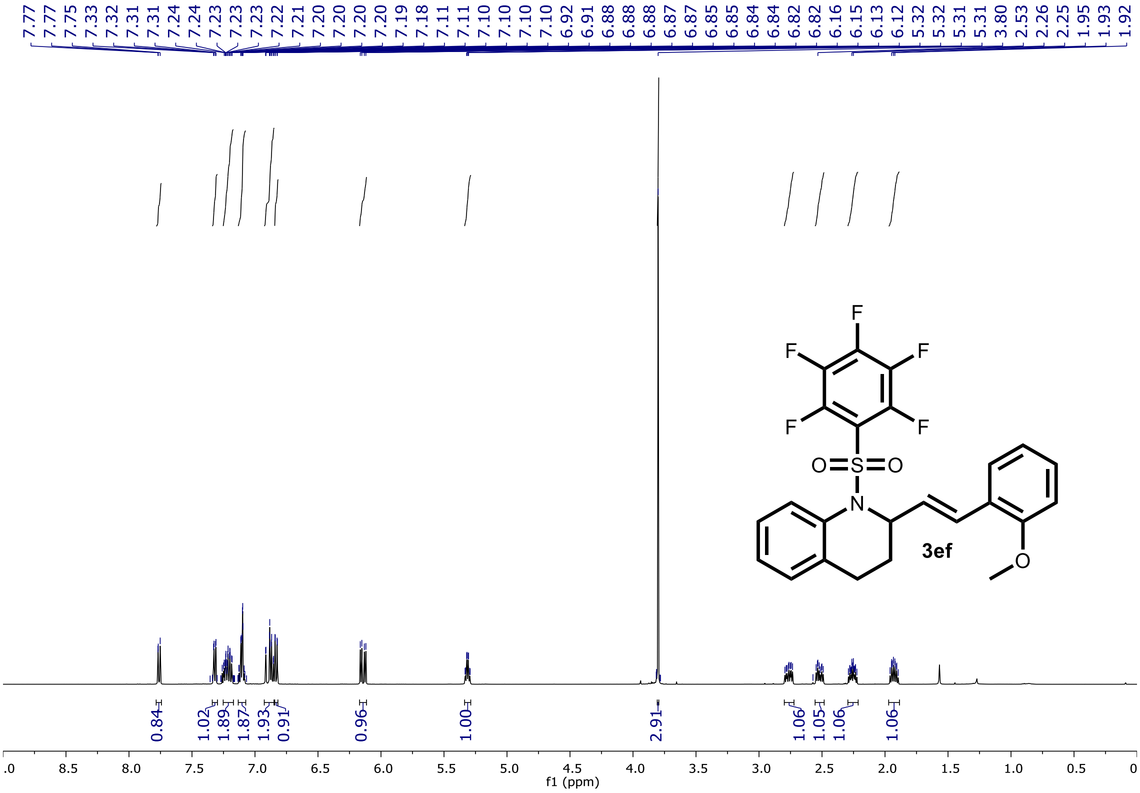

^{19}F NMR (471 MHz, $CDCl_3$)

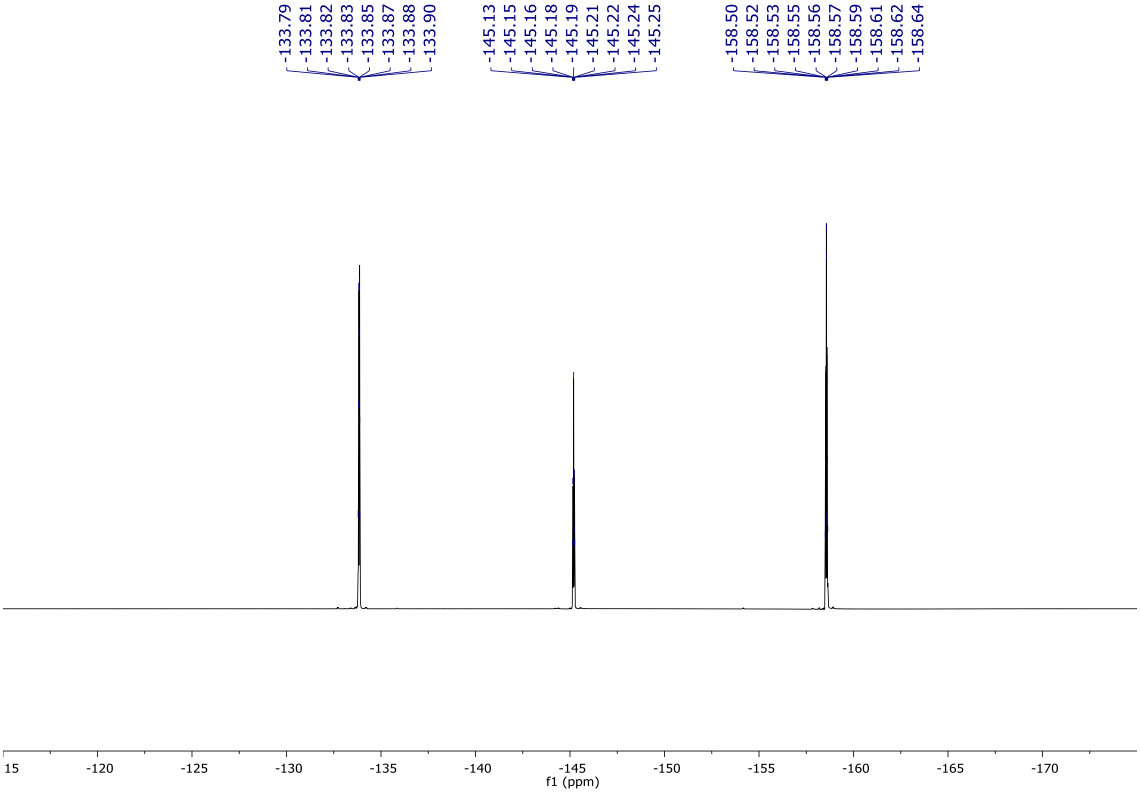

DEPT-135

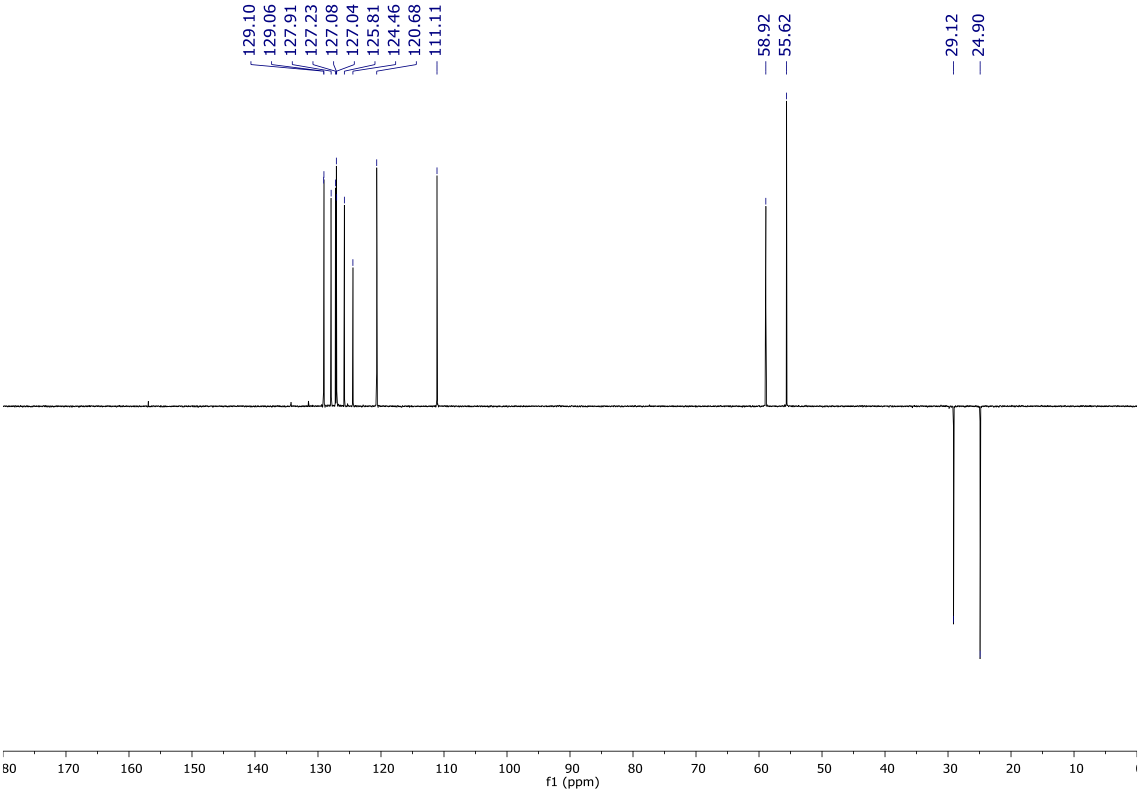

^{13}C NMR (126 MHz, $CDCl_3$)

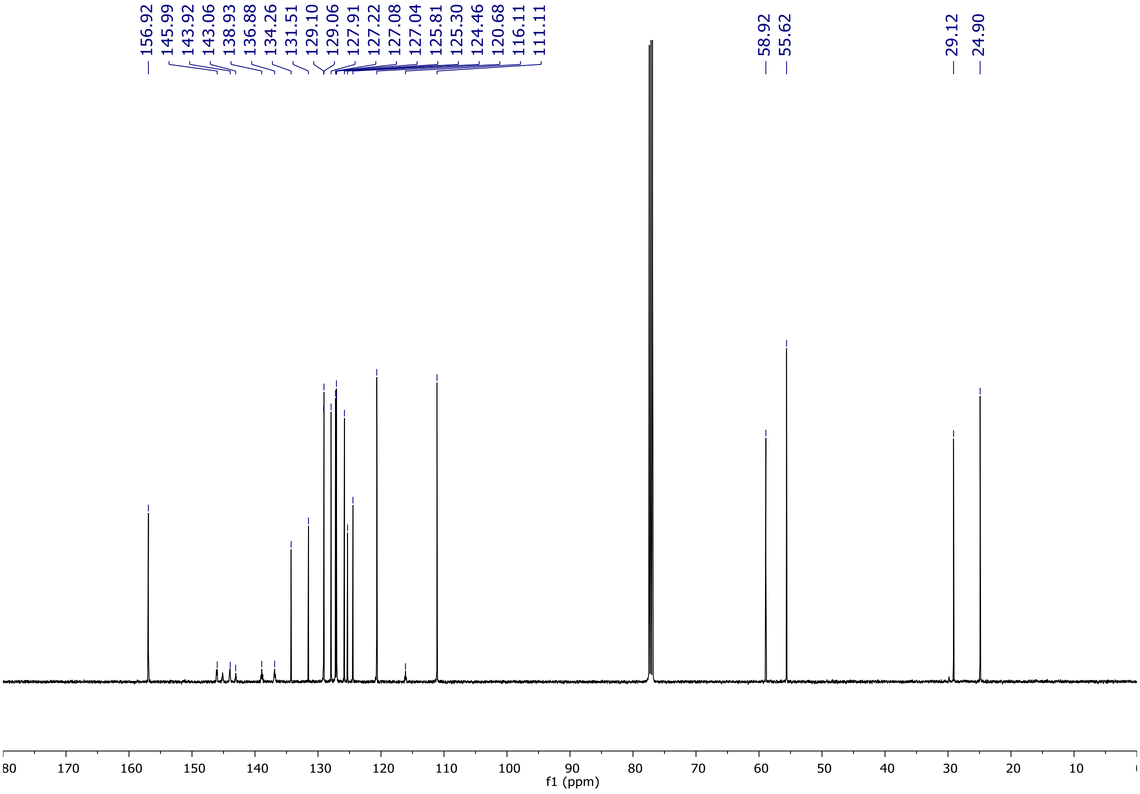

^{1}H NMR (500 MHz, $CDCl_3$)

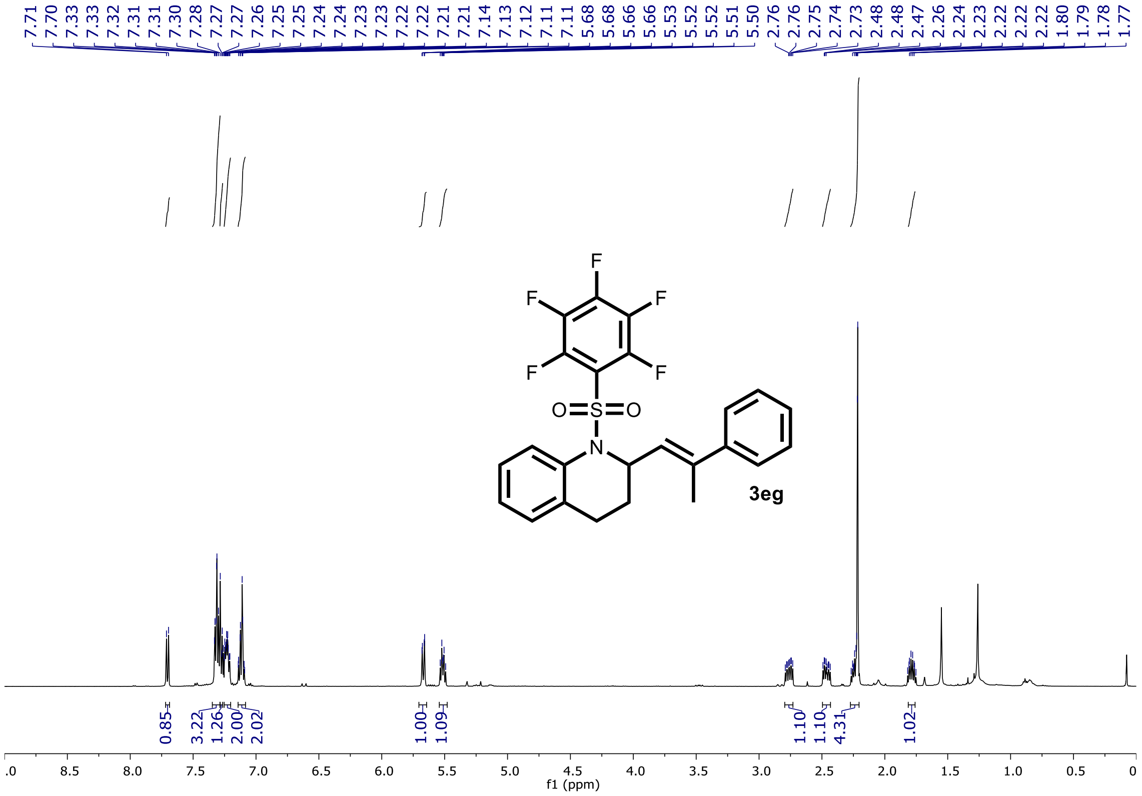

^{19}F NMR (471 MHz, $CDCl_3$)

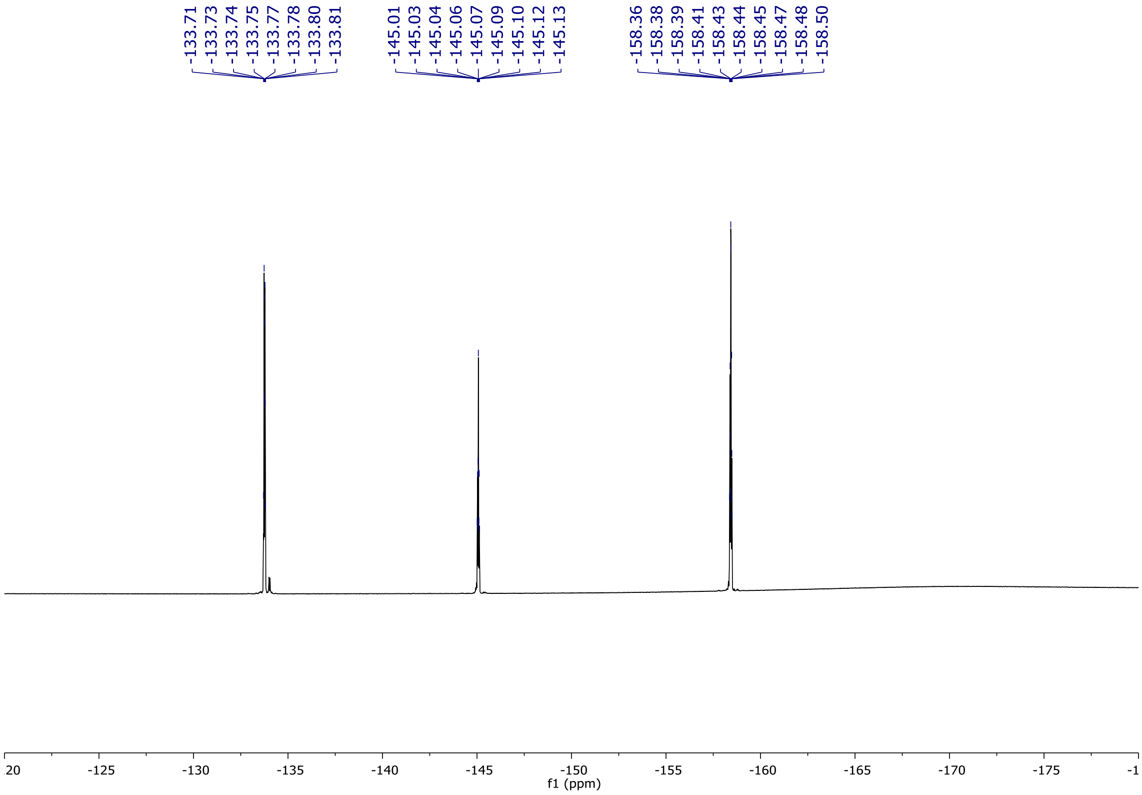

DEPT-135

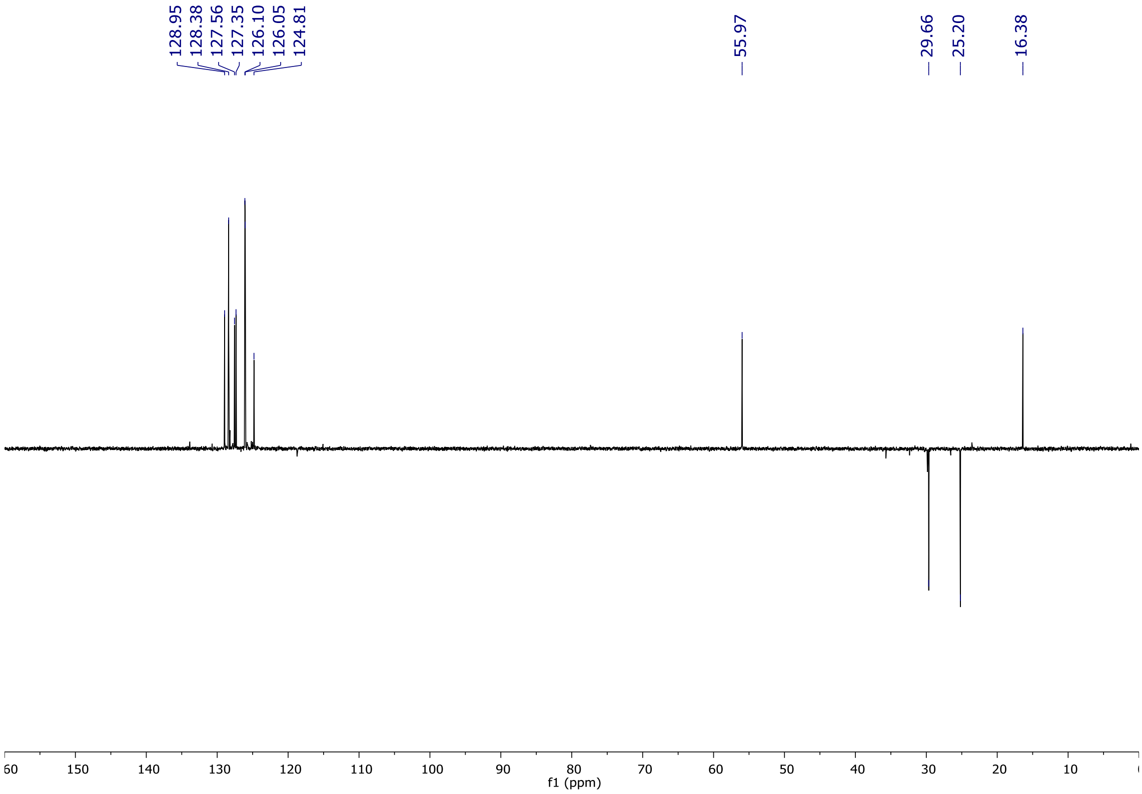

^{13}C NMR (126 MHz, $CDCl_3$)

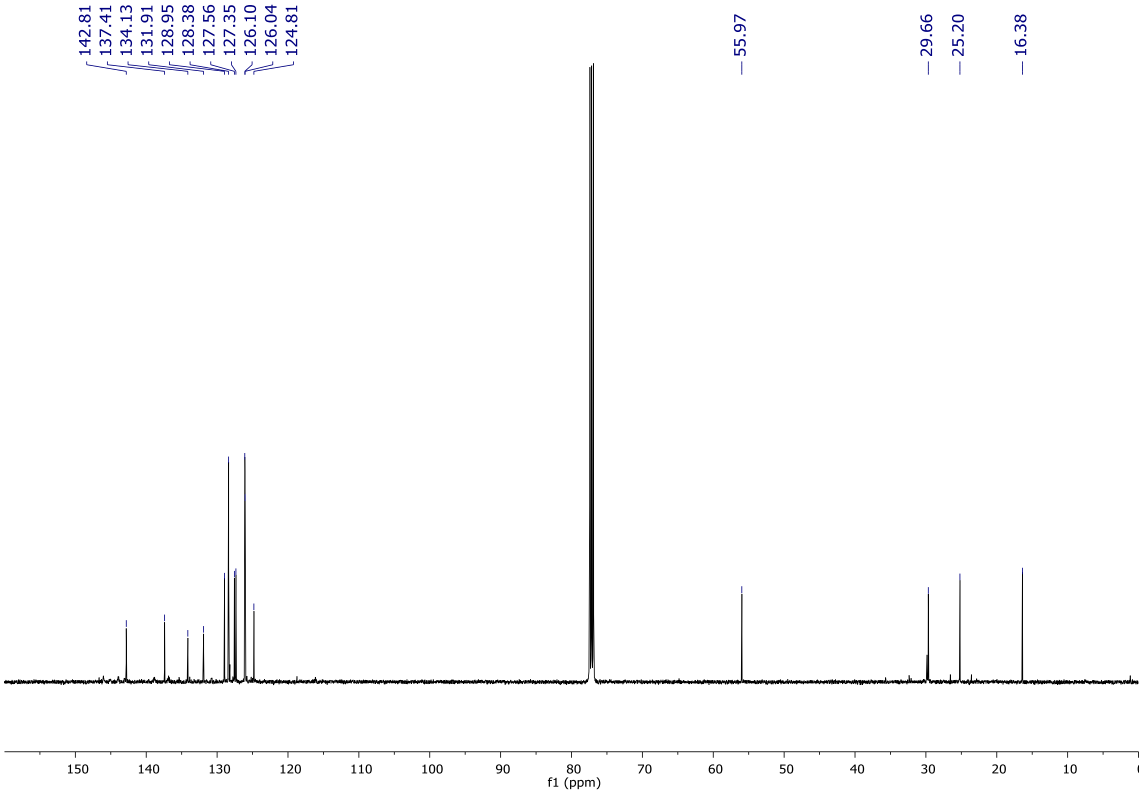

^{1}H NMR (500 MHz, $CDCl_3$)

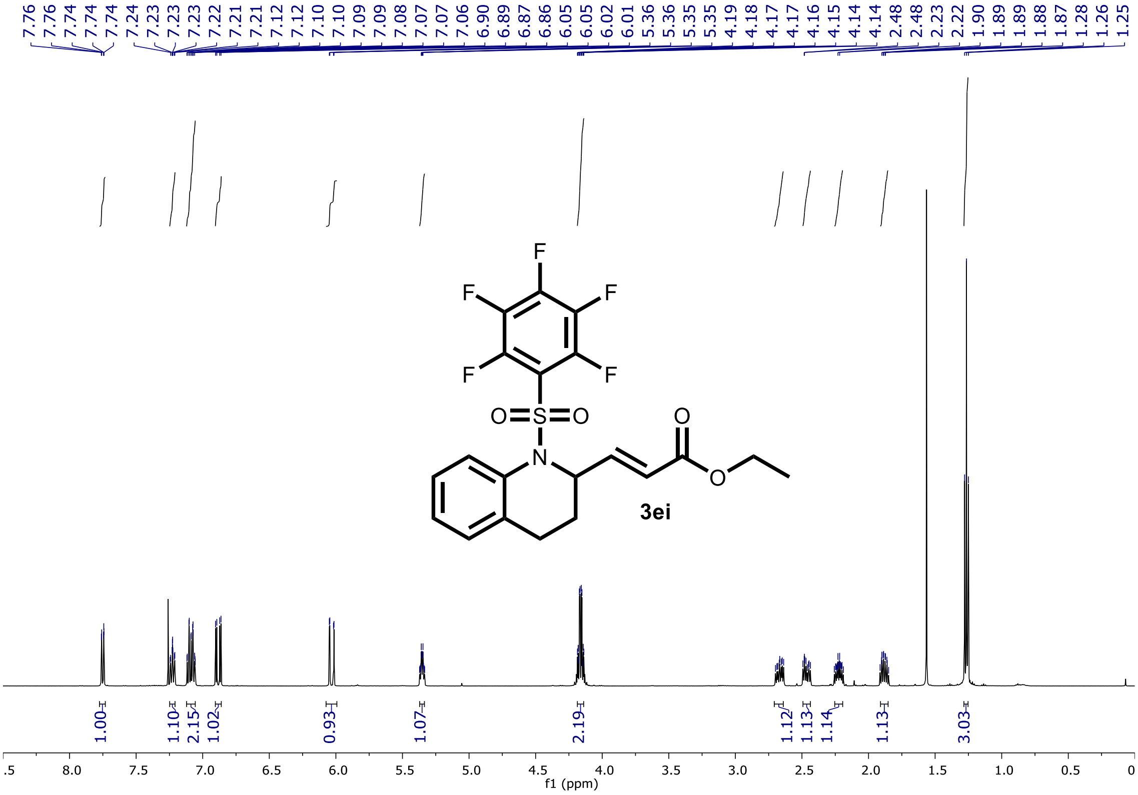

^{19}F NMR (471 MHz, $CDCl_3$)

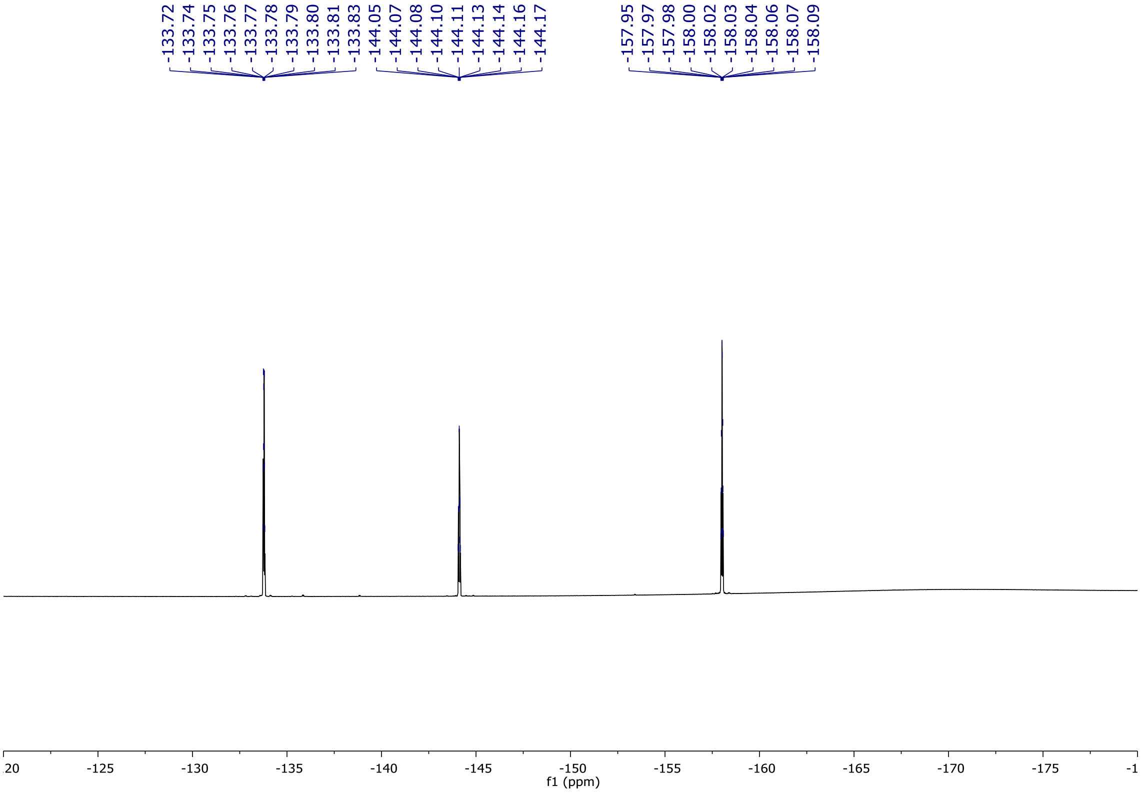

DEPT-135

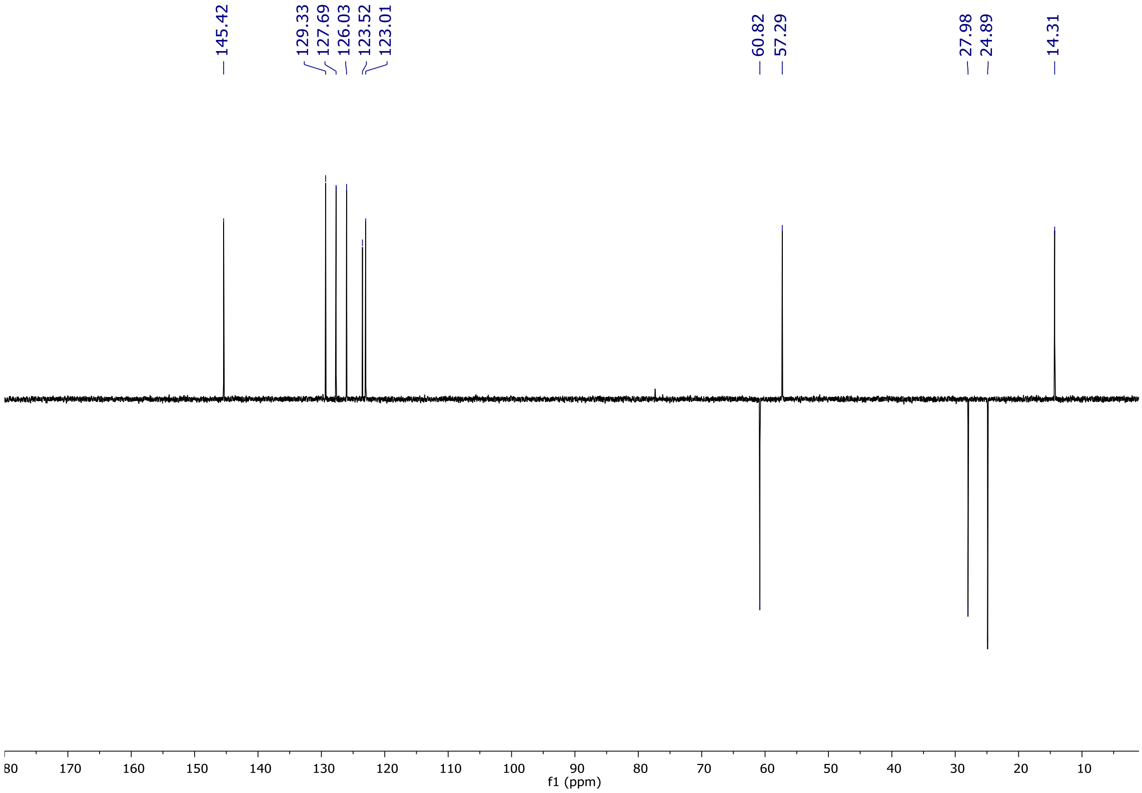

^{13}C NMR (126 MHz, $CDCl_3$)

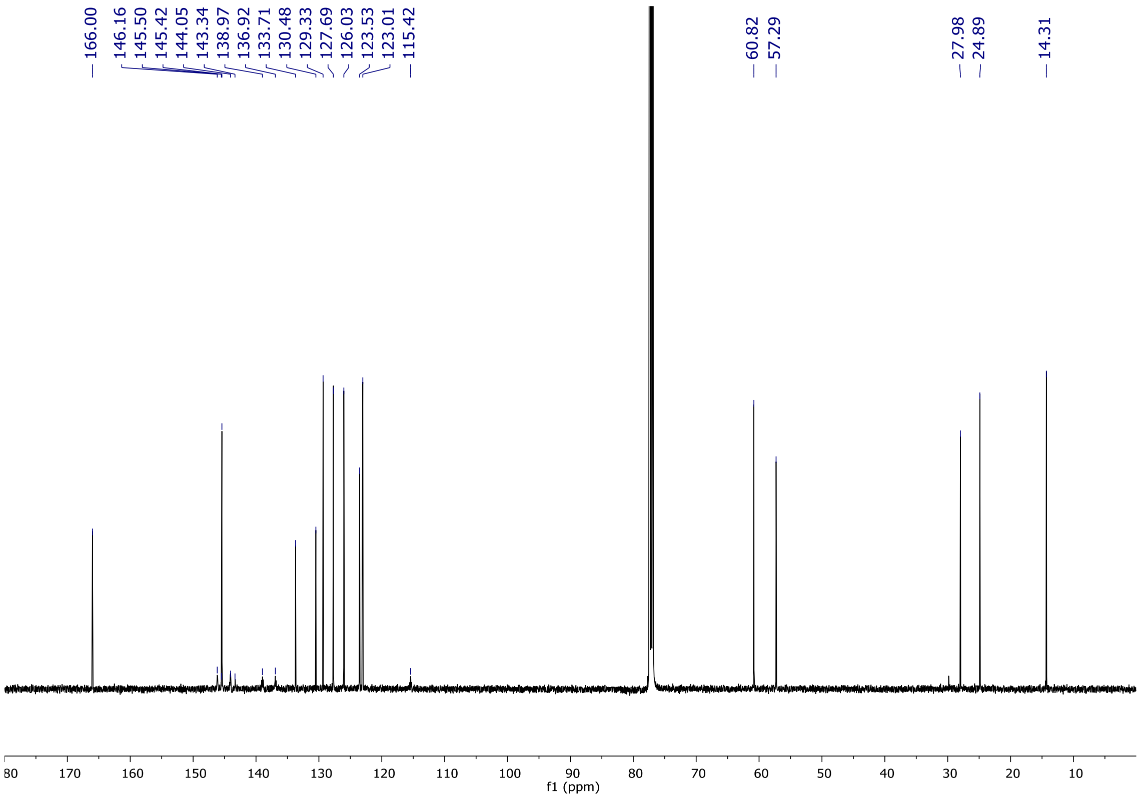

^{1}H NMR (500 MHz, $CDCl_3$)

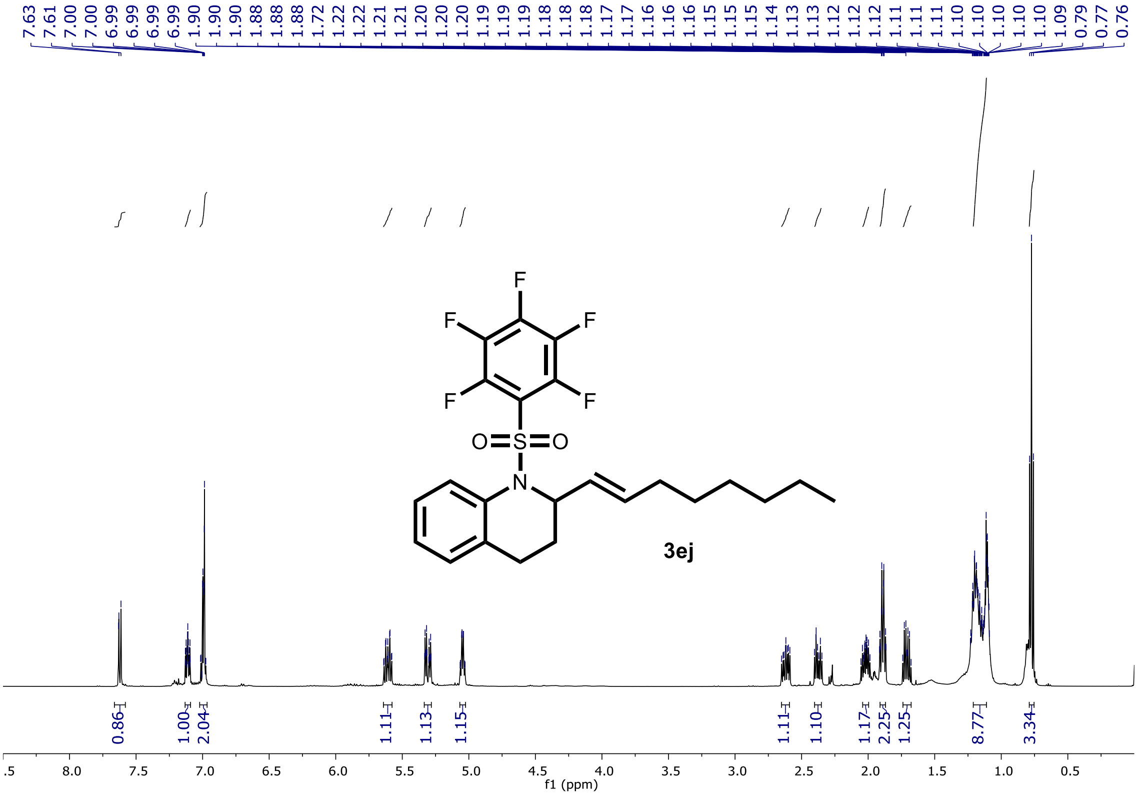

^{19}F NMR (471 MHz, $CDCl_3$)

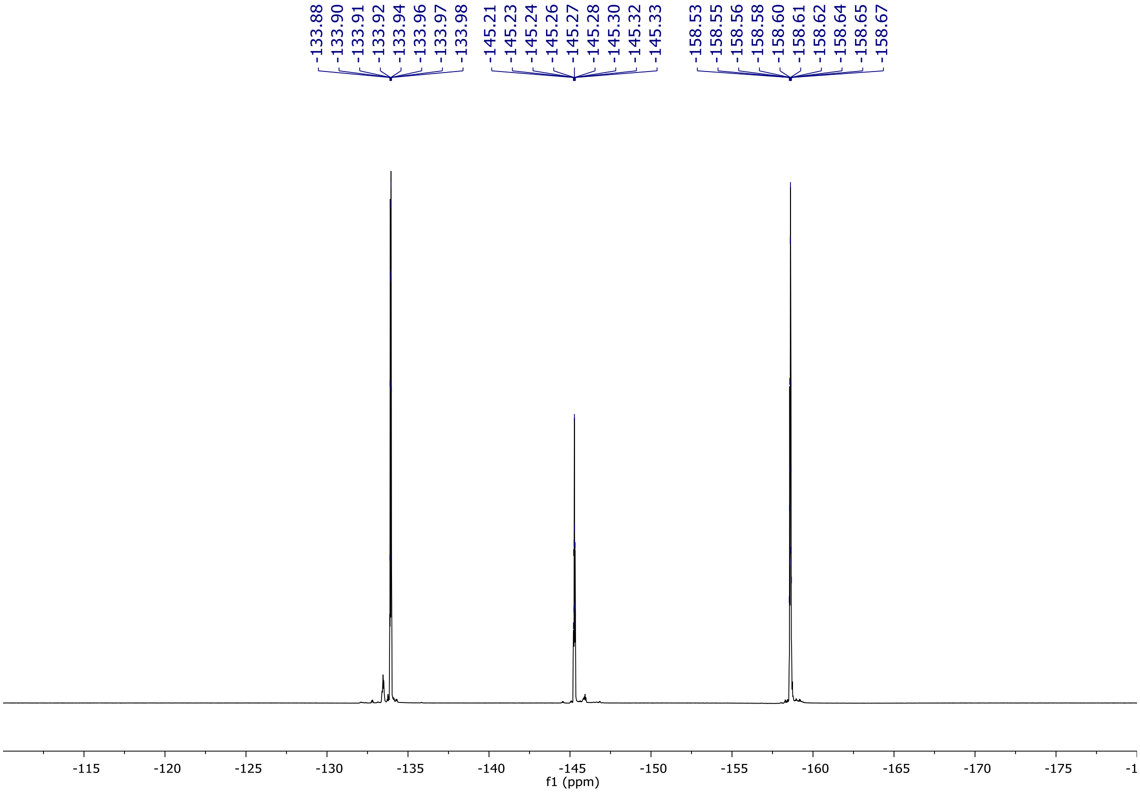

DEPT-135

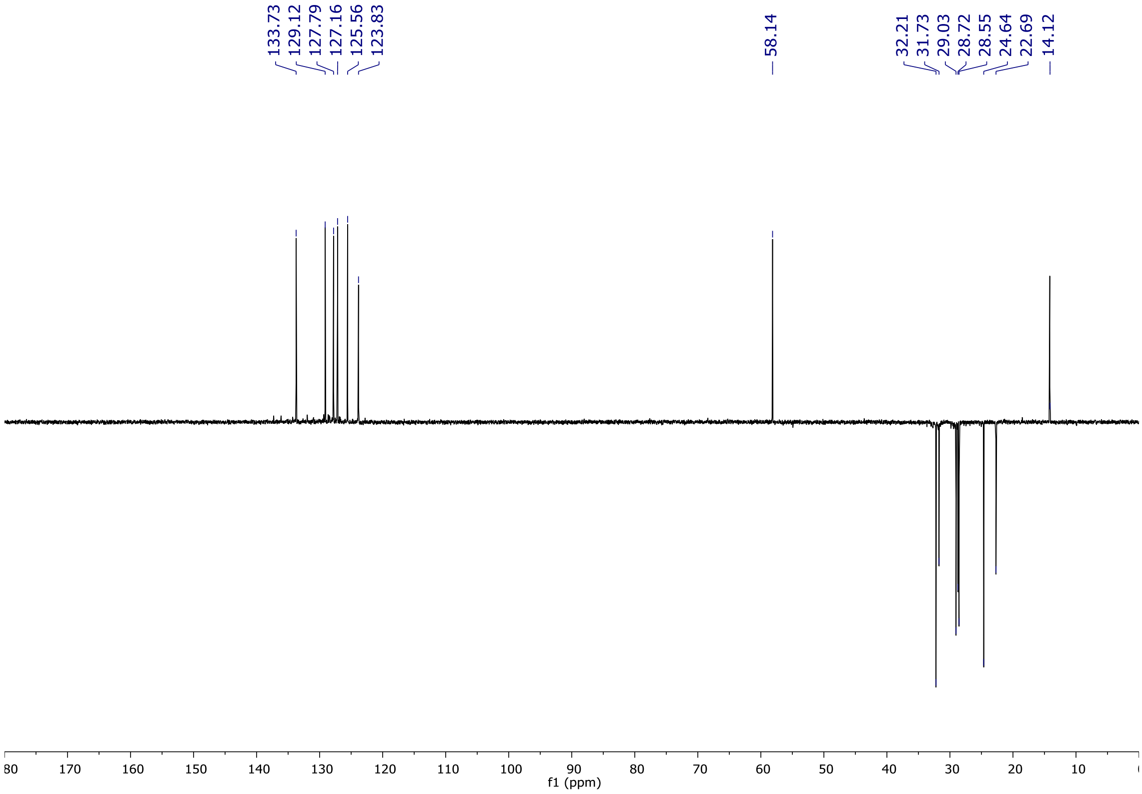

^{13}C NMR (126 MHz, $CDCl_3$)

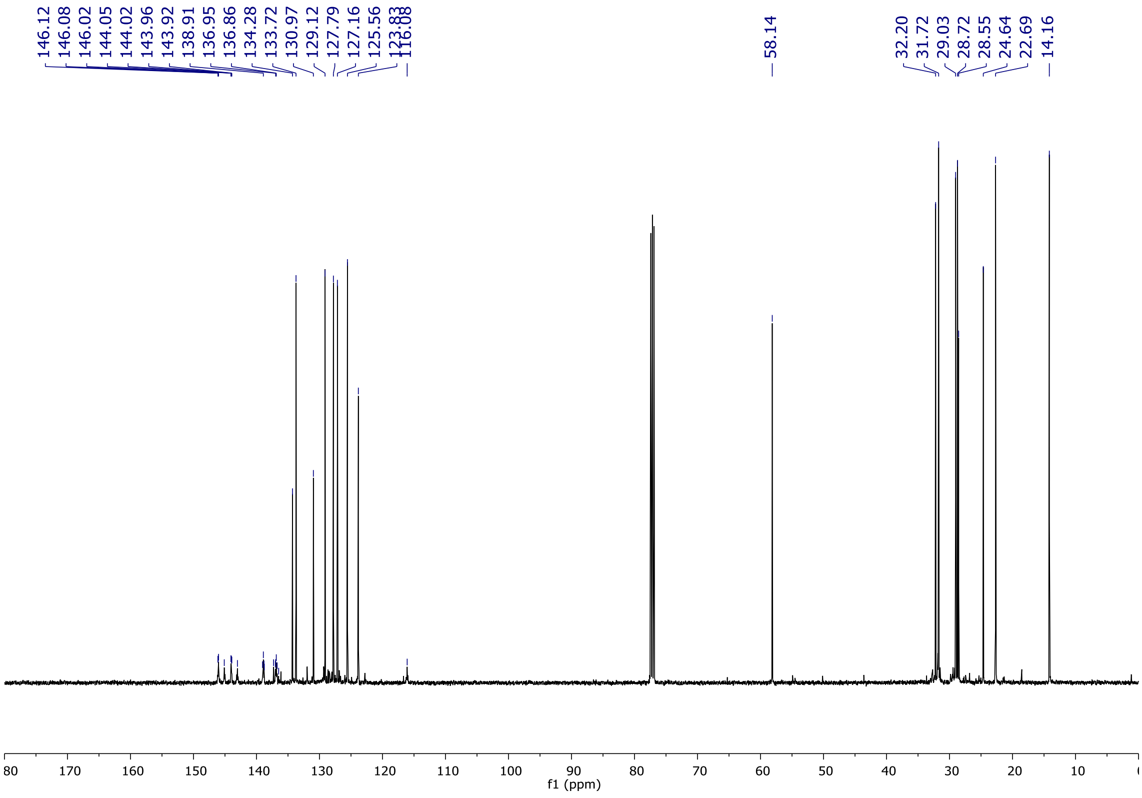

^{1}H NMR (500 MHz, $CDCl_3$)

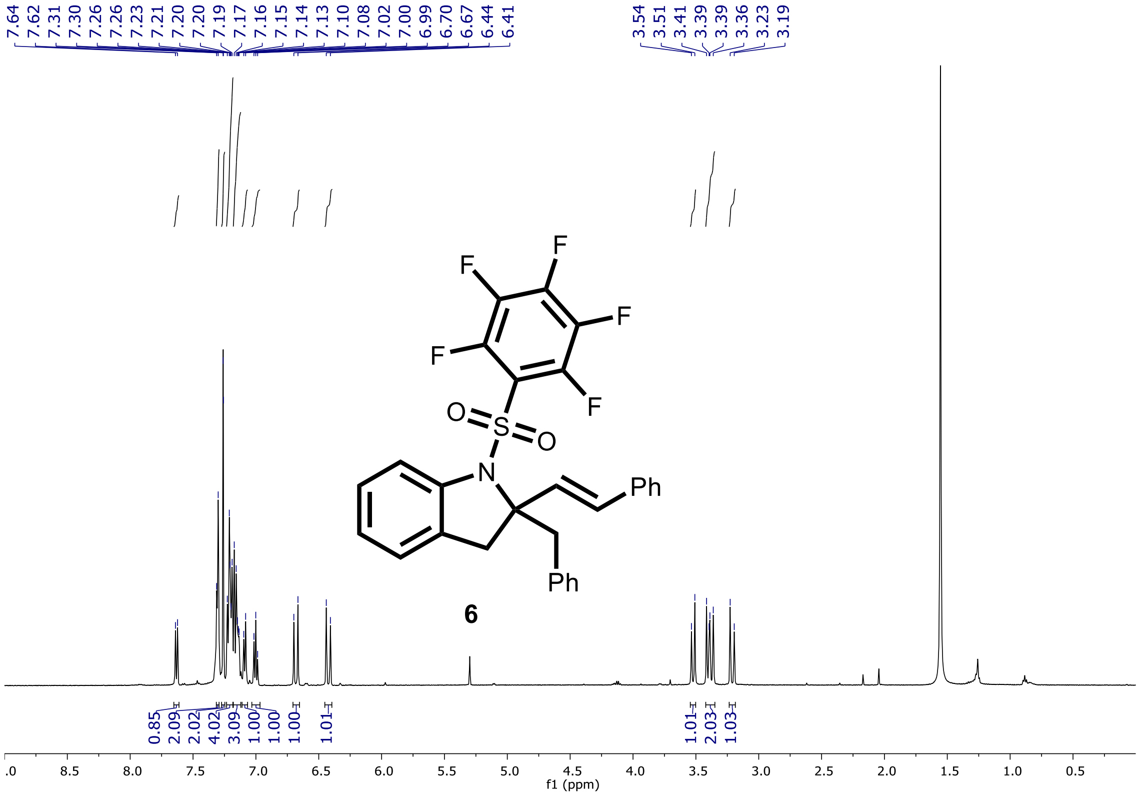

^{19}F NMR (471 MHz, $CDCl_3$)

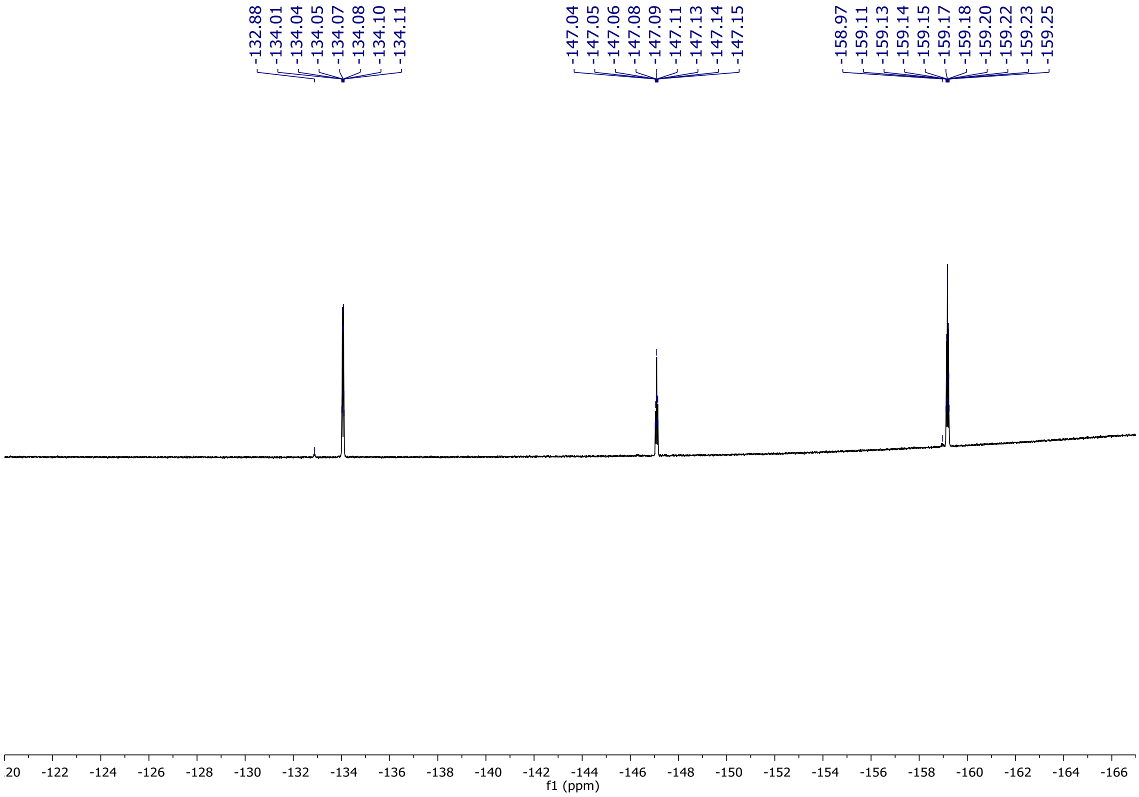

^{13}C NMR (126 MHz, $CDCl_3$)

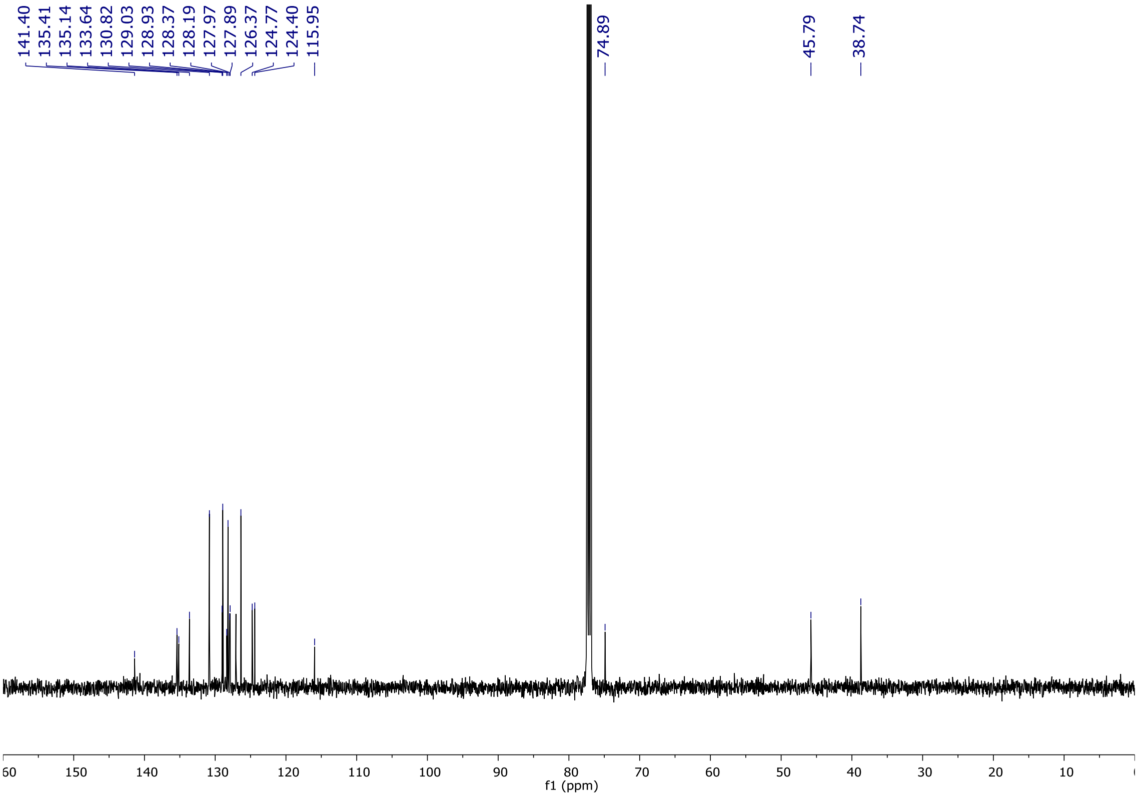

^{1}H NMR (300 MHz, $CDCl_3$)

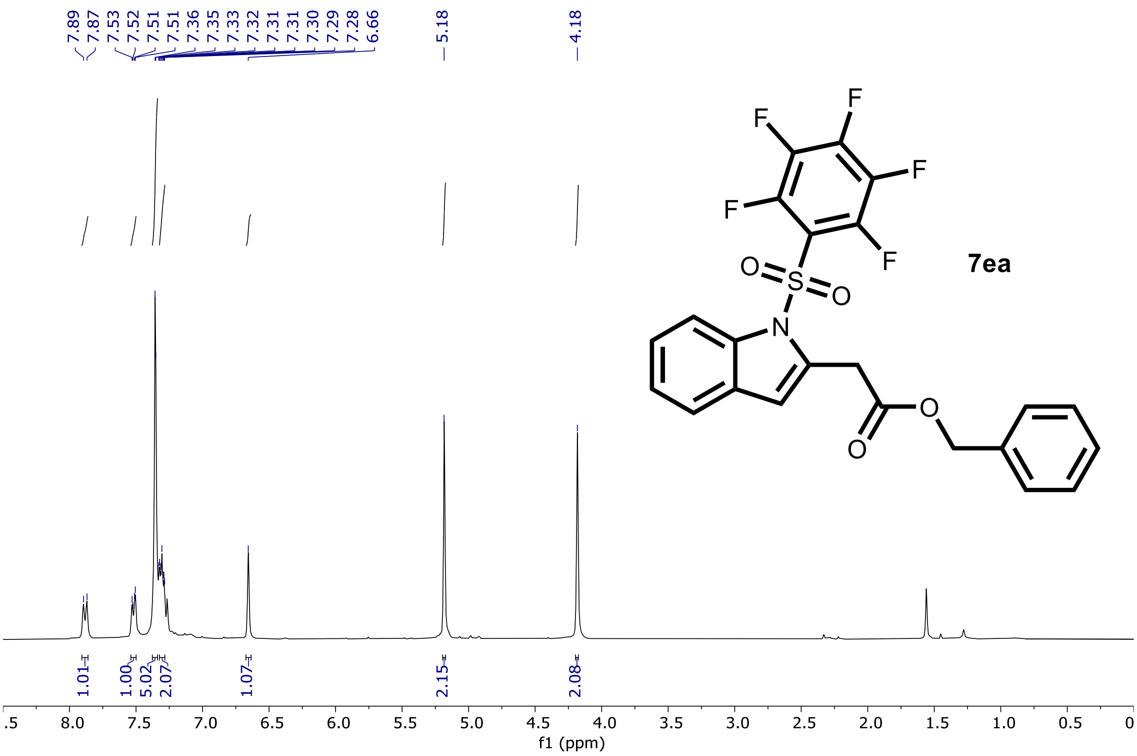

^{19}F NMR (282 MHz, $CDCl_3$)

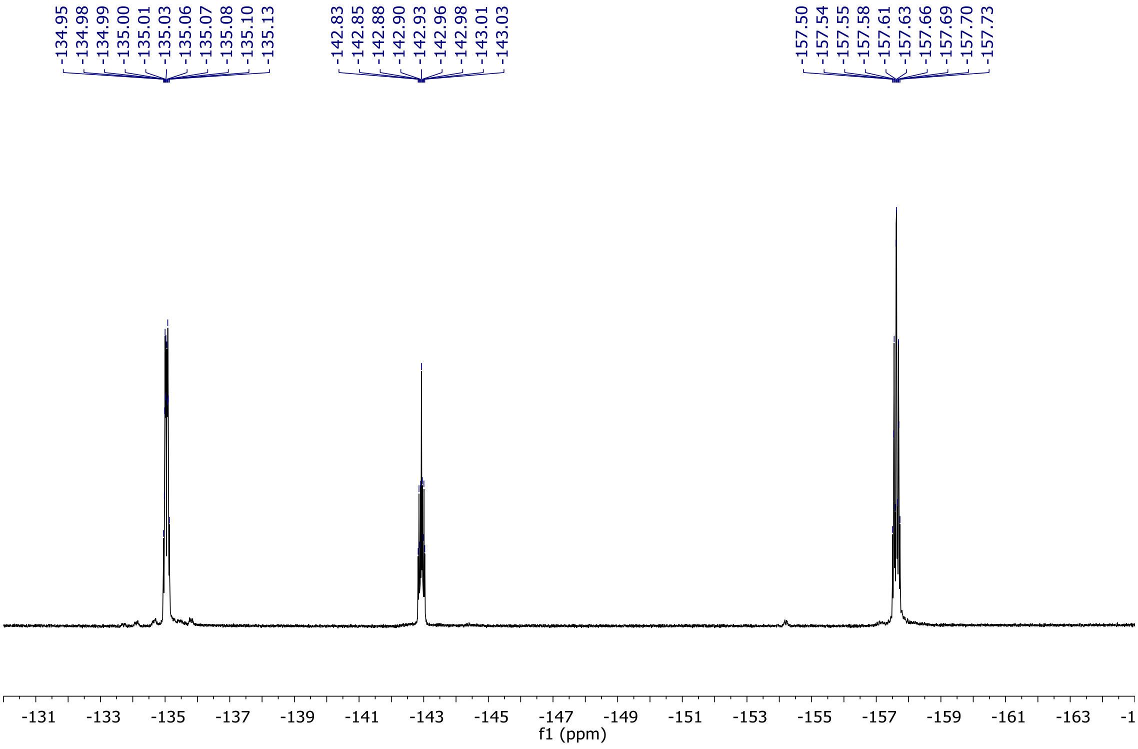

DEPT-135

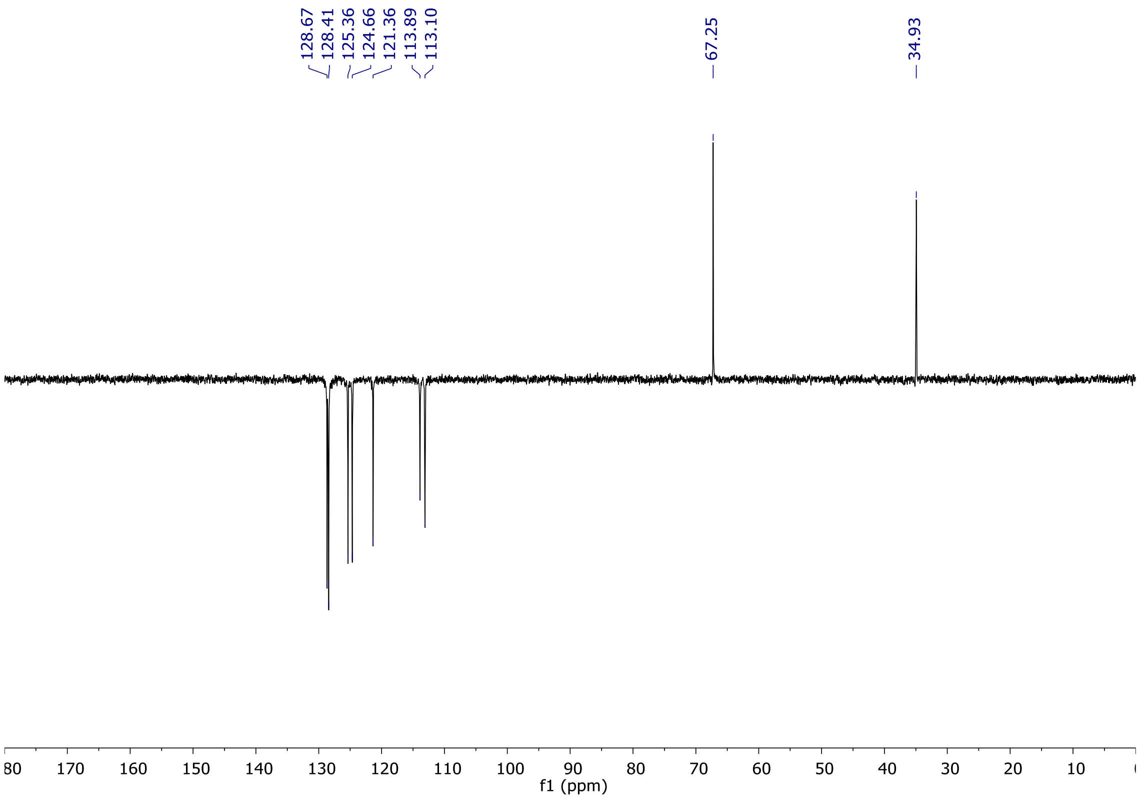

^{13}C NMR (75 MHz, $CDCl_3$)

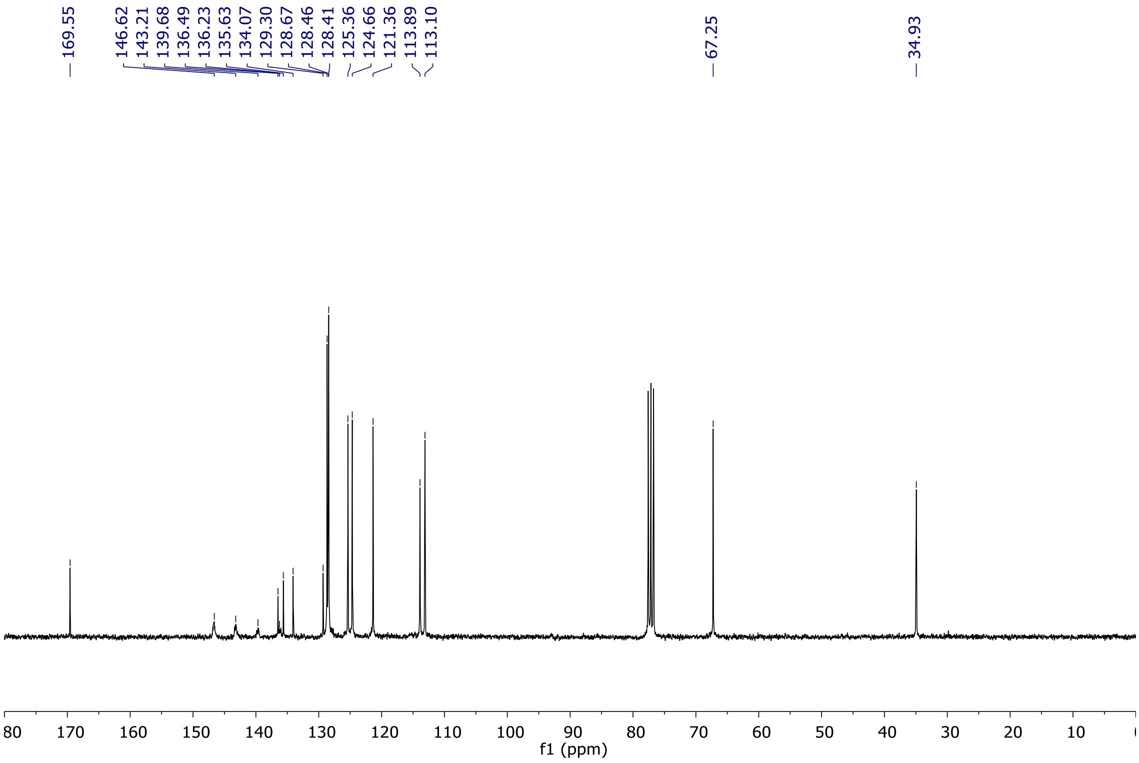

^{1}H NMR (500 MHz, CDCl$_3$)

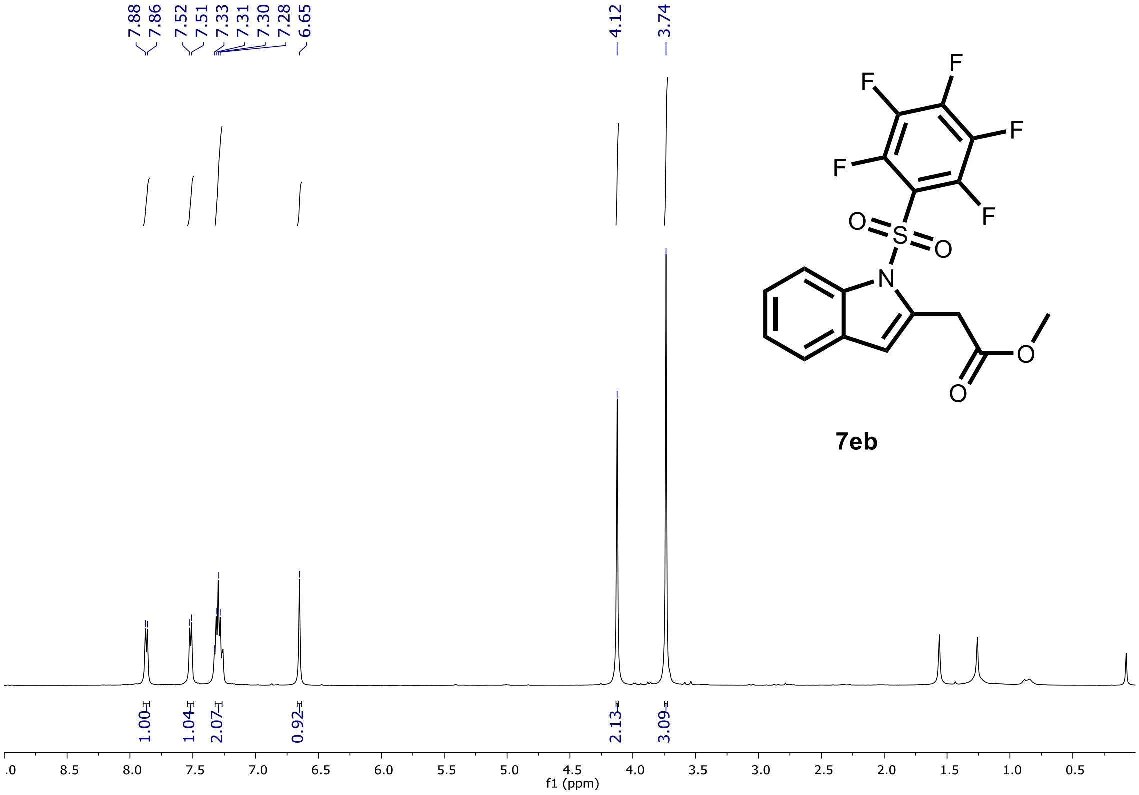

7eb

^{19}F NMR (471 MHz, CDCl$_3$)

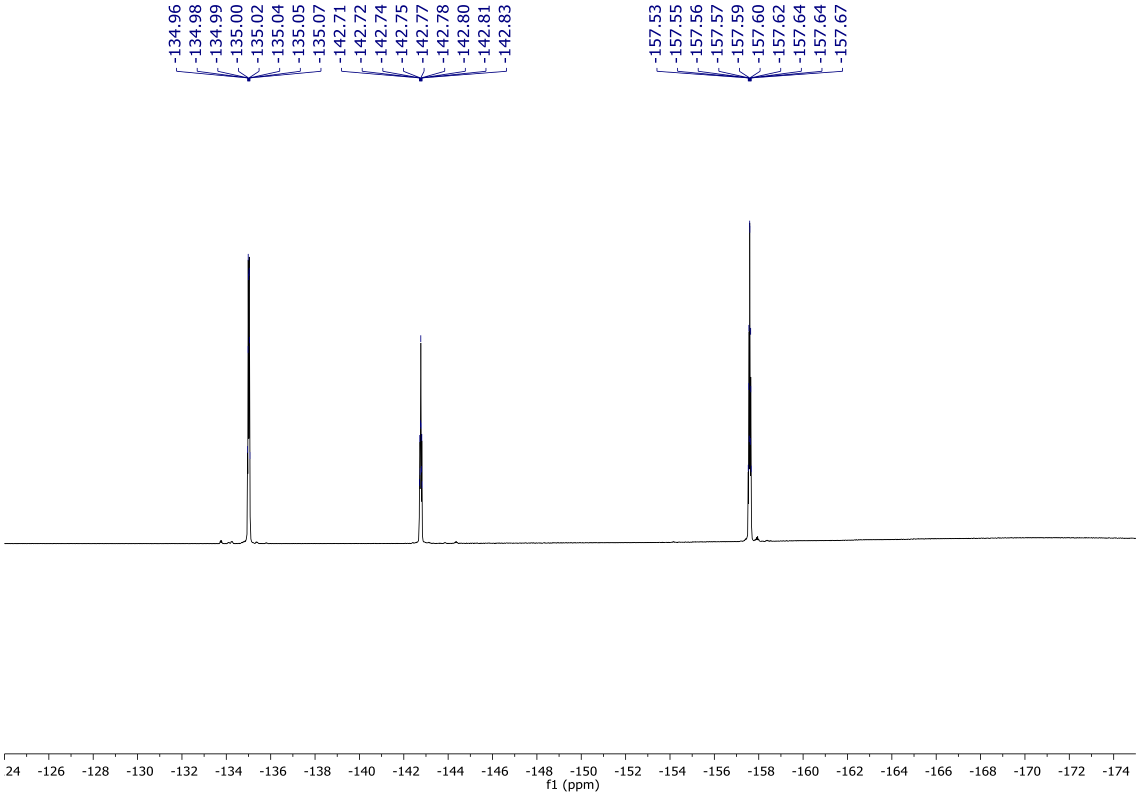

DEPT-135

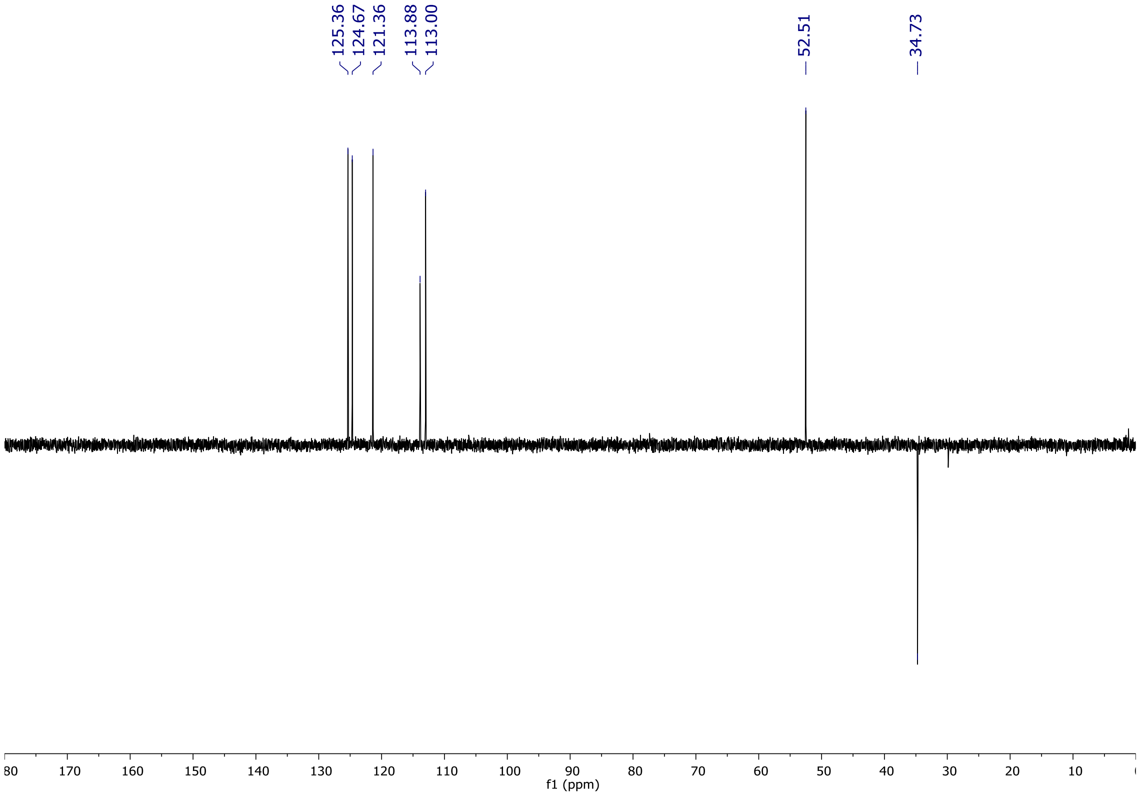

^{13}C NMR (126 MHz, $CDCl_3$)

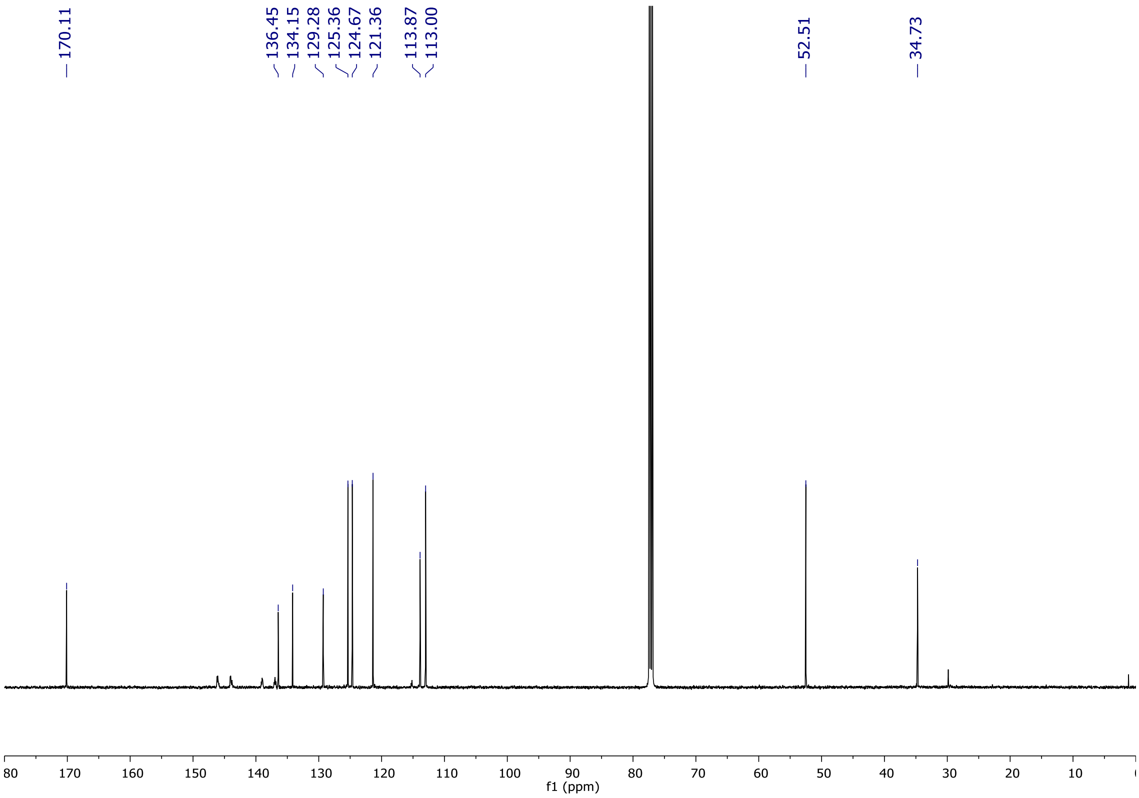

^{1}H NMR (500 MHz, $CDCl_3$)

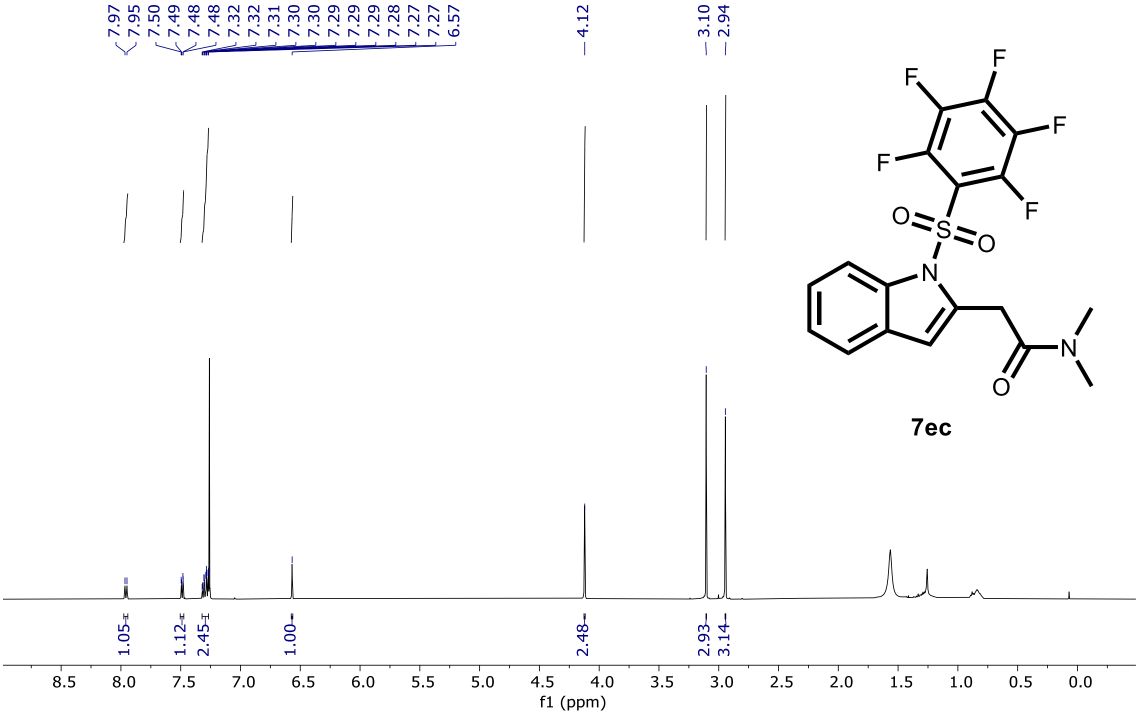

^{19}F NMR (471 MHz, $CDCl_3$)

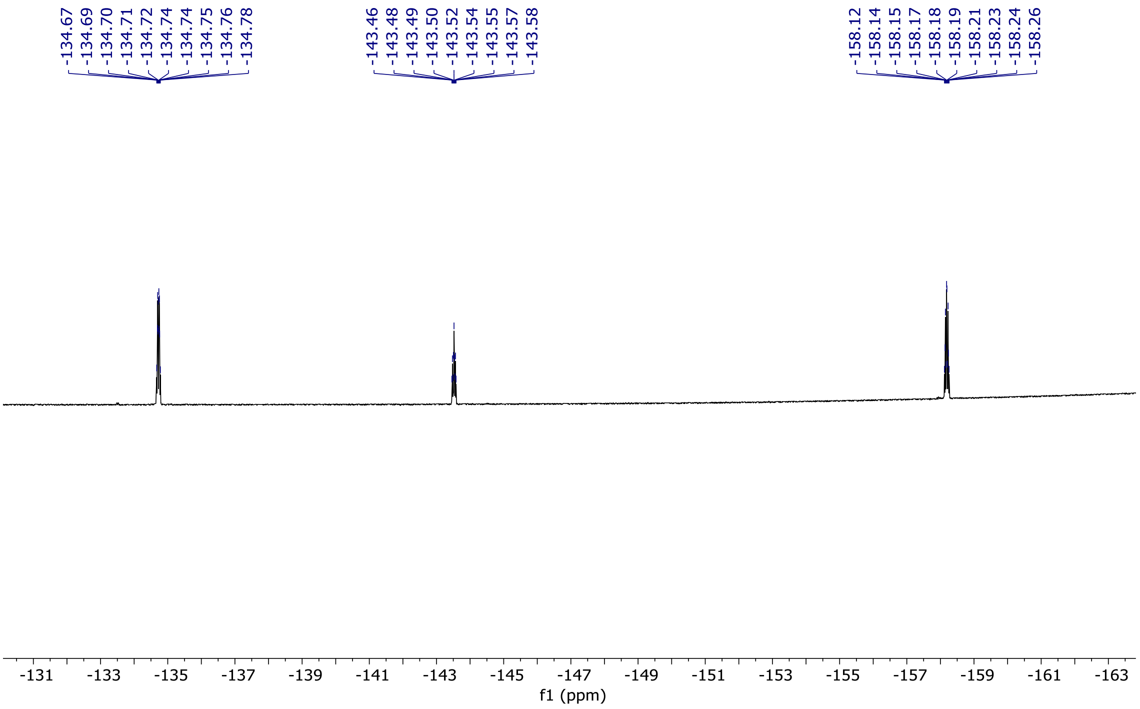

DEPT-135

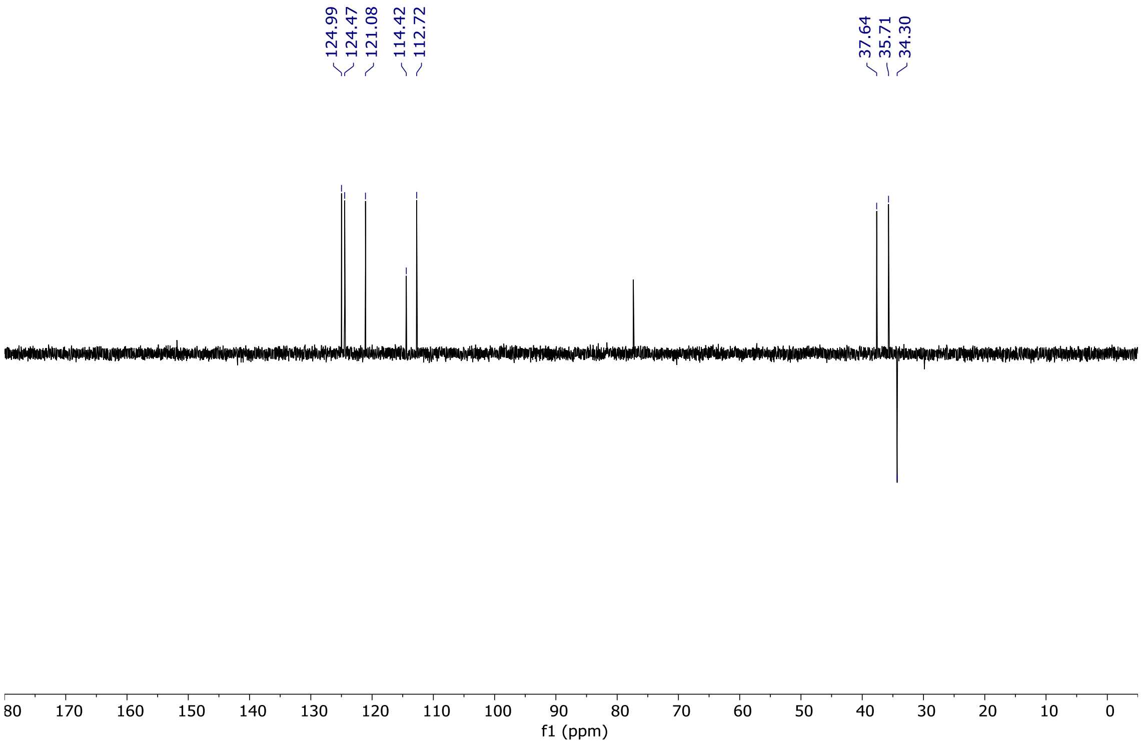

^{13}C NMR (126 MHz, $CDCl_3$)

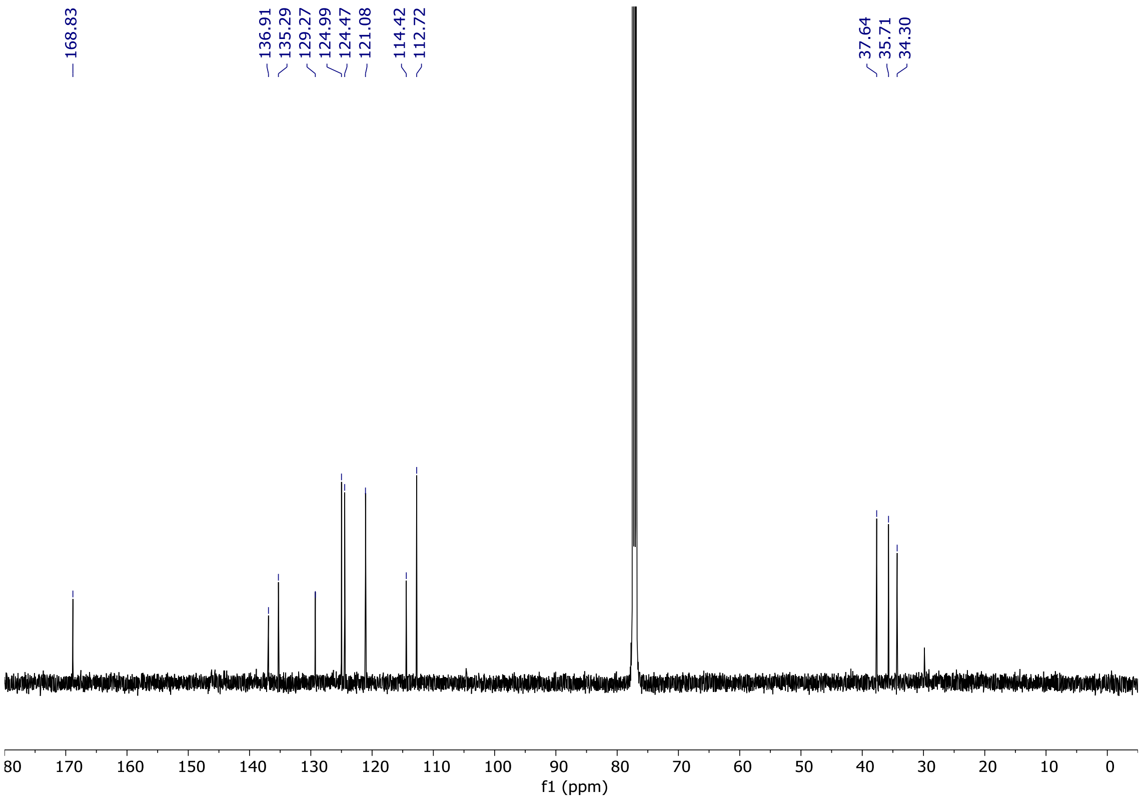

^{1}H NMR (300 MHz, CDCl$_3$)

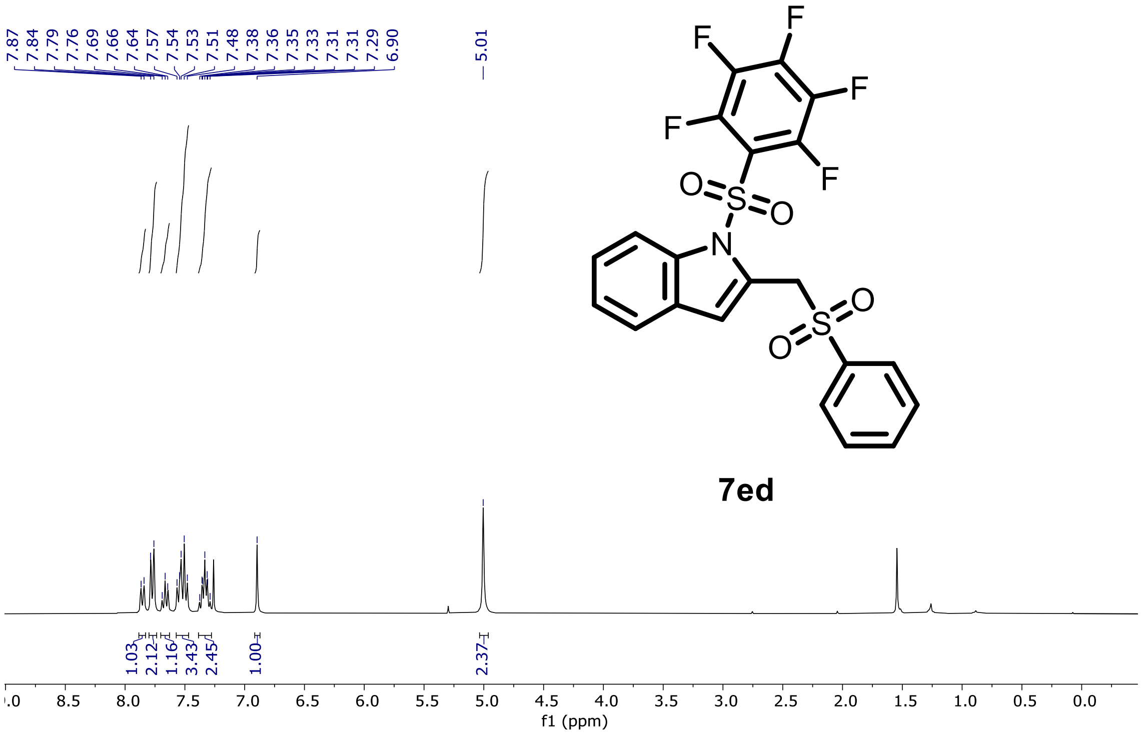

7ed

^{19}F NMR (282 MHz, CDCl$_3$)

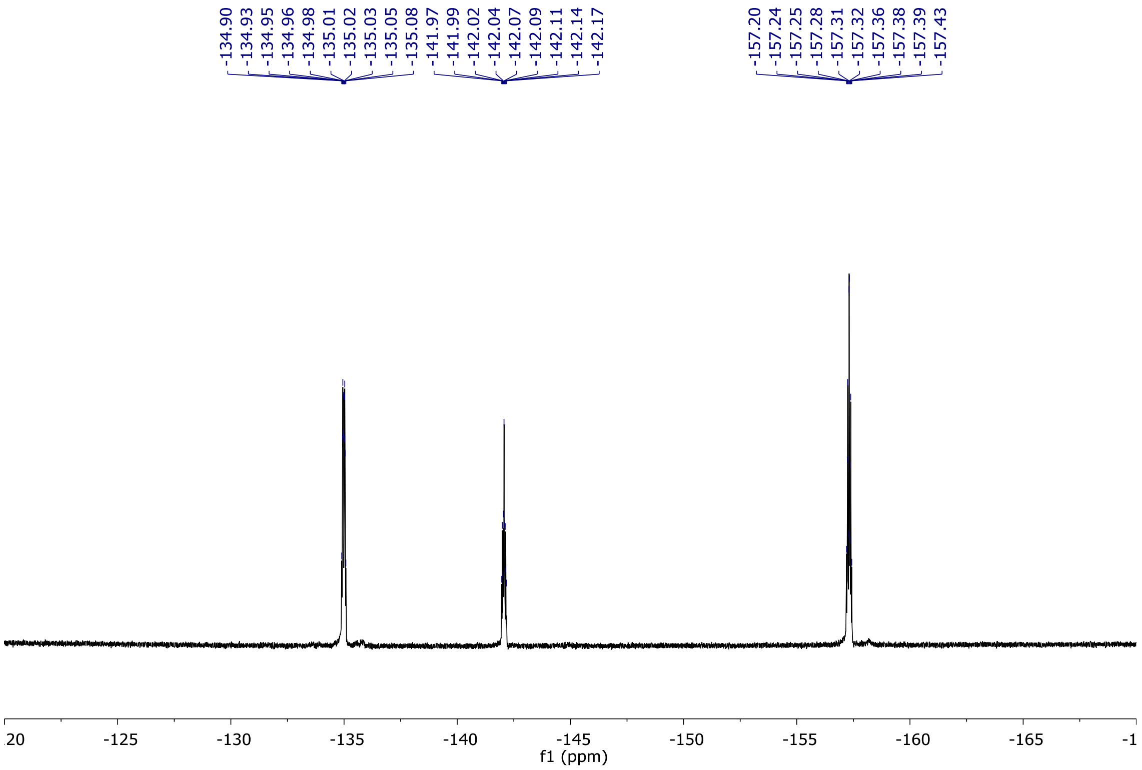

DEPT-135

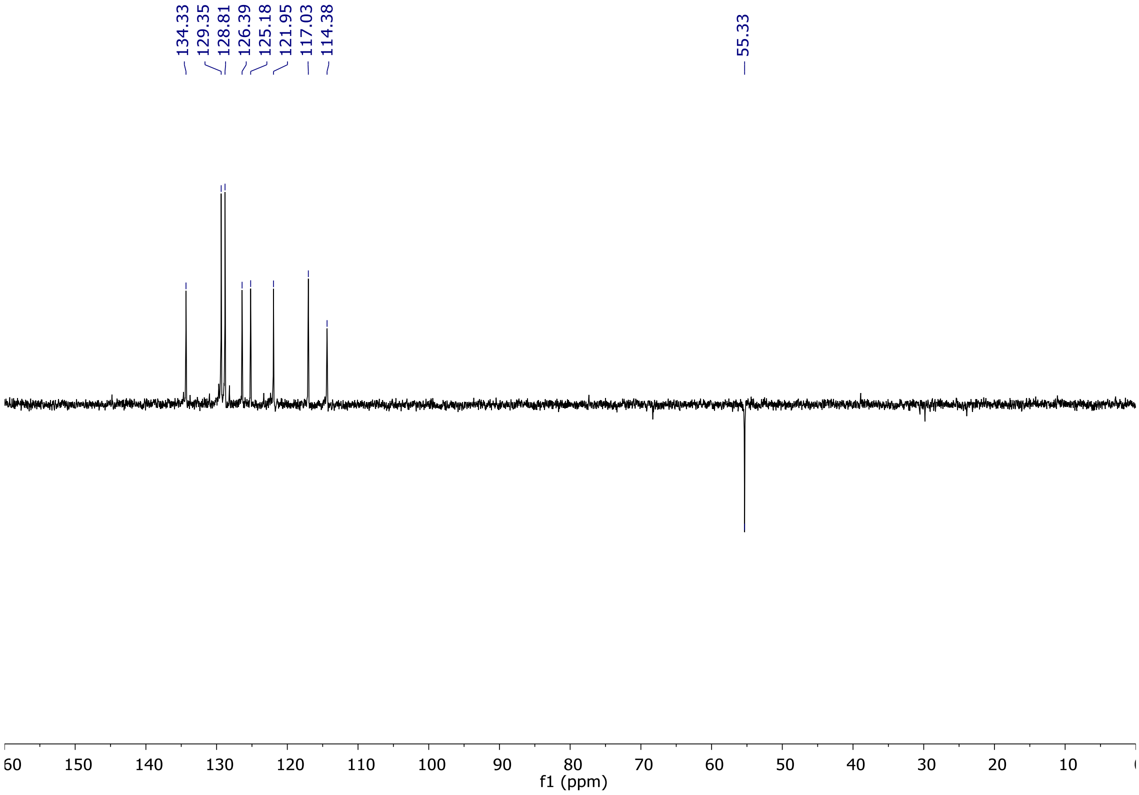

^{13}C NMR (75 MHz, $CDCl_3$)

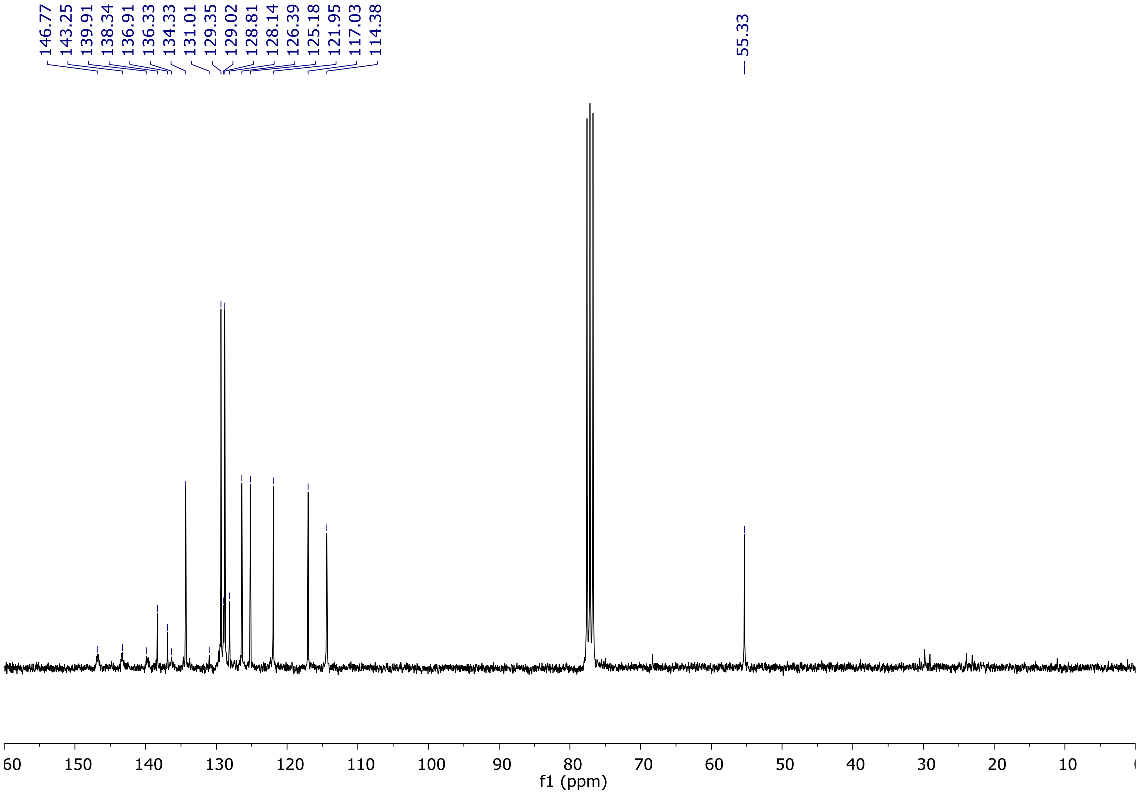

^{1}H NMR (500 MHz, $CDCl_3$)

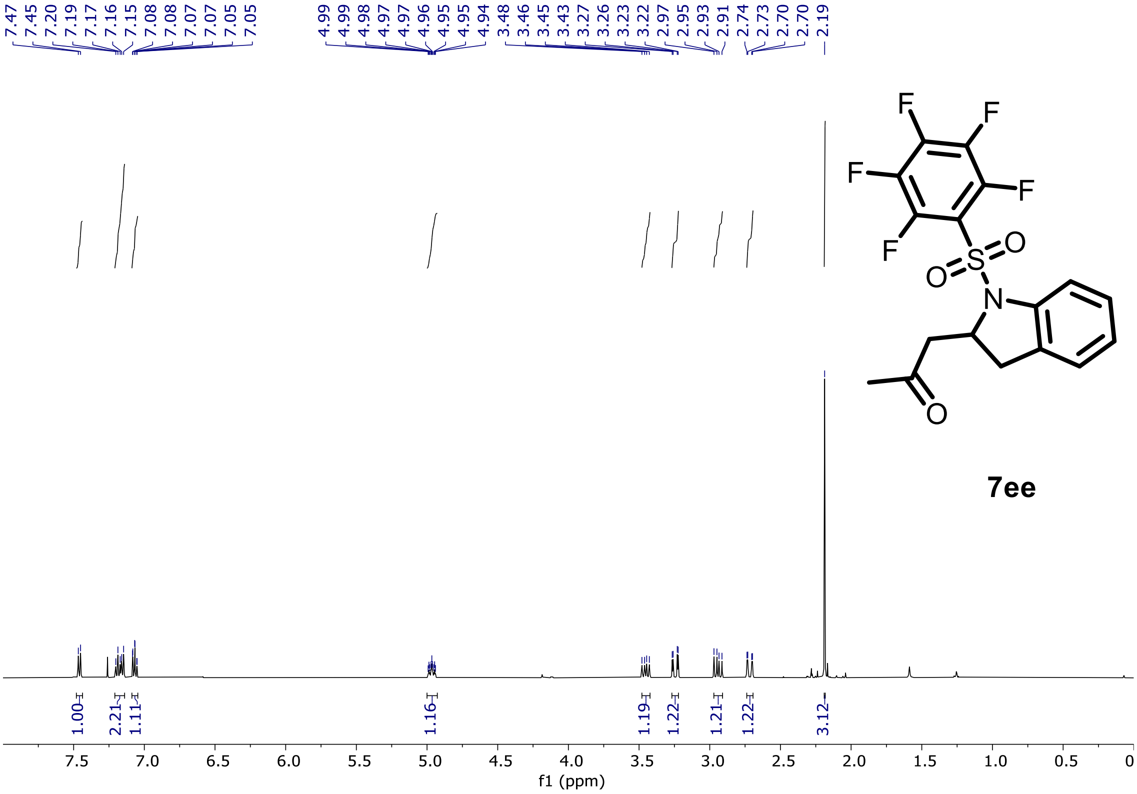

^{19}F NMR (471 MHz, $CDCl_3$)

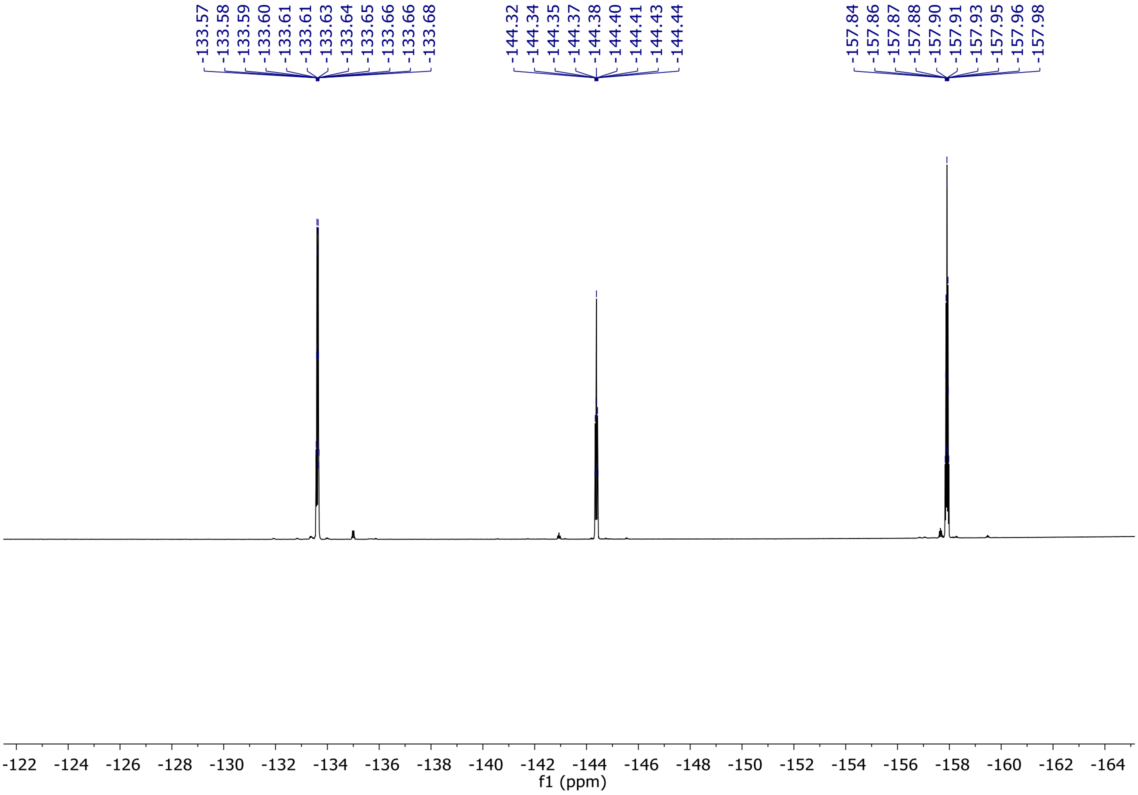

DEPT-135

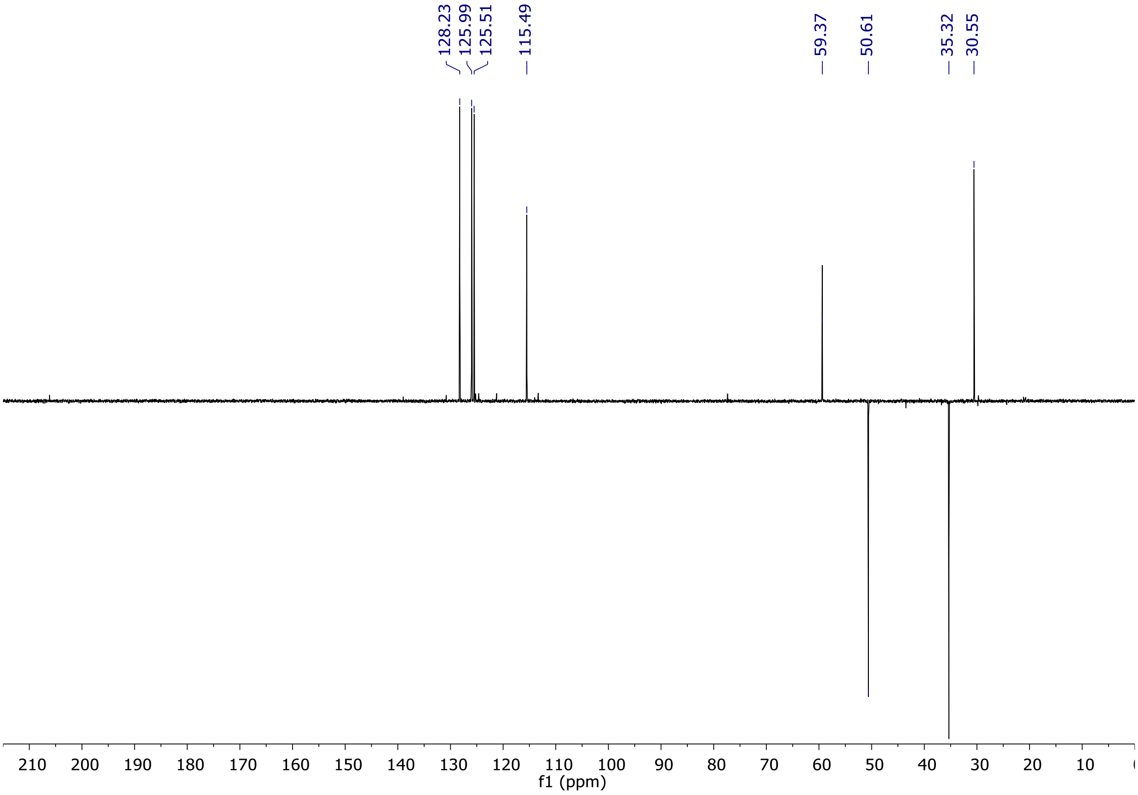

^{13}C NMR (126 MHz, $CDCl_3$)

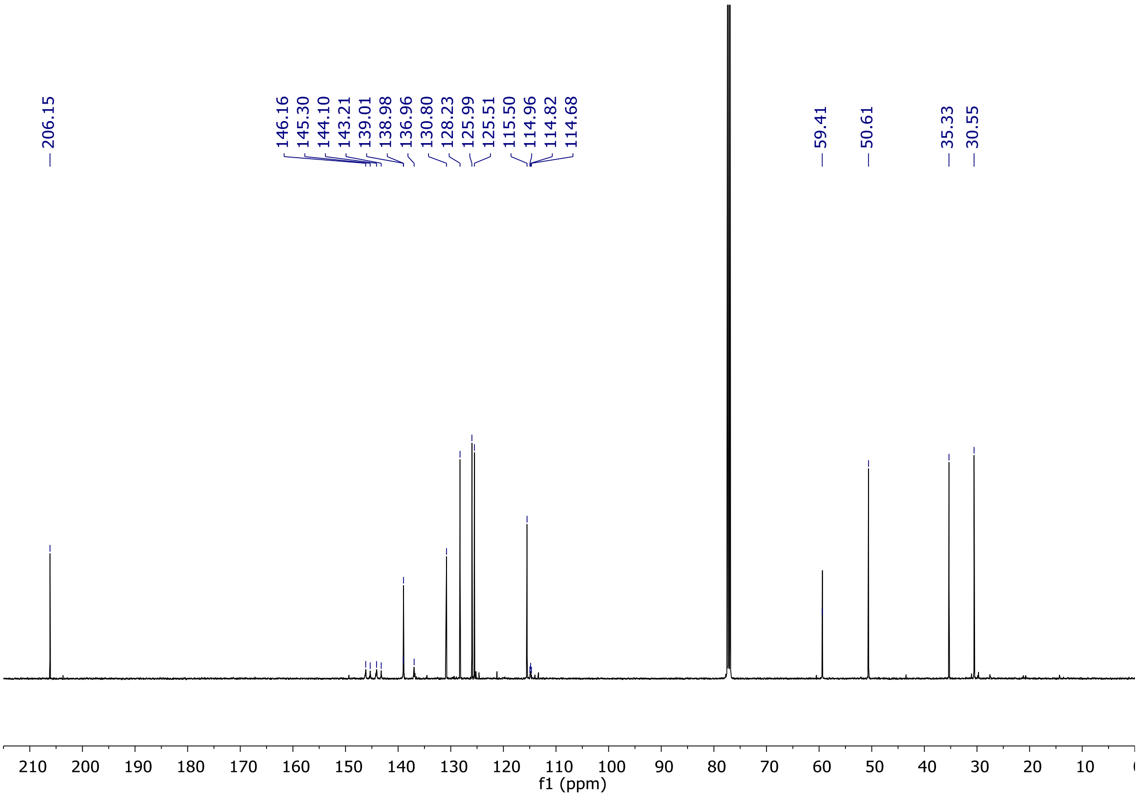

^{1}H NMR (500 MHz, $CDCl_3$)

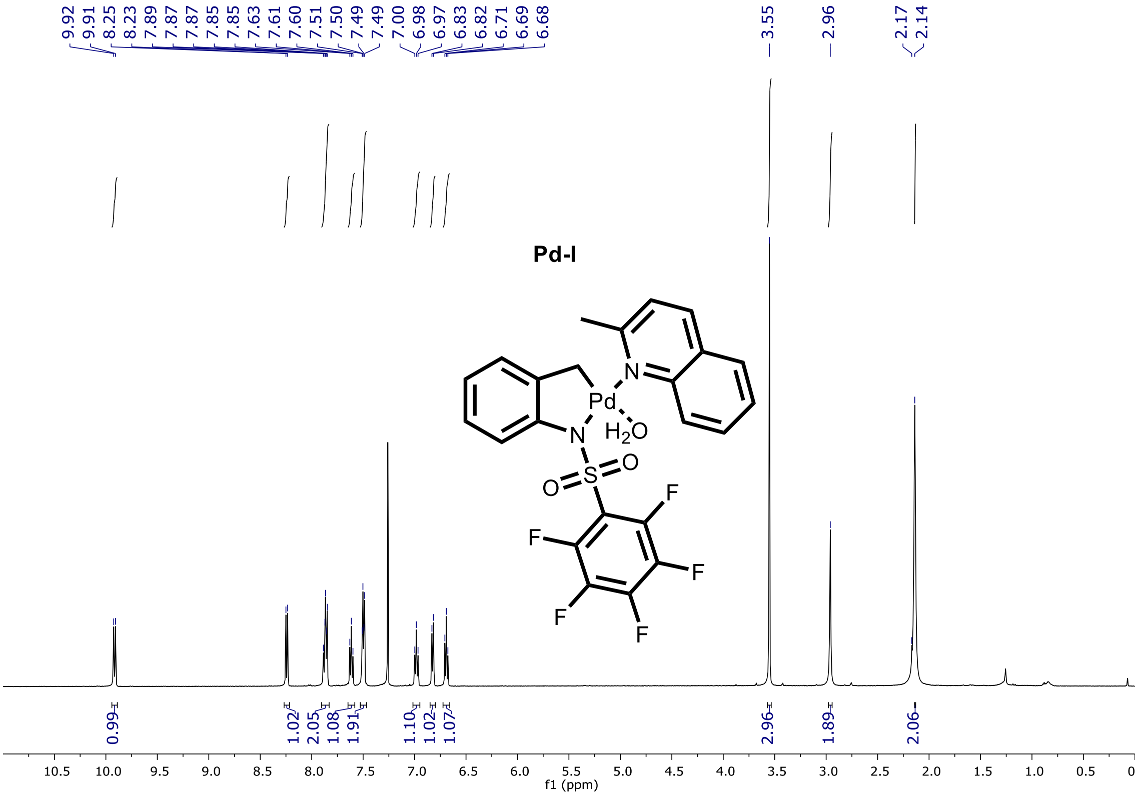

^{19}F NMR (471 MHz, $CDCl_3$)

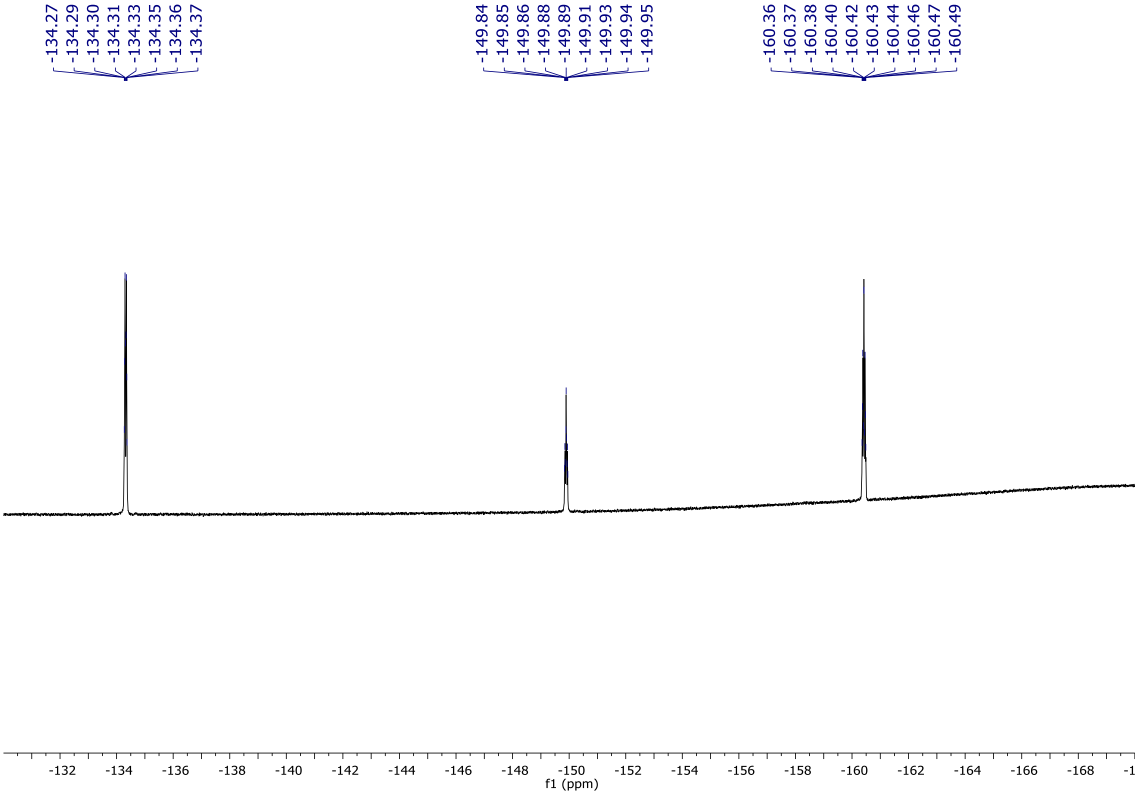

DEPT-135

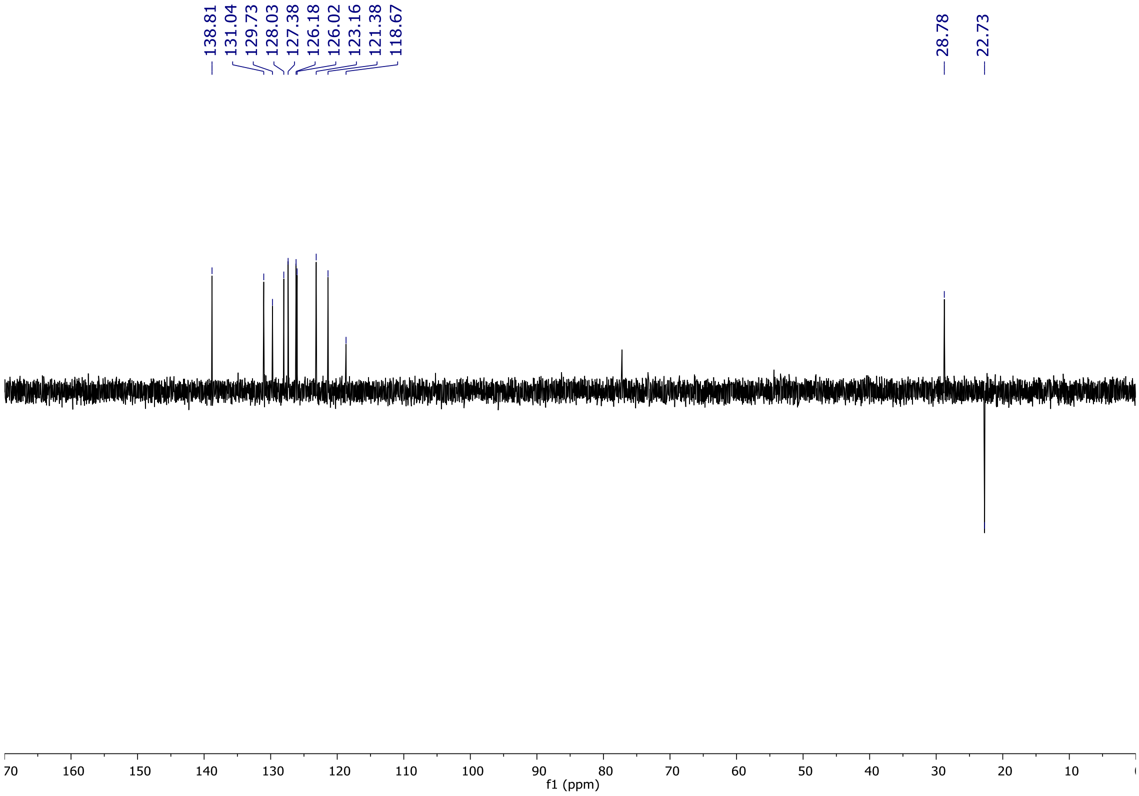

^{13}C NMR (126 MHz, $CDCl_3$)

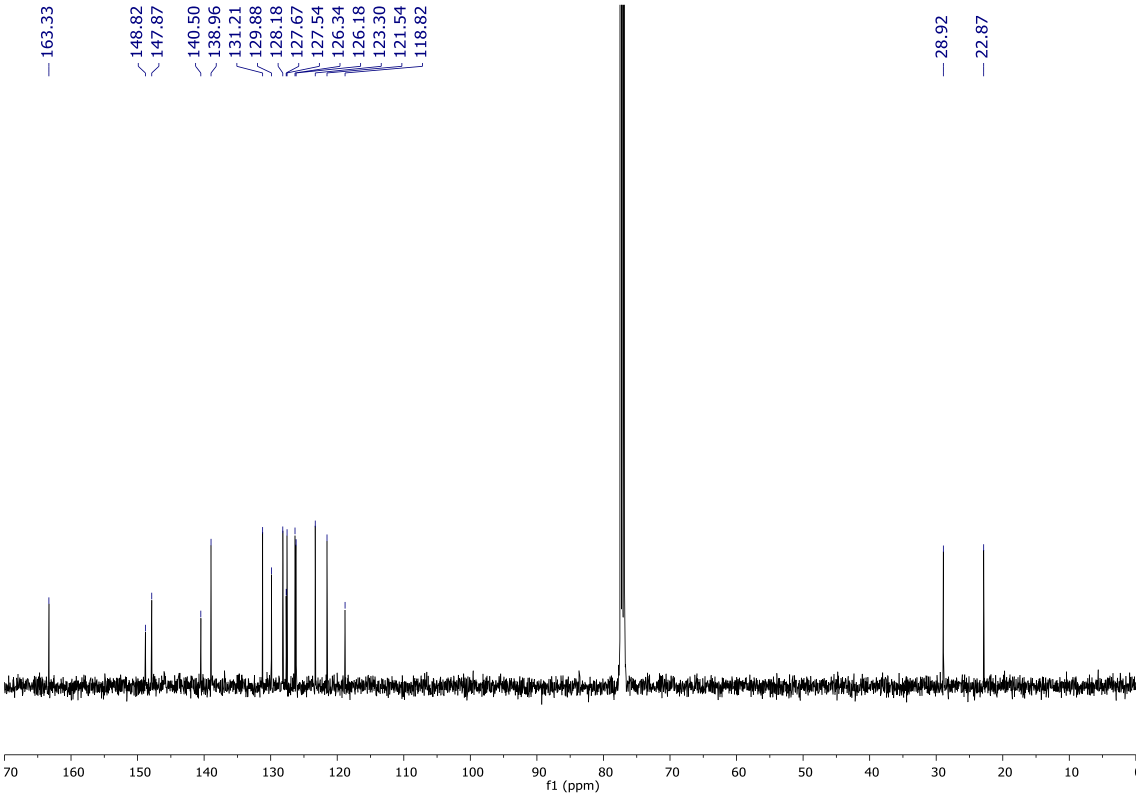

^{1}H NMR (500 MHz, $CDCl_3$)

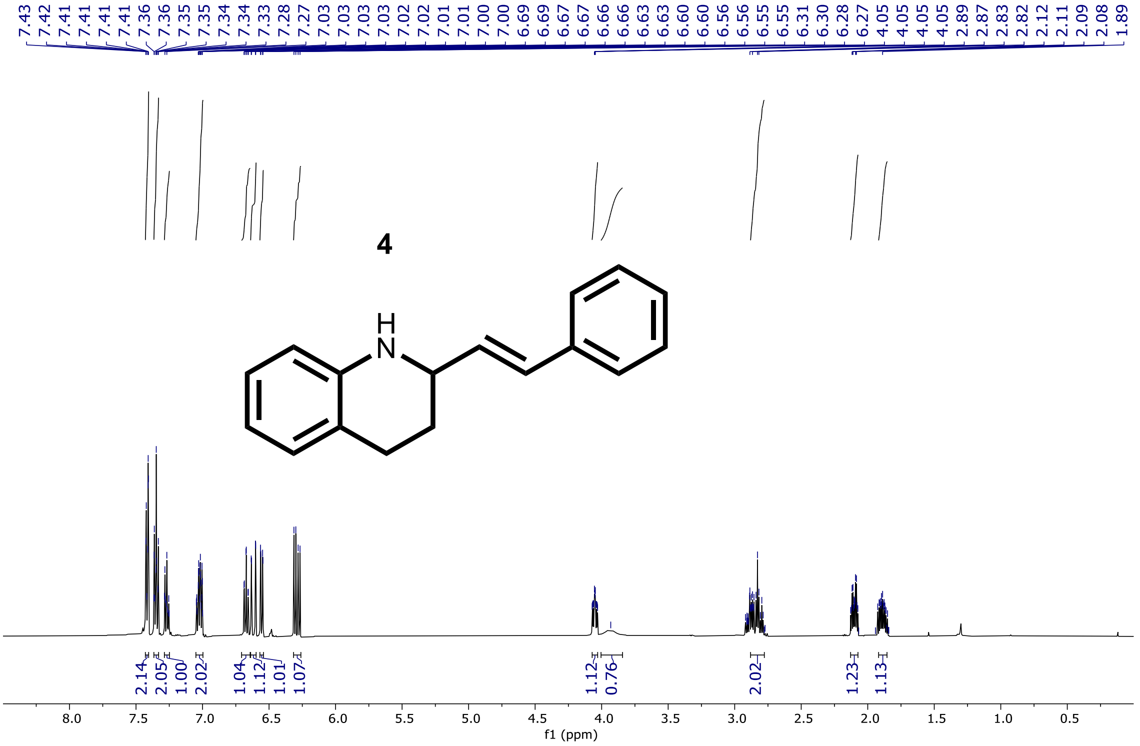

DEPT-135

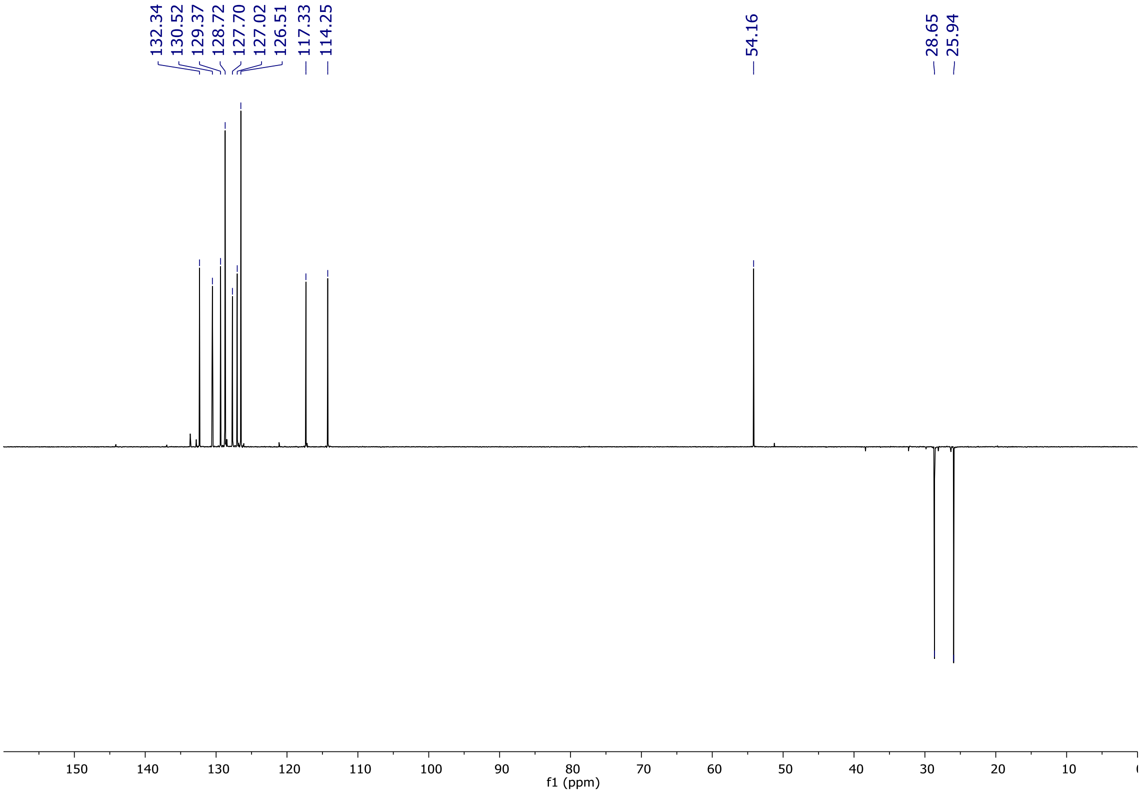

^{13}C NMR (126 MHz, CDCl$_3$)

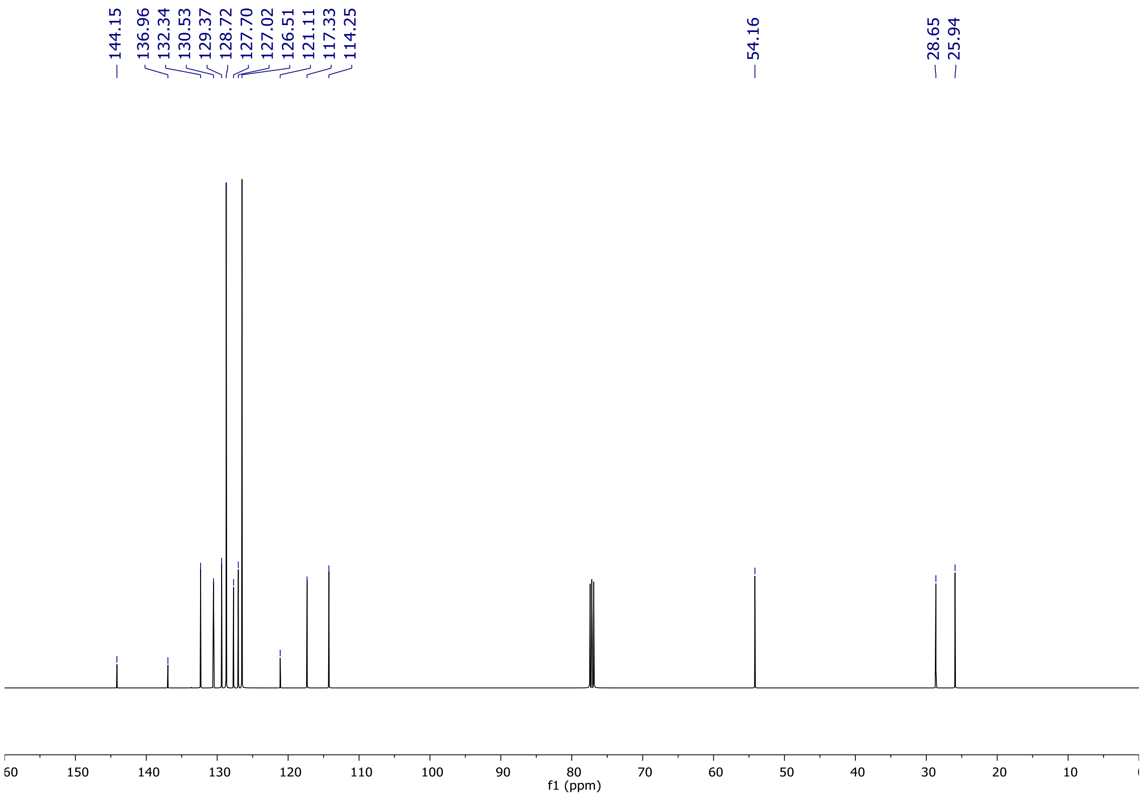

^{1}H NMR (500 MHz, $CDCl_3$)

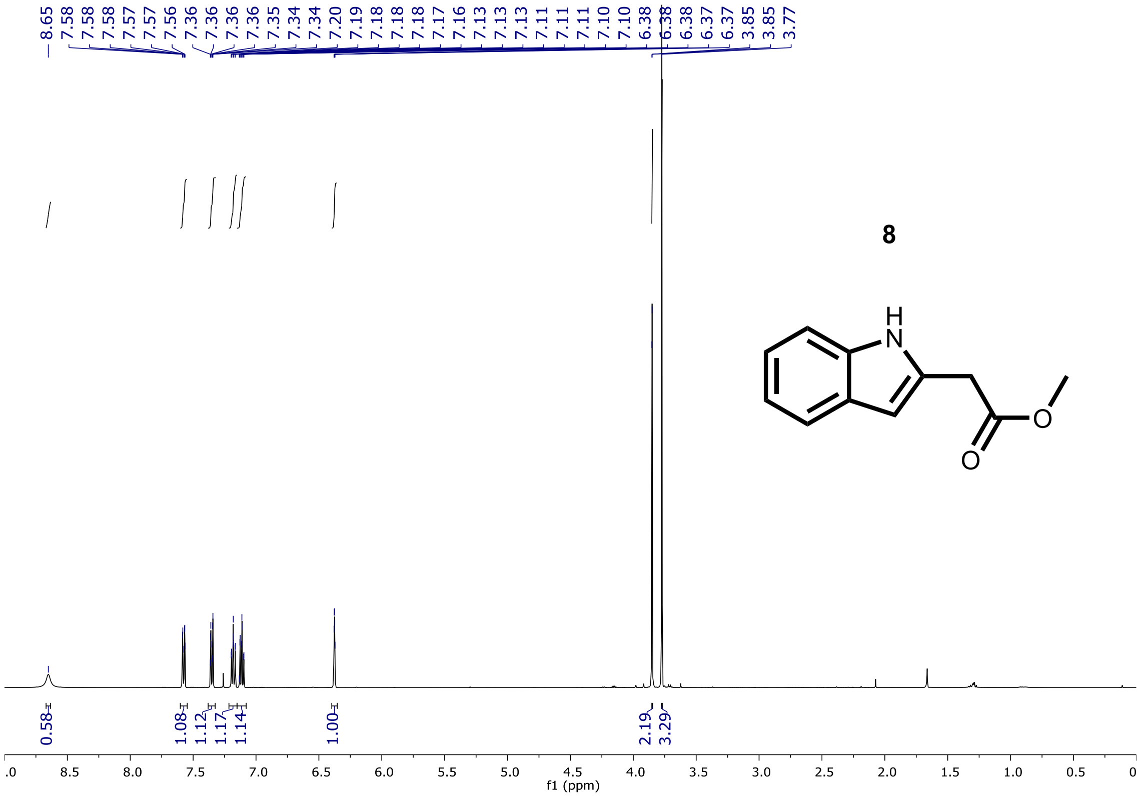

DEPT-135

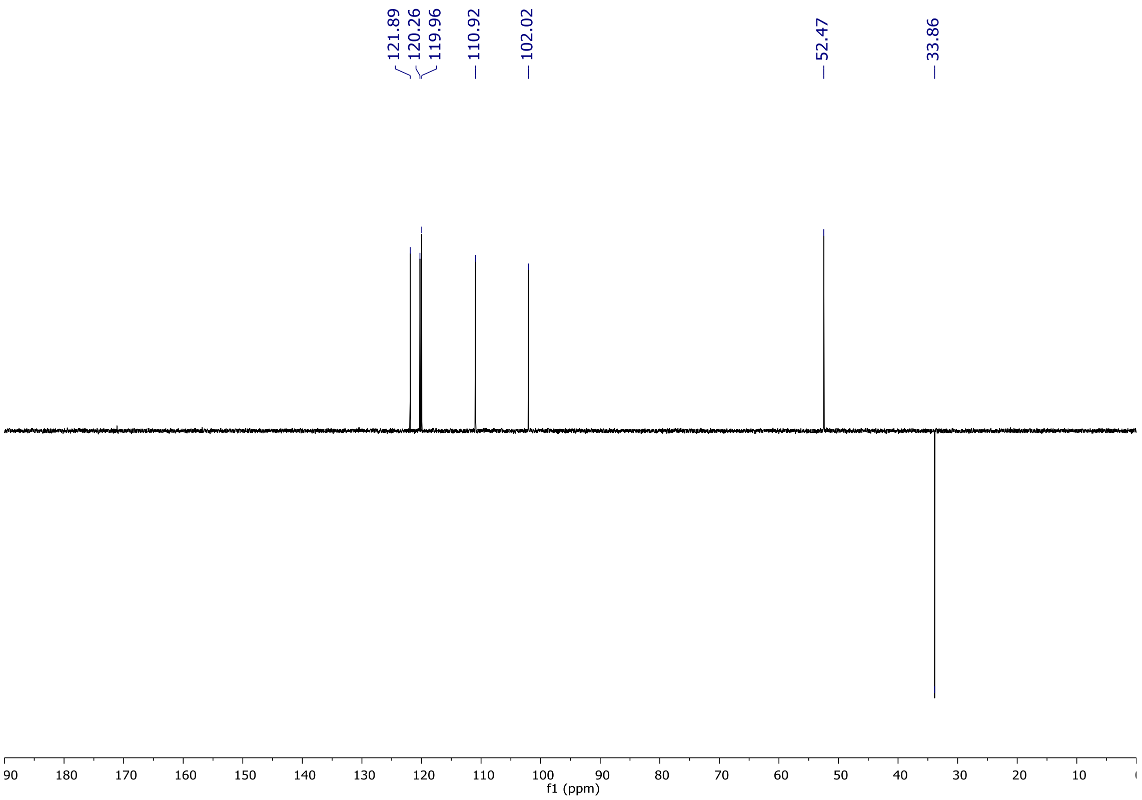

^{13}C NMR (126 MHz, $CDCl_3$)

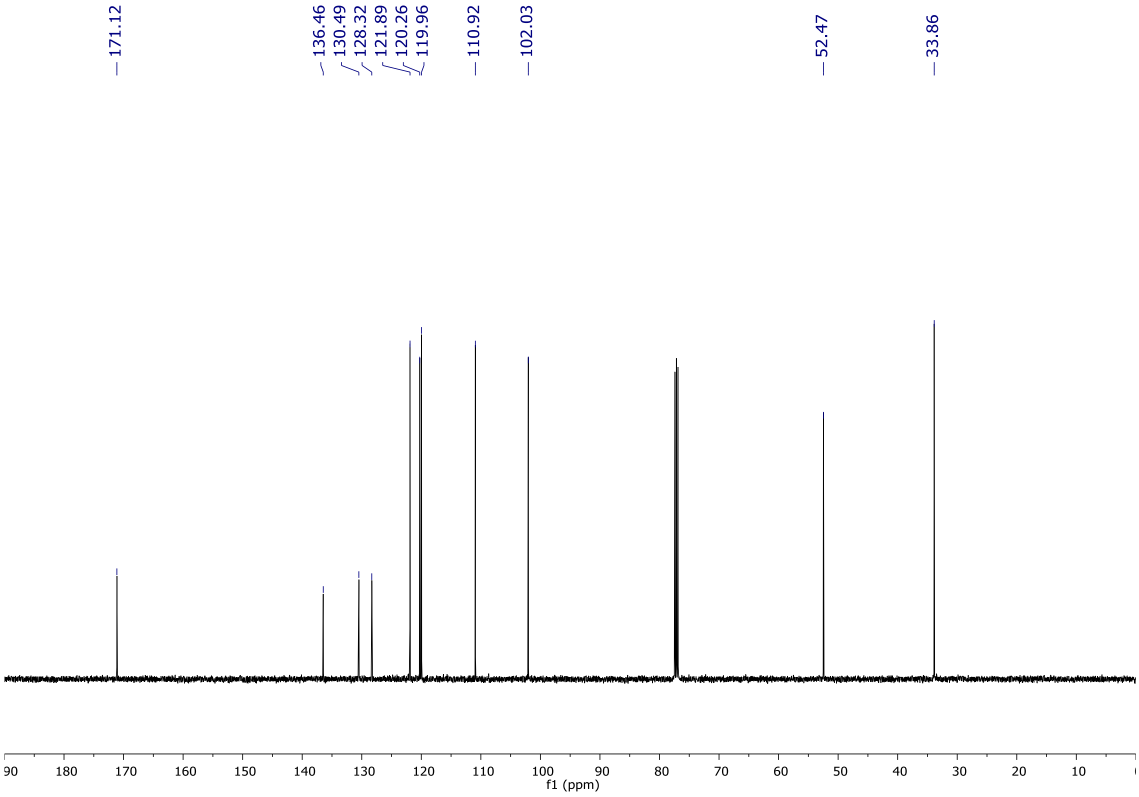

Supplement: Supplementary file 1 [file ol4c02292_si_001.pdf]
